# Structure-Dependent Cytotoxic and Cytoprotective Effects of Molybdenum(VI) Schiff Base Complexes in the Neuronal Cells In Vitro

**DOI:** 10.3390/molecules31162883

**Published:** 2026-08-18

**Authors:** Olga Tovchiga, Magdalena Narajczyk, Kornelia Kozłowska-Wysocka, Paweł Niedziałkowski, Grzegorz Romanowski, Iwona Inkielewicz-Stępniak

**Affiliations:** 1 Department of Pharmaceutical Pathophysiology, Medical University of Gdańsk, Dębinki 7, Building 27, 80-211 Gdańsk, Poland; olga.tovchiga@gumed.edu.pl; 2 Laboratory of Electron Microscopy, Faculty of Biology, University of Gdańsk, Wita Stwosza 59, 80-308 Gdańsk, Poland; magdalena.narajczyk@ug.edu.pl; 3 Faculty of Chemistry, University of Gdańsk, Wita Stwosza 63, 80-308 Gdańsk, Poland; kornelia.kozlowska@phdstud.ug.edu.pl (K.K.-W.); pawel.niedzialkowski@ug.edu.pl (P.N.)

**Keywords:** molybdenum, Schiff base complex, cytotoxicity, cytoprotection, antioxidant activity

## Abstract

Coordination compounds with transition metals, especially of molybdenum, are promising cytoprotective agents. In this work, we analyzed the changes in HT-22 cell viability under the influence of molybdenum(VI) Schiff base complexes per se or in cellular injury induced by hydrogen peroxide (annexin V and propidium iodine staining assay). The results allowed to identify the most active compound with dioxidomolybdenum(VI) ion coordinated to Schiff base derived from *1S*,*2S*-(+)-2-amino-1-(4-nitrophenyl)-1,3-propanediol and 5-methoxysalicylaldehyde, protecting the cells and possessing low cytotoxicity. We addressed a range of active concentrations (statistically significant effect from 5 µM to 50 µM) and the temporal characteristics of the effect (which is evident in 1-h preincubation regimen or adding the compound 1 h after hydrogen peroxide). Importantly, the effect was present at different levels of glucose in the medium and in cellular injury induced by glutamate. Less pronounced protective effect was seen in SH-SY5Y cells on the model of hydrogen peroxide-induced (but not in glutamate-induced) cytotoxicity. Among the possible mechanisms of cytoprotective activity, there is a direct interaction with hydrogen peroxide (measured by cyclic voltammetry), decrease in reactive oxygen species generation (DCFH-DA assay in HT-22 cells) and maintenance of catalase activity (assessed in lysates of HT-22 cells on the model of hydrogen peroxide-induced injury).

## 1. Introduction

Coordination compounds of metals ensure the basic processes of life. Thus, they remain within the focus of attention of pharmacologists. Together with the possibility to target the crucial mechanisms within the organism, such compounds enable a fundamental decrease in the toxicity of the metals compared with the correspondent inorganic metal salt toxicity (as well as with the organic ligand toxicity) [1,2,3].

Being an essential element for microorganisms, plants, and animals [3,4], molybdenum is of interest in the context of drug discovery, and the possible applications and capabilities of molybdenum-based metallodrugs have been profoundly summarized [5]. The important fields of such compounds use include anti-cancer and anti-diabetic treatments [5], but are not limited to these. Indeed, metal-based drugs are considered as a new and promising alternative to treat neurodegenerative diseases [6,7,8,9]. Novel approaches extend far beyond using chelators sequestering the ions limiting redox processes or interaction with protein folding. Specifically, the evidence from in vivo models suggests that there are possibilities to correct the changes in metal distribution, cellular deficiencies, or sequestration in proteinopathies [6,7].

At the same time, the level of toxicity of molybdenum compounds is considered to be low, and the tolerable upper intake level for molybdenum set by the Institute of Medicine (US) is 2000 μg/day [10]. Neurotoxicity is not among the primary toxic effects of molybdenum complexes, which can be considered as an additional benefit [11].

Oxidative stress remains one of incessant targets in the search for neuroprotectors, and of great interest in this context are data on metal-based coordination compounds which are characterized as “superoxide dismutase/catalase biomimetic compounds.” The changes in metals’ intracellular content indicated the existence of absorption mechanisms for the uptake of these metal-based complexes. Notably, their beneficial influence on the oxidative stress markers was translated into the prolongation of the life span [12]. The important mechanisms of this action are harbouring *in vitro* catalase (CAT) and/or superoxide dismutase (SOD) activity, with the subsequent reduction in oxidative stress biomarkers and protection of *Saccharomyces cerevisiae* cells under severe oxidative stress conditions [13]. Moreover, the cited authors have shown that those metal-based coordination compounds regulated the amounts of lipid droplet occurrence, thus maintaining cell integrity during ageing. In this context, it is worth mentioning that catalase functioning in peroxisomes may not be inconsequential to the lipid metabolism [14,15]. In addition, catalase dysfunction is considered to be an important process and a target of therapeutic intervention not only in anemias or Wilson’s disease but also in neurodegenerative processes. Thus, catalase–amyloid–*β* interaction (direct and indirect) is discussed in the context of counteraction to Alzheimer’s disease pathogenesis [16].

Indeed, the function of the natural enzymes, such as catalases, superoxide dismutases, or glutathione peroxidases, has inspired scientists to synthesize manganese metal complexes (manganosalen complexes and analogues), which are able to mimic the activity of redox enzymes that may be beneficial in counteracting age-related changes (similar to the approach described above). They were shown to exert a cytoprotective effect in a number of pathological states, including oxidative stress in SH-SY5Y cells [17].

As for molybdenum, it could be mentioned that this element’s deficiency has been identified in patients with Alzheimer’s disease, and it has been linked to the shifts in copper metabolism (as a biological antagonist of molybdenum). The partially unexpected consequence of such changes is the reduced activity of xanthine oxidase (the cofactor of which is molybdenum) and the decreased uric acid levels in biological media [18]. Uric acid is an ambiguous molecule which, together with its detrimental effects, can exert a neuroprotective activity, and reduced uricemia is often found in patients with neurodegenerative diseases [19,20].

Another enzyme which is dependent on molybdenum is sulfite oxidase. If its activity is decreased because of deficiency of molybdenum cofactor, high levels of sulfites can adversely affect glutamatergic neurotransmission by inhibiting glutamate dehydrogenase and degrading subunits of NMDA receptors, with further disturbances of learning and memory [21]. Sulfite appeared to be able to disrupt the energy production by brain mitochondria and induce mitochondrial permeability transition pore opening via thiol group modification [22]. Thus, in the article [21], supplementation with sodium molybdate is discussed in the context of disease-modifying treatment in Alzheimer’s disease, together with Se-methylselenocysteine, taurine, a combination of L-arginine and L-citrulline and others (in contrast to commonly used antioxidants like vitamin E, beta-carotene, flavonoids, vitamin C, L-carnitine, or acetyl-l-carnitine, which may actually decrease the activity of molybdenum-containing enzymes) [21].

The involvement of molybdenum deficiency in the ethiology of Parkinson’s disease is substantiated in the article [23] in the light of sulfite oxidase and xanthine oxidase decreased activity. These findings are supported by historical data from the regions with severe deficit of molybdenum and from the experimental studies on sheep (which, just like humans, possess an ability to salvage purines) [23].

The interactions of metal complexes and amyloid formation are highly complex, and together with the evidence of the deleterious effects, there are data on the potentially protective activity of discrete metal complexes in Alzheimer’s disease (the mechanisms include modulation of peptide aggregation towards non-toxic species together with the improved blood–brain barrier crossing) [24]. Moreover, among these mechanisms there is a possibility to neutralize reactive oxygen species (ROS) produced by beta-amyloid aggregates, and this protective property is maintained even when metal complexes get bound to beta-amyloid. This fact correlates well with the beforementioned data on the ability of discrete metal complexes to catalytically decompose superoxide and hydrogen peroxide (in a manner similar to the primary line of biological metalloenzymes) [13]. This effect is relevant in different biological contexts linked to cytoprotection.

In the light of these data, we aimed to perform the detailed study of the previously synthesized *cis*-dioxidomolybdenum(VI) complexes with chiral tetradentate Schiff bases which demonstrated the protective activity under the conditions of H_2_O_2_-induced injury [25,26,27]. These data were obtained in HT-22 cells (a mouse hippocampal cell line that is a generally accepted model for studying neuronal cell death and oxidative stress), and a concentration-dependent effect was seen in the range of 10–100 µM with the absence of cytotoxicity signs at the protective concentrations. Thus, it was expedient to perform the further studies of these compounds’ biological activity in neuronal cells *in vitro*.

Since pharmacological analysis presupposes the use of reference substances with the known mechanism of action, cyanocobalamin was chosen for this purpose. The reasons for this choice were as follows. Cyanocobalamin was selected as a coordination compound of metal with demonstrated neurotropic activity, including a neuroprotective one. As described above, metal-based coordination compounds may be characterized as “SOD/catalase biomimetic compounds,” which is of interest for the studied compounds, and specifically cyanocobalamin reacts with superoxide at rates approaching SOD [28]. In the context of the study, it is important that this compound was found able to protect SH-SY5Y cells from H_2_O_2_-induced oxidative stress [29], and in retinal ganglion cells its protective activity was linked to superoxide scavenging and was registered at a wide range of concentrations 10 nM to 1 mM [30].

The question of an active moiety and estimation of the role of the metal centre in biological activity is of high significance. Nevertheless, as stated above, the ability to mimic the function of the enzymes of antioxidant defence in inherent in coordination compounds of metals and hardly can be expected of their inorganic salts. Moreover, it is known that inorganic molybdenum substances release the molybdate ion [MoO_4_]^2−^ under physiological conditions as the only toxicologically relevant species [31]. Also, it has been shown that sodium molybdate at a concentration of 10 µM (which is of interest for our studies) increased production of ROS despite the inhibition of the ROS-producing molybdenum enzyme xanthine oxidase in HepG2 and HEK293 cells (in HepG2 cells, it so inhibited phosphatase activity) [32]. Proceeding from the role of the decreased xanthine oxidase activity (which arises in deficiency of molybdenum) in the development of neurodegenerative processes (in details described above), inhibition of this enzyme was not considered advantageous in the context of the current study (although it can be beneficial in other biological contexts). The main disadvantage, however, is the ability of molybdate to increase ROS level and low probability of positive influence on the system. Based on these data, molybdate inorganic salts were not used as reference substances.

The following objectives were formulated:

1. To elucidate the efficacy of the studied compounds on a model of hydrogen peroxide-induced cellular injury and to evaluate the cytotoxicity of the compounds per se, thereby estimating the safety and efficacy of the studied concentrations. These results served as the basis for the selection of the most effective compound and its concentration for further studies;
2. To determine the range of effective concentrations of the chosen compound;
3. To compare the protective activity of the chosen compound with the reference substance which is also a metal-based coordination compound with demonstrated cytoprotective activity;
4. To investigate the influence of the incubation regimen (preincubation vs. post-treatment/adding after the cytotoxic agent) on the efficacy of the chosen compound and to assess this efficacy at earlier time points;
5. To evaluate the realization of the protective activity of the chosen compound under the different glucose concentrations in media (namely, 5.55 mM; 25 mM; 50 mM);
6. To estimate the manifestation of the protective activity of the chosen compound on a model of cellular injury of different pathogenesis (namely, glutamate-induced cytotoxicity);
7. To evaluate the protective activity of the chosen compound using another cell line (namely, a human neuroblastoma cell line SH-SY5Y) on the model of H_2_O_2_-induced cellular injury and glutamate-induced cytotoxicity;
8. To characterize the probable mechanisms of the cytoprotective activity of the chosen compound, namely:

8.1. To study the direct interaction with hydrogen peroxide (by cyclic voltammetry);

8.2. To estimate the total reactive oxygen species (ROS) generation within HT-22 cells using 2′,7′-dichlorodihydrofluorescein diacetate (DCFH-DA);

8.3. To characterize the changes in catalase and SOD activity in HT-22 cells.

## 2. Results and Discussion

### 2.1. Cytoprotective and Cytotoxic Activity of Cis-Dioxidomolybdenum(VI) Schiff Base Complexes Compared with Cyanocobalamin in HT-22 Cells

In the initial phase, cell viability was analyzed after preincubation with the studied compounds **[(+)MoO_2_(HL^5^)]**, **[(+)MoO_2_(HL^1^)(CH_3_OH)]**, **[MoO_2_(HL^3^)]**, **and [MoO_2_(HL^6^)]**, **[MoO_2_(HL^5^)]** on the model of hydrogen peroxide-induced cytotoxicity [33] and cytotoxicity of the compounds per se was also assessed, in both cases after 48 h of incubation.

**Figure 1:**
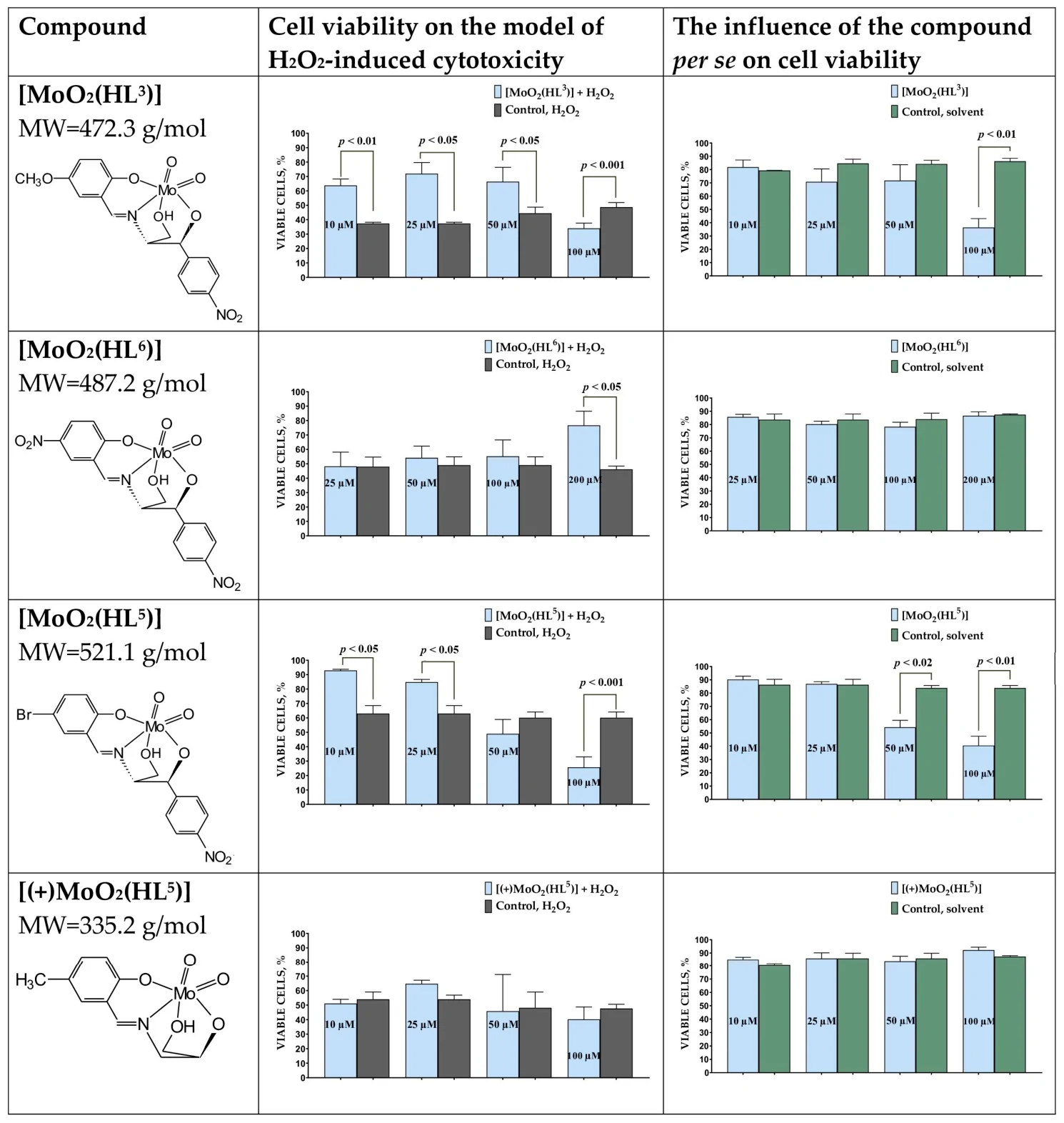

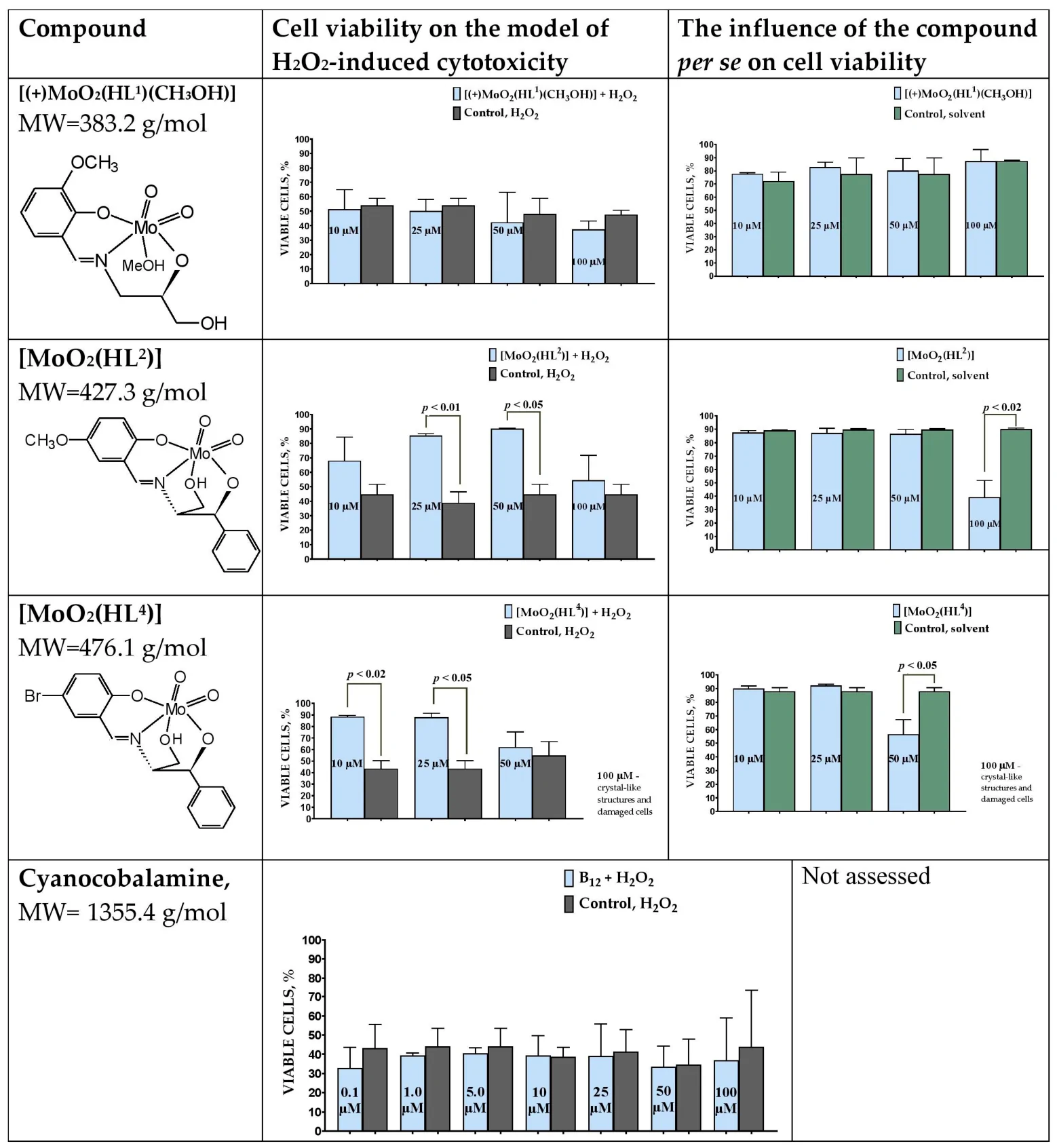
The effects of **[(+)MoO_2_(HL^5^)]**, **[(+)MoO_2_(HL^1^)(CH_3_OH)]**, **[MoO_2_(HL^3^)]**, **[MoO_2_(HL^6^)]**, **[MoO_2_(HL^5^)]**, **[MoO_2_(HL^2^)]**, **[MoO_2_(HL^4^)]** and cyanocobalamin on HT-22 cells viability per se and under the conditions of H_2_O_2_-induced cytotoxicity (500 µM, 48 h). The Y-axis shows the cell survival (percentage of the viable cells not undergoing necrosis and apoptosis). Data represent the mean ± SEM from at least three (3 to 4) independent biological replicates. Statistical significance of the differences compared with the values of the respective control group is indicated on each graph (*p* < 0.05 or less).

Under the conditions of hydrogen peroxide-induced cytotoxicity, **[MoO_2_(HL^5^)]**, **[MoO_2_(HL^3^)]**, and **[MoO_2_(HL^6^)]** exhibited statistically significant protective activity (Figure 1), and for **[MoO_2_(HL^6^)]** that was reached only after concentration was increased up to 200 µM (and confirmed by the data on microphotographs, Supplementary Files S1 and S2). Since the concentrations 50 and 100 µM showed only a tendency towards a protective effect, additional experiments were done for the concentration of 200 µM, for this reason there are differences in the studied range of concentrations between this one and other compounds (Figure 1). For this compound, in contrast to **[MoO_2_(HL^5^)]** and **[MoO_2_(HL^3^)]**, no signs of significant cytotoxicity were seen at concentrations up to 200 µM (Figure 1, microphotographs in the Supplementary File S2).

For **[MoO_2_(HL^5^)]**, protective effect was registered at concentrations 10 and 25 µM, the effect was absent at a concentration of 50 µM and significant decrease in cell viability was seen at a concentration of 100 µM (Figure 1). The microphotographs (Supplementary File S1, Section 1) indicate the inverse relationship between the concentration and protective activity. Moreover, significant cytotoxicity was seen at concentrations 50 and 100 µM (Figure 1); thus, it was the only one from these five compounds studied revealing the significant cytotoxicity at a concentration of 50 µM (that is also visible on microphotographs, Supplementary File S3, Section 2).

**(+)MoO_2_(HL^5^)]** and **[(+)MoO_2_(HL^1^)(CH_3_OH)]** did not demonstrate a statistically significant protective activity on the model of hydrogen peroxide-induced cytotoxicity. Variability was seen in the results for **[(+)MoO_2_(HL^5^)]**, and the final results indicated no significant effect even for the concentration of 25 µM (Figure 1) and neither is such effect visible on the microphotographs (Supplementary File S1, Sections 3 and 4). In the paper [25] these concentrations showed the close results with regard to the quantity of viable cell against the background of more significant cytotoxic effect of hydrogen peroxide. In addition, the measurement in this work was done at earlier time points (namely, after 24 h of incubation).

**[MoO_2_(HL^3^)]** influence on cell viability was concentration-dependent and also dependent on experimental conditions. It has shown a considerable protective activity at concentrations of 10 µM, 25 µM, and 50 µM, whereas subsequent increases in concentration led to a relative decline in cell survival (Figure 1). A reversal of the antioxidant effect to the prooxidant one is generally known phenomenon [34]. This concentration of 100 µM per se also significantly decreased the viability of the studied cells under standard conditions (without any cytotoxic agents; Figure 1), while at the lowest concentration no influence on cell survival was seen, and certain variability was observed at 25 and 50 µM.

**[MoO_2_(HL^4^)]** showed a significant protective activity at concentrations of 10 and 25 µM on the model of hydrogen peroxide-induced cellular injury (Figure 1, microphotographs in the Supplementary File S1, Section 5). However, at a concentration of 50 µM the effect was not seen and at a concentration of 100 µM it was reversed to a cytotoxic one; in these samples a plethora of the severely damaged cells and cell debris was seen (Supplementary File S1, Section 5). The compound at these concentrations, especially at the highest one, formed crystal-like structures which were visualized among severely damaged cells. The same picture was registered in the samples treated with the **[MoO_2_(HL^4^)]** per se, excluding the possibility of flow cytometry conducted in these samples. Moreover, the significant decrease in cell viability was registered against the background of this compound at a concentration of 50 µM (Supplementary File S3, Section 5). This compound was excluded from the further studies. It should be noted that the crystal-like structure formation was seen for the compound **[MoO_2_(HL^4^)]** only at higher concentrations. Neither did the rest of the compounds demonstrate such ability, while they were routinely observed during the experiment and additionally the microphotographs were taken at the defined terms (Supplementary Files S3 and S4).

Thus, Br-containing compounds demonstrated the most expressed toxicity (the results concerning **[MoO_2_(HL^5^)]** are described above) and were thus not prospective for further studies.

Since cyanocobalamin is a well-studied compound with confirmed level of safety, its effects per se were not analyzed in the current study. Under the conditions of hydrogen peroxide influence, cyanocobalamin was studied at concentrations similar to that of the studied compounds, namely 0.1 µM, 1 µM, 5 µM, 10 µM, 25 µM, 50 µM, and 100 µM. As evident from Figure 1, it did not exert any protective activity.

These findings are in correspondence with the data [35] obtained in brain endothelial cells on the model of exogenous hydrogen peroxide-induced injury. In this work the absence of cyanocobalamin protective activity (cell viability was measured 24 h after hydrogen peroxide addition) was partially attributed to the dissimilarity in compartmentalization of hydrogen peroxide and cyanocobalamin and/or to the differences in the concentrations of these substances used in the study (micromoles vs. nanomoles), as well as a non-sufficient uptake of the vitamin by the cells (but nevertheless the protective effect was absent in case of preincubation that is in agreement with our results). While cyanocobalamin effectively reduced O_2_^•−^, being protective on the model of ischemia/reperfusion in vivo [36], direct cytotoxicity of O_2_^•−^ is supposed together with its conversion to hydrogen peroxide; this latter step appeared not to be targeted by cyanocobalamin and neither did it modify hydrogen peroxide-induced changes in the expression of genes important for antioxidant defence such as Nrf2 and HO-1 [35].

**Figure 2:**
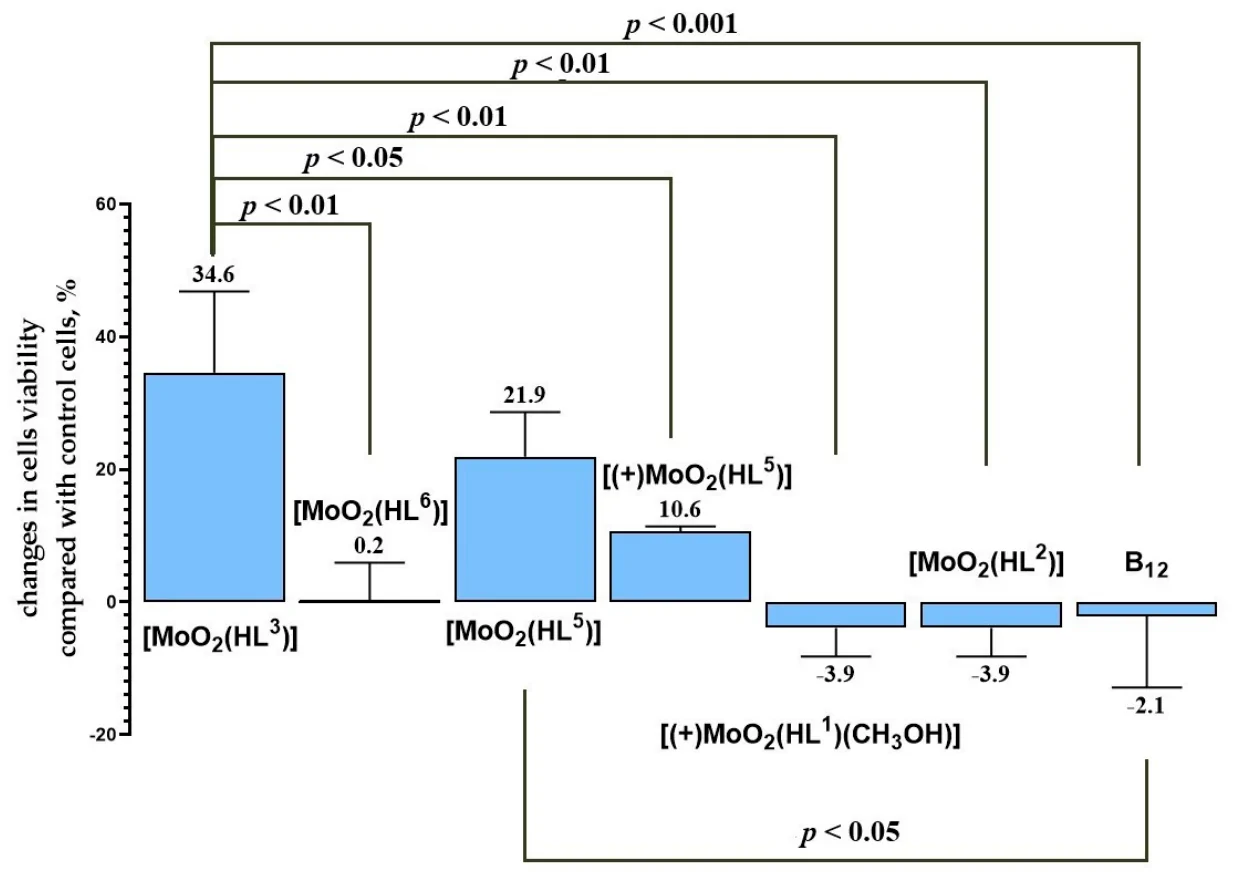
Comparison of the effects of **[(+)MoO_2_(HL^5^)]**, **[(+)MoO_2_(HL^1^)(CH_3_OH)]**, **[MoO_2_(HL^3^)]**, **[MoO_2_(HL^6^)]**, **[MoO_2_(HL^5^)]**, **[MoO_2_(HL^2^)]** and cyanocobalamin at a concentration of 25 µM on HT-22 cells viability under the conditions of H_2_O_2_-induced cytotoxicity (500 µM, 48 h). The Y-axis shows the difference in cell viability compared with the value of the control group (untreated cells injured with hydrogen peroxide). Data represent the mean ± SEM from at least three (3 to 4) independent biological replicates. One-way analysis of variance (ANOVA) with Tukey’s post hoc test was used to estimate intergroup differences. Statistical significance of the intergroup differences is indicated (*p* < 0.05 or less).

To avoid the problem of sequential comparisons, the direct comparison of all studied compounds’ protective effect (as mentioned before, compound **[MoO_2_(HL^4^)]** was excluded because of crystal-like structure appearance) at a concentration of 25 µM was done (namely, the single dataset was formed for the values representing the difference in cell viability compared with the value of the control group (untreated cells injured with hydrogen peroxide)). The results are depicted in Figure 2. They have confirmed that **[MoO_2_(HL^3^)]** demonstrates significant protective activity surpassing all the other groups except for the group of **[MoO_2_(HL^2^)]**.

As evident from Figure 1, **[MoO_2_(HL^2^)]** also displayed protective activity and favourable safety profile, comparable to that of **[MoO_2_(HL^3^)]**. No signs of cell viability decrease were seen at concentrations 10, 25, and 50 µM, and a protective activity at cytotoxic conditions was registered at two higher concentrations (Figure 1). Thus, it was necessary to compare the profile of cytoprotective concentrations for **[MoO_2_(HL^3^)]** and **[MoO_2_(HL^2^)]**, namely the lower limits of the active concentrations.

**Figure 3:**
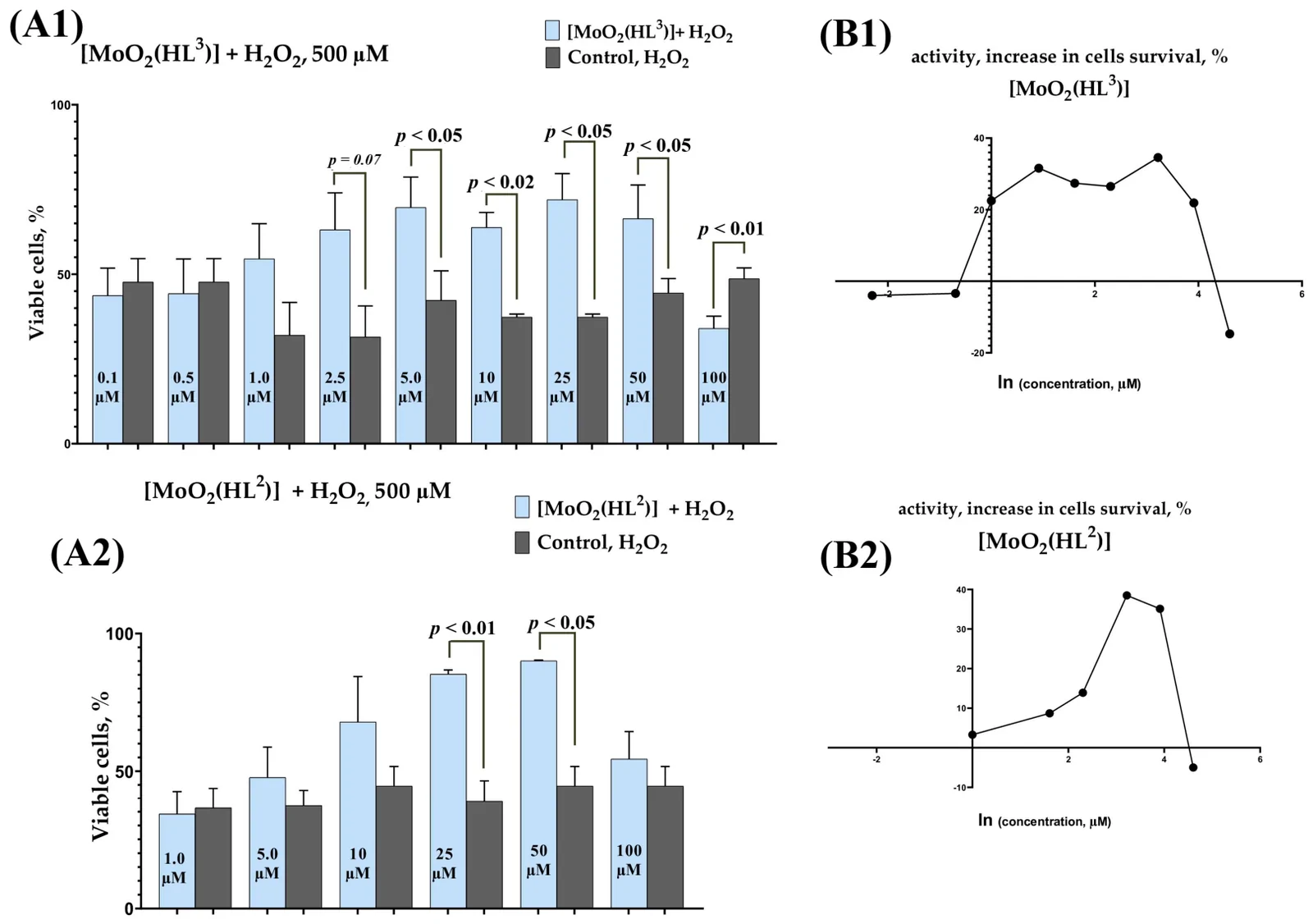
Comparison of the effects of **[MoO_2_(HL^3^)]** and **[MoO_2_(L^2^)]** on HT-22 cells viability under the conditions of H_2_O_2_-induced cytotoxicity (500 µM, 48 h). (**A1**,**A2**). The Y-axis shows the cell survival (percentage of the viable cells not undergoing necrosis and apoptosis). Data represent the mean ± SEM from at least three (3 to 5) independent biological replicates. Statistical significance of the differences compared with the values of the respective control group (untreated cells injured with hydrogen peroxide) is indicated on each graph (*p* < 0.05 or less). (**B1**,**B2**). Concentration–response curves for **[MoO_2_(HL^3^)]** and **[MoO_2_(HL^2^)]** with the X-axis being the logarithm of the concentration and the Y axis being response, namely percent increase in cell survival.

As shown in Figure 3, the statistically significant cytoprotective effect was seen at a concentration of 25 µM for **[MoO_2_(HL^2^)]**, while for **[MoO_2_(HL^3^)]**, it was seen at a concentration of 5 µM. There were no signs of cytotoxic effects when the cells were incubated with **[MoO_2_(HL^3^)]** or **[MoO_2_(HL^2^)]** per se at concentrations lower than 10 µM (Supplementary File S8). Concentration–response curves for **[MoO_2_(HL^3^)]** and **[MoO_2_(HL^2^)]** plotted using logarithm of the concentration clearly illustrate the wider therapeutic window for **[MoO_2_(HL^3^)]** (Figure 3).

Thus, these results illustrate the cytoprotective activity of **[MoO_2_(HL^3^)]**. Under the conditions of our study, it surpasses the reference substance (Figure 2) and demonstrates wide therapeutic window (Figure 3). Thus, the manifestation of the protective action at five-fold decreased concentration substantiates the choice of the **[MoO_2_(HL^3^)]** for the further studies aimed at further characteristics of this cytoprotective activity.

### 2.2. Characteristics of [MoO_2_(HL^3^)] Protective Effect Under the Conditions of Hydrogen Peroxide-Induced Cytotoxicity

**Figure 4:**
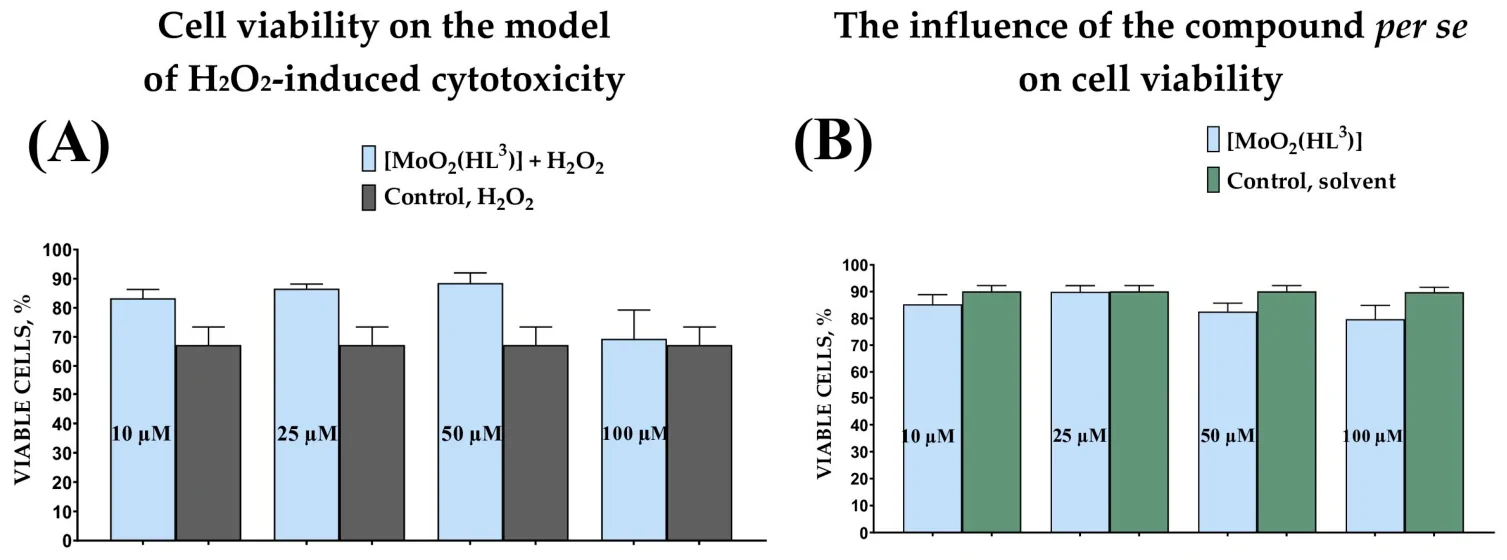
The effects of **[MoO_2_(HL^3^)]** on HT-22 cells viability after 24 h under the conditions of H_2_O_2_-induced cytotoxicity (500 µM, (**A**)) and per se (**B**) the Y-axis shows the cell survival (percentage of the viable cells not undergoing necrosis and apoptosis). Data represent the mean ± SEM from three independent biological replicates.

When the cytoprotective activity of **[MoO_2_(HL^3^)]** was measured after shorter time interval (namely, 24 h except for 48 h), the effect did not reach the level of statistical significance (Figure 4B). This shows that the protective effect develops gradually, not being limited to the early period of the experiment. Cytotoxicity signs were also not registered (Figure 4A), which is also fully consistent with the previous data obtained in MTT test with the compound per se after 24 h [26]. The data on cytoprotective effect showed the similar trend with the cited study; the latter indicated a significant protective activity against the background of more intensive cytotoxic effect of hydrogen peroxide, which is also described above.

Thus, under the conditions of the model used in the current study, the statistically significant cytoprotective effect of **[MoO_2_(HL^3^)]** (by the criteria of the increase in viable cell proportion against a background of hydrogen peroxide) was reached after 48 h only.

**Figure 5:**
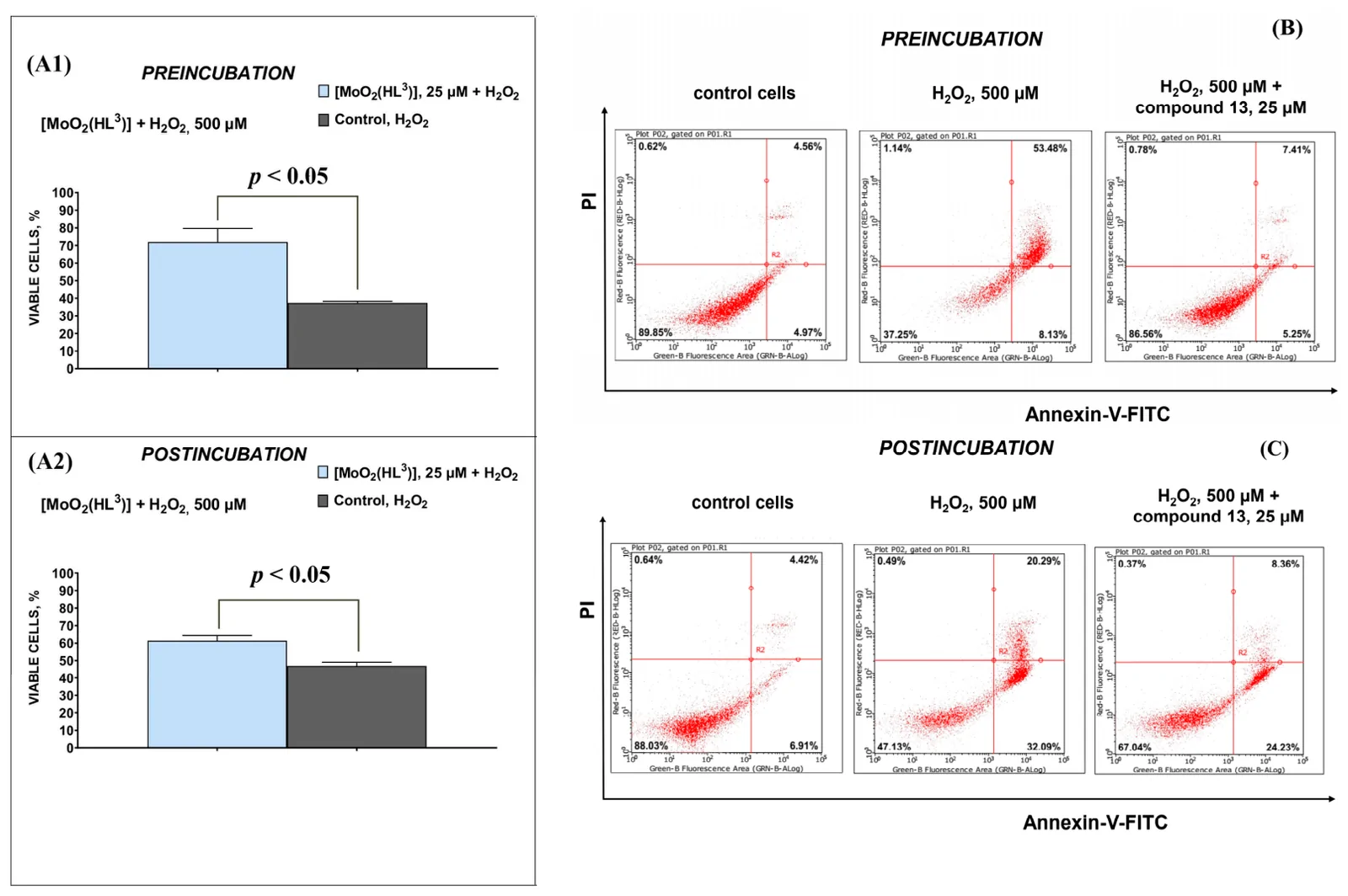
The effects of **[MoO_2_(HL^3^)]** (25 µM) on HT-22 cells viability after 48 h under the conditions of H_2_O_2_-induced cytotoxicity (500 µM); the compound was added 1 h before (“preincubation”) or 1 h after H_2_O_2_ (“postincubation” or adding after the cytotoxic agent). (**A1**,**A2**). The Y-axis shows the cell survival (percentage of the viable cells). Data represent the mean ± SEM from three independent biological replicates. Statistical significance of the differences compared with the values of the respective control group (untreated cells injured with hydrogen peroxide) is indicated on each graph (*p* < 0.05 or less). (**B**,**C**). Representative bivariate histograms of control cells, cells injured with hydrogen peroxide, and cells injured with hydrogen peroxide incubated with **[MoO_2_(HL^3^)]** (25 µM, “preincubation,” (**B**) or “postincubation,” (**C**)). PI—propidium iodide. The upper left (UL) quadrant (PI+/Annexin V−) represents necrotic cells, the left lower (LL) quadrant (PI−/Annexin V−) represents viable unchanged cells, the lower right (LR) quadrant (PI−/Annexin V+) represents early apoptotic cells, and the upper right (UR) quadrant (PI+/Annexin V+) represents late apoptotic cells.

Subsequently, the influence of the incubation regimen on the efficacy of the chosen compound was evaluated, namely the comparison of preincubation—adding 1 h before hydrogen peroxide vs. adding it 1 h after the cytotoxic agent—was done. As depicted in Figure 5, there were no statistically significant differences between these two regimens.

This provides indirect evidence that the direct inactivation of hydrogen peroxide by **[MoO_2_(HL^3^)]** is not the only mechanism of the cytoprotective effect (since in this case more significant effect could be expected after preincubation, though this requires further investigation).

**Figure 6:**
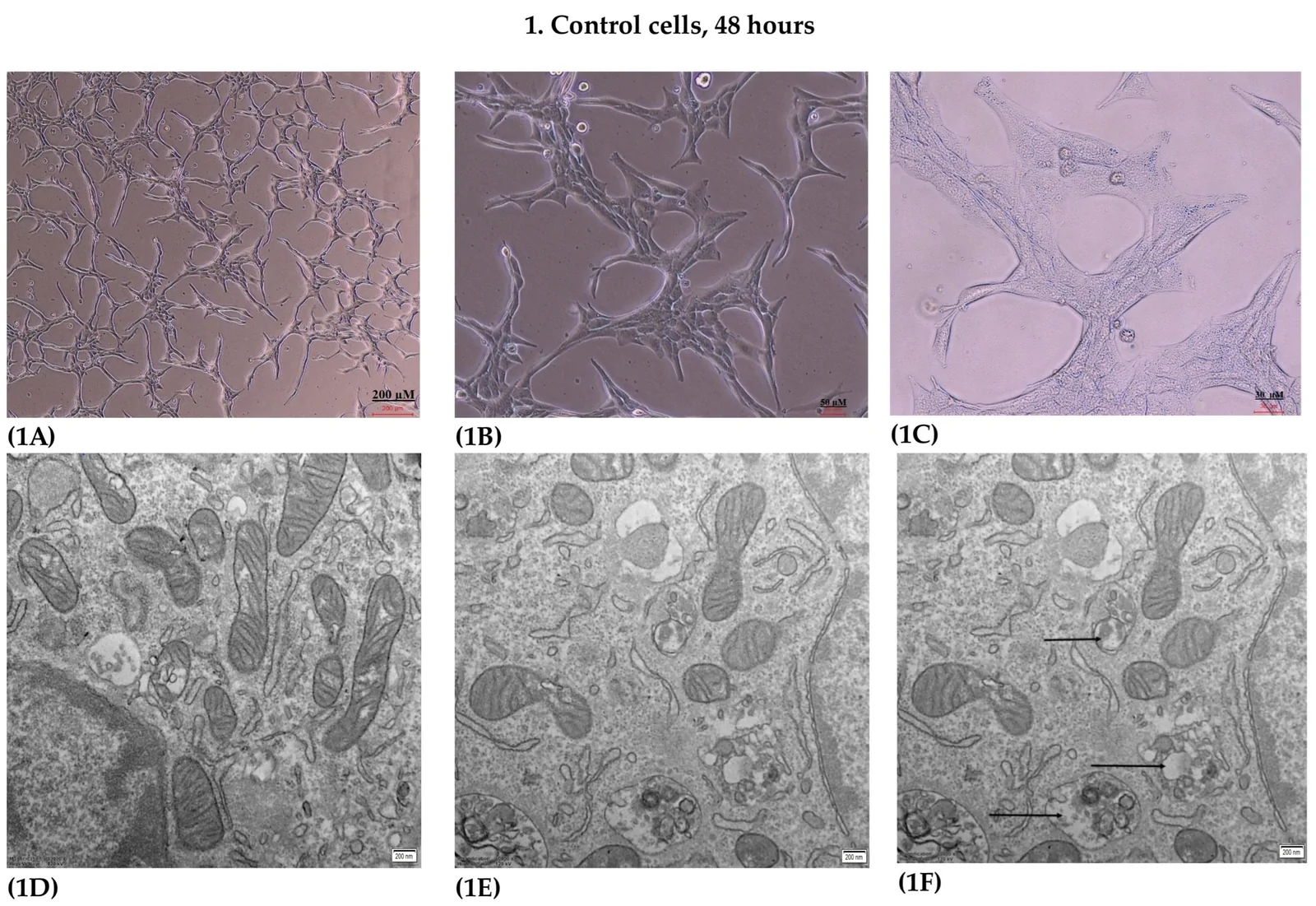

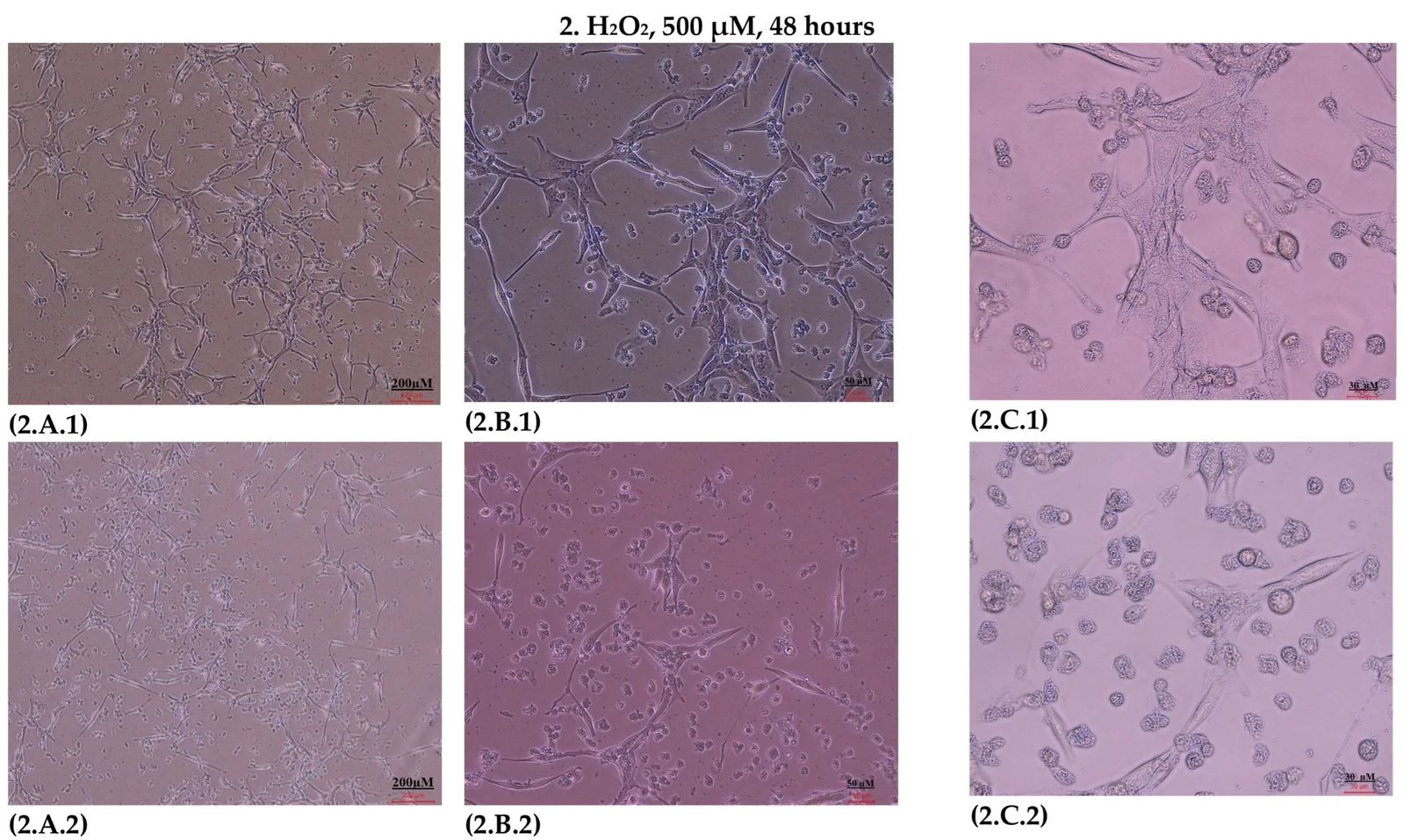

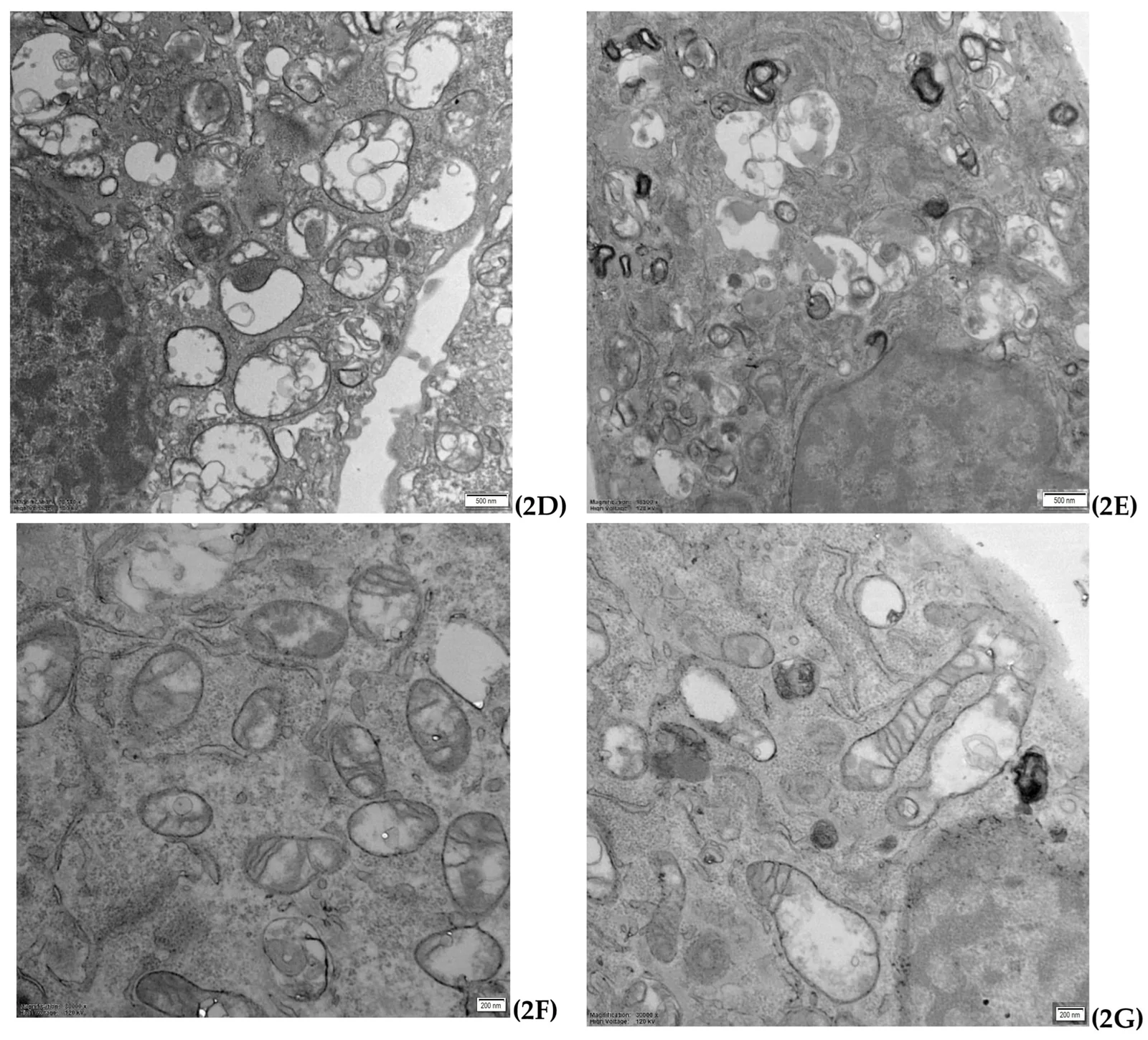

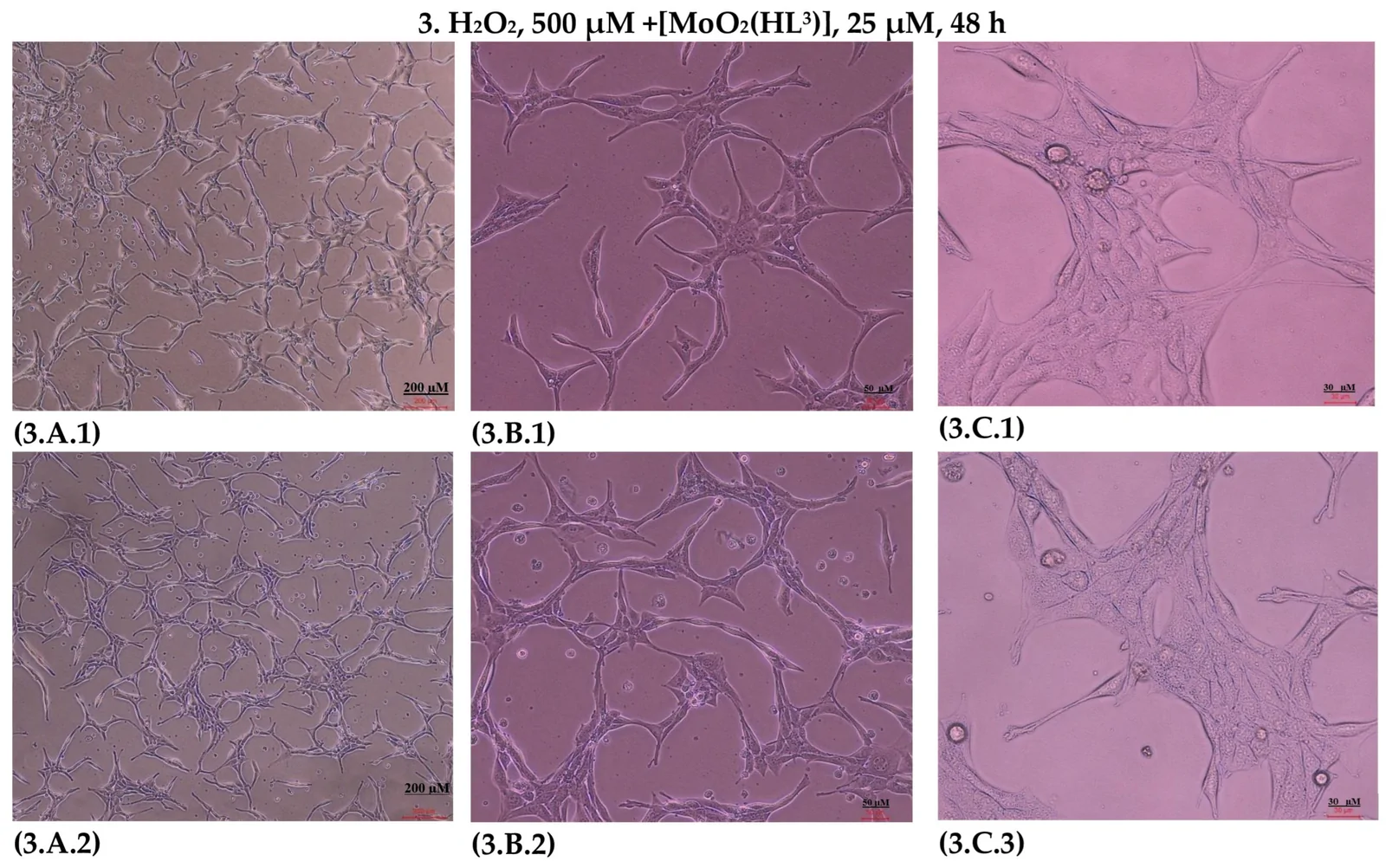

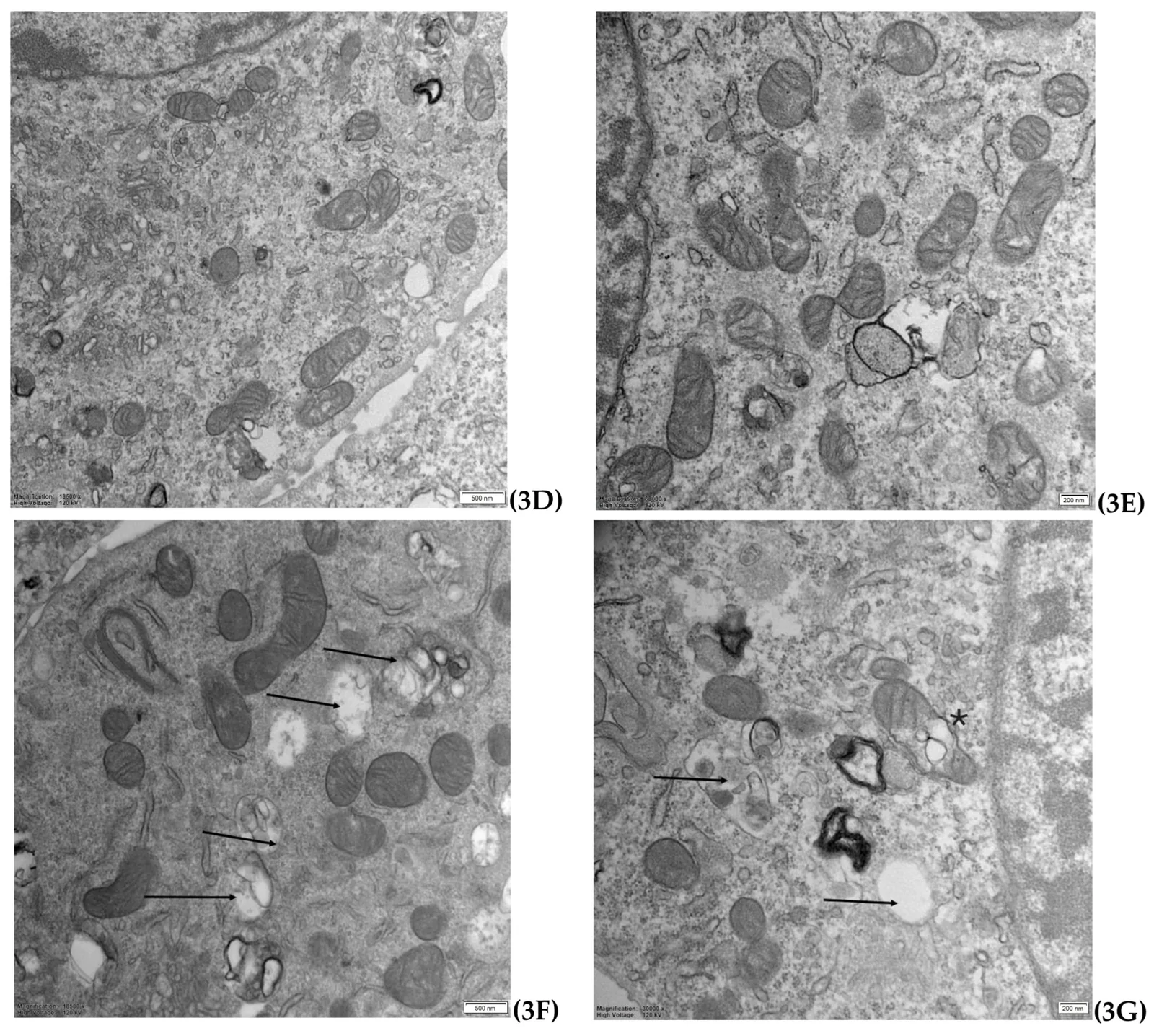
The effects of **[MoO_2_(HL^3^)]** (25 µM) on HT-22 cell structure (optical microscopy and TEM) under the conditions of H_2_O_2_-induced cytotoxicity (500 µM, 48 h). **1**. Control cells. Typical structure of the cells with the absence of visible changes in light microscopy (**1A**–**1C**). Namely the absense of the cells with the changes of shape (sporadic round-shape damaged structues are rarely seen among the layers of normal cells, (**1B**)), absense of cellular inclusions or visible changes in the membranes. In EM, the most of mitochondria are undamaged, mitochondria tubular cristae are of normal shape and size, membrane structures are without the signs of significant destruction (**1D**,**1E**). The limited quantity of autophagic lysosomes is seen ((**1F**), indicated with arrows). **2**. HT-22 cells exposed to H_2_O_2_ (500 µM, 48 h). Increased quantity of the cells with the signs of apoptosis in light microscopy (the microphotograps (**2.A.1**,**2.B.1**,**2.C.1**) depict the moderate degree of changes, the microphotograps (**2.A.2**,**2.B.2**,**2.C.2**) depict the changes of a significant severity), namely round-shaped, detached and floating cells with the changes progressing to cell debris (**2.B.2**,**2.C.1**,**2.C.2**). Significant reduction of the quantity of visible attached cells and cell layer structure. Significantly increased quantity of autophagic lysosomes visible in the entire field of view (**2D**,**2E**). Significant changes in the mitochondria structure, namely mitochondria with poorly formed cristae and matrix with the significantly decreased density (**2F**), swallowed mitochondria, matrix of which is with the significantly decreased density (**2G**). **3**. HT-22 cells exposed to H_2_O_2_ (500 µM, 48 h) pretreated with **[MoO_2_(HL^3^)]** (25 µM, in this concentration a statistically significant increase in cell viability due to the reduction of apoptotically changed cells was seen in flow cytometry). A moderate increase in the quantity of the cells with the signs of apoptosis in light microscopy (the microphotograps (**3.A.1**,**3.B.1**,**3.C.1**) depict the moderate degree of changes, the microphotograps (**3.A.2**,**3.B.2**,**3.C.2)** depict more significant changes, albeit the changes are much less prominent compared with the cells exposed to H_2_O_2_ per se as shown in the microphotograps (**2.A.1**–**2.B.3**). Only sporadic round-shaped, detached and floating cells were seen (**3.B.2**,**3.C.3**). Moderate reduction of the quantity of visible attached cells with normal cell layer structure being maintained (**3.A.1**,**3.A.2**). EM results also confirm the better condition of the cells compared with the cells exposed to H_2_O_2_ per se. Compared with the photos (**2D**–**2G**), decreased quantity of autophagic lysosomes and much less significant changes in the mitochodria, namely the maintenance of visible crista and sufficiently dense matrix (**3D**–**3F**). Still, the changed swallowed mitochondria ((**3G**), indicated with asterisk), as well as visible autophagic lysosomes were seen ((**3F**,**3G**), indicated with arrows) in certain microphotographs.

Thus, **[MoO_2_(HL^3^)]** effectively counteracted the development of apoptosis induced by hydrogen peroxide. This is confirmed by the data obtained by both optical microscopy and TEM, depicted in Figure 6.

Since hyperglycaemia and glucose metabolism dysregulation are involved in brain injury (including hippocamp injury) such as in diabetes mellitus [37,38], and glucose metabolism disorders can modulate both cytoprotective and cytotoxic processes [39,40,41], it was warranted to study the activity of **[MoO_2_(HL^3^)]** against the background of increased glucose level.

Since the previously described experiments were done using DMEM medium with low glucose content (5.55 mM, as consistent with the previous experiments [26]), in this set of experiments DMEM with high glucose content (25 mM, which can also be routinely chosen for cultivation of the cell line HT-22 cells) as well as increased glucose concentration up to 50 mM, which is considered as pathologically elevated and producing cellular injury [42] were used. Osmotic control including mannitol (25 mM + glucose, 25 mM) was also applied in this set of experiments.

**Figure 7:**
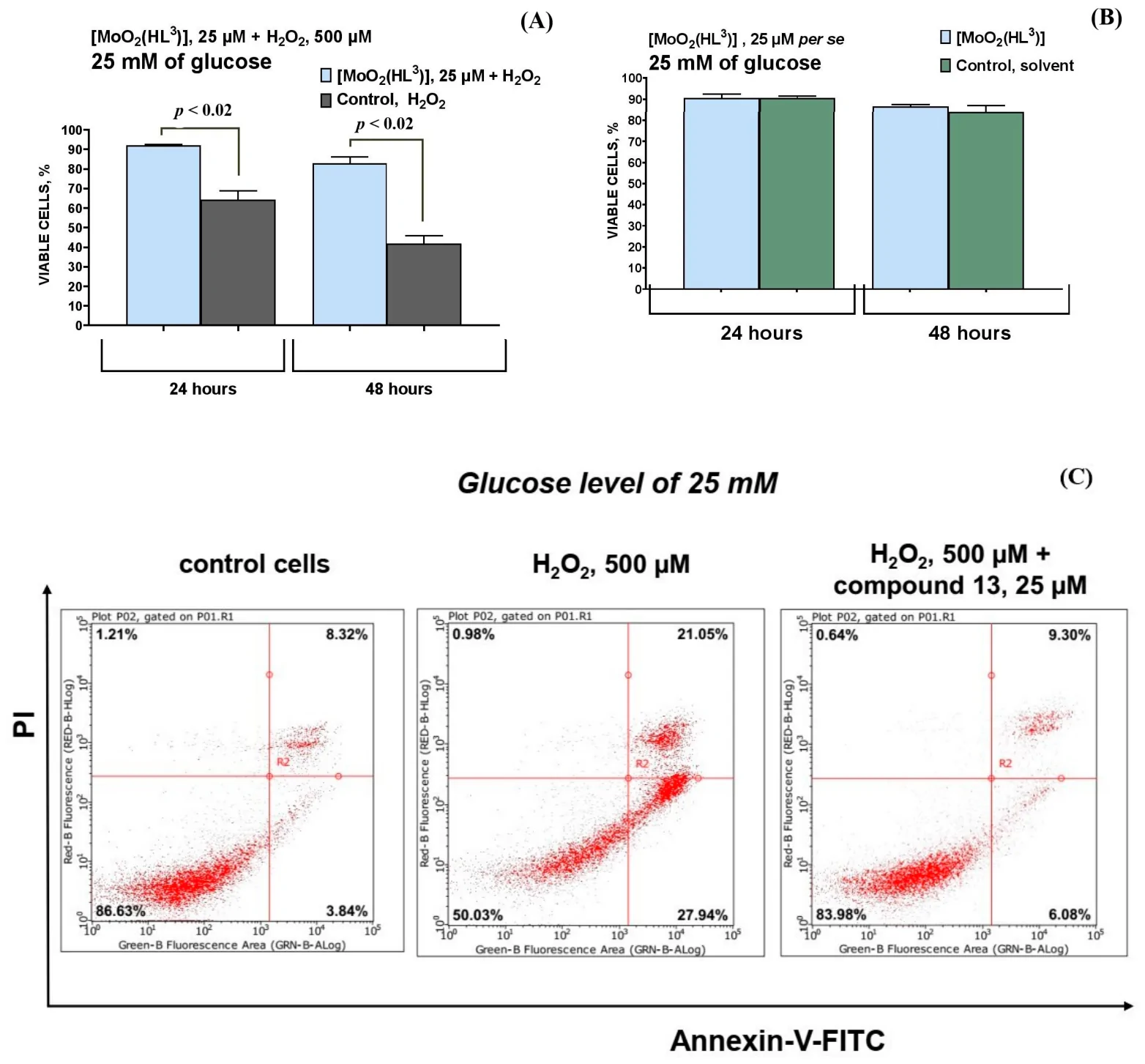
The effects of **[MoO_2_(HL^3^)]** at a concentration of 25 µM on HT-22 cells viability and under the conditions of H_2_O_2_-induced cytotoxicity (500 µM, (**A**)) and per se (**B**) in the media with glucose concentration of 25 mM (24 h and 48 h of incubation). The Y-axis shows the cell survival (percentage of the viable cells). Data represent the mean ± SEM from three independent biological replicates. Statistical significance of the differences compared with the values of the respective control group (untreated cells injured with hydrogen peroxide) is indicated on the graph (*p* < 0.05 or less). (**C**). Representative bivariate histograms of control cells, cells injured with hydrogen peroxide, and cells injured with hydrogen peroxide preincubated with **[MoO_2_(HL^3^)]** (25 µM) after 48 h of incubation, glucose concentration of 25 mM. The upper left (UL) quadrant (PI+/Annexin V−) represents necrotic cells, the left lower (LL) quadrant (PI−/Annexin V−) represents viable unchanged cells, the lower right (LR) quadrant (PI−/Annexin V+) represents early apoptotic cells, and the upper right (UR) quadrant (PI+/Annexin V+) represents late apoptotic cells.

As shown in Figure 7, **[MoO_2_(HL^3^)]** at a concentration of 25 µM did not induce any changes in cell viability. Moreover, in contrast to the previous results obtained in low-glucose medium (Figure 2), no fluctuations of cell viability were seen, and even in all of the groups analyzed cell viability differences were changed (in the direction of increase compared with the synchronous control, though this change was very moderate). These differences could possibly be attributed to more severe conditions in low-glucose medium after 48 h of incubation; in addition to serum starvation, glucose deficit might arise in these cells.

**Figure 8:**
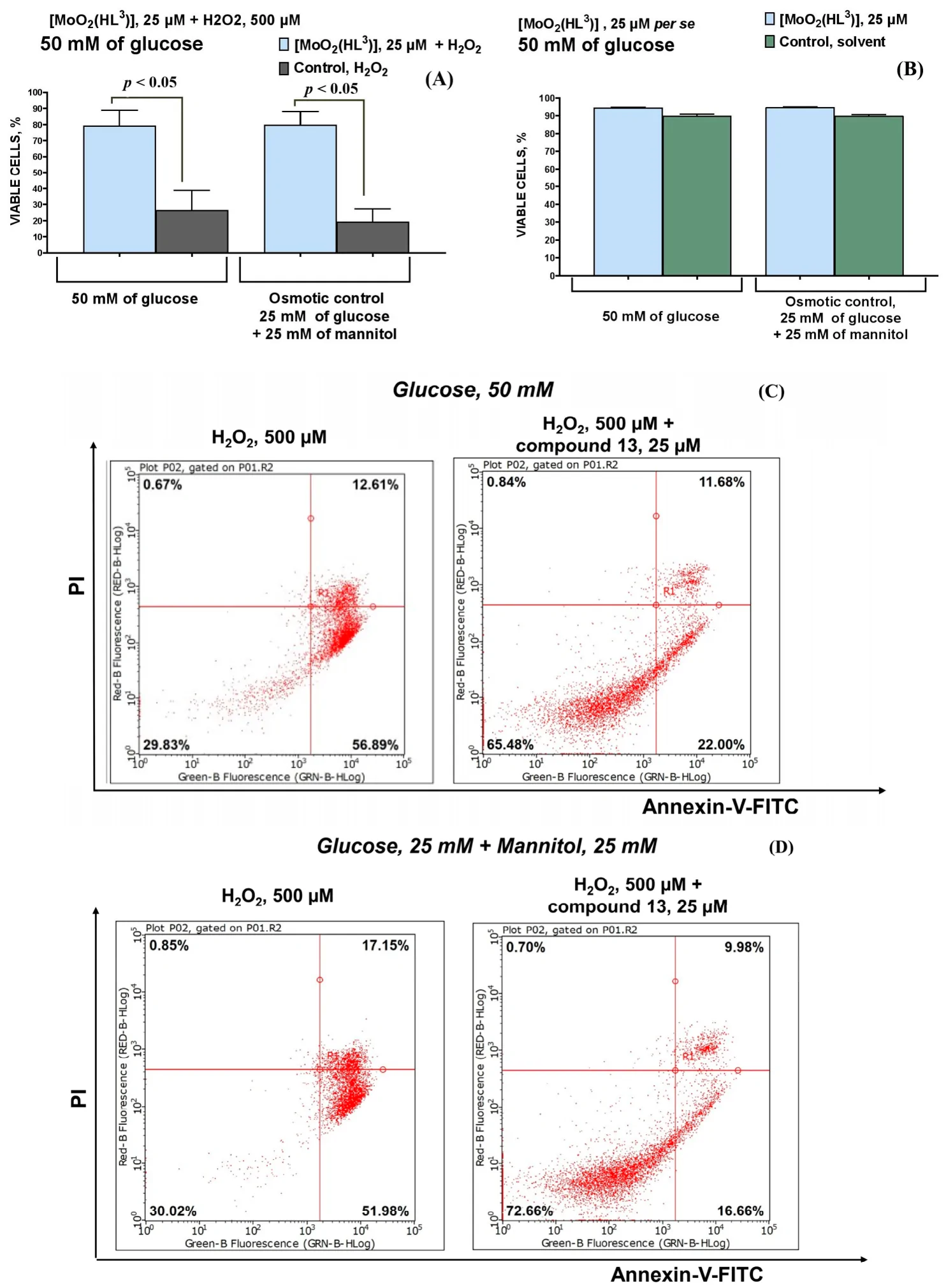
The effects of **[MoO_2_(HL^3^)]** at a concentration of 25 µM on HT-22 cells viability under the conditions of H_2_O_2_-induced cytotoxicity (500 µM, (**A**,**C**,**D**)), and per se (**B**) in the media with glucose concentration of 50 mM (48 h of incubation) and in the control medium (25 mM of glucose + 25 of mannitol), 48 h of incubation in all cases. The Y-axis shows the cell survival (percentage of the viable cells). Data represent the mean ± SEM from three independent biological replicates. Statistical significance of the differences compared with the values of the respective control group (untreated cells injured with hydrogen peroxide) is indicated on the graph (*p* < 0.05 or less) (**C**,**D**). Representative bivariate histograms of the cells injured with hydrogen peroxide, and cells injured with hydrogen peroxide preincubated with **[MoO_2_(HL^3^)]** (25 µM) after 48 h of incubation, glucose concentration of 50 mM (**C**) or osmotic control (25 mM of glucose + 25 mM of mannitol, (**D**)). The upper left (UL) quadrant (PI+/Annexin V−) represents necrotic cells, the left lower (LL) quadrant (PI−/Annexin V−) represents viable unchanged cells, the lower right (LR) quadrant (PI−/Annexin V+) represents early apoptotic cells, and the upper right (UR) quadrant (PI+/Annexin V+) represents late apoptotic cells.

In the medium with 25 mM of glucose, the cytotoxic effect of hydrogen peroxide developed to a similar extent (Figure 7) as in the low-glucose medium (Figure 1), and the protective activity of **[MoO_2_(HL^3^)]** was observed (both at 24 and 48 h). In the medium with extremely high glucose content (50 mM) as well as in the corresponding group of osmotic control, cytotoxicity of hydrogen peroxide was enhanced (Figure 8). Notably, even under these exacerbated conditions, **[MoO_2_(HL^3^)]** realized the protective effect increasing the cell viability up to 79%, which can be characterized as a significant effect.

### 2.3. Protective Effect of [MoO_2_(HL^3^)] Under the Conditions of Glutamate-Induced Cytotoxicity

**Figure 9:**
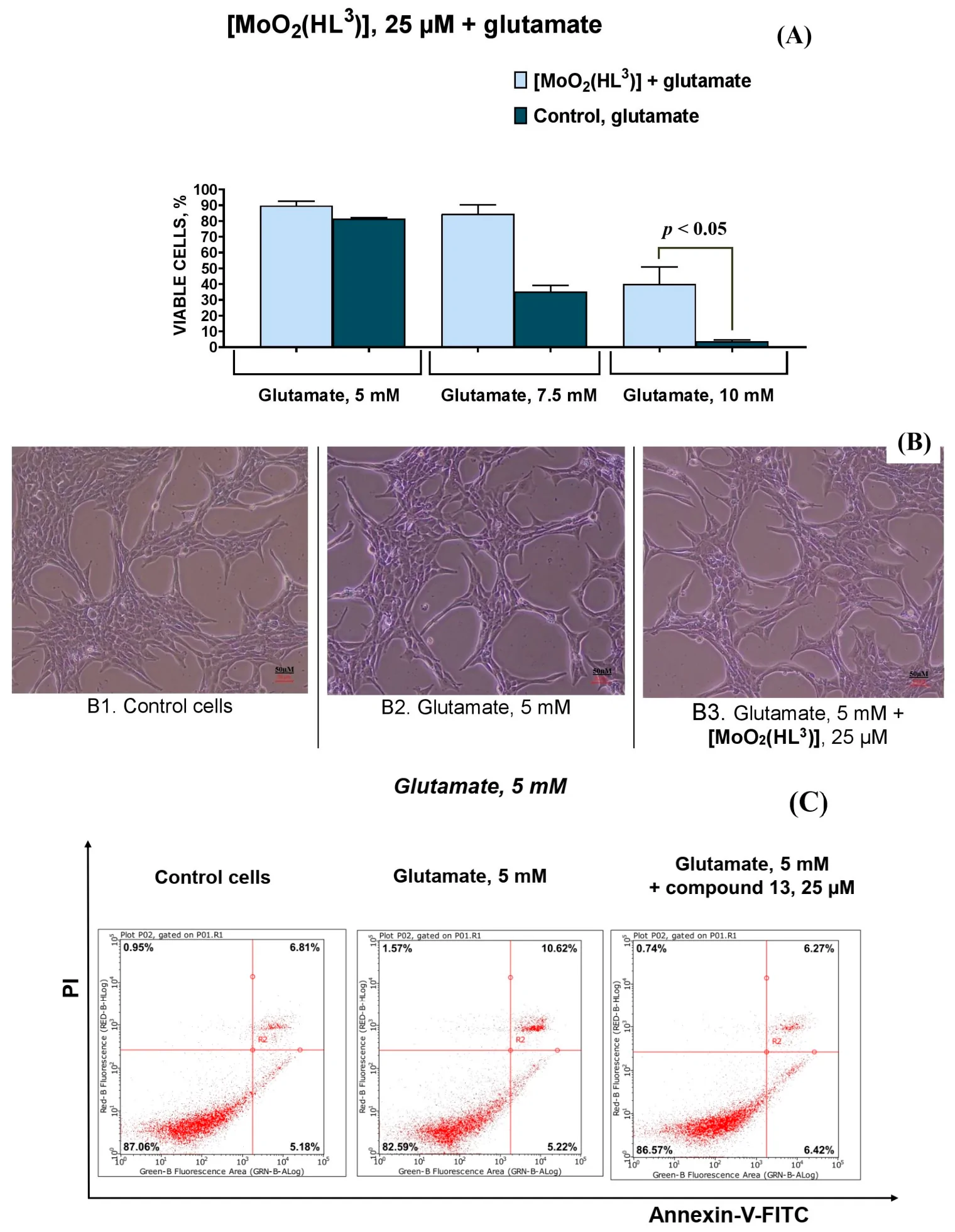

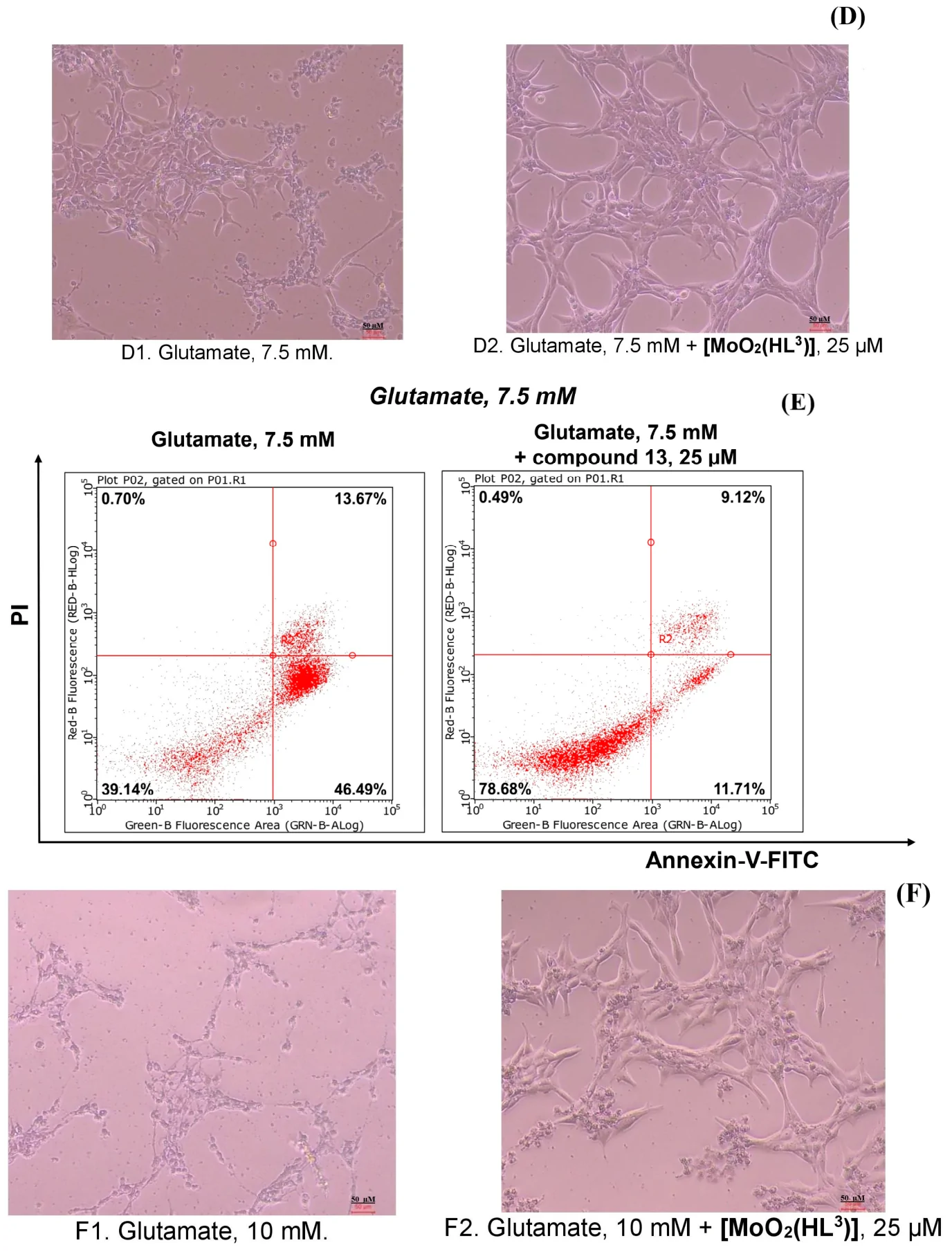

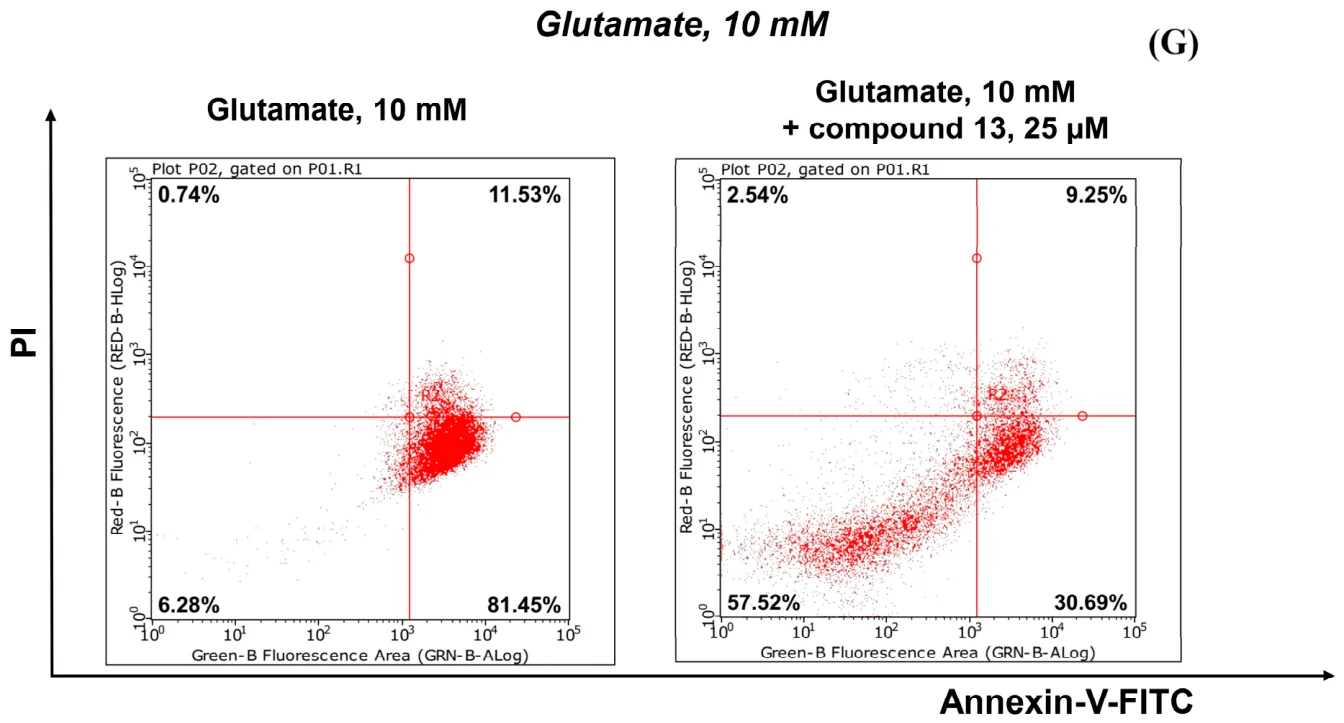
The effects of **[MoO_2_(HL^3^)] (25 µM) on HT-22 cell viability under the conditions of glutamate-induced cytotoxicity after 48 h**. (**A**). The Y-axis shows the cell survival (percentage of the viable cells). Statistical significance of the differences compared with the values of the respective control group (untreated cells injured with glutamate) is indicated on the graph (*p* < 0.05). (**B**) Representative microphotographs of HT-22 cells under the conditions of glutamate-induced cytotoxicity (5 mM) incubated with **[MoO_2_(HL^3^)]** for 48 h. (**B1**). Typical morphological structure of the control cells, which are attached—the cell shape is unchanged. (**B2**,**B3**). The typical morphological structure of the cells is maintained, the cells are attached, the cells shape is unchanged, and there are no visible changes in cell confluence compared with the microphotographs of the control cells. (**C**). Representative bivariate histograms of control cells, cells injured with glutamate (5 mM), and cells injured with glutamate (5 mM) preincubated with **[MoO_2_(HL^3^)]** (25 µM). (**D**). Representative microphotographs of HT-22 cells under the conditions of glutamate-induced cytotoxicity (7.5 mM) incubated with **[MoO_2_(HL^3^)]** for 48 h. (**D1**). Decrease in the quantity of visible attached cells and change in the shape of the cells, among which there are elongated cells. Presence of severely damaged cells which are round-shaped and detached. (**D2**). Unchanged cell confluence, change in the shape of the certain single cells, which are elongated or round-shaped. (**E**). Representative bivariate histograms of the cells injured with glutamate (7.5 mM) and cells injured with glutamate (7.5 mM) preincubated with **[MoO_2_(HL^3^)]** (25 µM). (**F**). Representative microphotographs of HT-22 cells under the conditions of glutamate-induced cytotoxicity (10 mM) incubated with **[MoO_2_(HL^3^)]** for 48 h. (**F1**). Significant reduction in the quantity of visible attached cells, change in the shape of the cells, which are elongated and fibrelike, presence of round-shaped, severely damaged cells as well as cell debris. (**F2**). Significant decrease in cell confluence, change in the shape of the cells, among which there are elongated, presence of moderate quantity of round-shaped, damaged cells and fragments of cell debris. (**G**). Representative bivariate histograms of the cells injured with glutamate (10 mM) and cells injured with glutamate (10 mM) preincubated with **[MoO_2_(HL^3^)]** (25 µM). In all histograms on this figure (**C**,**E**,**G**) the upper left (UL) quadrant (PI+/Annexin V−) represents necrotic cells, the left lower (LL) quadrant (PI−/Annexin V−) represents viable unchanged cells, the lower right (LR) quadrant (PI−/Annexin V+) represents early apoptotic cells, and the upper right (UR) quadrant (PI+/Annexin V+) represents late apoptotic cells.

At the next stage of the studies, **[MoO_2_(HL^3^)]** efficacy was evaluated under the conditions of the model induced by another cytotoxic factor, namely glutamate. It is well known that its cytotoxic effect is concentration-dependent; moreover, HT-22 cell line does not express NMDA receptors; thus, it is resistant to excitotoxicity and cellular injury to a significant degree is caused by oxidative stress [43]. Glutamate concentrations were chosen according to the data [43], and the magnitude of their cytotoxicity was consistent with previously reported data (Figure 9).

Positive effect of **[MoO_2_(HL^3^)]** in the experiments with different degrees of glutamate cytotoxicity is especially important. Namely, the compound did not exert any negative effects on cell viability against the background of glutamate concentration which did not produce cytotoxicity (5 mM) and showed a significant protective activity in the experiments in which glutamate caused practically complete cell death (10 mM). The effect against the background of glutamate at a concentration 7.5 mM/L did not reach the level of statistical significance but was registered microscopically. The microphotographs (Figure 9) confirm the dose-dependent cytotoxic effect of glutamate in HT-22 cells. In all cases, in the experiments with varying degrees of glutamate cytotoxicity, the protective effect of **[MoO_2_(HL^3^)]** was registered, and in these groups, less significant changes in cell confluence and shape were visible.

### 2.4. The Effects of [MoO_2_(HL^3^)] in SH-SY5Y Cells

**[MoO_2_(HL^3^)]** was further studied using SH-SY5Y cell line, namely the level of cytotoxicity and the cytoprotective activity on the models of H_2_O_2_-induced cellular injury and glutamate-induced cytotoxicity.

**Figure 10:**
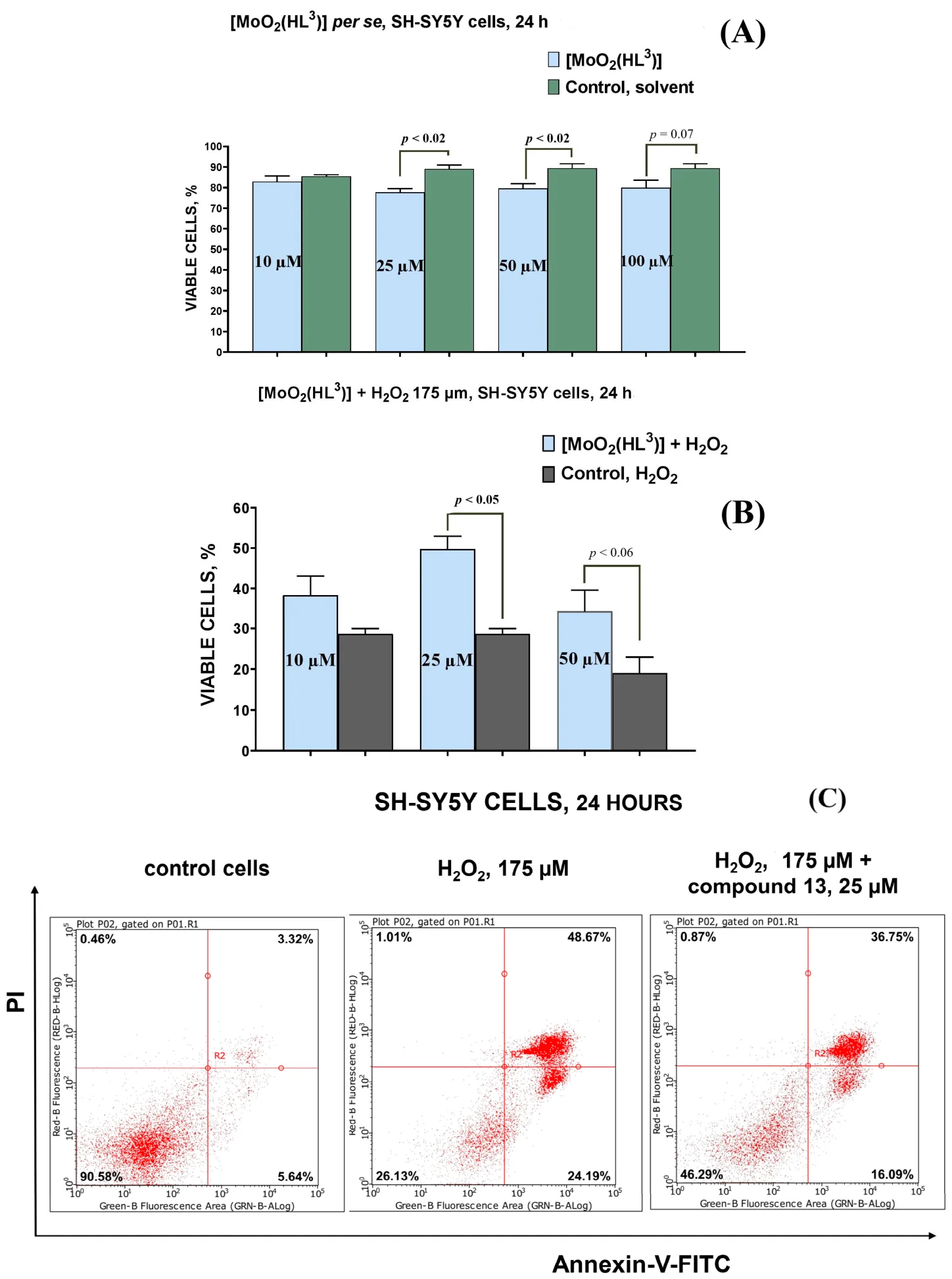

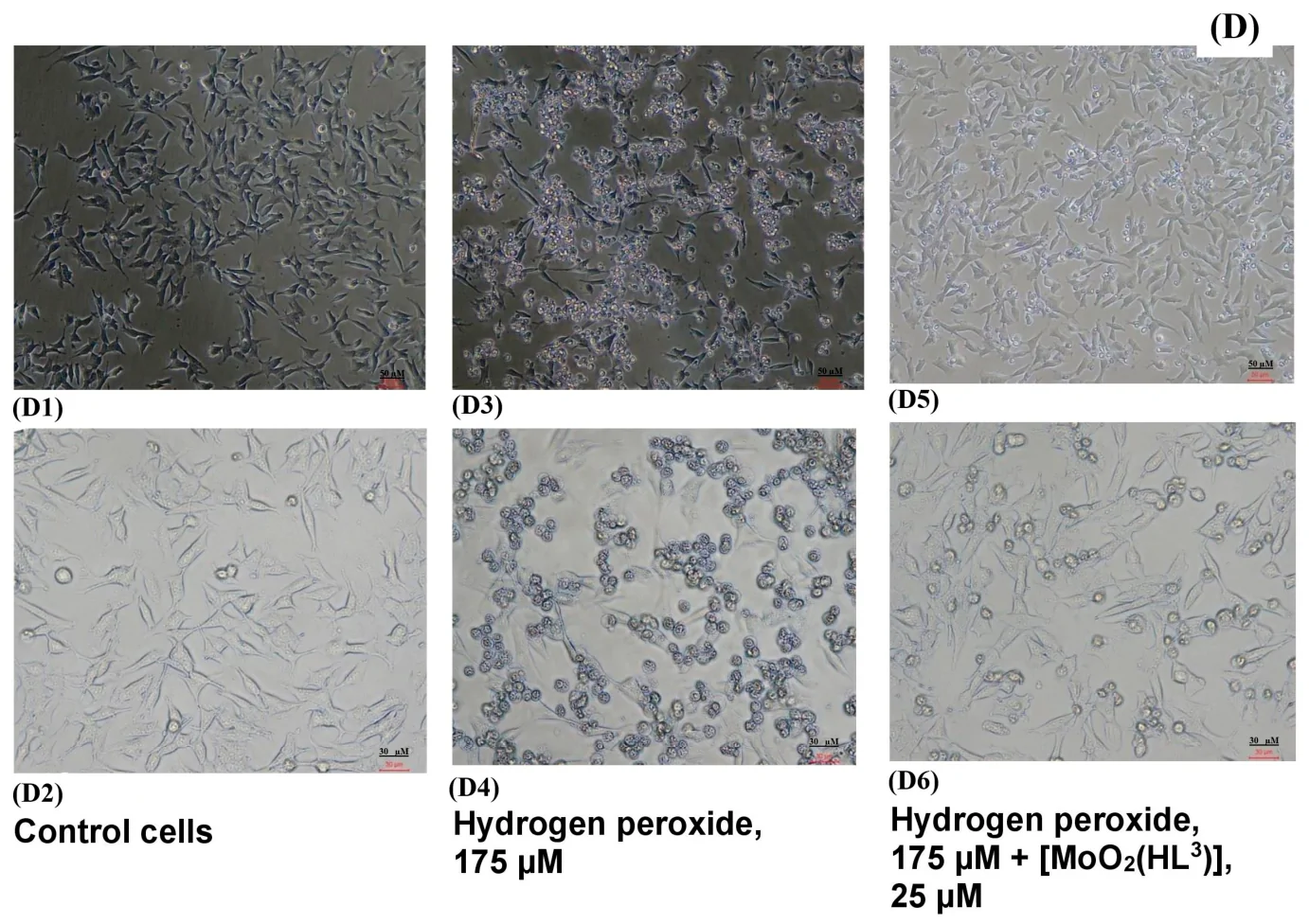
The effects of **[MoO_2_(HL^3^)]** on SH-SY5Y cells viability per se (**A**) and under the conditions of H_2_O_2_-induced cytotoxicity (175 µM, (**B**,**C**)). (**A**,**B**). The Y-axis shows the cell survival (percentage of the viable cells not undergoing necrosis and apoptosis). Data represent the mean ± SEM from at least three (3 to 4) independent biological replicates. Statistical significance of the differences compared with the values of the respective control group is indicated in each graph (*p* < 0.05 or less). (**C**). Representative bivariate histograms of control cells, cells injured with hydrogen peroxide, and cells injured with hydrogen peroxide preincubated with **[MoO_2_(HL^3^)]** (25 µM) after 24 h of incubation. The upper left (UL) quadrant (PI+/Annexin V−) represents necrotic cells, the left lower (LL) quadrant (PI−/Annexin V−) represents viable unchanged cells, the lower right (LR) quadrant (PI−/Annexin V+) represents early apoptotic cells, and the upper right (UR) quadrant (PI+/Annexin V+) represents late apoptotic cells. (**D**). Representative microphotographs of SH-SY5Y cells under the conditions of hydrogen peroxide-induced cytotoxicity (175 µM) incubated with the **[MoO_2_(HL^3^)]** for 24 h. (**D1**,**D2**). Typical morphological structure of the control cells; the cell shape is unchanged. Sporadic single round-shaped cells are visible. (**D3**). Significant reduction in the quantity of visible attached cells and an increase in the quantity of detached round-shaped cells. (**D4**). Significant quantity of the cells with the different degrees of injury up to cell debris. (**D5**,**D6**). The typical morphological structure of the cells and cell confluence are maintained, presence of single round-shaped cells in moderate quantity and sporadic fragments of cell debris. Note. The data concerning other concentrations of **[MoO_2_(HL^3^)]** are given in the Supplementary File S7.

As shown in Figure 10A, **[MoO_2_(HL^3^)]** from the concentration of 25 µM led to a slight albeit statistically significant decrease in cell viability. Generally, the microphotographs (Supplementary File S6) corroborate the findings regarding the increased sensitivity of SH-SY5Y cells compared with HT-22 cells to the cytotoxic effect of the studied compound as well as a clear concentration-dependent effect.

At the same time, just the concentrations 25 µM and 50 µM demonstrated protective activity under the model of hydrogen peroxide-induced cellular injury (Figure 10B). The realization of the protective activity on two cell lines supports the evidence of the beneficial cytoprotective effect of the studied compound.

**Figure 11:**
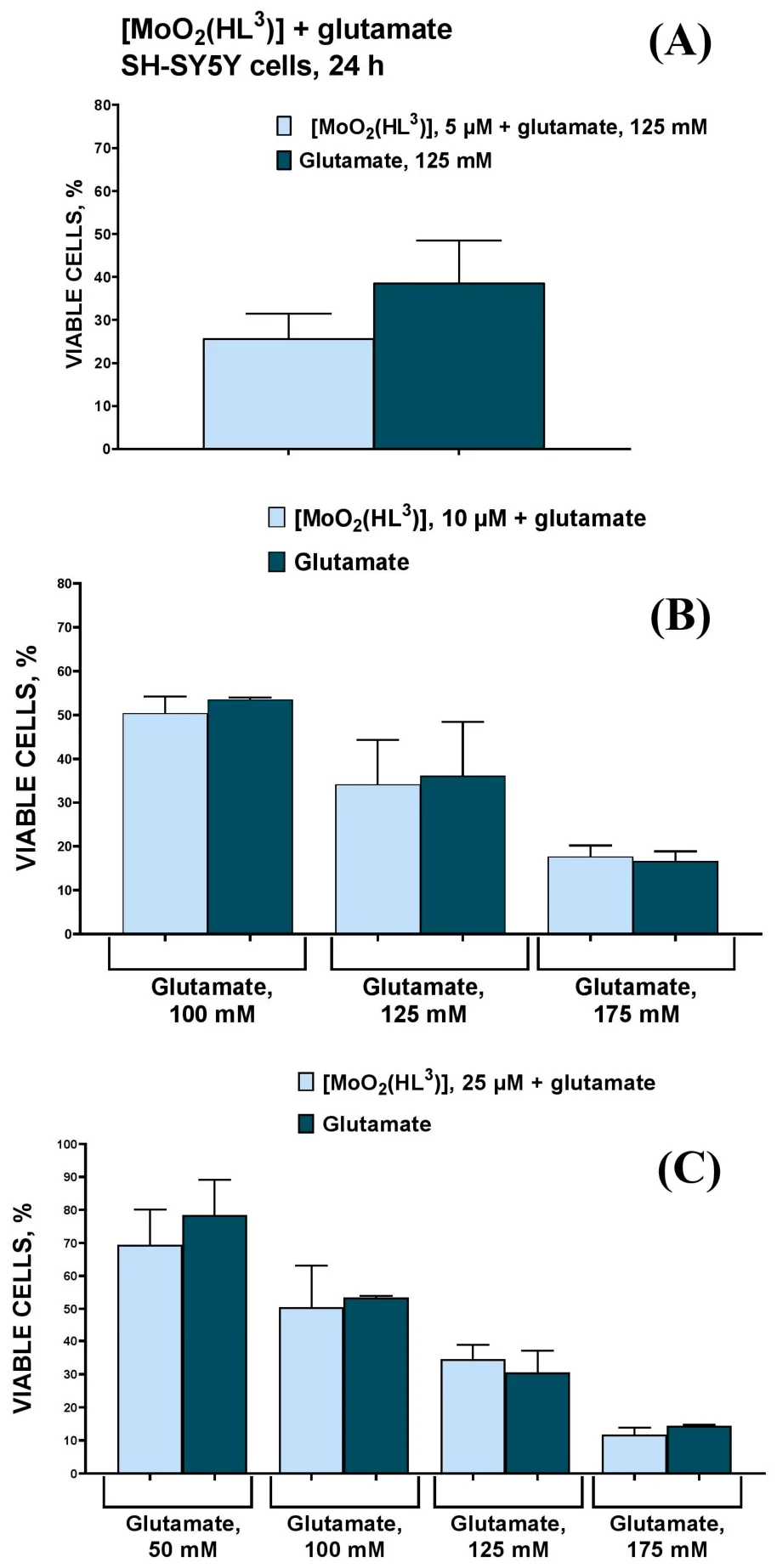

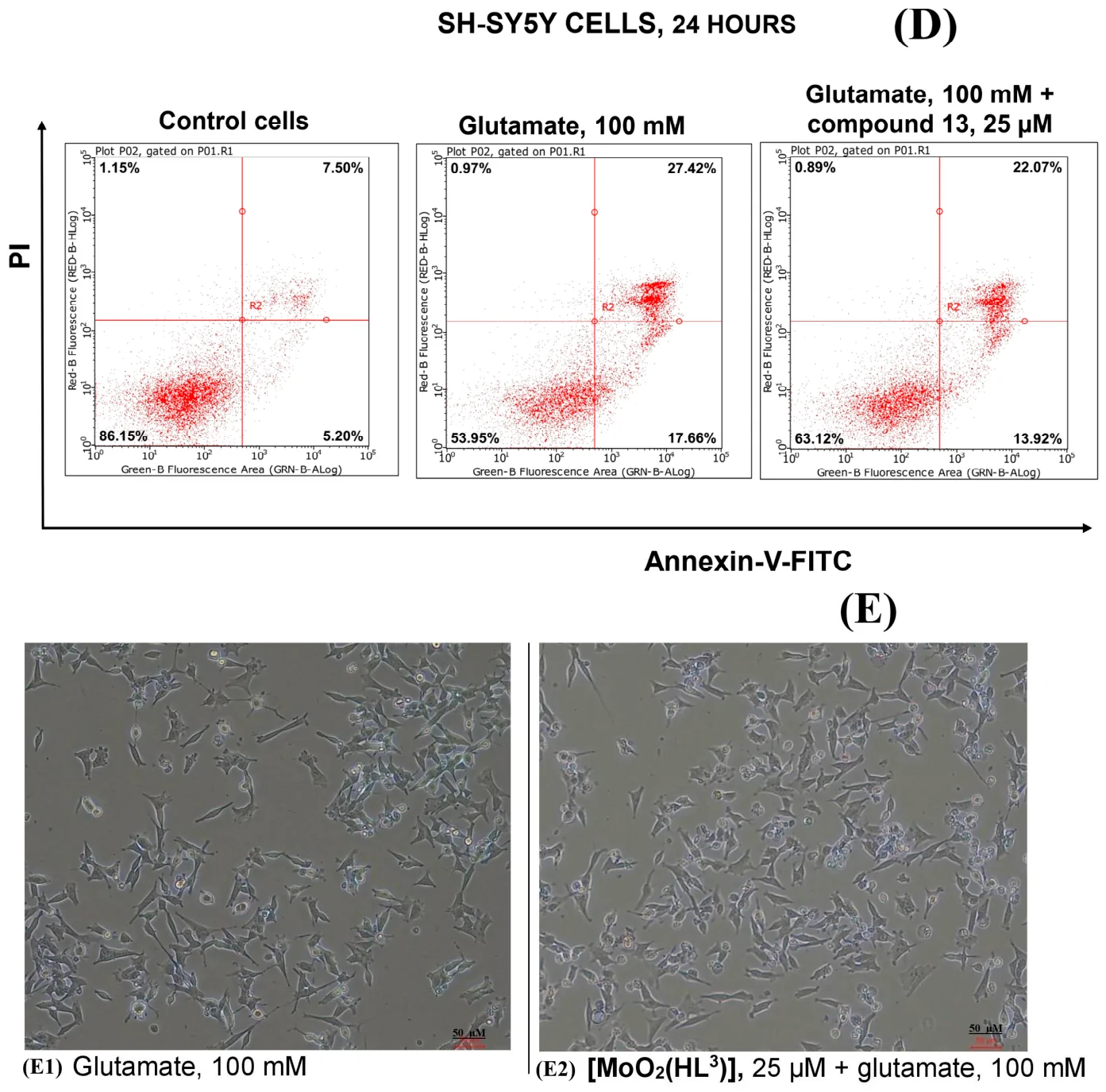
The effects of **[MoO_2_(HL^3^)]** at the different concentrations on SH-SY5Y cells viability under the conditions of glutamate-induced cytotoxicity. (**A**–**C**). The Y-axis shows the cell survival (percentage of the viable cells not undergoing necrosis and apoptosis). Data represent the mean ± SEM from at least three (3 to 4) independent biological replicates. (**D**). Representative bivariate histograms of control SH-SY5Y cells, cells injured with glutamate (100 mM), and cells injured with glutamate (100 mM) preincubated with **[MoO_2_(HL^3^)]** (25 µM) after 24 h of incubation. The upper left (UL) quadrant (PI+/Annexin V−) represents necrotic cells, the left lower (LL) quadrant (PI−/Annexin V−) represents viable unchanged cells, the lower right (LR) quadrant (PI−/Annexin V+) represents early apoptotic cells, and the upper right (UR) quadrant (PI+/Annexin V+) represents late apoptotic cells. (**E**). Representative microphotographs of control SH-SY5Y cells, cells injured with glutamate (100 mM), and cells injured with glutamate (100 mM) preincubated with **[MoO_2_(HL^3^)]** (25 µM) after 24 h of incubation. (**E1**,**E2**). The typical morphological structure of the cells is maintained, the cell confluence is decreased, presence of single round-shaped cells in moderate quantity and sporadic fragments of cell debris. Absence of significant visible differences between the cells incubated with **[MoO_2_(HL^3^)]** or control cells under the conditions of glutamate-induced cellular injury.

The microphotographs (Figure 10, Supplementary File S7) indicate that the most significant protective effect of **[MoO_2_(HL^3^)]** against the cellular injury induced by hydrogen peroxide in SH-SY5Y cells is seen at a concentration of 25 µM. This is consistent with the results of cell viability analysis using Annexin V and propidium iodine staining (Figure 11C).

Since at a concentration of 50 µM the protective effect did not reach the level of significance *p* < 0.05 and a decrease in viability level was seen at concentrations of 50 µM and 100 µM, the latter concentration was not studied in the assay with hydrogen peroxide.

Glutamate causes different level of cytotoxicity in HT-22 and SH-SY5Y cell lines, and our results are in correspondence with the data [44,45] indicating much higher cytotoxic concentrations in the latter cell line.

Nevertheless, on the model of glutamate-induced cytotoxicity, **[MoO_2_(HL^3^)]** did not demonstrate any protective properties (Figure 11). Glutamate was used at several concentrations causing different level of cellular injury (in order to evaluate the activity of the studied compound under the different degree of cytotoxicity); still, there were no trends to the increase in cell viability. **[MoO_2_(HL^3^)]** at lower concentrations of 5 µM and 10 µM (the latter did not cause any cytotoxicity signs) also did not improve cell survival rate studied at several concentrations of glutamate (Figure 11).

Oxidative glutamate toxicity but not excitotoxicity is mentioned as the main pathological process for both HT-22 and SH-SY5Y cell lines [46]. Both of these cell lines were found to be suitable models of glutamate toxicity for the adult brain by the criterion of miRNA profile (substantial overlap of miRNA profiles with primary mouse neurons was registered), and just HT-22 cell line showed the highest correlation to the mouse primary neuronal cultures [47].

However, the absence of the effect in SH-SY5Y can be partially attributed to the lower involvement of the oxidative stress into the cytotoxicity of glutamate. Thus, in the study [45], the lowest percentage of ROS-positive cells was seen just in SH-SY5Y cells among four tested cell types, despite being treated with a much higher dose of glutamate. Also, involvement of caspases differs between these cell lines as follows: HT-22 cells lack caspase activation, their death proceeds via a caspase-independent pathway involving calpain and the translocation of Apoptosis-Inducing Factor (AIF) from the mitochondria to the nucleus. SH-SY5Y cells, however, utilize caspase-dependent pathways [46]. Nevertheless, these mechanisms were not directly addressed; thus, further investigation is required to confirm these differences.

Further studies were directed to the evaluation of the mechanisms of **[MoO_2_(HL^3^)]** cytoprotective activity.

### 2.5. Cyclic Voltammetry Studies on Direct Interaction with H_2_O_2_

**Figure 12:**
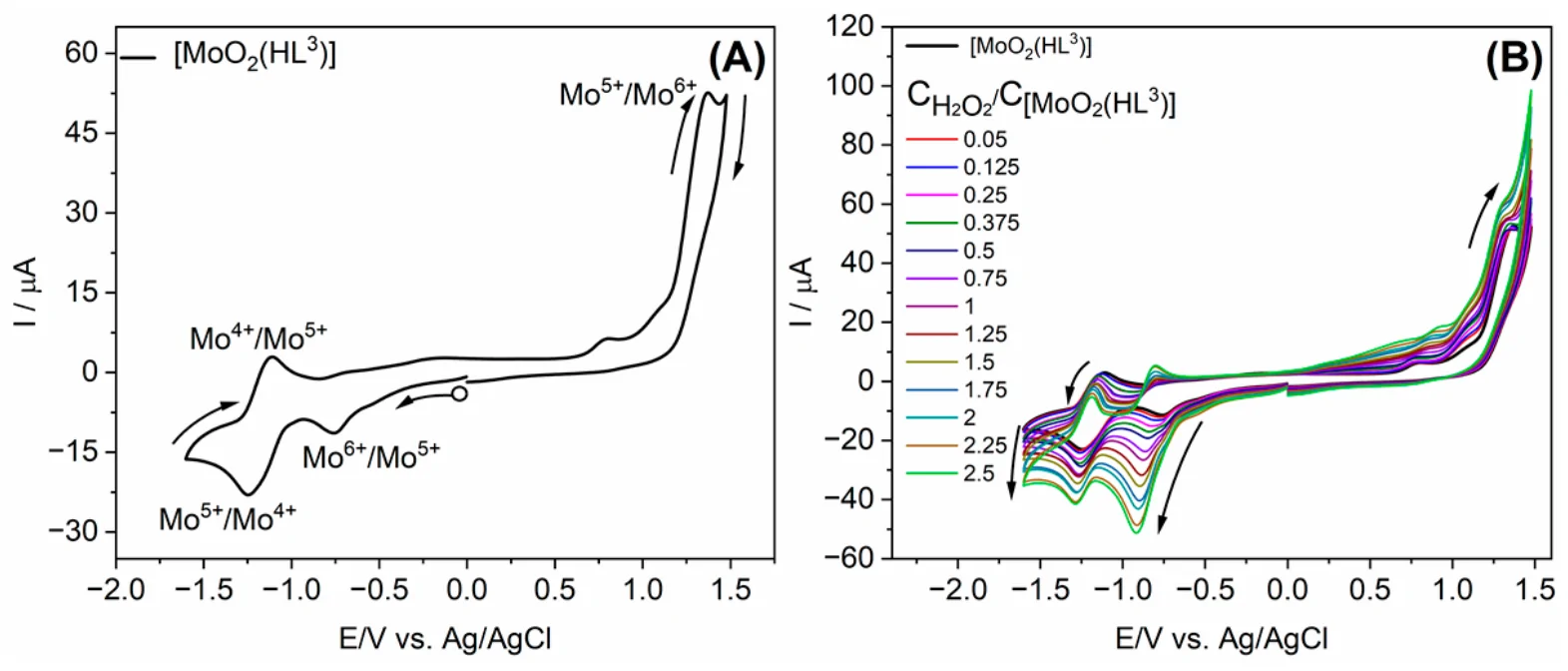
Cyclic voltammograms recorded for: (**A**) the **[MoO_2_(HL^3^)]** (2 mM) in DMSO containing 0.1 M tetrabutylammonium tetrafluoroborate (TBABF_4_), used as the supporting electrolyte. (**B**) **[MoO_2_(HL^3^)]** in DMSO before and after the addition of H_2_O_2_ dissolved in DMSO, for H_2_O_2_ to complex concentration ratios ranging from 0.05 to 2.5; scan rate: 100 mV/s.

Electrochemical studies of the **[MoO_2_(HL^3^)]** were carried out in a DMSO solution containing a supporting electrolyte TBABF_4_. The cyclic voltammetry results presented in Figure 12A indicate that the molybdenum complex undergoes reduction, as evidenced by characteristic cathodic peaks at −0.74 V and −1.24 V, corresponding to the Mo^6+^/Mo^5+^ and Mo^5+^/Mo^4+^ redox couples, respectively. Upon oxidation, two characteristic anodic peaks are observed. The first peak, at −1.10 V, corresponds to the Mo^4+^/Mo^5+^ oxidation process. Subsequently, a pronounced anodic peak with a significantly higher current intensity appears in the positive potential range at +1.36 V can be attributed to the Mo^5+^/Mo^6+^ oxidation. Additionally, a peak observed at approximately +0.76 V may be associated with a non-catalytic process occurring after the reduction in the complex.

To verify the ability of the investigated compound to interact with hydrogen peroxide, cyclic voltammetry (CV) measurements were performed. The experiments were conducted over a concentration range different from that used in the biological studies, as no measurable electrochemical signals were observed at the H_2_O_2_ concentrations applied in the biological assays. Therefore, CV measurements were carried out at various H_2_O_2_ to **[MoO_2_(HL^3^)]** concentration ratios, ranging from 0.05 to 2.5. The results of the electrochemical measurements are presented in Figure 12B.

**Figure 13:**
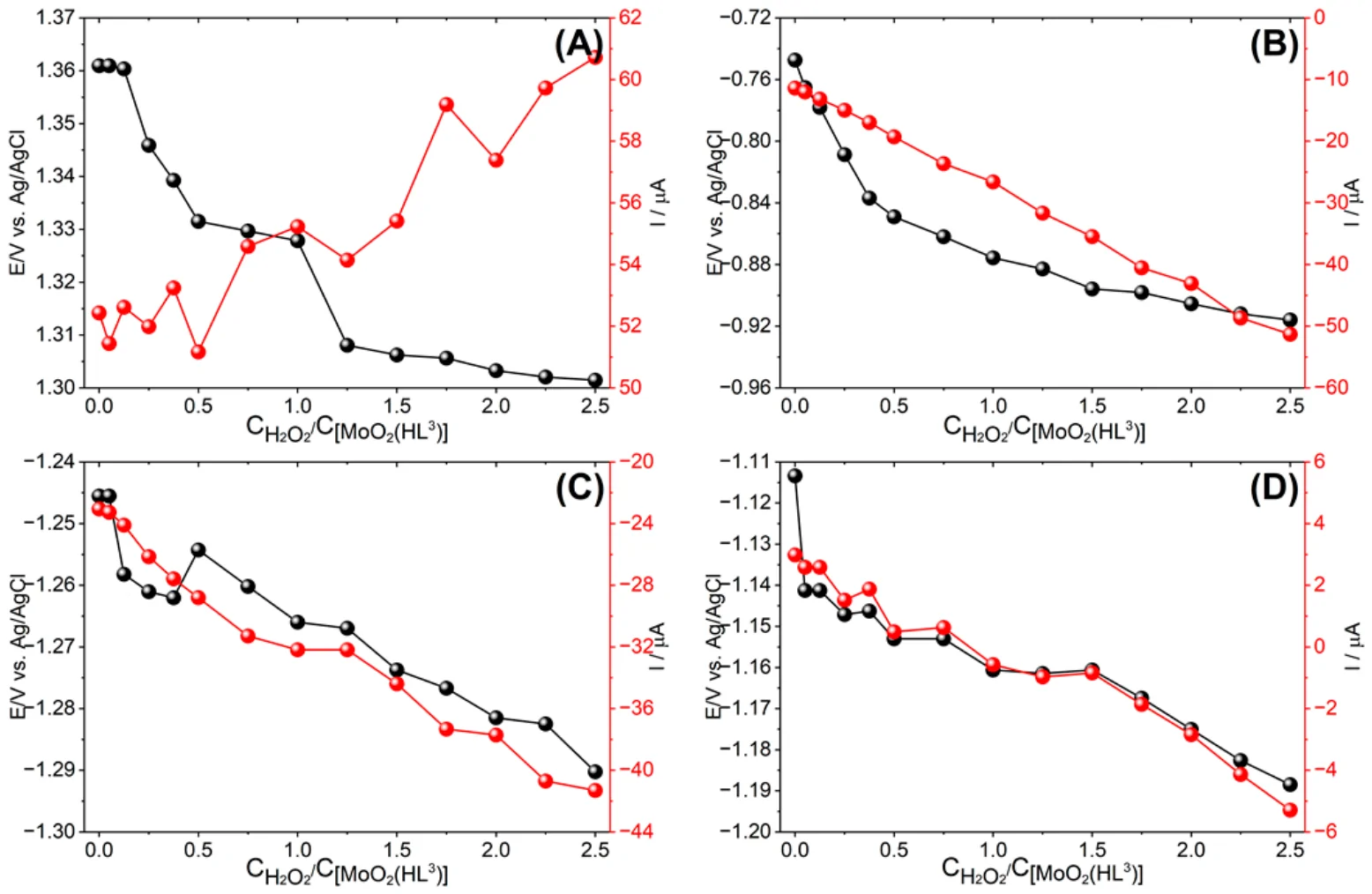
Correlation between peak current intensities and the corresponding peak potentials for the voltammograms shown in Figure 12B after H_2_O_2_ addition, with H_2_O_2_ to complex concentration ratios. Data are presented for the peaks observed at +1.36 V, −0.76 V, −1.24 V, and −1.11 V (**A**–**D**), respectively.

The obtained results directly demonstrate that the addition of H_2_O_2_ to the solution containing **[MoO_2_(HL^3^)]** induces significant changes in the cyclic voltammograms. Importantly, the addition of H_2_O_2_ leads to noticeable changes in both cathodic and anodic peaks, reflected by shifts in their peak potentials as well as variations in their current intensities. Furthermore, upon exceeding a molar ratio of 0.5 (H_2_O_2_ to complex, 0.5:1), an additional peak appears at approximately −0.87 V. This observation suggests the formation of new electroactive species and/or the occurrence of additional redox processes induced by the presence of hydrogen peroxide. For a more detailed analysis of the obtained voltammograms, Figure 13 presents the dependence of peak current intensities and peak potentials on increasing H_2_O_2_ concentration. The analysis of the peaks in the voltammograms clearly indicates that even the smallest amount of hydrogen peroxide, at a molar ratio of 0.05:1 (H_2_O_2_ to complex), induces noticeable changes in both peak positions and peak currents. The analysis includes the oxidation peak at +1.36 V, the reduction peaks at −0.72 V and −1.24 V, as well as the peak at −1.11 V.

At a potential of +1.36 V, the most significant changes in current and peak potential are observed at the lowest tested ratios as well as after exceeding a ratio of 0.125, as shown in Figure 13A. Studies of the peak at −0.76 V (Figure 13B) reveal that even minimal additions of H_2_O_2_ change both the peak height and its potential. Subsequent additions result in a linear decrease in current accompanied by a shift in peak potential. A similar trend is observed for the peak at −1.25 V (Figure 13C), although even the smallest addition of H_2_O_2_ (0.05:1) causes pronounced changes in peak position. In the case of the peak at −1.11 V (Figure 13D), only minor changes in current, about 10 µA and potential about 0.1 V, are observed. Nevertheless, the progressive changes with each successive H_2_O_2_ addition are clearly evident.

In summary, these results demonstrate a linear and concentration-dependent response, confirming that **[MoO_2_(HL^3^)]** actively interacts with hydrogen peroxide across the entire range of tested molar ratios. The electrochemical studies indicate that **[MoO_2_(HL^3^)]** interacts with hydrogen peroxide.

Such interaction can be expected to be realized in the biological media and thus represent one of the mechanisms of the **[MoO_2_(HL^3^)]** protective activity. Therefore, these results are consistent with the data obtained on the model of hydrogen-peroxide-induced injury.

### 2.6. DCF-DA Assay and Influence on Catalase and SOD Activity

**Figure 14:**
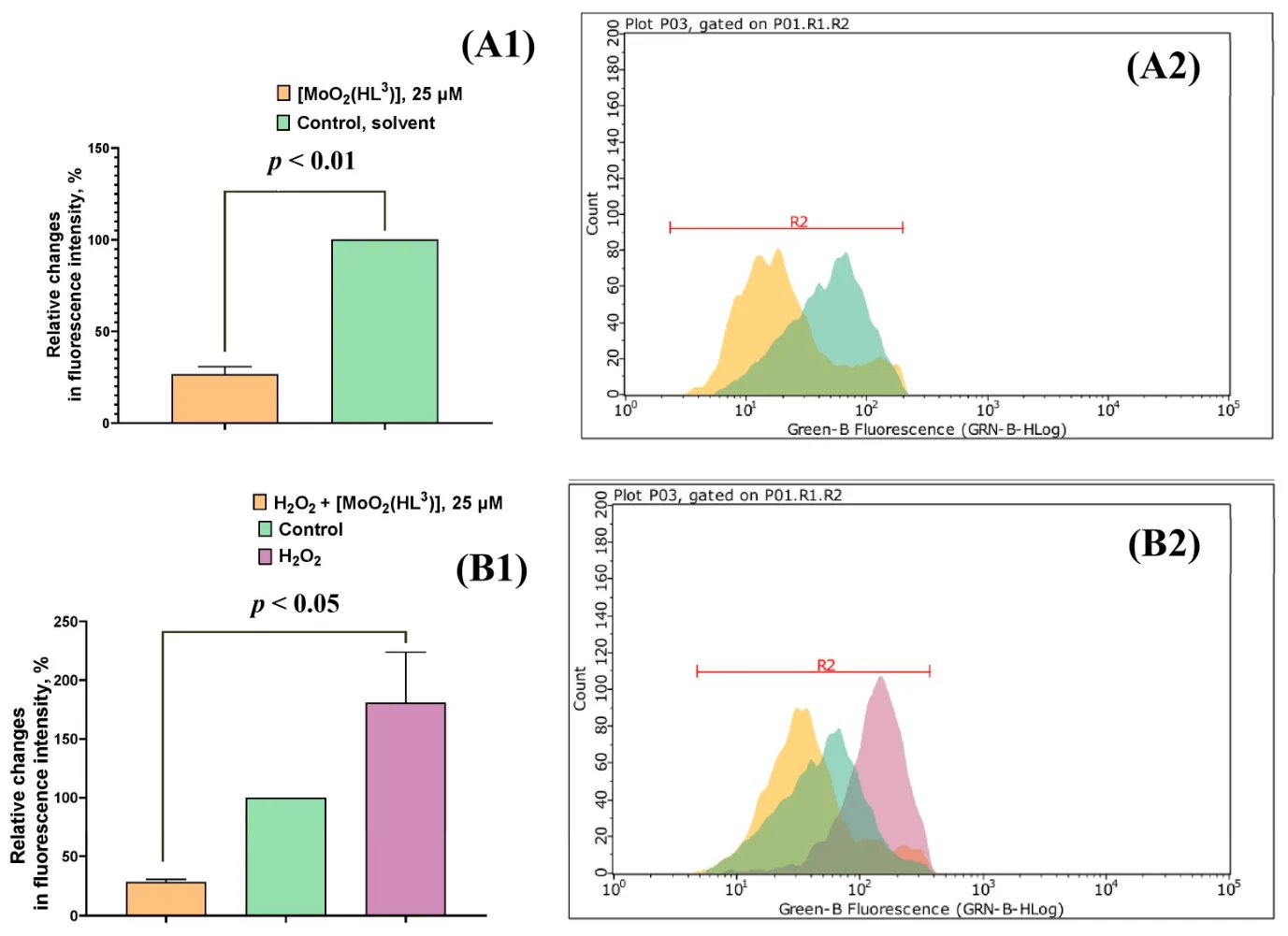
The effects of **[MoO_2_(HL^3^)]** at a concentration of 25 µM on the fluorescence intensity in HT-22 cells (compound per se (**A1**,**A2**) and against the background of hydrogen peroxide (**B1**,**B2**) 2′,7′-dichlorodihydrofluorescein diacetate (DCF-DA) assay, flow cytometry (1-h preincubation with the compound 13, adding DCF-DA simultaneously with hydrogen peroxide or without it, measurement 30 min after). (**A1**,**B1**). The Y-axis shows the relative changes in fluorescence intensity (the value of the control cells was set as 100%). Data represent the mean ± SEM from three independent biological replicates. Statistical significance of the differences compared with the values of the respective control group is indicated on each graph (*p* < 0.05 or less). (**A2**,**B2**). Representative bivariate histograms visualizing shifts in fluorescence peaks between experimental groups.

Among the possible mechanisms of the cytoprotective activity of **[MoO_2_(HL^3^)]** antioxidative effect was of a special interest. DCF-DA assay results have shown that the studied compound per se decreased DCF fluorescence intensity in HT-22 cells (Figure 14A), suggesting direct free radical scavenging activity. It is generally known that fluorescent DCF is formed from non-fluorescent DCFH through oxidation by intracellular ROS, and DCFH is also formed intracellularly with the participation of esterase [48].

Thus, these assay results indirectly support the fact of **[MoO_2_(HL^3^)]** transport through the cellular membrane inside the cell.

**Figure 15:**
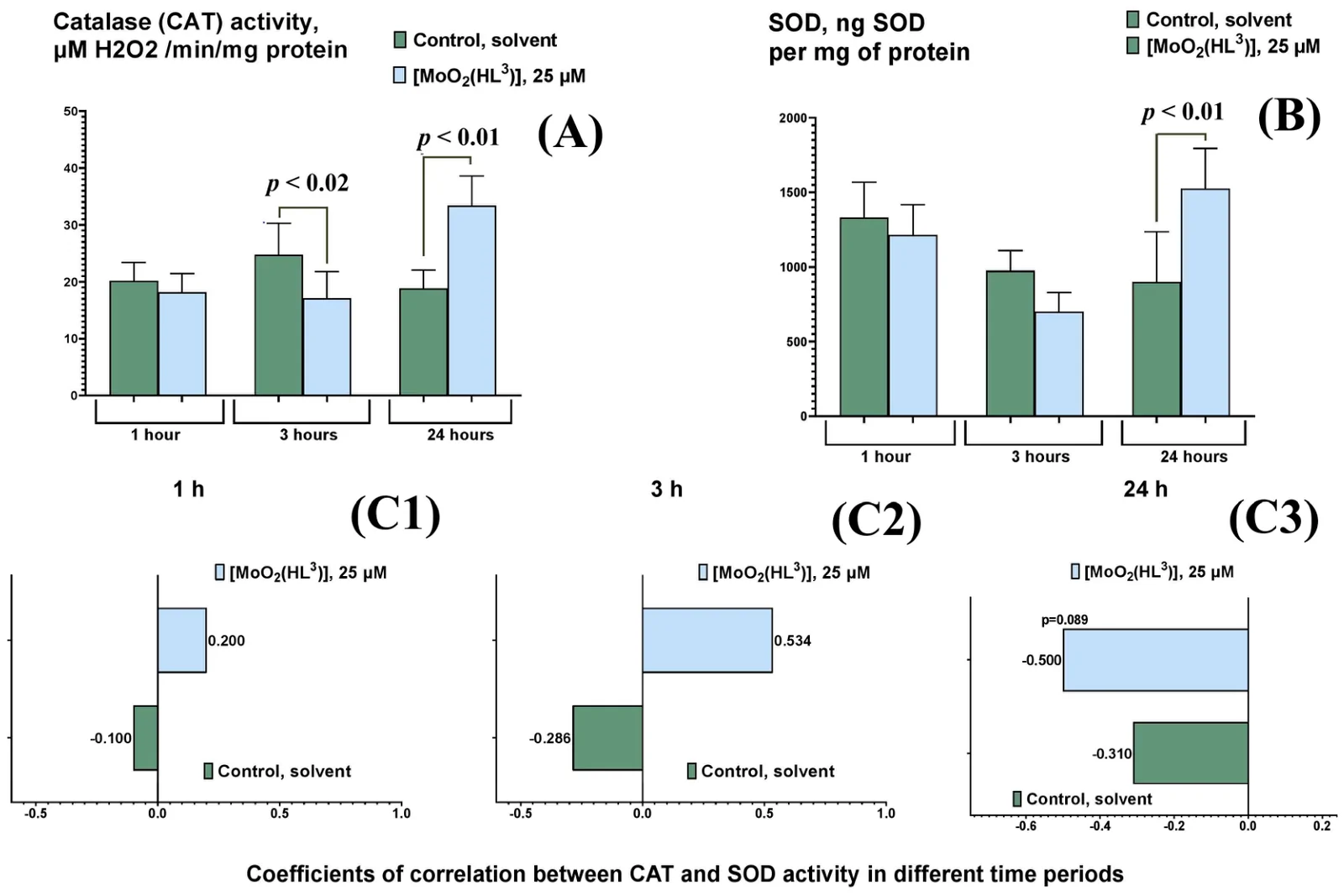
The influence of **[MoO_2_(HL^3^)]** per se at a concentration of 25 µM on catalase and SOD activity in HT-22 cells in different time periods. (**A**). Catalase (CAT) activity, μM H_2_O_2_/min/mg protein. (**B**). SOD, ng SOD per mg of protein. Data represent the mean ± SEM from at least three (3 to 7) independent biological replicates. Statistical significance of the differences compared with the values of the respective control group is indicated on each graph (*p* < 0.02 or less). (**C1**–**C3**). Coefficients of correlation between CAT and SOD activity in different time periods.

It is consistent with the finding that catalase activity after 3 h of incubation with **[MoO_2_(HL^3^)]** per se was statistically significantly decreased in HT-22 cells (after 1 h of incubation the changes were not statistically significant; Figure 15). Functioning of catalase is regulated at various levels [49], and previous reports indicate that direct antioxidants, which decrease superoxide and subsequently hydrogen peroxide level, can downregulate catalase expression and activity [50]. Under the short duration of incubation used in the described experiment, only downregulation of activity is more possible; still, these aspects need further investigation. SOD activity in this time period (1 and 3 h) only tended towards decrease.

After 24 h, an opposing trend was observed; namely, catalase and SOD activity were higher in cells pretreated with **[MoO_2_(HL^3^)]** than in the control cells (Figure 15). As mentioned above, the cells were under serum deprivation and decreased glucose supply; thus, this effect could be a positive one. From the other point of view, the absence of prolonged decrease in catalase activity is also favourable. Directionality of correlation between SOD and catalase activity was not changed by **[MoO_2_(HL^3^)]**, compared with the value of the control cells. It should be noted that in the control cells, in all studied time periods, no statistically significant correlations between SOD and catalase activity were seen. It could be explained by the fact that catalase represents not the only way to inactivate hydrogen peroxide within the cell, and it is known that under low levels of oxidative stress (which could be expected in these control cells), glutathione peroxidases generally realize this inactivation [51].

**Figure 16:**
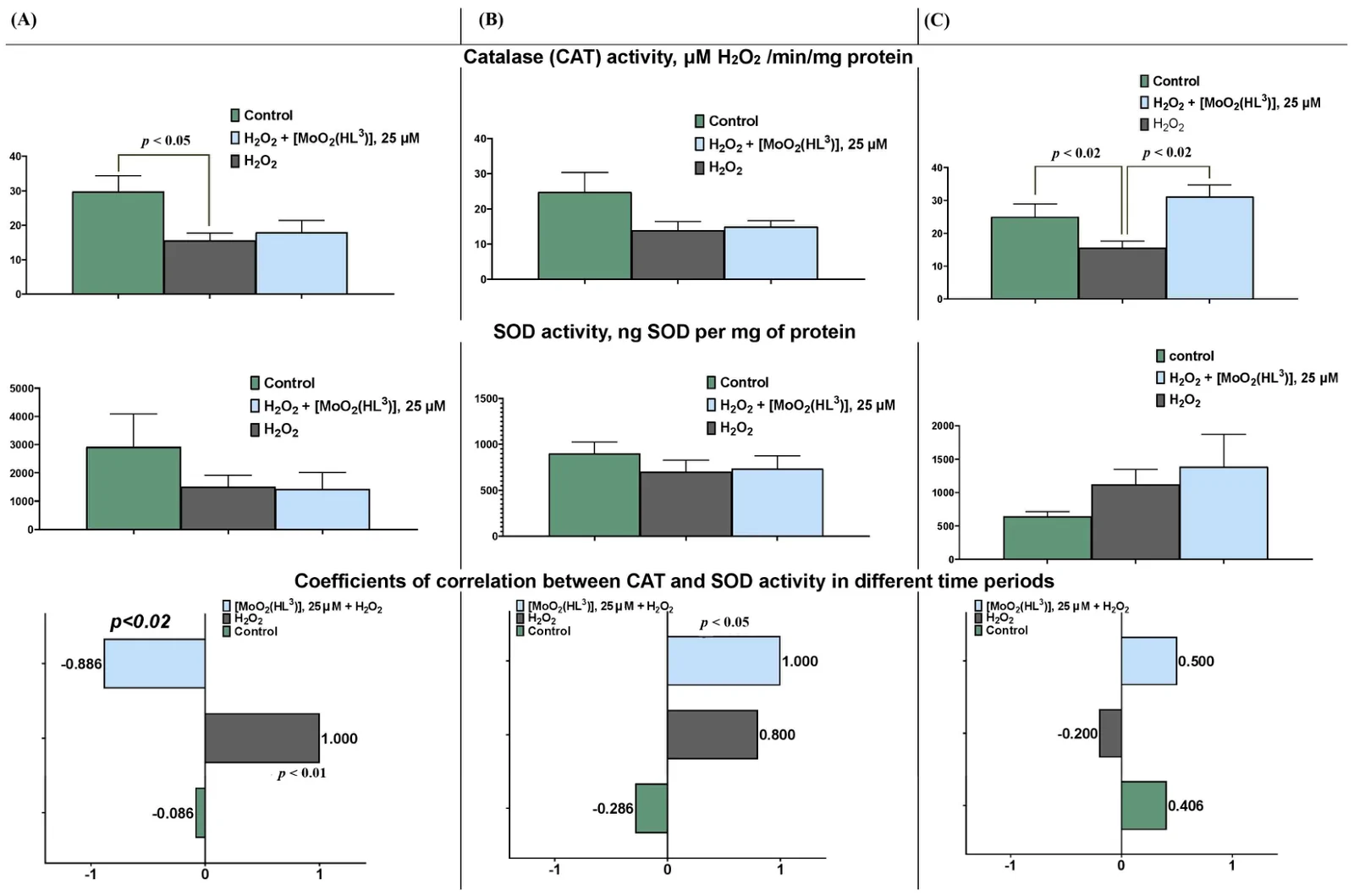
The influence of **[MoO_2_(HL^3^)]** at a concentration of 25 µM on catalase and SOD activity in HT-22 cells under the conditions of H_2_O_2_-induced cytotoxicity (500 µM) at the different time periods. (**A**) 30 min after adding hydrogen peroxide; (**B**) 2 h after adding hydrogen peroxide; (**C**) 24 h after adding hydrogen peroxide. Data represent the mean ± SEM from at least three (3 to 7) independent biological replicates. Statistical significance of the differences compared with the values of the respective control group is indicated on each graph (*p* < 0.05 or less).

At the same time, when intensification of the prooxidative processes was induced by hydrogen peroxide, the studied compound effectively reduced DCF fluorescence intensity. Since this concentration of **[MoO_2_(HL^3^)]** also significantly increased cell viability under the conditions of hydrogen peroxide-induced cytotoxicity, limiting prooxidative processes through the possible scavenging of free radicals could be supposed to be involved into the mechanisms of cytoprotective activity. At early time periods the studied compound did not change the decreased catalase activity, namely, 30 min and 2 h after adding H_2_O_2_ (Figure 16; SOD activity also tended towards a decrease in these time periods). However, the decreased activity of the enzymes was evident against a background of a significant burst of ROS after H_2_O_2_ influence on control cells and against a background of a decreased ROS levels in cells preincubated with **[MoO_2_(HL^3^)]** (Figure 14).

**Figure 17:**
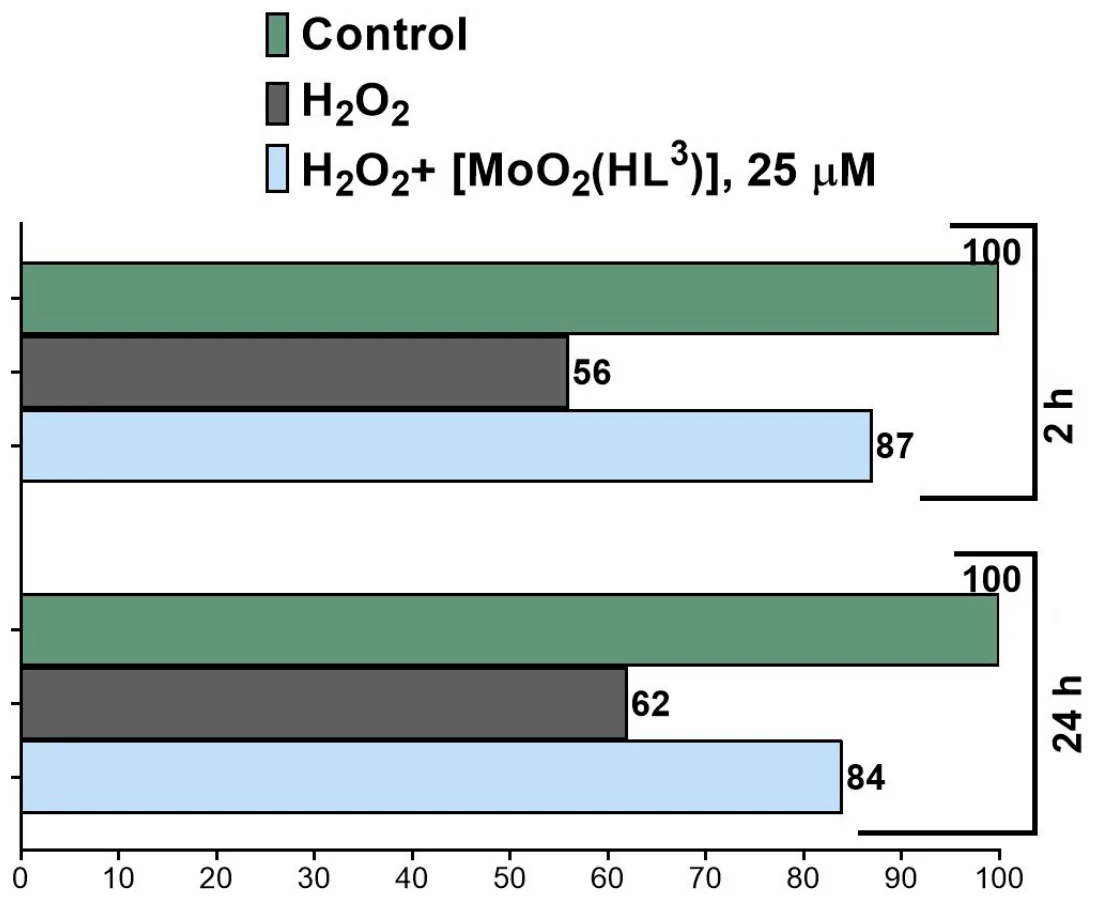
The relative changes in catalase activity (control value is set as 100%) in HT-22 cells injured with H_2_O_2_ and pretreated with **[MoO_2_(HL^3^)]** at a concentration of 25 µM.

When the relative decrease in catalase activity was calculated (namely, in each time period we compared the values in two pairs of datasets, such as control cells compared with cells injured with hydrogen peroxide and the cells pretreated with **[MoO_2_(HL^3^)]**, and compared with these cells similarly injured with hydrogen peroxide), the conclusion can be made that the relative decrease in catalase activity induced by hydrogen peroxide was significantly alleviated by **[MoO_2_(HL^3^)]**, both after 2 h and after 24 h (Figure 17).

These events can determine the prognosis for cell survival which, as described above, was considerably improved in cells pretreated with **[MoO_2_(HL^3^)]**. In these cells, catalase activity after 24 h was completely restored (Figure 17). Available data do not permit a definitive conclusion as to whether this effect results from direct interaction of the compound with the enzyme or as a consequence of significantly decreased cellular injury. In any case, this is in full accordance with the data on improvement of cell survival and cell morphology described above.

SOD activity, in contrast to catalase activity, was not decreased on the control cells which underwent H_2_O_2_-induced injury. This can represent an unfavourable change, since excessive quantities of H_2_O_2_ can arise if SOD activity is not accompanied with the corresponding catalase activity (either leading to cytotoxicity or overloading of other antioxidant systems, such as glutathione peroxidases, which generally function under low levels of oxidative stress [51]). The change in the directionality of correlation between these two enzymes also indicates the discoordination in the system of the antioxidant defence (nevertheless, the correlation coefficients did not reach the level of statistical significance; Figure 16). In the cells incubated with **[MoO_2_(HL^3^)]**, the increased SOD activity was seen against the background of significantly enhanced catalase activity, and the correlation between enzymatic activities closely resembled that of the control cells which did not undergo cellular injury.

Additional study was done in the conditions of lower injury in the cells (lower concentration of H_2_O_2_, namely 250 µM), and in this case no shifts in the enzymes activity were caused by **[MoO_2_(HL^3^)]**. Thus, these data align well with the results, proving the increase in cell viability and improvement in their morphology described above.

## 3. Summary of Results and Future Research Prospects

The present study was based on the previous findings, which have shown that molybdenum(VI) Schiff base complexes (in contrast to oxidovanadium(V) complexes with the similar structures) showed no cytotoxicity and protective activity against cell injury induced by hydrogen peroxide by the results of MTT test after 24 h [25,26].

These data are consistent with the current studies, which employed the longer periods of observation and advanced analysis of cell viability. Based on these results, the selection of the **[MoO_2_(HL^3^)]** for further studies was substantiated by the following rationale: the presence of statistically significant protective effect in the concentration range from 10 to 50 µM, with significant cytotoxicity, was observed only at a concentration of 100 µM. In contrast, significant activity of **[MoO_2_(HL^6^)]** was observed at a concentration 200 µM only; **[MoO_2_(HL^5^)]** demonstrated cytotoxicity at a concentration of 50 µM.

Further investigation focused on establishing the limits of the protective effect of **[MoO_2_(HL^3^)]** under similar experimental conditions, which revealed statistically significant protective effects at concentrations up to 5 µM, with an increased cell viability (though not reaching the level of statistical significance) observed at concentrations up to 1 µM (Figure 3). A relatively wide range of effective concentrations can be considered a clear advantage of the studied compound.

Concurrently, at concentrations of 25 µM and 50 µM, significant cytoprotective activity was observed in HT-22 and, at the lower concentration, in SH-SY5Y cells. However, these same concentrations caused a shift towards a decrease in cell viability in HT-22 cells and a slight, albeit statistically significant, reduction in this value in SH-SY5Y cells.

A partial explanation for these patterns may be found in the concept of hormesis, as a dose–response phenomenon characterized by low-dose stimulation and high-dose inhibition. It is hypothesized [52,53,54] that the mechanism underlying the efficacy of low doses of such molecules consists of the activation of pro-survival pathways, such as heat shock response (HSR) pathways, and other mechanisms under the control of protective genes, including those counteracting apoptosis. On both models of cellular injury used in our study, the decrease in the percentage of the cells undergoing apoptosis was registered (with the corresponding increment of the viable cells percentage). While apoptosis induction is a generally recognized mechanism of metal-based compound cytotoxicity, the neuroprotective activity of molybdenum-containing compounds can be realized through apoptosis counteraction [5,55]. Such properties have recently been confirmed not only for molybdenum-containing nanoparticles [56] but also for sodium molybdate. For the latter compound, anti-apoptotic activity was demonstrated in an in vivo model of neurodegeneration caused by beta-amyloid (verified by the decrease in the Bax/Bcl-2 ratio) [57].

As described in Section 2.4, the involvement of cell-death pathways is illustrated by the difference between the efficacy of **[MoO_2_(HL^3^)]** on glutamate-induced cytotoxicity model in HT-22 cells (caspase-independent model) and lack of its efficacy in SH-SY5Y cells (caspase-dependent model [46]). Further research is needed to establish certain differences.

At the same time, the current study was focused on the influence on the oxidative stress as a target of neuroprotection. In this context, simultaneous influence on multiple oxidative pathways is advantageous. This approach is more rational than exerting a direct antioxidant effect, particularly given that direct antioxidants can act as pro-oxidants, thus exacerbating cellular damage [58,59,60]. Additionally, it should be noted that the studied compound demonstrated the following several mechanisms of action related to oxidative stress: (1) interaction with hydrogen peroxide (proven by cyclic voltammetry measurements; see Section 2.5); (2) inactivation of free radicals in the intracellular space (supported by results of the DCF-DA assay); and (3) maintenance of catalase and SOD activity in injured cells (see Section 2.6).

These results are consistent with data in the published literature.

Firstly, regarding free radical inactivation, chelation compounds are considered to be a substitute for “traditional” synthetic antioxidants. Their advantages include improved activity arising from the characteristics of both metals and ligands [61]. Notably, molybdenum was able to decrease reactive oxygen species in HK-2 kidney cells challenged with hydrogen peroxide-induced injury [62]. It did not show significant cytotoxicity (despite the generally recognized concept of nephrotoxicity of metals) and upregulated MnSOD expression in cells undergoing oxidative injury caused by hydrogen peroxide. This finding is partially consistent with our results concerning the maintenance of SOD activity under the influence of the studied compound, which also counteracted hydrogen peroxide-induced pro-oxidative injury. Moreover, such phenomena were observed not only in vitro, but also clinically. Thus, in the cited population-based cohort study [62], it was shown that higher urinary molybdenum levels were associated with lower levels of systemic oxidative stress. From the point of view of safety, it is important to mention that the reduction in Mo^VI^ to Mo^V^ or Mo^IV^ does not happen in the cellular environment. Thus, the cellular injury through the increased production of ROS is less possible [63]. It has also been shown that nanomaterials based on molybdenum disulfide (MoS_2_) differ in their systemic toxicity, and this toxicity is mitigated in cases of potent molybdenum complexation [63,64,65], which once again emphasizes complexation role in the development of safe metal-based biologically active compounds.

Secondly, among interventions counteracting oxidative mechanisms, novel strategies involving the use of SOD mimetics have been developed. These mimetics, in accordance with the concept described in [58] involve not only the removal of superoxide but also the scavenging of hydrogen peroxide, thus preventing the formation of hydroxyl radicals. This mechanism is consistent with our results on the interaction of the studied compound with hydrogen peroxide and maintenance of antioxidant enzyme activity.

It is noted that such SOD mimetics, despite lower rate constants compared to native enzymes, have the advantage of remaining active in extracellular spaces where endogenous enzymes are sparse [58]. Synthetic catalytic SOD/catalase mimetics are of great interest as potential neuroprotective agents [66]. The fact that metal-based compounds (albeit other than molybdenum) considered SOD/CAT mimetics not only protect cells against oxidative stress but also prolong life span has been demonstrated in the study [12] in the context of neurodegeneration as an ageing-related processes. A series of salen-Mn complexes have been developed as synthetic SOD and catalase mimetics and have been shown to be beneficial in numerous oxidative stress models. They have demonstrated the ability to attenuate mitochondrial injury, as demonstrated in models induced by ionizing radiation, absence of mitochondrial SOD (Sod2−/− mice), and ischemia–reperfusion in ABC-me−/+ mice vulnerable to oxidative stress [67]. Notably, **[MoO_2_(HL^3^)]** protective activity against hydrogen peroxide-induced injury was also manifested in the normalization of the mitochondria structure, with the maintenance of visible crista and sufficiently dense matrix (see Figure 9).

At the same time, our results represent preliminary screening data identifying a promising compound among the synthetized complexes based on the verification of protective activity and cytotoxicity level. Obviously, they have certain limitations. Within the influence on pro-oxidant-antioxidant balance, the causal sequence regarding enzymatic activity (mainly maintained activity of catalase) was not established definitively. The evidence from the studies with adding of **[MoO_2_(HL^3^)]** after the cellular injury with hydrogen peroxide as well as from the studies on glutamate-induced model in HT-22 cells indirectly support the concept that inactivation H_2_ O_2_ (and/or binding to free radicals with their inactivation) is not the only mechanism for the decreased toxic influence on the cells and thus maintained activity of antioxidant enzymes. Nevertheless, the crucial direction for the further experiments is evaluating the possibility whether the compound influences the expression of antioxidant defence genes such as Nrf2 and HO-1 and whether there are changes in the expression of catalase and SOD.

As described in Section 4.9, no changes in catalase activity were seen when HT-22 cell lysates were incubated with **[MoO_2_(HL^3^)]**, neither there were the changes in the enzyme activity registered when HT-22 cells were incubated with this compound for one hour (Figure 16). Thus, direct influence on catalase activity (on its active and allosteric centres) is less possible, though we have not studied this process in details. Other aspect of interest is the fact that catalase activity in neural cells in vitro can be decreased by cytotoxic concentrations of aggregated beta-amyloid peptides, which directly interact with the enzyme, and these interactions are successfully prevented by small molecule inhibitors, which in this way protect the cell from the augmented level of H_2_O_2_ and the consequent oxidative injury [68]. It would be of great interest to investigate whether such mechanism is inherent in the coordination compounds of molybdenum.

Finally, with due regard to the lack of specificity of the used DCF-DA assay in regard to the certain different reactive oxygen species or their intracellular origin, further in vitro and/or in silico studies are reasonable. In the current study we addressed only the influence of **[MoO_2_(HL^3^)]** on pyrogallol autoxidation, and the results evidence the absence of such influence (Section 4.9), allowing to exclude the direct superoxide scavenging under studied conditions.

Other limitation outside the scope of this study is pharmacokinetics. Indeed, the dictum “no on-site protection, no protection” is emphasized in the review [58] in the context of free radical neutralization. Addition to the generally recognized influence of binding to albumins on both the protective activity and toxicity of metal-containing complexes and the possible transformation of organic complexes in aqueous media (such as ligand exchange, hydrolysis, chemical bond breaking, and redox reactions) is emphasized as an overlooked process for these complexes [63].

At the same time, there is evidence about bioavailability of molybdenum-contained compounds. Cell membrane molybdate transporters (MoT2) are expressed in eukaryotes, and their presence in humans has been confirmed. After delivery to the cells, molybdate is incorporated into molybdopterin-cofactor (Moco), which is the biologically active prosthetic group in molybdenum-dependent enzymes [11,55,65,69]. Under the conditions of brain injury, these processes can contribute to the beneficial maintenance of enzymes, such as xanthine oxidase (given that uric acid can exert neuroprotective activity under specific conditions [18,19,20,64]) as well as sulfite oxidase (removing cytotoxic sulfite [21,22,64] and participating in NO production in astrocytes as a protective mechanism in hypoxia [70]).

These data together with the results on cytoprotective activity obtained in the current study substantiate the expediency of studies of quantitative intracellular uptake measurements for **[MoO_2_(HL^3^)]** together with the estimation of the ability of the compound to penetrate through BBB. If they confirm sufficient intracellular uptake and if they are accompanied with further evidence of cytoprotective mechanisms realization (especially such as Nrf2 expression), further advanced models can be implied. Proceeding from the coordination metal compound effects described in the literature and summarized above, the most prospective models are those elucidating beta-amyloid toxicity counteraction (preliminary analysis on traditional cell cultures with further transition to neuron-glia models and 3D tri-culture, including the analysis of catalase activity in the presence of beta-amyloid).

## 4. Materials and Methods

All *cis*-dioxidomolybdenum(VI) complexes with chiral tetradentate Schiff bases chosen for our studies were synthesized and spectroscopically characterized in our previous papers, i.e., **[(+)MoO_2_(HL^1^)(CH_3_OH)]**, **[(+)MoO_2_(HL^5^)]** [71] **[MoO_2_(HL^3^)]**, **[MoO_2_(HL^5^)]**, **[MoO_2_(HL^6^)]** [72] **[MoO_2_(HL^2^)]** and **[MoO_2_(HL^4^)]** [73].

All compounds have excellent solubility in DMSO; thus, the stock solutions were made using DMSO (Sigma-Aldrich, Merck KGaA, Darmstadt, Germany) as described below.

### 4.1. Cell Culture

All cell cultures were maintained at 37 °C in a humidified atmosphere supplemented with 5% CO_2_.

#### 4.1.1. The Culture of HT-22 Cells

The mouse hippocampal neuronal cell line HT-22 was a kind gift from Prof. Tilman Grune (Friedrich Schiller University, Jena, Germany), and the authors are grateful for this.

HT-22 cells were cultured in Dulbecco′s Modified Eagle′s medium (DMEM, PAN-Biotech GmbH, Aidenbach, Germany, catalogue number P04-01550), supplemented with 10% fetal bovine serum (FBS), 100 U/mL penicillin, and 100 µg/mL streptomycin. DMEM with low glucose content equalling 5.55 mM was chosen, because specifically this type of medium was found to be optimal by our team and was used in the screening study of the chosen compounds as described in [25,26,27].

Cells were regularly split using trypsin and subcultured up to 80–90% confluence (3 times per week).

#### 4.1.2. The Culture of SH-SY5Y Cells

A human neuroblastoma SH-SY5Y cell line was used (number in ATCC is CRL-2266).

These cells were cultured in Dulbecco‘s modified Eagle’s medium: Nutrient Mix F-12 (DMEM/F-12, PAN-Biotech GmbH, Aidenbach, Germany, catalogue number P04-41250) supplemented with 15% FBS (as this concentration of FBS in DMEM/F-12 has been demonstrated to be optimal for high proliferation rate of SH-SY5Y cells [74]), 100 U/mL penicillin and 100 µg/mL streptomycin.

Cells were regularly split using trypsin and subcultured up to 80–90% confluence (1–2 times per week); the medium was changed each 3 days.

### 4.2. Treatments

HT-22 cells were seeded 8 × 10^4^ cells in a well of a 12-well plate for 48 h experiment (and at 1.2 × 10^5^ cells in a well of a 12-well plate for 24 h experiment, at 1.5 × 10^5^ cells in a well of a 12-well plate for 24 h for reactive oxygen species measurement, and 2 × 10^5^ cells in a well of a 6-well plate for catalase activity measurement). The cells were allowed to attach and grow under standard conditions in an incubator for 24 h (for 48 h in the case of 6-well plate for catalase activity measurement). After this, the cells were incubated in serum-free medium containing the studied compounds (dissolved in DMSO); an equal volume of DMSO was added to the control wells (final concentration of DMSO in the medium was 0.5%). The solutions of the studied compounds in medium were prepared *ex tempore* immediately prior to addition to the wells.

For the viability assessment, HT-22 cells were incubated for 48 h.

The procedure for SH-SY5Y cells was the same, the medium mentioned above was used, and 2 × 10^5^ cells in a well of a 12-well plate for 24 h experiment were seeded.

For the cytoprotective activity assessment, the cytotoxic agent, namely H_2_O_2_ or sodium glutamate, was added 1 h after replacing the full medium with serum-free medium containing the studied compounds. After this, the cells were incubated for 48 h. On the H_2_O_2_-induced model, additionally, the time point 24 h was also used in the studies of the most effective compound. The additional regimen was also applied with first adding H_2_O_2_, and 1 h later, the studied compound (in this case, the small amount of medium) was replaced with that containing the studied compound and mixed.

In order to establish whether the protective effect is manifested under the conditions of different glucose concentrations in media, in a separate series of the experiment, the cells were seeded to 12-well plates in full DMEM with high glucose content, namely 25 mM (PAN-Biotech GmbH, Aidenbach, Germany, catalogue number P04-03596), as well as this medium with additionally dissolved glucose up to the concentration of 50 mM. The osmotic pressure was adjusted with D-mannitol (25 mM, Sigma-Aldrich, Merck KGaA, Darmstadt, Germany) [42]. Corresponding FBS-free media were used to dissolve the studied substance and incubate the cells. All of these media were additionally filtered after dissolution through 0.22 μm sterile syringe filter (GoogLab, Rokocin, Poland).

As substantiated above, cyanocobalamin (SunFarm solution, 1000 µg/mL) was used as a reference substance on the H_2_O_2_-induced cytotoxicity model in HT-22 cells. According to the results described in the article [30], its protective activity was realized at a wide range of concentrations from 10 nM to 1 mM. This allowed us to use cyanocobalamin at the same concentrations as the studied compound **[MoO_2_(HL^3^)]**, namely, 10 µM, 25 µM, 50 µM, 100 µM, and an additional lower concentration of 1 µM.

### 4.3. H_2_O_2_-Induced Model of Cellular Injury

A 500 µM concentration of H_2_O_2_ in medium was chosen to reproduce the model of cellular injury [33] in HT-22 cells. H_2_O_2_ solution from Sigma-Aldrich (Merck KGaA, Darmstadt, Germany, catalogue number H1009) was used. At the time points described above (after 1-h incubation with the studied compounds), saline solution with H_2_O_2_ (20 mM) was added to each well after the removal of the equivalent volume of medium (2.5% of the well volume) and mixed. After this, the cells were incubated for 48 h (or 24 h in additional experiments), and viability was measured.

Since SH-SY5Y cell line differs in the sensitivity to hydrogen peroxide, after the preliminary studies, 175 µM concentration of H_2_O_2_ in medium was used for these cells (it is within the ranges of concentrations used in the studies [75,76]). The protocol of treatment was similar to that one used in HT-22 cells, but the duration of incubation was 24 h.

### 4.4. Glutamate-Induced Model of Cellular Injury

Glutamate concentrations used in HT-22 cells were chosen according to the data [43], and equalled 5 mM, 7.5 mM and 10 mM. L-glutamic acid monosodium salt hydrate from Sigma-Aldrich (Merck KGaA, Darmstadt, Germany, catalogue number G5889) was used. At the time points described above (after 1-h incubation with the studied compounds), saline solution with glutamate (300 mM) was added to each well after the removal of the equivalent volume of medium (maximum 3.3% of the well volume) and mixed. After this, the cells were incubated for 48 h, after which viability was measured.

It is well known that glutamate causes different levels of cytotoxicity in HT-22 and SH-SY5Y cell lines, and in the studies on the latter concentrations such as 50 mM, 100 mM, 125 mM, and 175 mM [44] were used. The protocol of treatment was similar to that one used in HT-22 cells, but the duration of incubation was 24 h.

### 4.5. Assessment of Cell Viability and the Type of Cell Death

FITC Annexin V Apoptosis Detection Kit I (BD Pharmingen™, Becton, Dickinson and Company, San Diego, CA, USA) was used for apoptosis analysis. After the incubation time, floating and attached cells were collected (the latter after trypsinisation, centrifugation regimen was 1500 rpm, acceleration 9, and deceleration 9 at 4 °C for 5 min), further washed with ice-cold phosphate-buffered saline (PBS) containing 2.5% of fetal bovine serum (FBS), centrifuged at the same regimen and resuspended in binding buffer (0.01 M Hepes/NaOH (pH 7.4), 0.14 M NaCl, and 2.5 mM CaCl_2_). The total of 5 μL of FITC Annexin V and 5 μL of propidium iodine (PI) were added to the cells, gently resuspended once again, and incubated at room temperature in the dark for 15 min.

Before flow cytometry analysis, cells were further diluted in binding buffer, and analysis was carried out using a Guava^®^ easyCyte™ automatic cytometer (Cytek^®^ Biosciences Inc. Fremont, CA, USA) and software provided by this manufacturer (version 4.5.25). Also, 10,000 cell-specific events were analyzed for each experiment. Compensations and quadrants were set up using the control samples as follows: unstained cells, cells stained with FITC Annexin V (no PI), and cells stained with PI (no FITC Annexin V).

Before cell collection for apoptosis assay, microphotographs were taken from the cells seeded in 12-well plates (and additional photos from the cells seeded in 6-well plates) using the microscope Opta-tech MW 50 (OPTA-TECH Sp. z o.o., Warsaw, Poland).

### 4.6. Transmission Electron Microscopy (TEM)

TEM was conducted according to the method described in the article [77] with modifications. The cells were treated with the studied compound and incubated for 48 h, rinsed with PBS and fixed in 2.5% glutaraldehyde. They were post-fixed in a mixture of 1% osmium tetroxide and 1% potassium ferricyanide. After dehydration through a graded ethanol series (30–100%), cells were embedded in EPON resin (Agar Scientific). Ultrathin sections (65 nm) were cut using a Leica UC7 ultramicrotome and stained with Uranyless and Reynold’s lead citrate (Delta Microscopies). Imaging was performed on a Tecnai Spirit BioTWIN TEM (FEI Company, Thermo Fisher Scientific, Waltham, MA, USA) operating at 120 kV. The analyst evaluated the microphotographs without knowing the sample identities.

### 4.7. Cyclic Voltammetry

The interactions between **[MoO_2_(HL^3^)]** and H_2_O_2_ were investigated by cyclic voltammetry (CV). All measurements were carried out using a 2 mM solution of the **[MoO_2_(HL^3^)]** in 0.1 M tetra-*n*-butylammonium tetrafluoroborate (TBABF_4_) in DMSO used as the supporting electrolyte. The total volume of the solution used for the experiments was 2 mL. A 2 mM hydrogen peroxide stock solution was prepared by dilution of 30% aqueous solution of H_2_O_2_ (Stanlab Sp. z o.o., Lublin, Poland) in anhydrous DMSO. All studies were conducted using molar ratios of H_2_O_2_/**[MoO_2_(HL^3^)]** ranging from 0.05 to an excess of 2.5. To minimize dilution errors, all CV measurements were corrected using an appropriate dilution correlation factor. Electrochemical experiments were carried out using a potentiostat (Autolab Metrohm AG, Herisau, Switzerland) in a three-electrode configuration. A glassy carbon electrode (3 mm diameter) served as the working electrode and was polished prior to each measurement using alumina (3 µm) on a polishing cloth (Buehler, Lake Bluff, IL, USA). An Ag|AgCl (3 M KCl) electrode with a double junction system was used as the reference electrode, and the outer compartment was filled with 0.1 M TBABF_4_ in methanol. A platinum wire was employed as the counter electrode. All cyclic voltammograms were acquired at a scan rate of 100 mV/s over a potential range from 0 V–1.6 V, followed by a reverse scan to +1.5 V.

### 4.8. Reactive Oxygen Species Measurement

A 2′,7′-dichlorodihydrofluorescein diacetate (Sigma-Aldrich, Merck KGaA, Darmstadt, Germany, catalogue number D6883, DCF-DA) assay was employed [48,78]. The most appropriate time of analysis and period of incubation were selected in preliminary experiments. After 24-h growth, as described above, the cells were incubated for 1 h with the compound 13 at a concentration of 25 µM; after this, the medium was supplemented with 10 µL of 0.2 mM DCF-DA, the methanol stock solution of which was previously dissolved *ex tempore* in serum-free medium. In the experiments with H_2_O_2_ it was added at a concentration of 500 µM simultaneously with DCF-DA. After a 30 min incubation in the dark at 37 °C, media were discarded and the cells were washed with PBS and collected after trypsinization; centrifugation regimen was 1500 rpm, acceleration 9, and deceleration 9 at 4 °C for 5 min. The cells were washed twice with ice-cold phosphate-buffered saline (PBS) and resuspended in 200 µL of PBS. Fluorescence of oxidized DCF (525/30 nm) was measured by flow cytometry using a Guava^®^ easyCyte™ automatic cytometer (Cytek^®^ Biosciences Inc. Fremont, CA, USA) and software provided by this manufacturer (version 4.5.25). Median fluorescence for a set of gated events was registered and the data were expressed as a percentage of the control cells values which were set as 100%.

### 4.9. Catalase and SOD Activity Measurement

The method for the determination of catalase (CAT) activity and superoxide dismutase (SOD) content described in [79] was used. HT-22 cells were seeded to 6-well plates and incubated with the studied compound and/or hydrogen peroxide as described above. After this, the media were removed, the cells were washed with PBS, trypsinized, and trypsin activity was stopped by PBS containing magnesium and calcium ions. The cell suspension was centrifuged at 1000× *g* for 4 min at room temperature. The supernatant was discarded and the cell pellet was re-suspended in ice-cold potassium phosphate buffer (pH 7.4). The cell suspension was sonicated for 30 s at 5 W using the homogeniser Bandelin Sonopuls mini2 (BANDELIN electronic GmbH & Co. KG, Berlin, Germany) and centrifuged at 10,000× *g* for 10 min at 4 °C. The supernatant was frozen at −80 °C and further used for the measurement of CAT activity, SOD content and protein level.

As described in [79], determination of CAT activity was based on the measurement of the enzyme-catalyzed decomposition of H_2_O_2_ using titration with an excess KMnO_4_. Modifications were made for the quantity of cell lysates and time of incubation, since it is generally known that CAT activity in neural cells is lower than in the liver cells used in the original study [79]. As recommended in the cited study, protein content in the supernatants of cell lysates was determined according to the method described by Bradford using bovine serum albumin as the standard. The results were expressed as μM H_2_O_2_/min/mg protein.

We aimed to exclude the possibility of chemical interference of the studied compound with the methods used for enzymatic assays as described below.

The changes in optical density at the same conditions (490 nm) were assessed after mixing DMSO stock solution of the leader compound with HT-22 cells lysates (prepared by the similar procedure as in all experiments, but from untreated cells), phosphate buffer, saline, sulphuric acid and excess KMnO_4_ (similar conditions to those used during catalase assessment). The samples were incubated for 3 min; in order to increase the probability of revealing any changes, the duration was increased to 15 min. In both cases, no statistically significant changes were registered after using the following concentrations of the studied compound: 2.5 μM, 5 μM, 12,5 μM, and 25 μM. (Namely, after 3-min incubation, the changes in activity compared to the control value (100 %) equalled 102%, 99%, 68%, 90% at the mentioned concentrations respectively. After 15-min incubation, the corresponding values equalled 110%, 123%, 99%, 97%. These differences did not reach the level of statistical significance).

The changes in optical density at the same conditions (490 nm) were also registered after mixing DMSO stock solution of the leader compound with phosphate buffer, saline, sulphuric acid and excess KMnO_4_ (similar conditions to those used during catalase assessment).

No significant changes in optical density were seen at concentrations of the studied compound 2.5 μM and 25 μM in the added samples, while there was a slight albeit statistically significant decrease in optical density at the concentrations of compound 13 of 5 μM and 12.5 μM (on 10% and 7% compared with the control value, respectively). This moderate decrease in optical density probably could not cause bias in the results of catalase activity measured.

The decreased optical density in catalase assay is generally seen when more hydrogen peroxide was left unmetabolized after incubation and thus further reacted with KMnO_4_. Such a situation was not evident in the samples of the cells incubated with the studied compounds, several of which, on the contrary, demonstrated increased optical density (and increased catalase activity). As seen from the previous paragraphs, this increment is probably not caused by the direct interference of the studied compound with the chemical principle of the method nor by the direct influence on hydrogen peroxide in the reaction mixture or on catalase activity within it.

SOD content was measured based on the reduction in the rate of autoxidation of pyrogallol in DTPA/tris-buffer solution (pH 8.2) in the presence of the studied cell lysates (their quantity was also increased compared with the original study [79]). Immediately after adding pyrogallol solution to the studied samples and mixing, absorbance was measured at 560 nm every 30 s for 4.5 min. Linear segments were determined on the obtained curves (similar for the control and corresponding experimental samples) and the change in absorbance was calculated. One unit of SOD was defined as the amount of enzyme necessary to inhibit 50% of the reaction, one SOD unit was accepted as 125 ng SOD, and the values were normalized as ng SOD/mg protein [79].

The results of the possible influence of the studied compound per se on pyrogallol autoxidation were also checked (this method can also be used to estimate direct antioxidant activity of compounds). The same procedure as described above was used but instead of cell lysates solutions of the studied compounds were used. Analysis of the curves obtained at both 560 nm and 420 nm (the latter was additionally used since the use of this wavelength is described for the study of pyrogallol autoxidation in the presence of metal complexes with organic ligands [80]) at pH 8.2 and 7.4 (the latter is recommended for the in vitro assay of the antioxidant effect [81]) showed that there was no evidence of the inhibitory influence of the studied compound on pyrogallol autoxidation.

### 4.10. Statistical Analysis

Data are presented as mean ± SEM. At least three separate replicates were done for each assay. GraphPad Prism 9 software (GraphPad Software, Inc., Boston, MA, USA) was used to perform statistical analysis. One-way analysis of variance (ANOVA) with Tukey’s post hoc test was used, and for the data not corresponding to the normal distribution (assessed by Shapiro–Wilk test), mostly the enzyme activity, the Friedman test with Dunn’s multiple comparisons test was used.

For the analysis of the cell viability, which was always made with the synchronous controls (since simultaneous measurement of all concentrations in a single dataset was not possible), as well as for the certain enzymatic assays paired *t*-test or Wilcoxon, matched pair test was used to establish the differences with the synchronous control groups.

## 5. Conclusions

Thus, among molybdenum(VI) Schiff base complexes, the substance with an excellent cytoprotective activity has been identified, namely **[MoO_2_(HL^3^)]**. Its safety and cytoprotective activity were studied in two cell lines, namely HT-22 and SH-SY5Y. The range of active cytoprotective concentrations in hydrogen peroxide-induced injury in HT-22 cells is from 5 µM to 50 µM. At the most active concentration of 25 µM, the protective effect is present at different levels of glucose in the medium and is also seen in SH-SY5Y cells. In HT-22 cells, **[MoO_2_(HL^3^)]** is also active against glutamate-induced injury. Among the possible mechanism of **[MoO_2_(HL^3^)]** cytoprotective activity, there is a direct interaction with hydrogen peroxide, decrease in reactive oxygen species generation and maintenance of catalase activity.

## Data Availability

The primary data supporting the findings of this study are available from the corresponding author on reasonable request.

## Supplementary Materials

The following supporting information can be downloaded at: https://www.mdpi.com/article/10.3390/molecules31162883/s1, Supplementary File S1: The effects of [(+)MoO_2_(HL^5^)], [(+)MoO_2_(HL^1^)(CH_3_OH)], [MoO_2_(HL^5^)], [MoO_2_(HL^6^)], [MoO_2_(HL^4^)] on HT-22 cells viability under the conditions of H_2_O_2_-induced cytotoxicity (500 µM, 48 h); Supplementary File S2: The effects of [MoO_2_(HL^3^)], [MoO_2_(HL^2^)] and the reference drug cyanocobalamin on HT-22 cells viability under the conditions of H_2_O_2_-induced cytotoxicity (500 µM, 48 h); Supplementary File S3: The effects of [(+)MoO_2_(HL^5^)], [(+)MoO_2_(HL^1^)(CH_3_OH)], [MoO_2_(HL^5^)], [MoO_2_(HL^6^)], [MoO_2_(HL^4^)] per se on HT-22 cells viability (48 h); Supplementary File S4: The effects of [MoO_2_(HL^3^)] and [MoO_2_(HL^2^)] per se on HT-22 cells viability (48 h); Supplementary File S5: The effects of [MoO_2_(HL^3^)] on HT-22 cells viability in the media with the different levels of glucose; Supplementary File S6: The effects of [MoO_2_(HL^3^)] per se on the viability of SH-SY5Y cells; Supplementary File S7: The effects of [MoO_2_(HL^3^)] on the viability of SH-SY5Y cells under the conditions of H_2_O_2_-induced cytotoxicity; Supplementary File S8: The effects of [MoO_2_(HL^3^] and [MoO_2_(HL^2^)] per se at concentrations lower than 10 µM on HT-22 cells viability (48 h), the additional graphs representing the percentage of the viable cells.

## Abbreviations

The following abbreviations are used in this manuscript:

CAT	Catalase
CV	Cyclic voltammetry
DCF-DA	2′,7′-dichlorodihydrofluorescein diacetate
DMEM	Dulbecco′s modified Eagle′s medium
DTPA	Diethylenetriaminepentaacetic acid
FBS	Fetal bovine serum
HO-1	Heme oxygenase 1
Nrf2	Nuclear Factor Erythroid 2-Related Factor 2
PBS	Phosphate-buffered saline
SOD	Superoxide dismutase
TBABF_4_	tetra-*n*-butylammonium tetrafluoroborate
TEM	Transmission electron microscopy
