# Supplementary material for: Structure-Dependent Cytotoxic and Cytoprotective Effects of Molybdenum(VI) Schiff Base Complexes in the Neuronal Cells In Vitro
Source: Molecules. 2026 Aug 18;31(16):2883. doi: 10.3390/molecules31162883 (PMC13515801; doi:10.3390/molecules31162883)
Supplement: Supplementary file 1 [file molecules-31-02883-s001.zip › SUPPLEMENTARY FILE S1.pdf]

Supplementary file 1.

The effects of [(+)$MoO_2(HL^5)$], [(+)$MoO_2(HL^1)(CH_3OH)$], [$MoO_2(HL^5)$], [$MoO_2(HL^6)$], [$MoO_2(HL^4)$] on HT-22 cells viability under the conditions of H_2O_2-induced cytotoxicity (500 µM, 48 hours)

1. [$MoO_2(HL^5)$]

Microphotographs of control HT-22 cells and the cells under the conditions of H_2O_2-induced cytotoxicity incubated with [$MoO_2(HL^5)$] for 48 hours.

**Control**<br><br>A1,A2.<br>Typical morphological structure of the control cells, which are attached, the cells shape is unchanged.	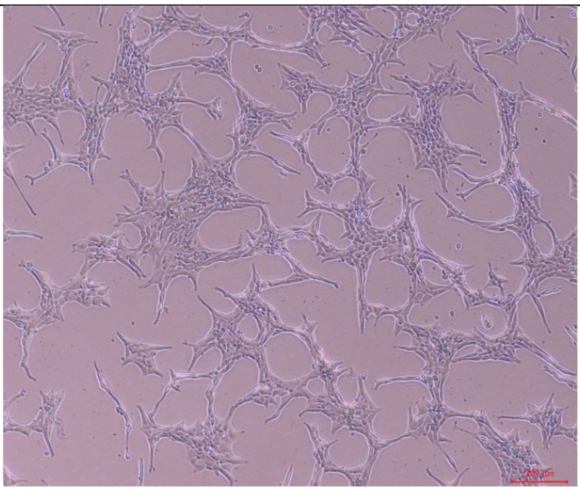<br>A1	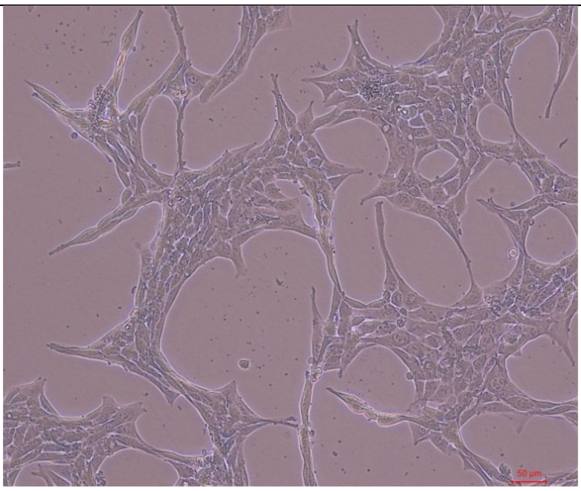<br>A2
**H_2O_2, 500 µM**<br><br>B1,B2. Significant reduction in the quantity of visible attached cells and an increase in the quantity of damaged cells which are round-shaped, detached and floating.	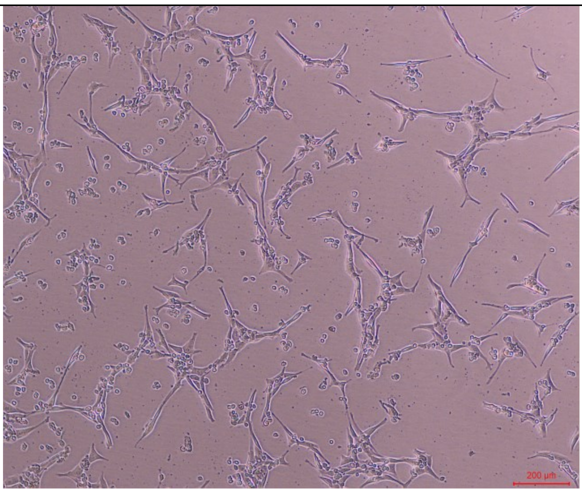<br>B1	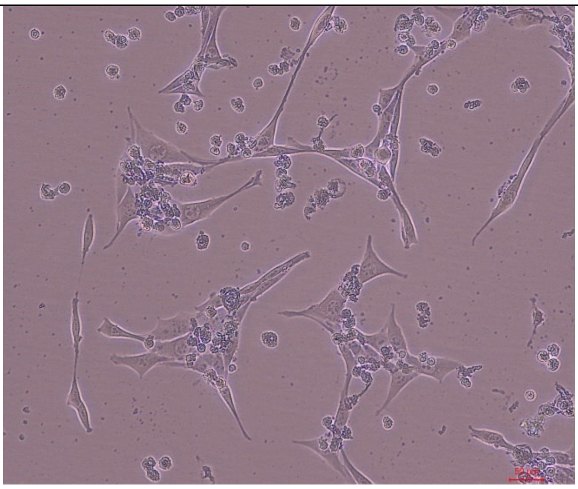<br>B2
**[$MoO_2(HL^5)$], 10 µM, + H_2O_2, 500 µM**<br>C1,C2. The typical morphological structure of the cells is maintained, the cells are attached, the cells shape is unchanged, there are no visible changes in cell confluence compared with the microphotographs of the control cells.	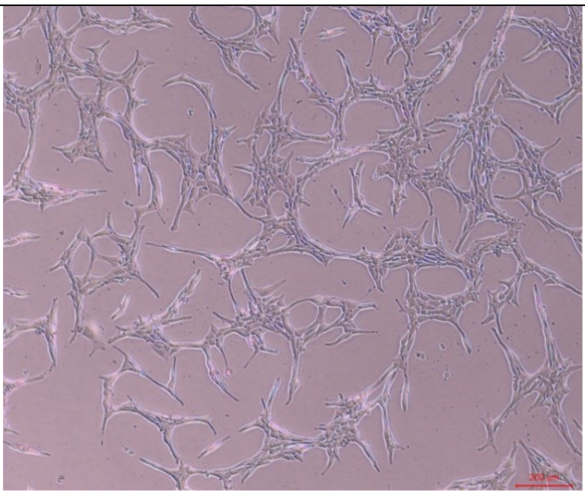<br>C1	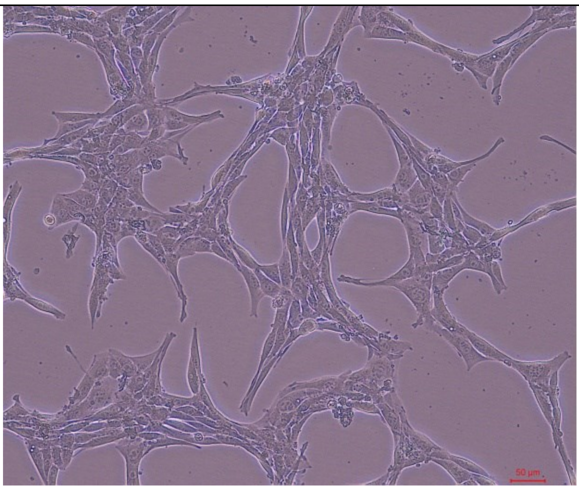<br>C2

**[$MoO_2(HL^5)$], 25 µM + H_2O_2, 500 µM**<br><br>D1,D2. The typical morphological structure of the cells is maintained, but the cell confluence is decreased and the damaged cells are present, which are round-shaped, detached and floating.	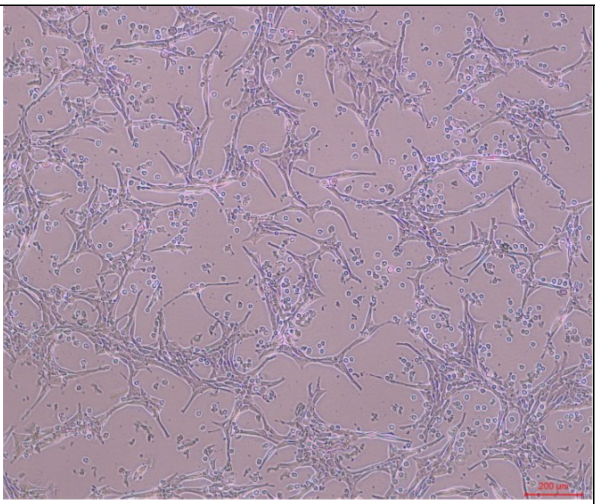<br>D1	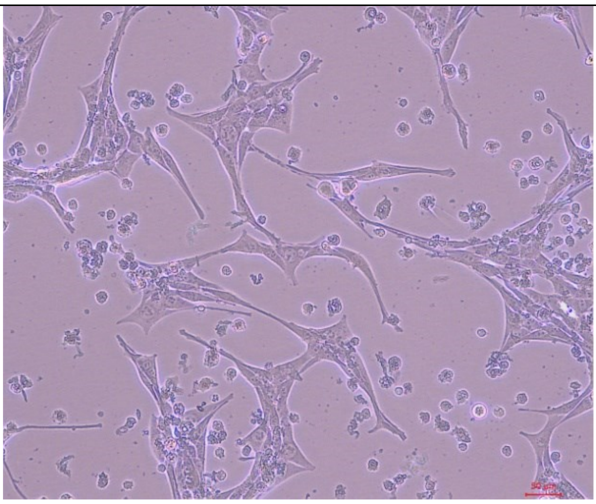<br>D2
**[$MoO_2(HL^5)$], 50 µM + H_2O_2, 500 µM**<br><br>E1,E2. Significant reduction in the quantity of visible attached cells and an increase in the quantity of damaged cells which are round-shaped, detached and floating.	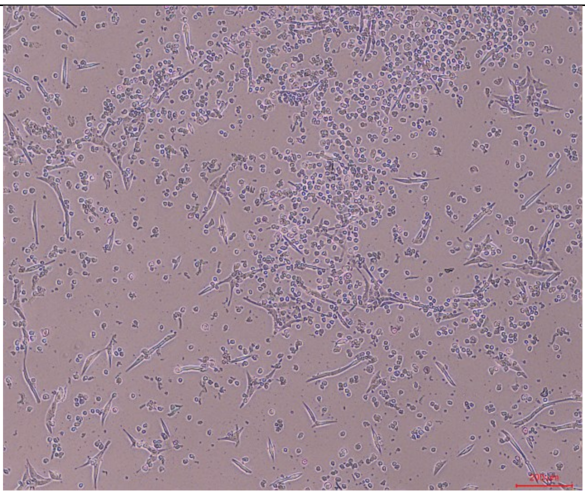<br>E1	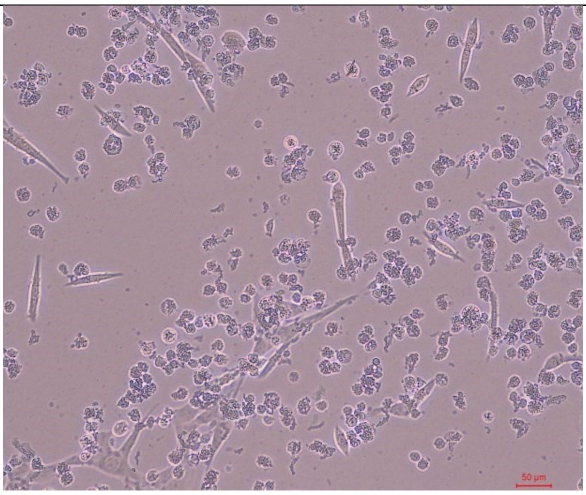<br>E2
**[$MoO_2(HL^5)$], 100 µM + H_2O_2, 500 µM**<br><br>F1,F2. Significant reduction in the quantity of visible attached cells (only single normal cells in the field of vision), a plethora of the severely damaged cells and cell debris.	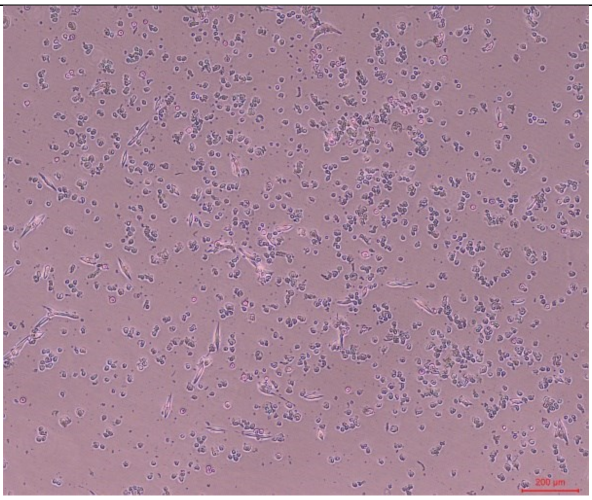<br>F1	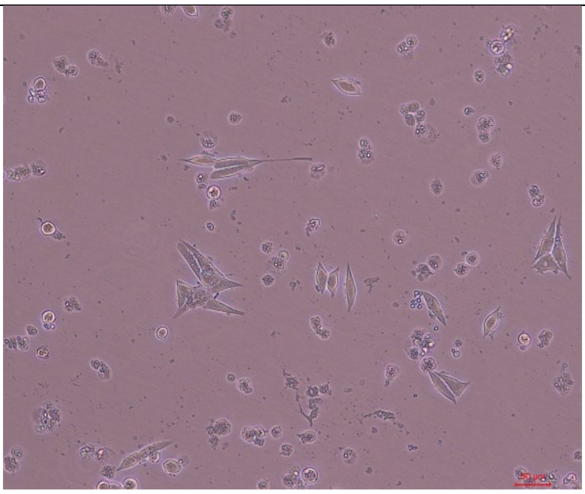<br>F2

2. $[MoO_2(HL^6)]$

2.1. Microphotographs of control HT-22 cells and the cells under the conditions of H_2O_2-induced cytotoxicity incubated with $[MoO_2(HL^6)]$ (25 µM; 50 µM; 100 µM) for 48 hours.

**Control**<br><br>A1,A2. Typical morphological structure of the control cells, which are attached, the cells shape is unchanged.	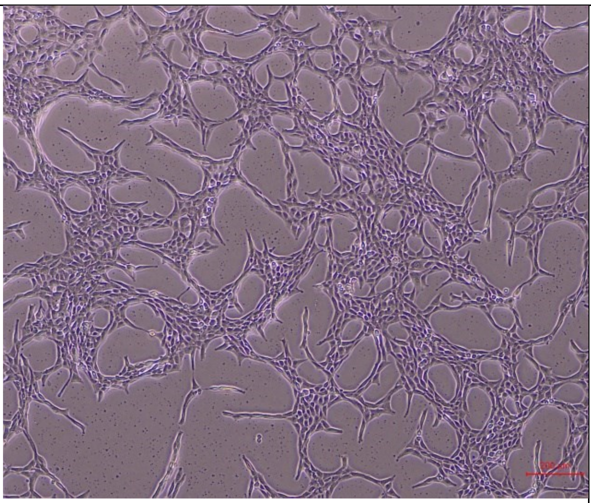<br>A1	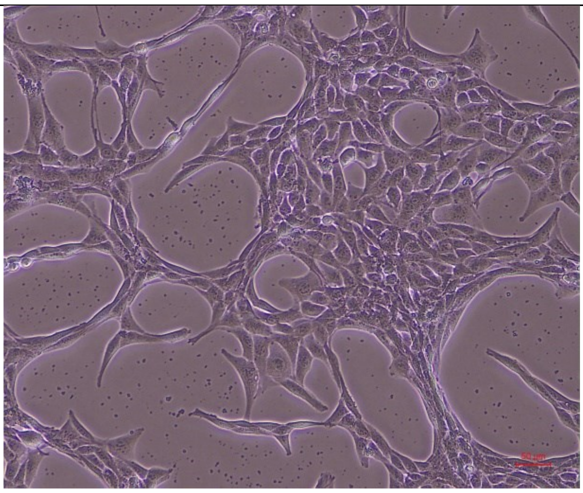<br>A2
**H_2O_2, 500 µM**<br><br>B1,B2. Significant reduction in the quantity of visible attached cells and an increase in the quantity of the damaged cells which are round-shaped, detached and floating.	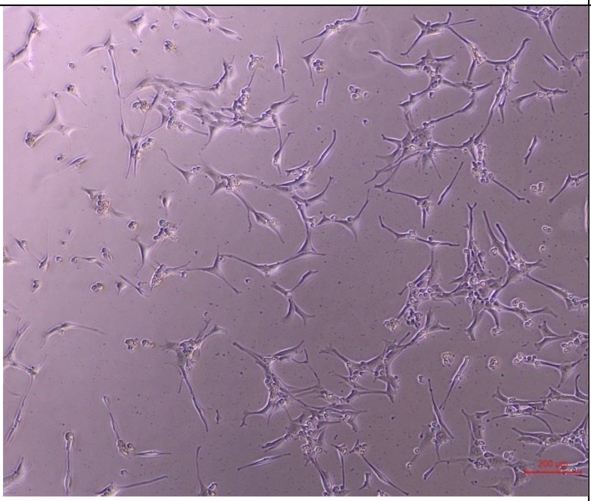<br>B1	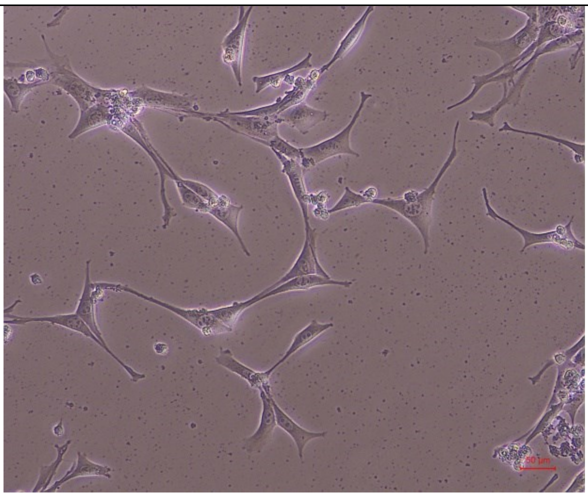<br>B2
**$[MoO_2(HL^6)]$, 25 µM + H_2O_2, 500 µM**<br><br>C1,C2. Significant reduction in the quantity of visible attached cells and presence of damaged cells which are round-shaped, detached and floating.	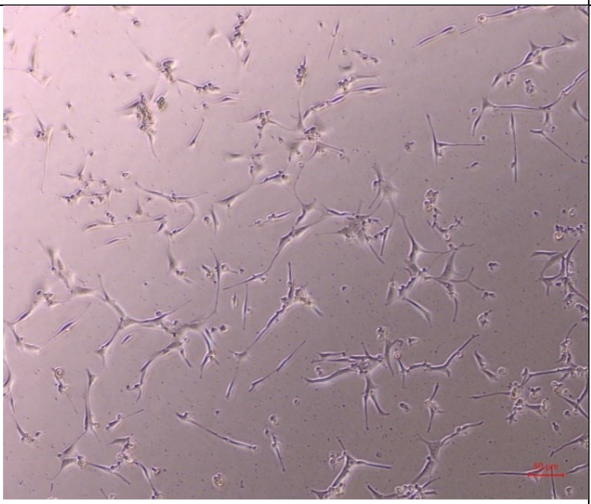<br>C1	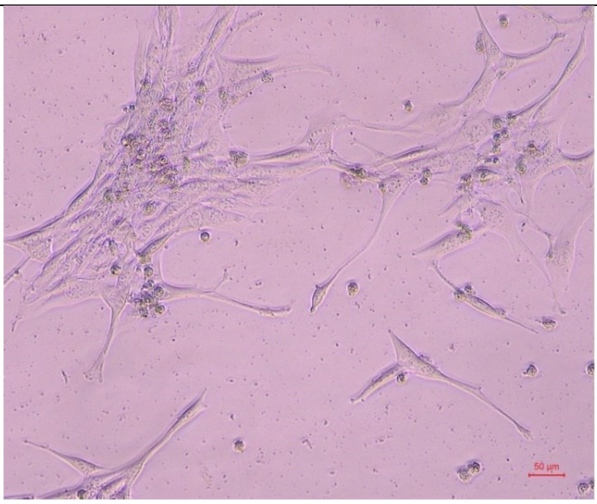<br>C2

**[$MoO_2(HL^6)$], 50 µM + H_2O_2, 500 µM**<br><br>D1,D2. Significant reduction in the quantity of visible attached cells and presence of damaged cells which are round-shaped, detached and floating.	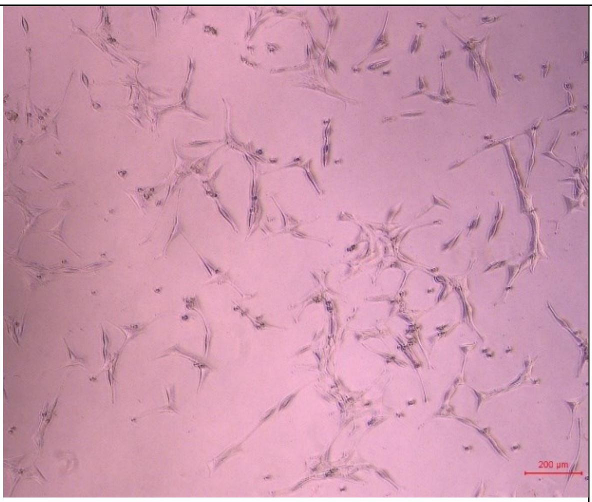<br>D1	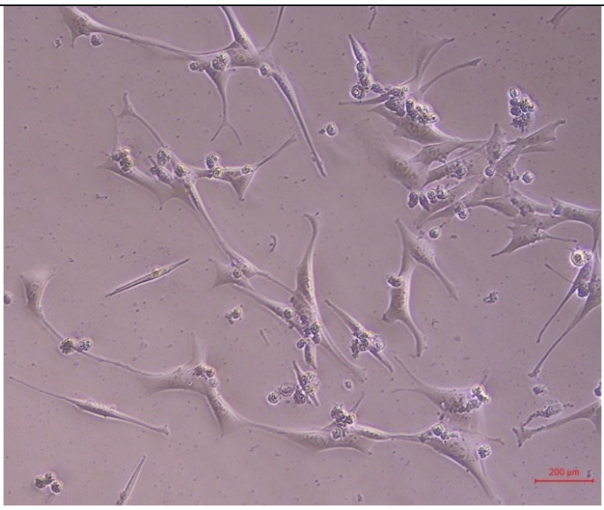<br>D2
**[$MoO_2(HL^6)$], 100 µM + H_2O_2, 500 µM**<br><br>E1,E2. Significant reduction in the quantity of visible attached cells and presence of damaged cells which are round-shaped, detached and floating.	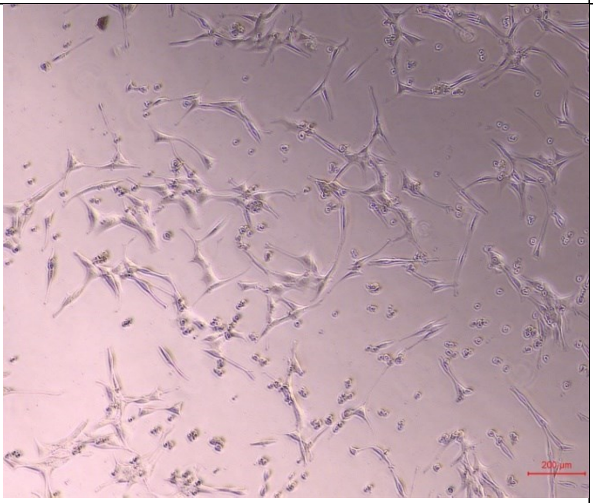<br>E1	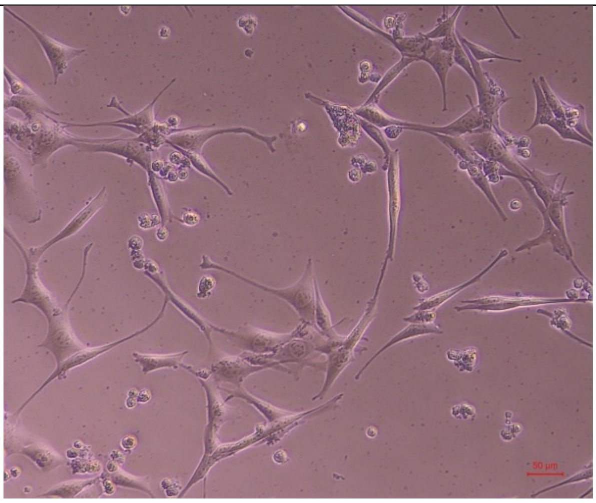<br>E2

2.2. Microphotographs of control HT-22 cells and the cells under the conditions of H_2O_2-induced cytotoxicity incubated with $[MoO_2(HL^6)]$ (200 µM) for 48 hours.

Since not all concentrations were accessed at once, the correspondent representative control photos are given in each dataset.

**Control**<br><br>F1,F2. Typical morphological structure of the control cells, which are attached, the cells shape is unchanged.	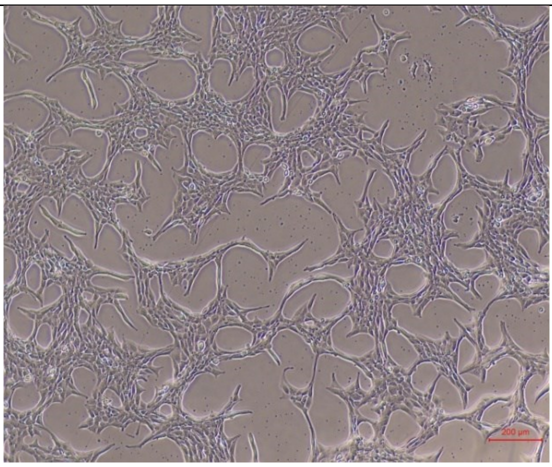<br>F1	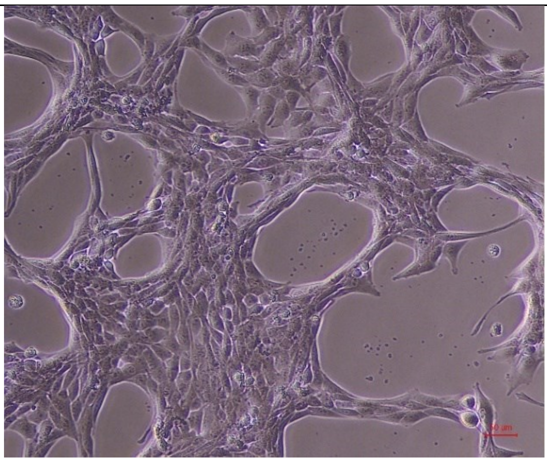<br>F2
**H_2O_2,**<br>**500 µM**<br><br>G1,G2. Significant reduction in the quantity of visible attached cells and presence of damaged cells which are round-shaped, detached and floating.	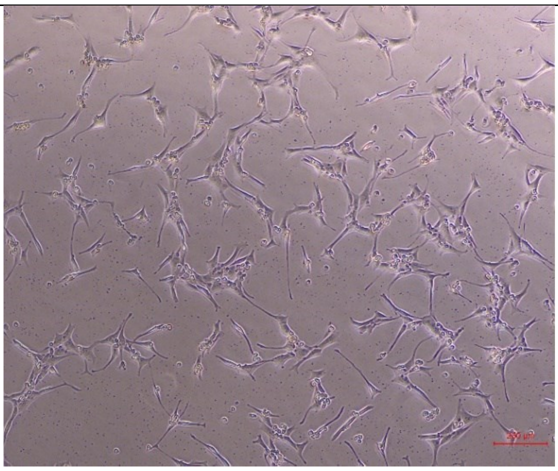<br>G1	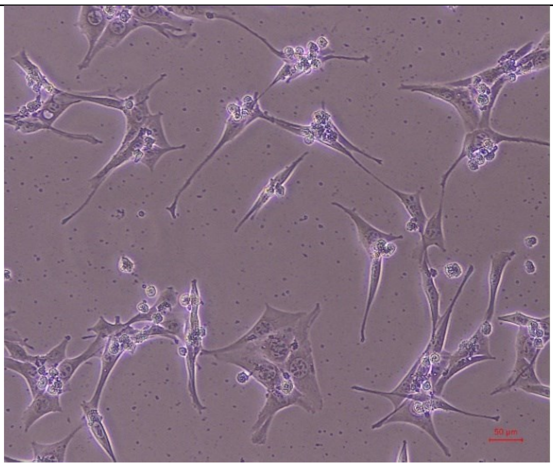<br>G2
**$[MoO_2(HL^6)]$,**<br>**200 µM**<br>**+ H_2O_2, 500 µM**<br><br>H1,H2. The typical morphological structure of the cells is maintained, the cells are attached, the cells shape is unchanged, cell confluence is significantly restored compared with the microphotographs G1,G2, only single damaged cells are present which are round-shaped, detached and floating.	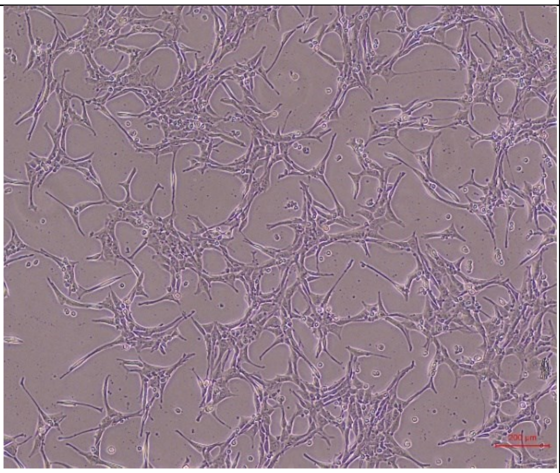<br>H1	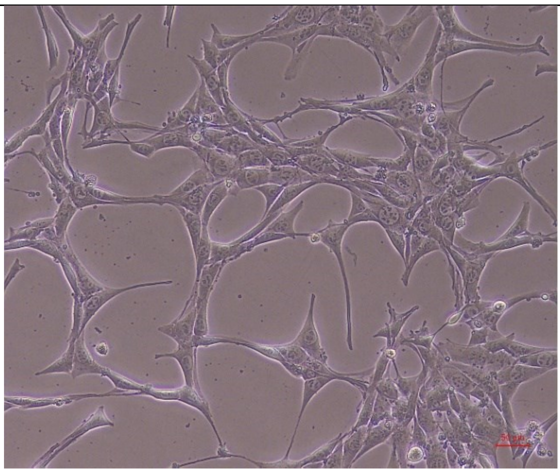<br>H2

3. $[(+)MoO_2(HL^5)]$

3.1. Microphotographs of control HT-22 cells and the cells under the conditions of H_2O_2-induced cytotoxicity incubated with $[(+)MoO_2(HL^5)]$ (10 µM; 25 µM; 50 µM) for 48 hours.

**Control**<br><br>A1,A2. Typical morphological structure of the control cells, which are attached, the cells shape is unchanged.	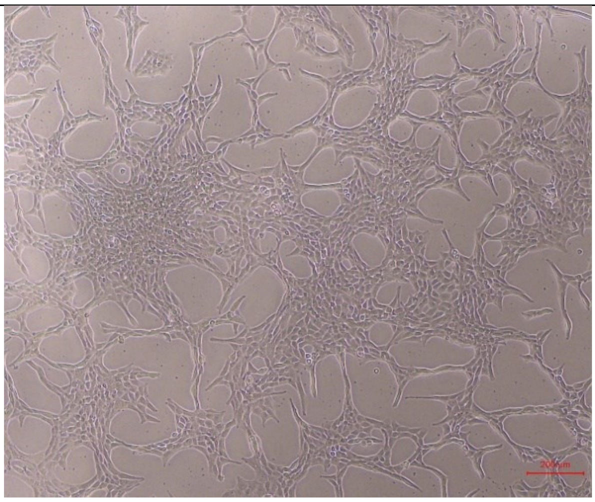<br>A1	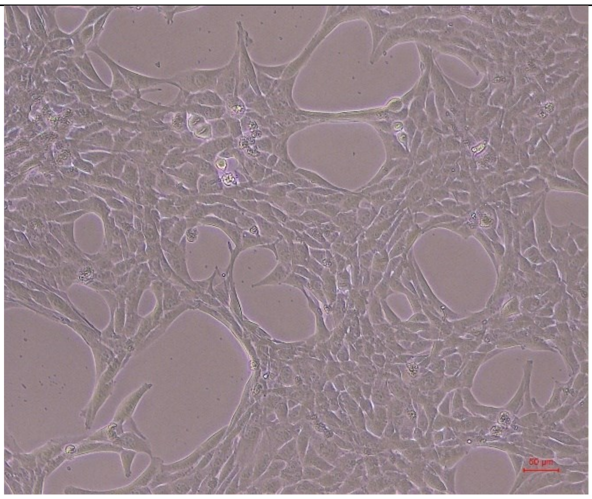<br>A2
**H_2O_2, 500 µM**<br><br>B1,B2. Significant reduction in the quantity of visible attached cells and presence of the damaged cells which are round-shaped, detached and floating.	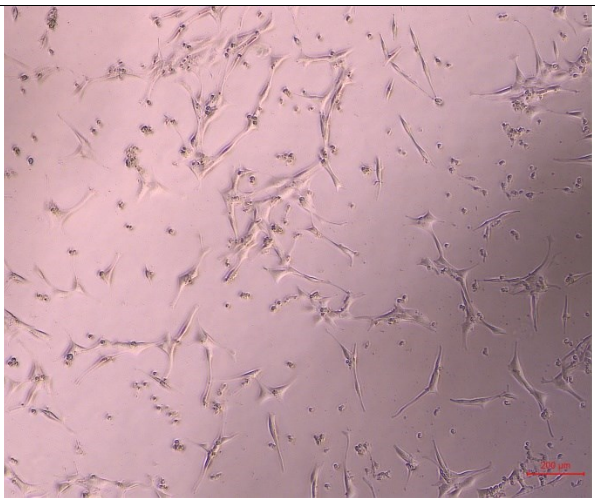<br>B1	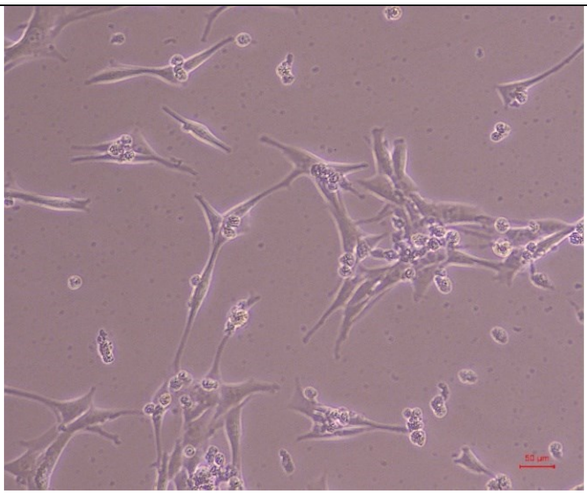<br>B2
**$[(+)MoO_2(HL^5)]$, 10 µM + H_2O_2, 500 µM**<br>C1,C2. Significant reduction in the quantity of visible attached cells and presence of the damaged cells which are round-shaped, detached and floating. No signs of significant improvement compared with the microphotographs B1,B2.	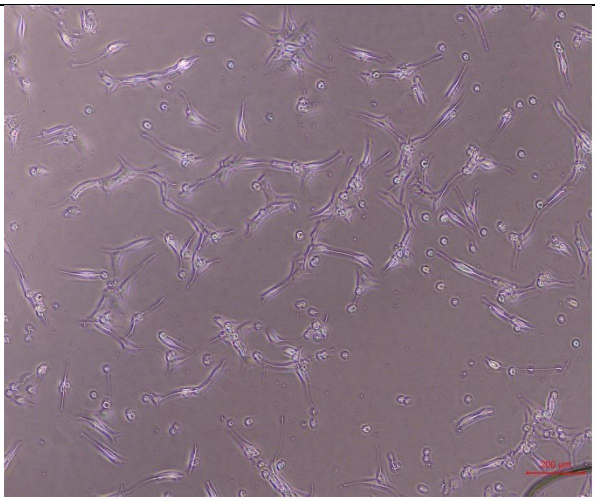<br>C1	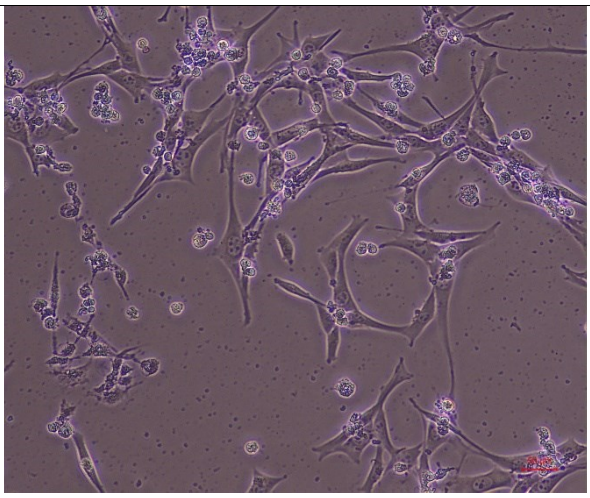<br>C2

**$[(+)MoO_2(HL^5)]$, 25 µM + H_2O_2, 500 µM**<br><br>D1,D2. Significant reduction in the quantity of visible attached cells and presence of the damaged cells which are round-shaped, detached and floating. No signs of significant improvement compared with the microphotographs B1,B2.	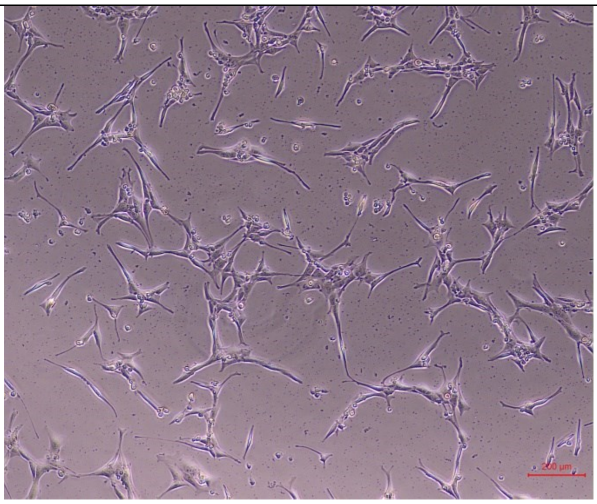<br>D1	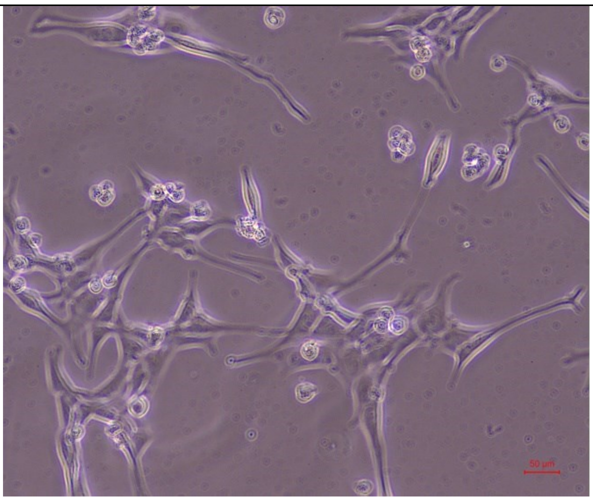<br>D2
**$[(+)MoO_2(HL^5)]$, 50 µM + H_2O_2, 500 µM**<br><br>E1,E2. Significant reduction in the quantity of visible attached cells and presence of the damaged cells which are round-shaped, detached and floating. No signs of significant improvement compared with the microphotographs B1,B2.	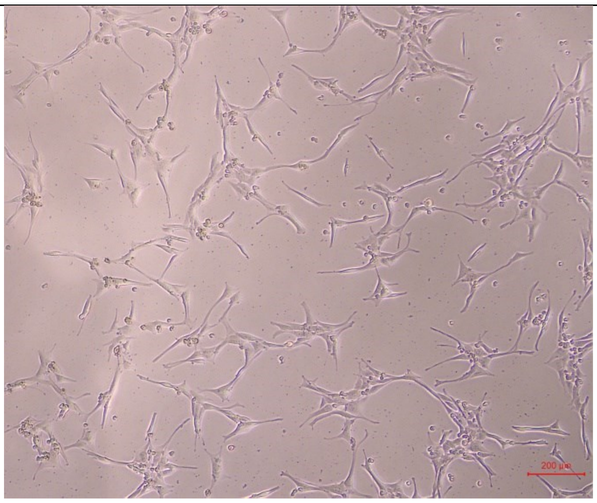<br>E1	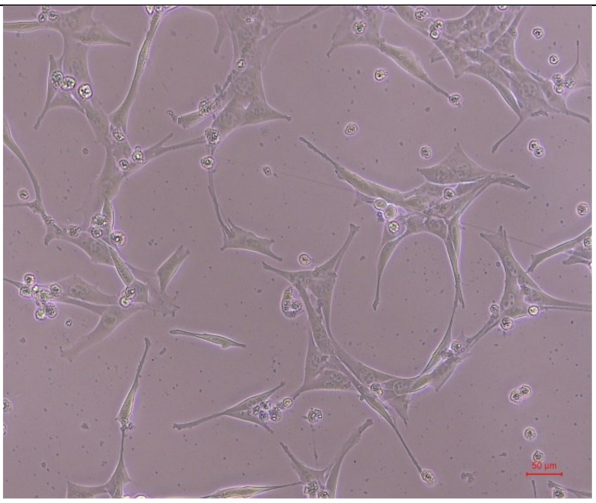<br>E2

3.2. **Microphotographs of control HT-22 cells and the cells under the conditions of H_2O_2-induced cytotoxicity incubated with [(+)$MoO_2(HL^5)$] (100 µM) for 48 hours**

Since not all concentrations were accessed at once, the correspondent representative control photos are given in each dataset.

**Control**<br><br>F1,F2. Typical morphological structure of the control cells, which are attached, the cells shape is unchanged.	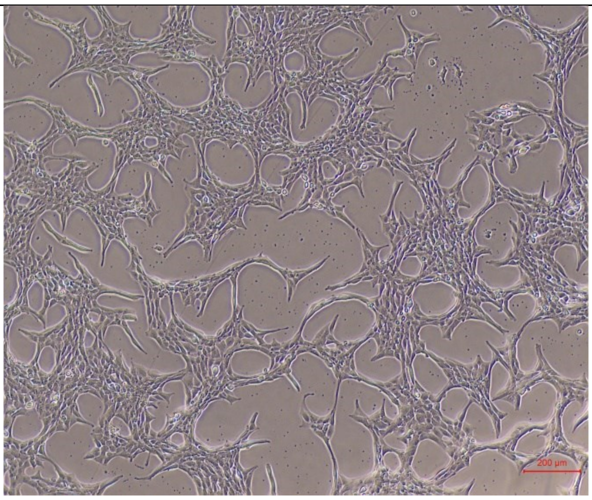<br>F1	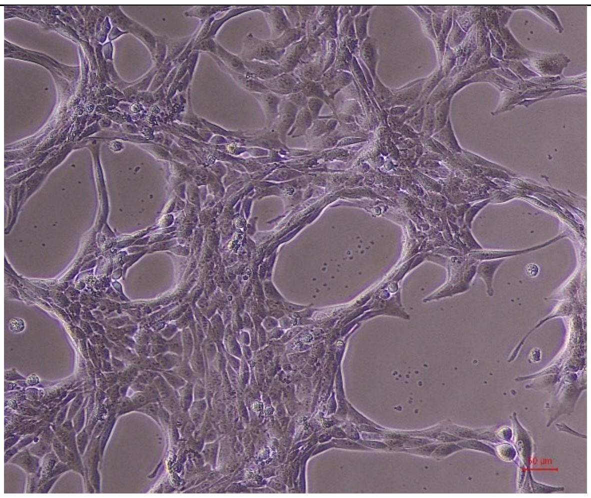<br>F2
**H_2O_2,<br>500 µM**<br><br>G1,G2. Significant reduction in the quantity of visible attached cells and presence of the damaged cells which are round-shaped, detached and floating.	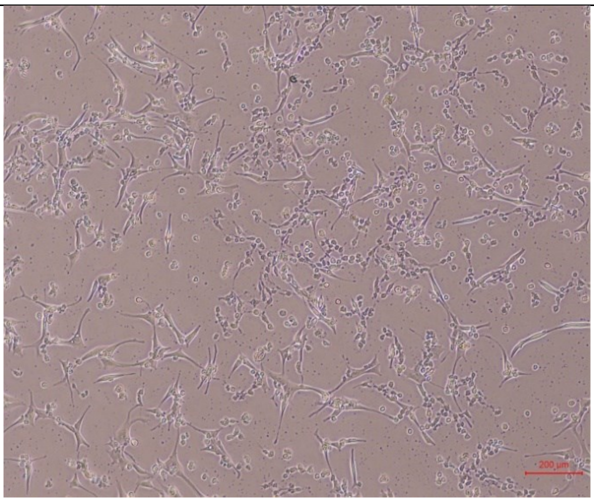<br>G1	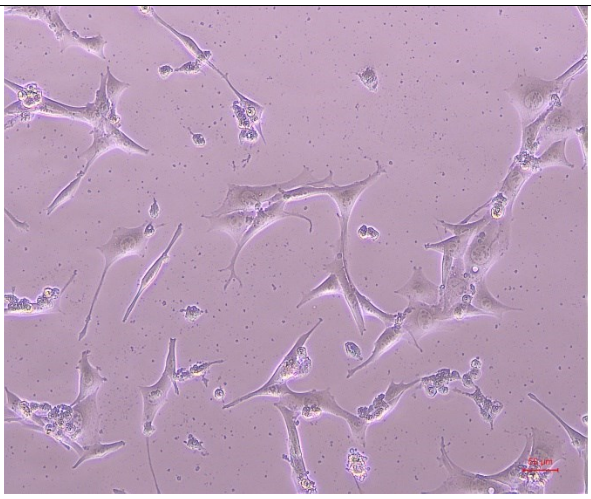<br>G2
**[(+)$MoO_2(HL^5)$],<br>100 µM<br>+ H_2O_2,<br>500 µM**<br>H1,H2. Significant reduction in the quantity of visible attached cells and presence of the damaged cells which are round-shaped, detached and floating. No signs of significant improvement compared with the microphotographs G1,G2.	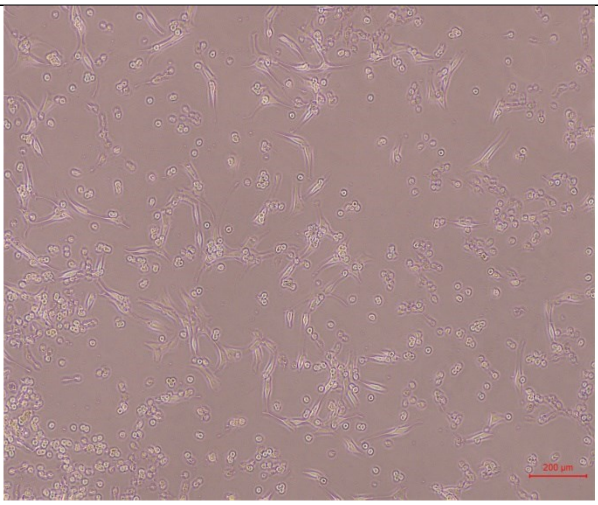<br>H1	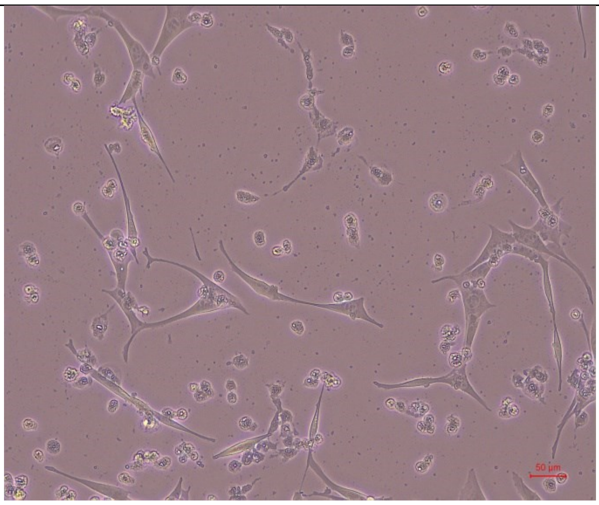<br>H2

4. [(+)$MoO_2(HL^1)(CH_3OH)$]

4.1. Microphotographs of control HT-22 cells and the cells under the conditions of H_2O_2-induced cytotoxicity incubated with [(+)$MoO_2(HL^1)(CH_3OH)$] (10 µM; 25 µM; 50 µM) for 48 hours

**Control**<br><br>A1,A2. Typical morphological structure of the control cells, which are attached, the cells shape is unchanged.	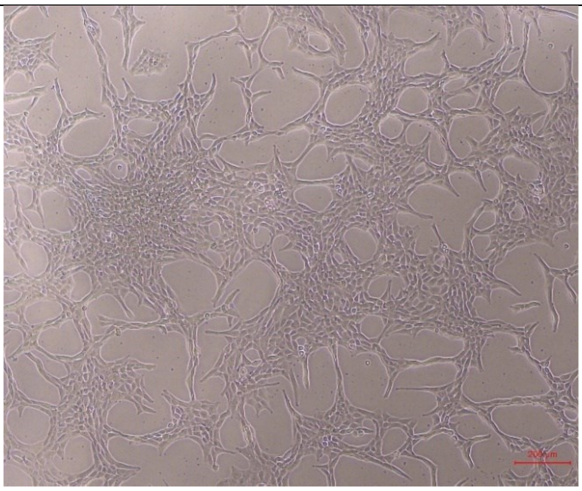<br>A1	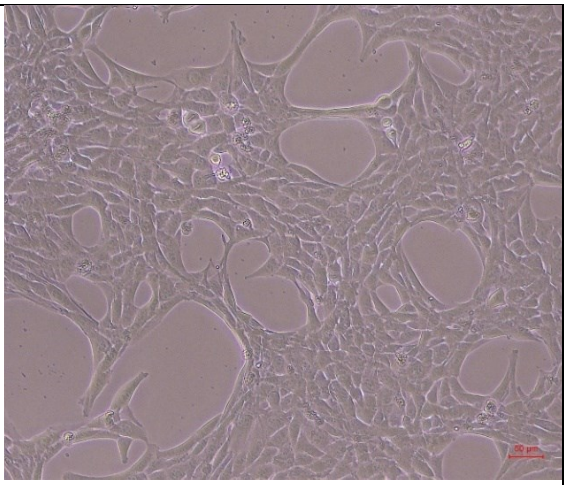<br>A2
**H_2O_2, 500 µM**<br><br>B1,B2. Significant reduction in the quantity of visible attached cells and presence of the damaged cells which are round-shaped, detached and floating.	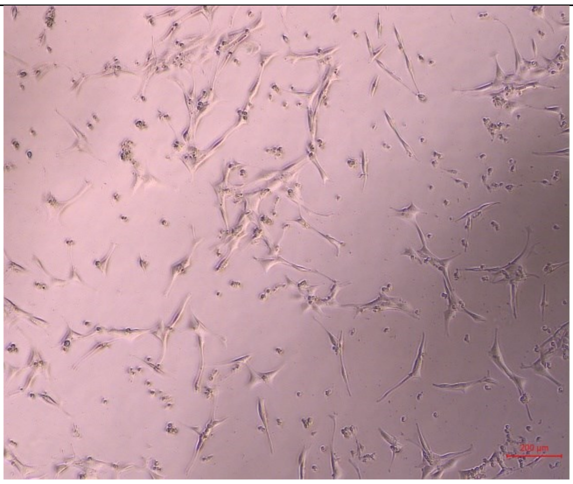<br>B1	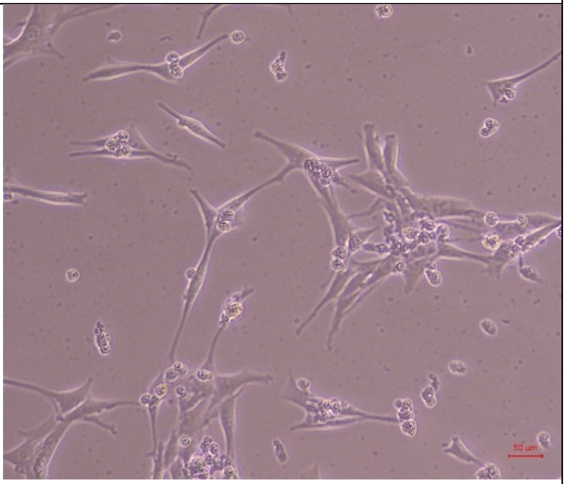<br>B2
**[(+)$MoO_2(HL^1)$ (CH_3OH)] , 10 µM + H_2O_2, 500 µM**<br><br>C1,C2. Significant reduction in the quantity of visible attached cells and presence of the damaged cells which are round-shaped, detached and floating.	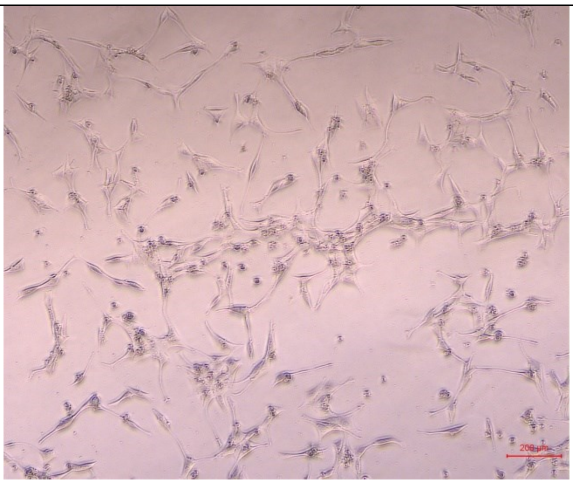<br>C1	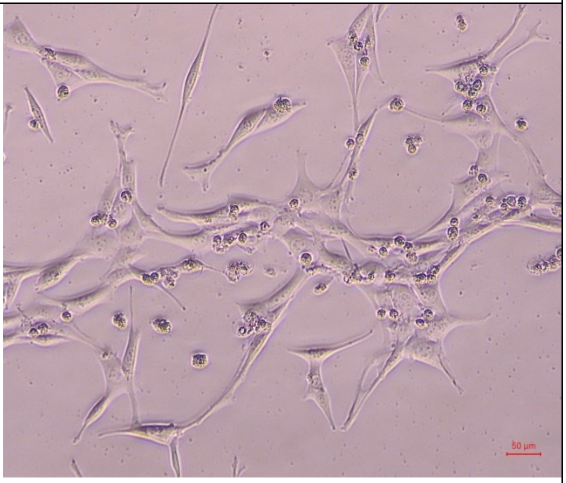<br>C2

**[(+)$MoO_2(HL^1)$ (CH_3OH)], 25 μM + H_2O_2, 500 μM**<br>D1,D2. Significant reduction in the quantity of visible attached cells and presence of the damaged cells which are round-shaped, detached and floating.	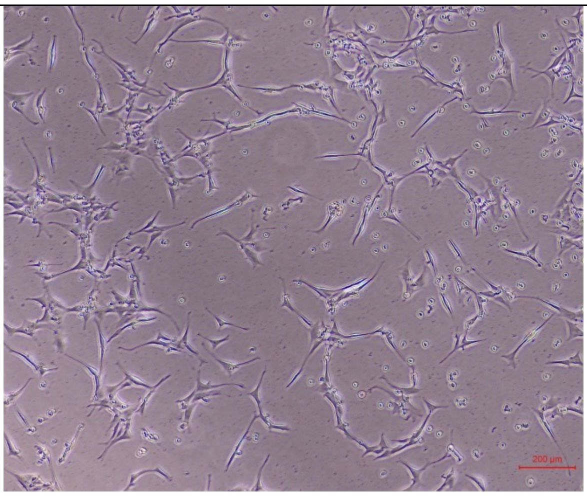<br>D1	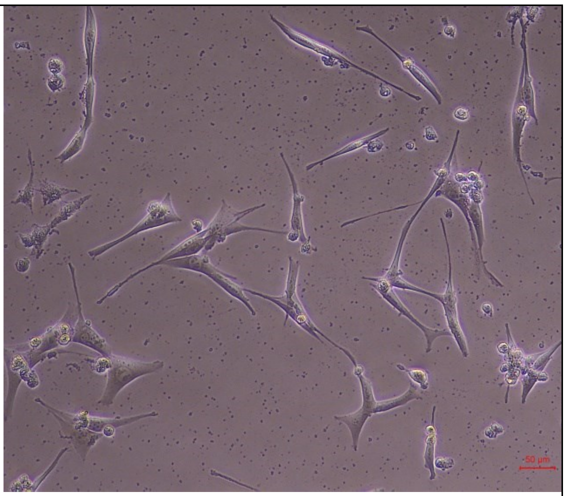<br>D2
**[(+)$MoO_2(HL^1)$ (CH_3OH)], 50 μM + H_2O_2, 500 μM**<br>E1,E2. Significant reduction in the quantity of visible attached cells and presence of the damaged cells which are round-shaped, detached and floating.	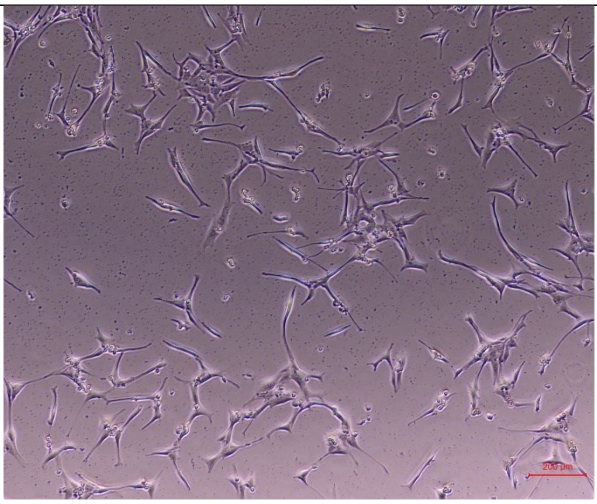<br>E1	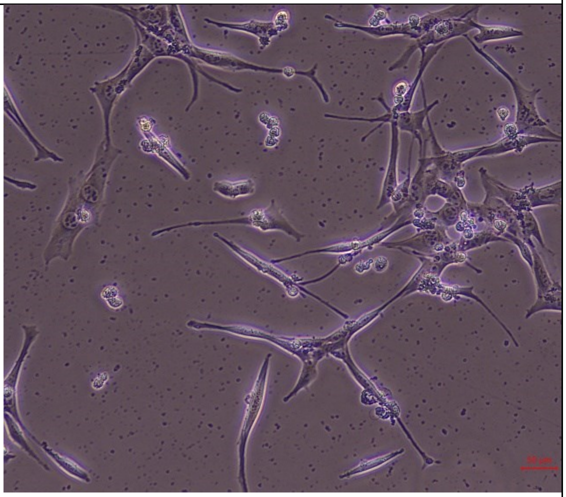<br>E2

4.2. Microphotographs of control HT-22 cells and the cells under the conditions of H_2O_2-induced cytotoxicity incubated with $[(+)MoO_2(HL^1)$ $(CH_3OH)]$ (100 µM) for 48 hours

Since not all concentrations were accessed at once, the correspondent representative control photos are given in each dataset.

**Control**<br><br>F1,F2. Typical morphological structure of the control cells, which are attached, the cells shape is unchanged.	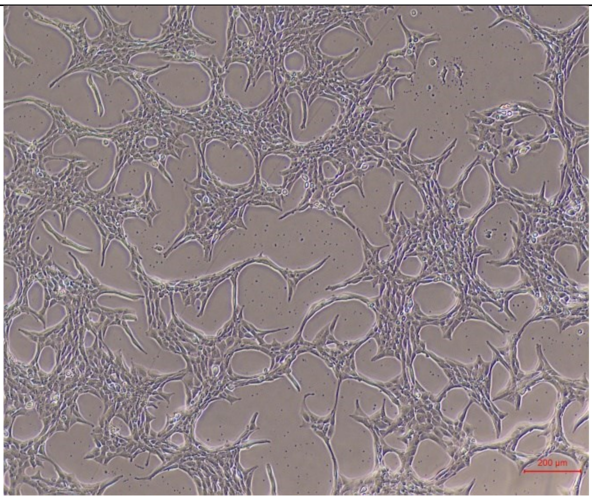<br>F1	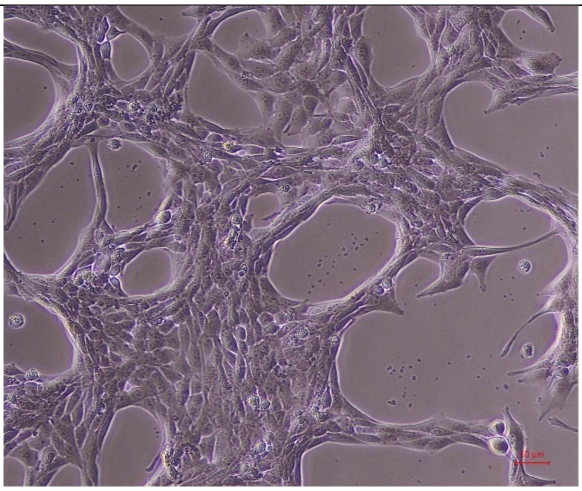<br>F2
**H_2O_2, 500 µM**<br><br>G1,G2. Significant reduction in the quantity of visible attached cells and presence of the damaged cells which are round-shaped, detached and floating.	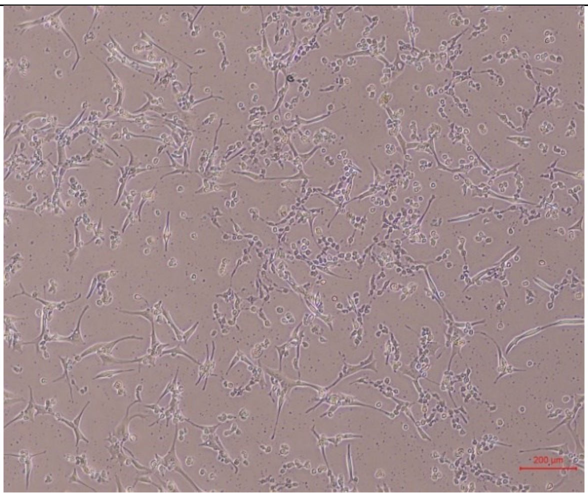<br>G1	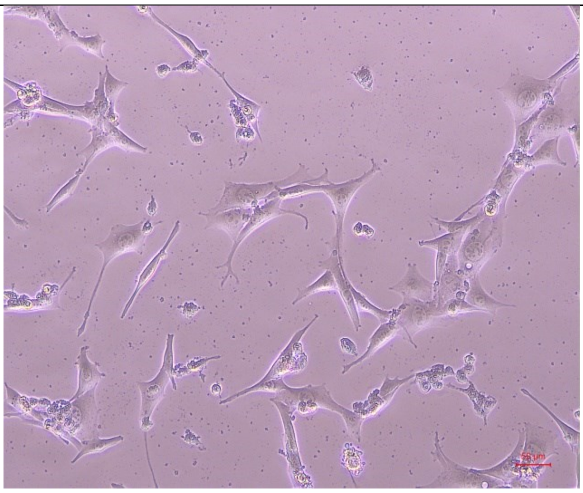<br>G2
**$[(+)MoO_2(HL^1)$ $(CH_3OH)]$, 100 µM + H_2O_2, 500 µM**<br><br>H1,H2. Significant reduction in the quantity of visible attached cells and presence of the damaged cells which are round-shaped, detached and floating.	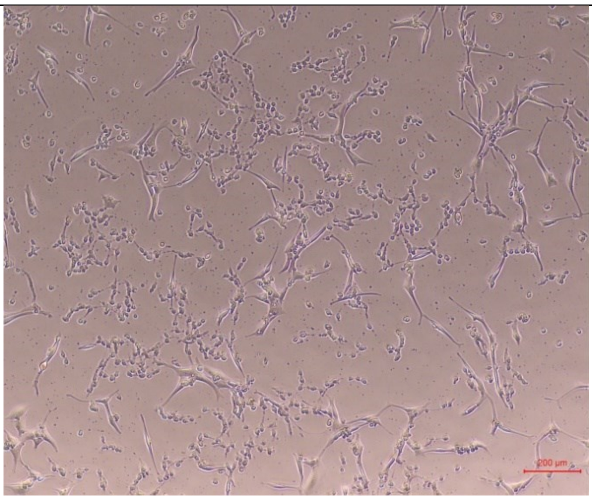<br>H1	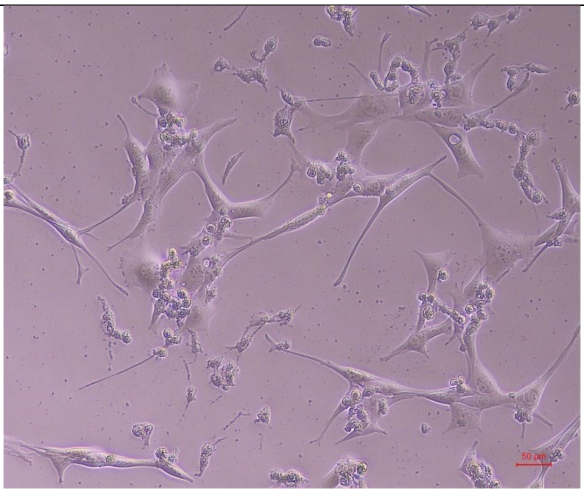<br>H2

5. [$MoO_2(HL^4)$]

Microphotographs of control HT-22 cells and the cells under the conditions of H_2O_2-induced cytotoxicity incubated with [$MoO_2(HL^4)$] for 48 hours

**Control**<br><br>A1,A2. Typical morphological structure of the control cells, which are attached, the cells shape is unchanged.	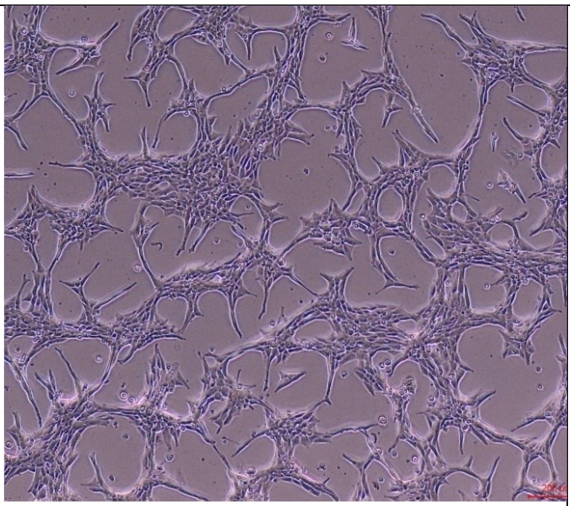<br>A1	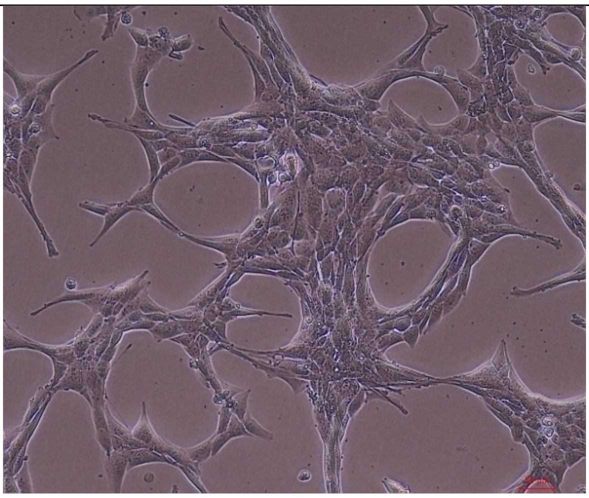<br>A2
**H_2O_2, 500 μM**<br><br>B1,B2. Significant reduction in the quantity of visible attached cells and an increase in the quantity of the damaged cells which are round-shaped, detached and floating.	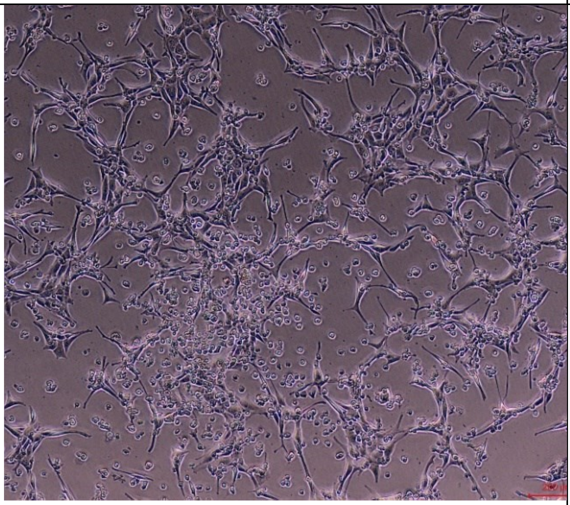<br>B1	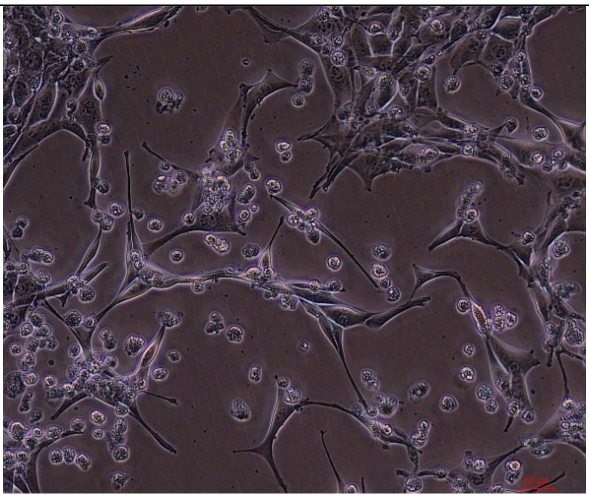<br>B2
**[$MoO_2(HL^4)$], 10 μM + H_2O_2, 500 μM**<br>C1,C2. The typical morphological structure of the cells is maintained, the cells are attached, the cells shape is unchanged, there are no visible changes in cell confluence compared with the microphotographs of the control cells.	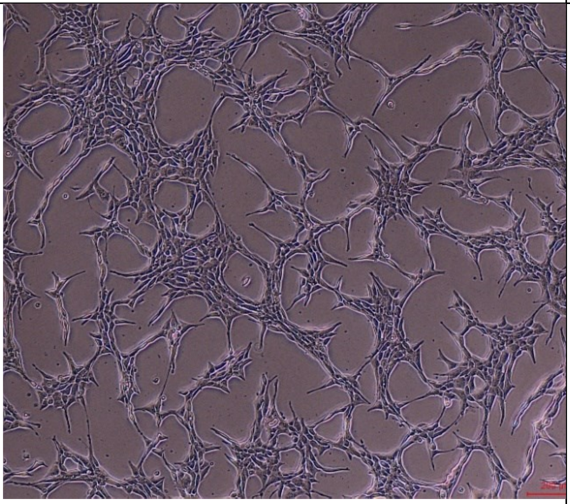<br>C1	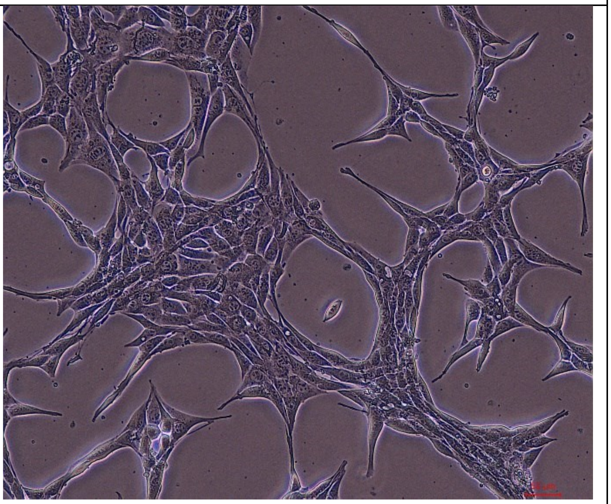<br>C2

**$[MoO_2(HL^4)]$, 25 µM + H_2O_2, 500 µM**<br><br>D1,D2. The typical morphological structure of the cells is maintained, the cells are attached, the cells shape is unchanged, there are no visible changes in cell confluence compared with the microphotographs of the control cells.	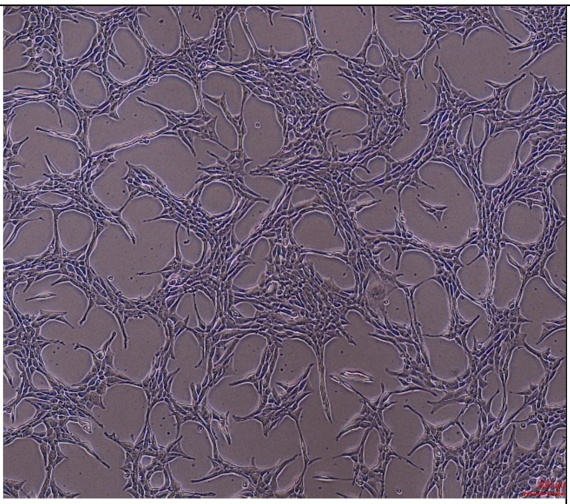<br>D1	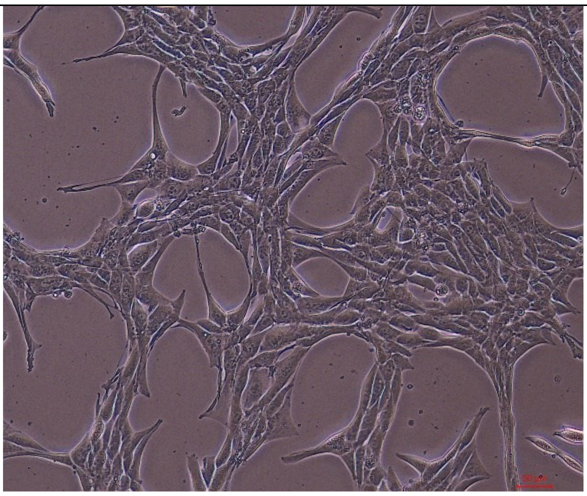<br>D2
**$[MoO_2(HL^4)][MoO_2(HL^4)]$, 50 µM + H_2O_2, 500 µM**<br><br>E1,E2,E3. Significant reduction in the quantity of visible attached cells and an abrupt increase in the quantity of severely damaged cells which are round-shaped, detached and floating.<br><br>Crystal-like structures (presumably, formed by the studied compound) are visible among the damaged cells (E3).	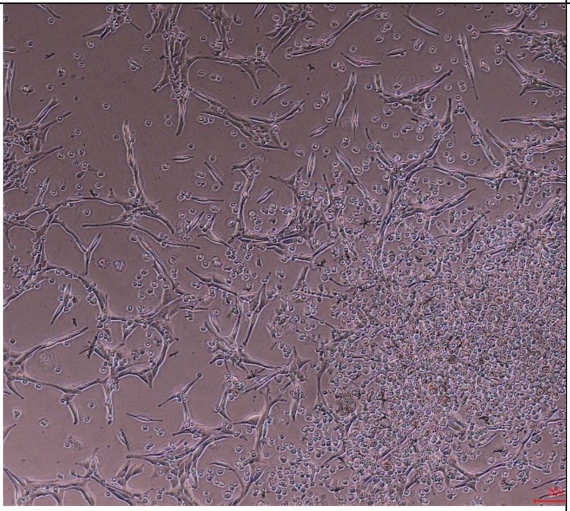<br>E1	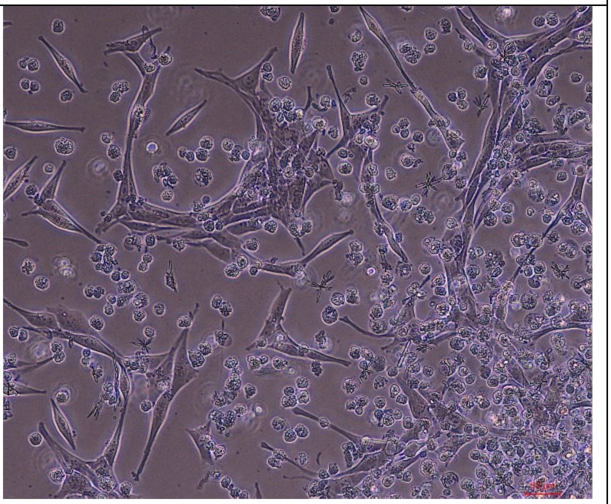<br>E2
	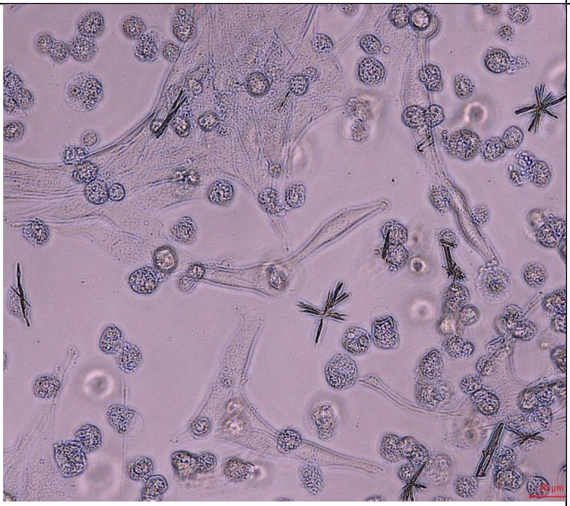<br>E3	
