# Supplementary material for: Structure-Dependent Cytotoxic and Cytoprotective Effects of Molybdenum(VI) Schiff Base Complexes in the Neuronal Cells In Vitro
Source: Molecules. 2026 Aug 18;31(16):2883. doi: 10.3390/molecules31162883 (PMC13515801; doi:10.3390/molecules31162883)
Supplement: Supplementary file 1 [file molecules-31-02883-s001.zip › SUPPLEMENTARY FILE S2.pdf]

Supplementary file 2.

The effects of [$MoO_2(HL^3)$], [$MoO_2(HL^2)$] and the reference drug cyanocobalamin on HT-22 cells viability under the conditions of H_2O_2-induced cytotoxicity (500 μM, 48 hours)

1. [$MoO_2(HL^3)$]
Microphotographs of control HT-22 cells and the cells under the conditions of H_2O_2-induced cytotoxicity incubated with [$MoO_2(HL^3)$]for 48 hours

**Control**<br><br>A1,A2. Typical morphological structure of the control cells, which are attached, the cells shape is unchanged.	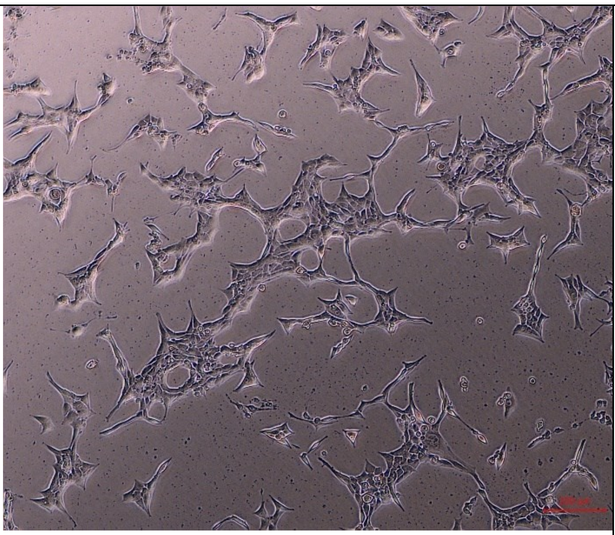<br>A1	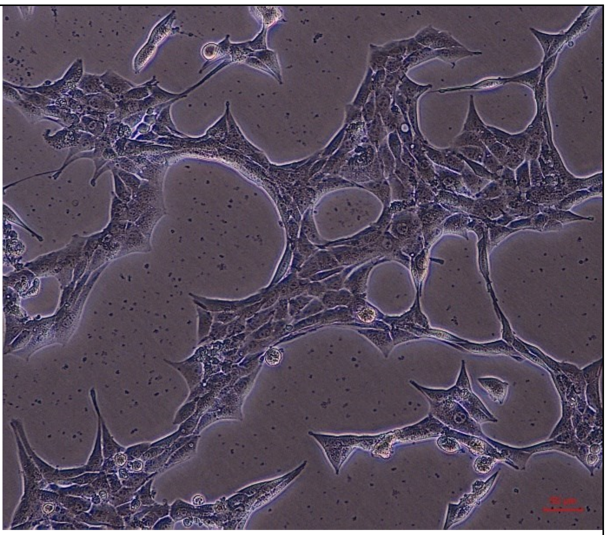<br>A2
**H_2O_2, 500 µM**<br>B1,B2. Significant reduction in the quantity of visible attached cells, change in the shape of the cells, among which there are elongated and those with the changed surface. Presence of severely damaged cells which are round-shaped, detached and floating as well as fragments of cell debris.	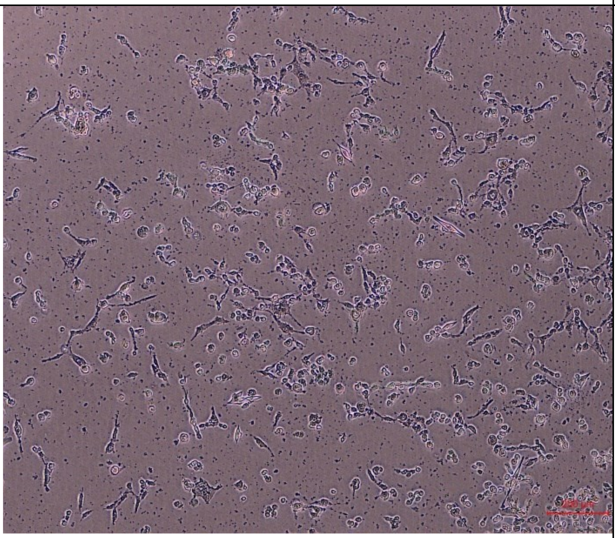<br>B1	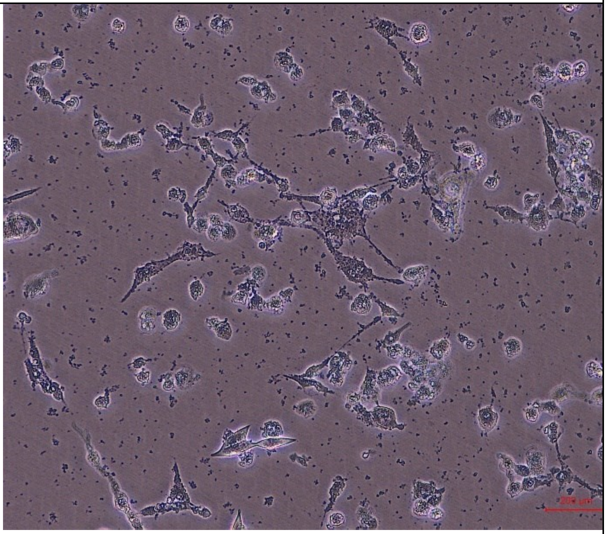<br>B2
**[$MoO_2(HL^3)$], 0.1 µM + H_2O_2, 500 µM**<br>C1,C2. Significant reduction in the quantity of visible attached cells, change in the shape of the cells, among which there are elongated and those with the changed surface. Presence of severely damaged cells which are	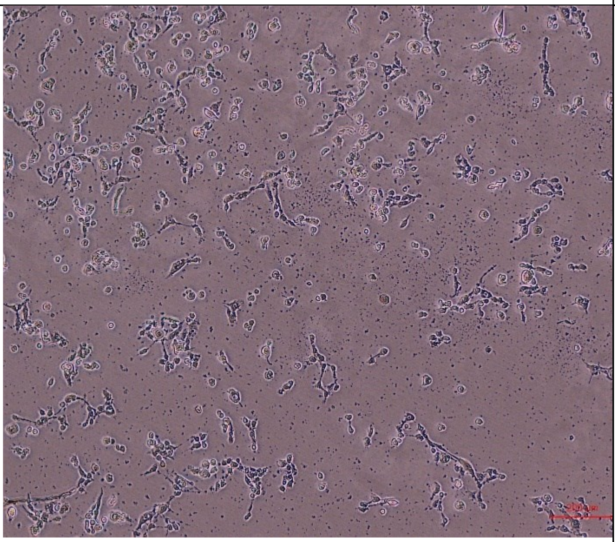<br>C1	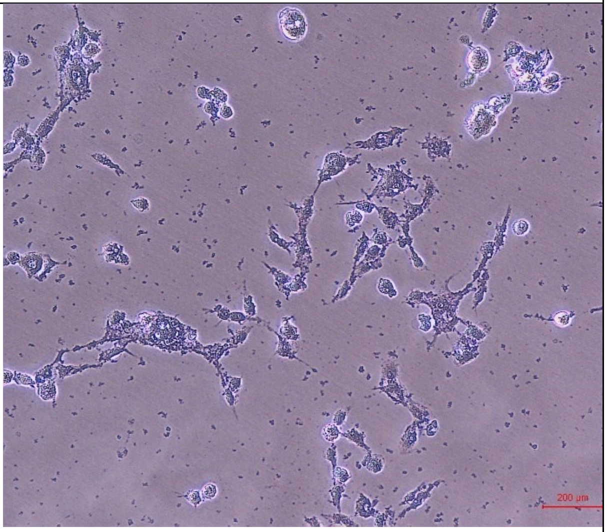<br>C2

round-shaped, detached and floating as well as fragments of cell debris.		
**[$MoO_2(HL^3)$], 0.5 µM + H_2O_2, 500 µM**<br><br>D1,D2. Significant reduction in the quantity of visible attached cells, change in the shape of the cells, among which there are elongated and those with the changed surface. Presence of severely damaged cells which are round-shaped, detached and floating as well as fragments of cell debris.	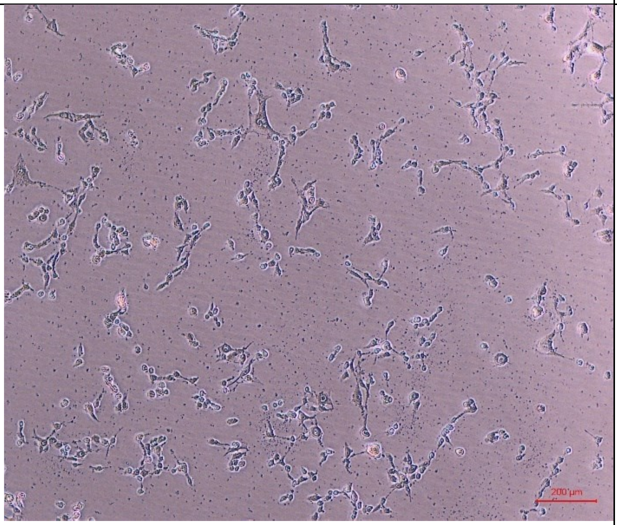<br>D1	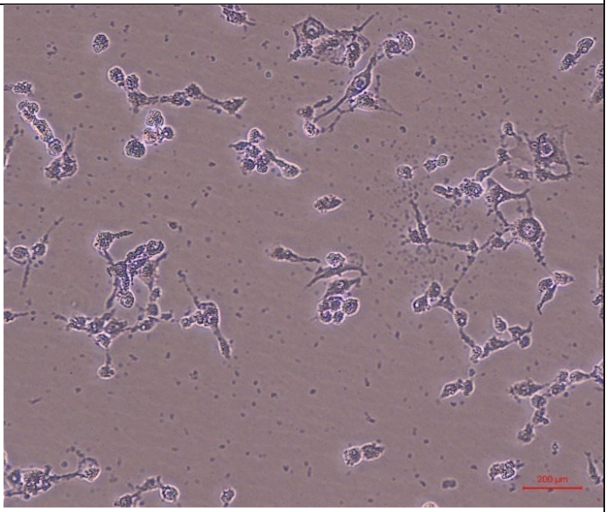<br>D2
**[$MoO_2(HL^3)$], 1.0 µM + H_2O_2, 500 µM**<br><br>E1,E2. Significant reduction in the quantity of visible attached cells, change in the shape of the cells, among which there are elongated and those with the changed surface. Presence of severely damaged cells which are round-shaped, detached and floating as well as fragments of cell debris.	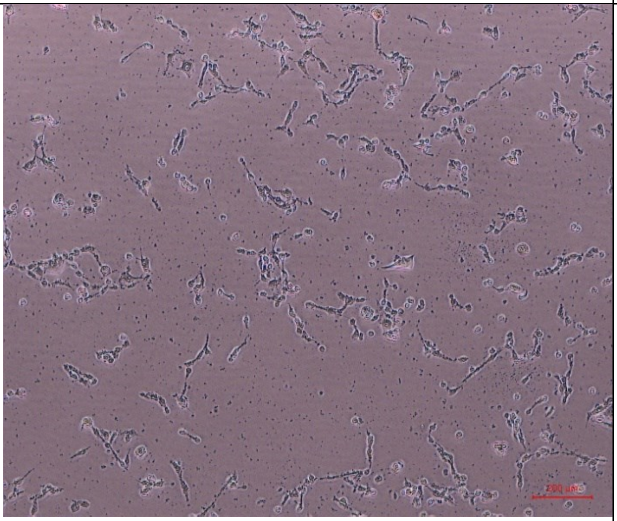<br>E1	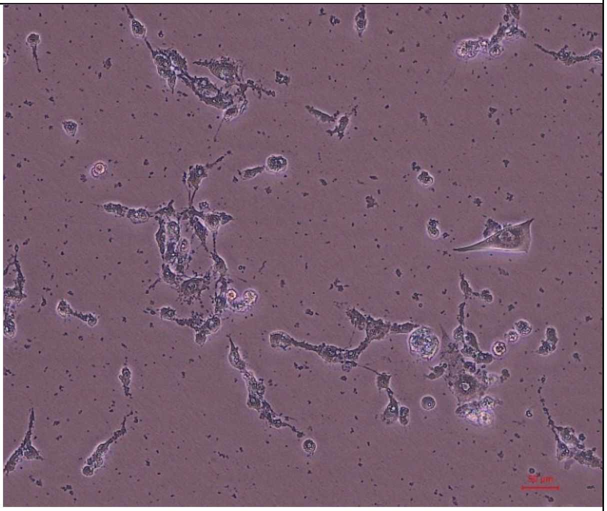<br>E2

**[$MoO_2(HL^3)$], 2.5 µM + H_2O_2, 500 µM**<br><br>F1,F2. Significant reduction in the quantity of visible attached cells, change in the shape of the cells, among which there are elongated and those with the changed surface. Presence of severely damaged cells which are round-shaped, detached and floating as well as fragments of cell debris. In contrast to the microphotographs B1,B2,C1,C2,D1,D2,E1,E2 the single cells maintain the shape close to the normal one (F2).	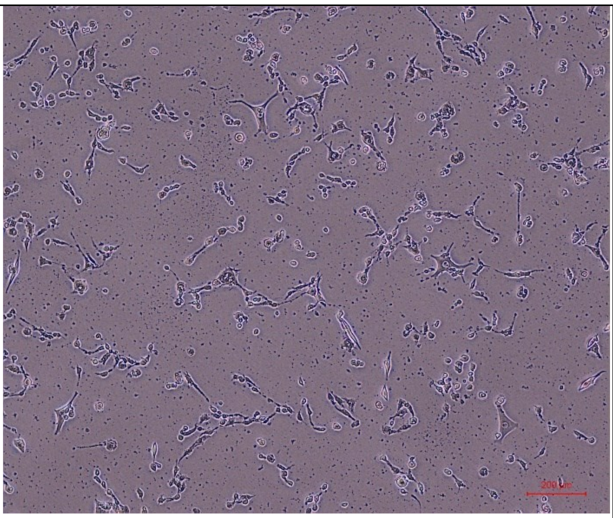<br>F1	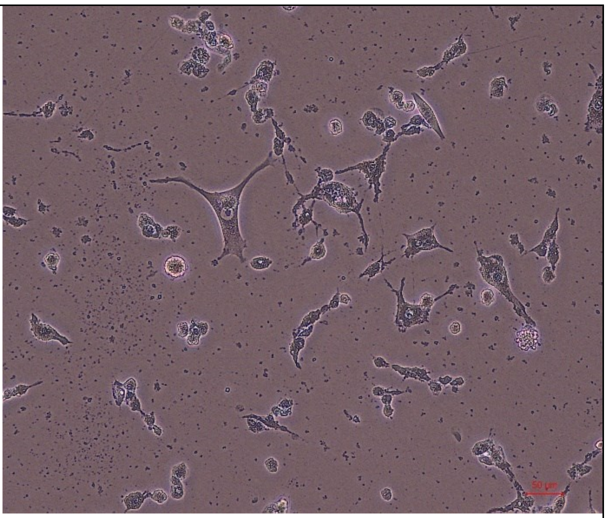<br>F2
**[$MoO_2(HL^3)$], 5 µM + H_2O_2, 500 µM**<br><br>G1,G2. Significant reduction in the quantity of visible attached cells, change in the shape of the cells, among which there are elongated and those with the changed surface. Presence of severely damaged cells which are round-shaped, detached and floating as well as fragments of cell debris. In contrast to the microphotographs B1,B2,	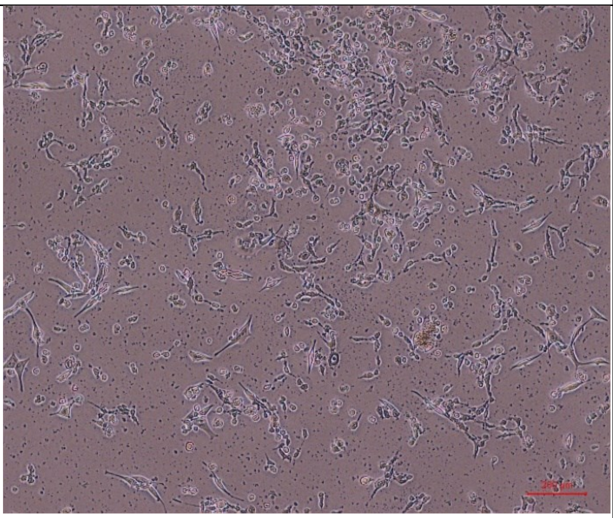<br>G1	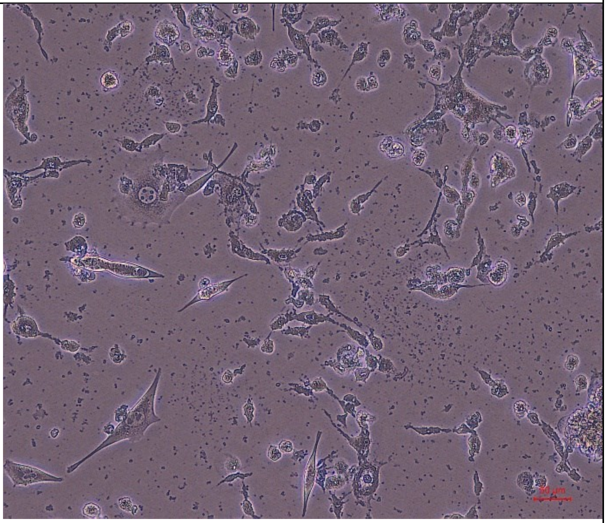<br>G2

C1,C2,D1,D2,E1,E2 certain cells maintain the shape close to the normal one (G2).		
**[$MoO_2(HL^3)$], 10 µM + H_2O_2, 500 µM**<br><br>H1,H2. The typical morphological structure of the cells is maintained, the cell confluence is decreased, change in the shape of single cells, among which there are elongated. Presence of separate severely damaged cells which are round-shaped, detached and floating. Quantity of the attached cells is significantly increased compared with the microphotographs B1,B2,C1,C2,D1,D2,E1,E2.	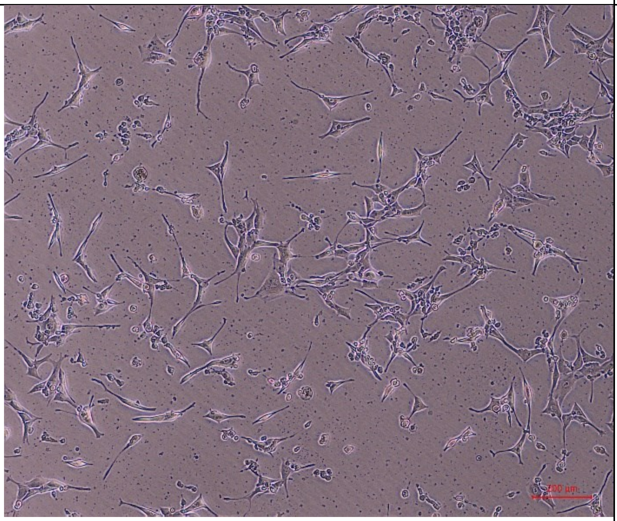<br>H1	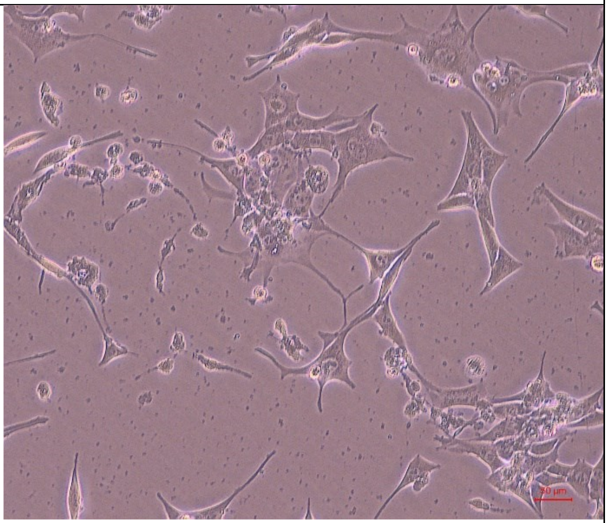<br>H2
**[$MoO_2(HL^3)$], 25 µM + H_2O_2, 500 µM**<br><br>I1,I2. The typical morphological structure of the cells is maintained; the cell confluence is moderately decreased. Only single damaged cells, which are round-shaped, are present. These microphotographs demonstrate the most significant differences with the control cells injured with hydrogen peroxide (B1,B2).	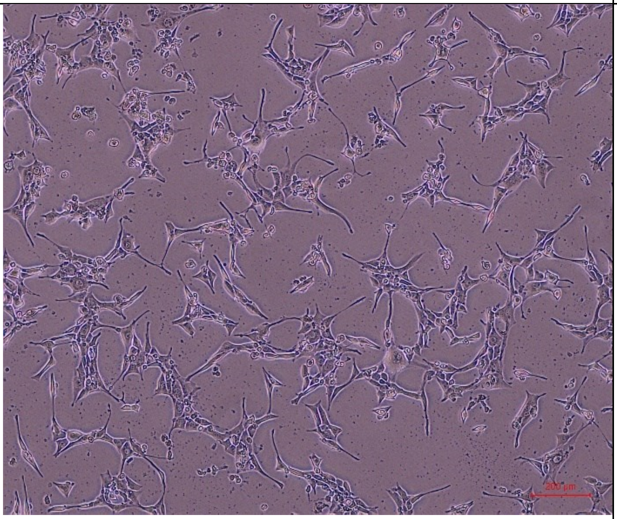<br>I1	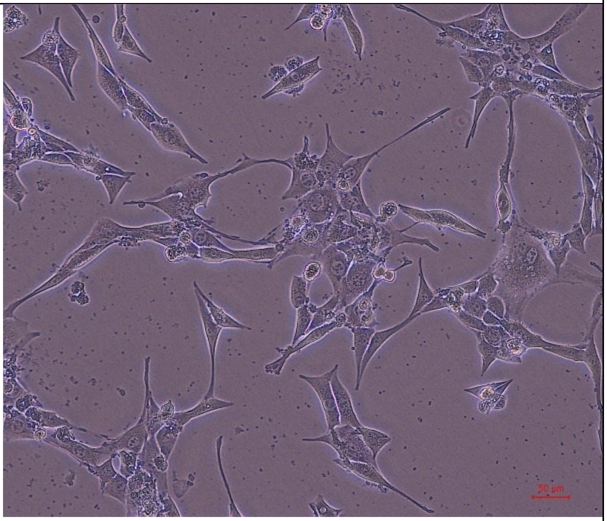<br>I2

**[$MoO_2(HL^3)$], 50 µM + H_2O_2, 500 µM**<br>J1,J2. The typical morphological structure of the cells is maintained, the cell confluence is decreased, change in the shape of single cells, among which there are elongated. Presence of separate severely damaged cells which are round-shaped, detached and floating. Quantity of the attached cells is significantly increased compared with the microphotographs B1,B2,C1,C2,D1,D2,E1,E2.	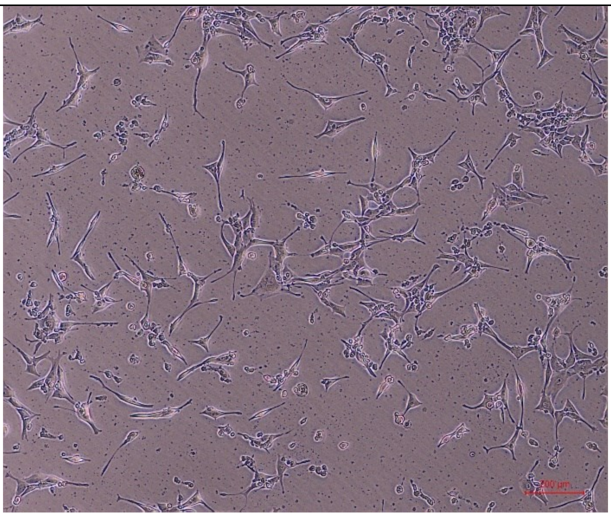<br>J1	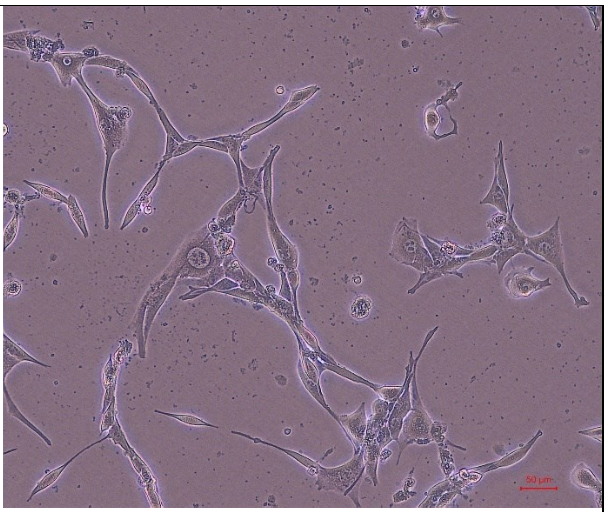<br>J1
**[$MoO_2(HL^3)$], 100 µM + H_2O_2, 500 µM**<br><br>K1,K2. The typical morphological structure of the cells is maintained, the cell confluence is significantly decreased, the shape of the cells is changed, there are elongated cells. The damaged, round-shaped cells are present in high quantities, as well as the fragments of cell debris.	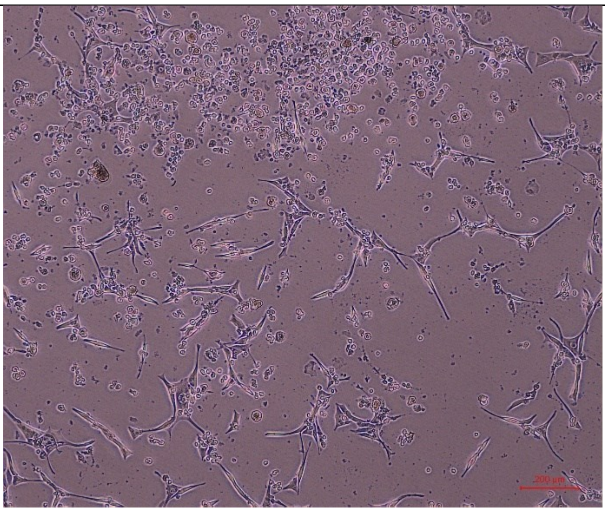<br>K1	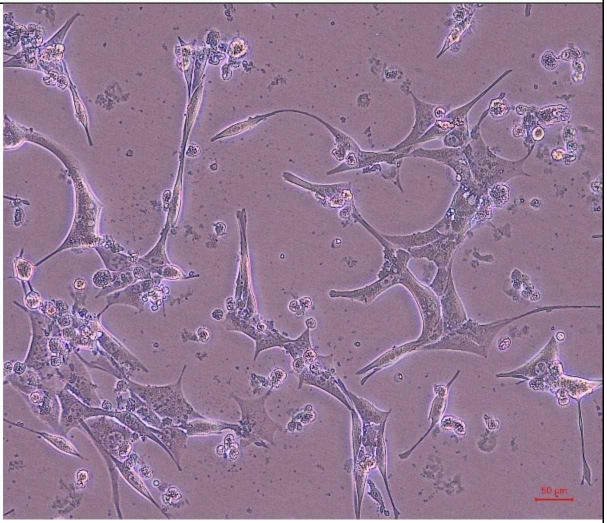<br>K2

2. [$MoO_2(HL^2)$]

Microphotographs of control HT-22 cells and the cells under the conditions of H_2O_2-induced cytotoxicity incubated with [$MoO_2(HL^2)$] for 48 hours

**Control**<br><br>A1,A2. Typical morphological structure of the control cells, which are attached, the cells shape is unchanged.	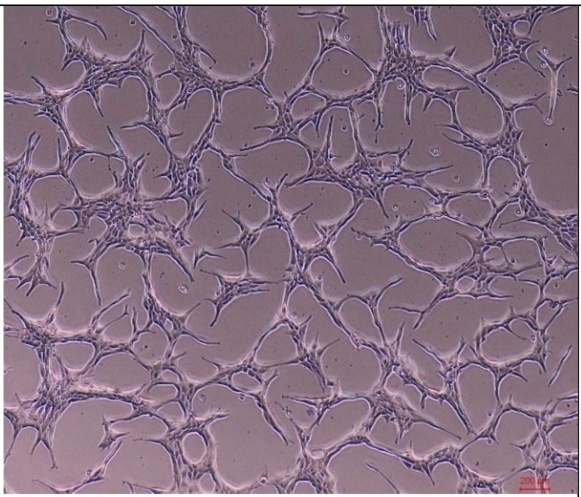<br>A1	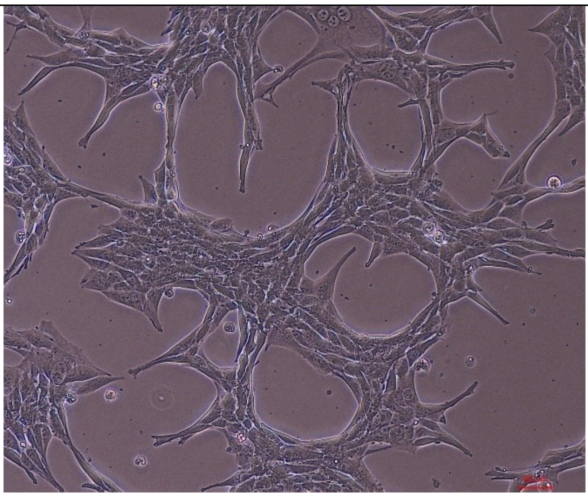<br>A2
**H_2O_2, 500 µM**<br><br>B1,B2. The typical morphological structure of the cells is maintained, but the cell confluence is decreased, change in the shape of the cells, among which there are elongated. The damaged, round-shaped cells are present in significant quantities, as well as the fragments of cell debris.	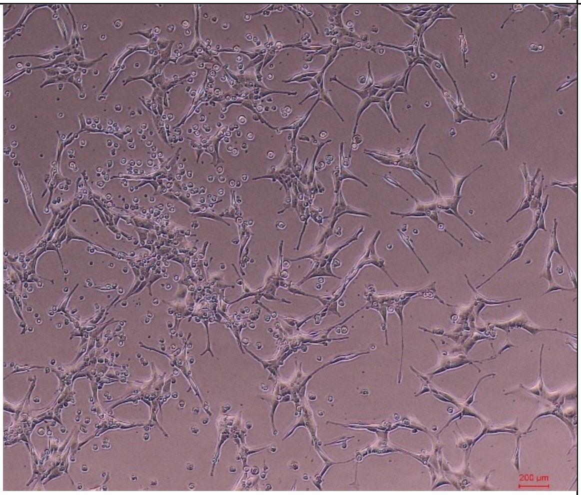<br>B1	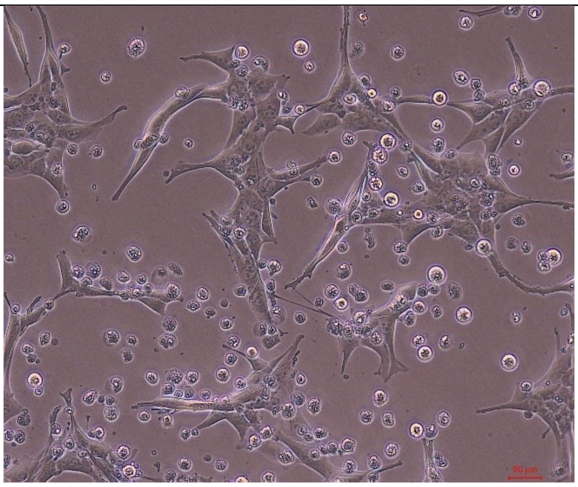<br>B2
**[$MoO_2(HL^2)$], 1.0 µM + H_2O_2, 500 µM**<br><br>C1,C2. The typical morphological structure of the cells is maintained, but the cell confluence is decreased, change in the shape of the cells, among which there are elongated. The damaged, round-shaped cells are present in significant quantities, as well as the fragments of cell debris	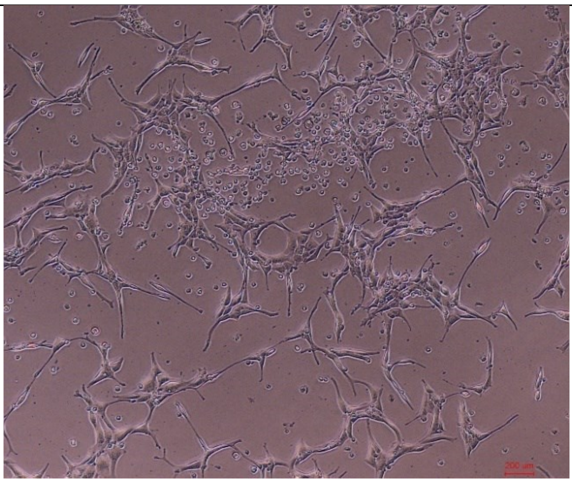<br>C1	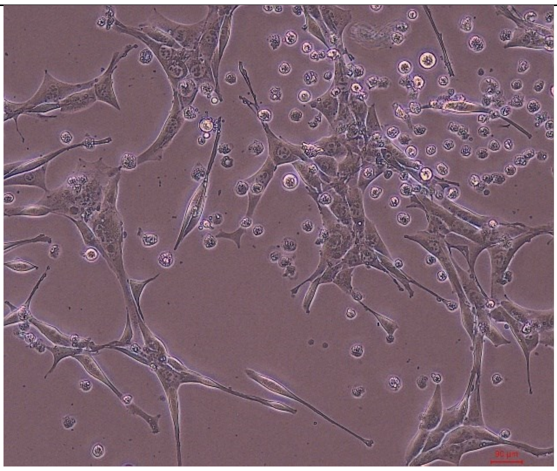<br>C2

**$[MoO_2(HL^2)]$,**<br>**5 µM**<br>**+ H_2O_2, 500 µM**<br><br>D1,D2. The typical morphological structure of the cells is maintained, but the cell confluence is decreased, change in the shape of the cells, among which there are elongated. The damaged, round-shaped cells are present in significant quantities, as well as the fragments of cell debris	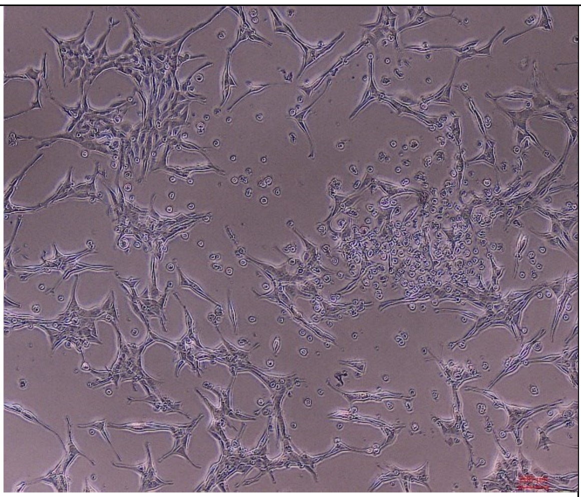<br>D1	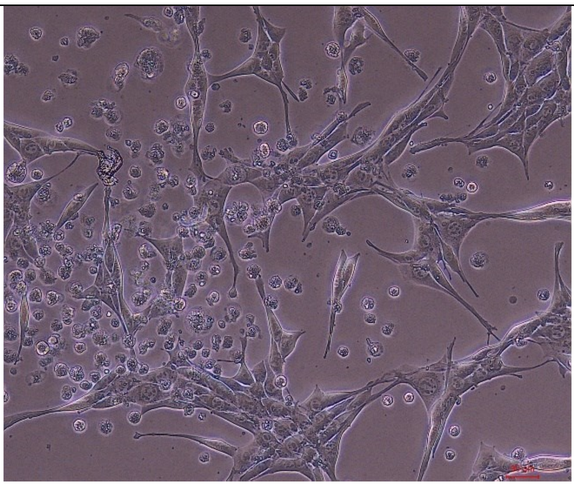<br>D2
**$[MoO_2(HL^2)]$,**<br>**10 µM**<br>**+ H_2O_2, 500 µM**<br><br>E1,E2. The typical morphological structure of the cells is maintained. Moderate decrease of cell confluence, change in the shape of the cells, among which there are elongated. The damaged, round-shaped cells are visible at certain microphotographs, but their quantity is significantly reduced compared with the microphotographs B1,B2 (untreated cells injured with hydrogen peroxide).	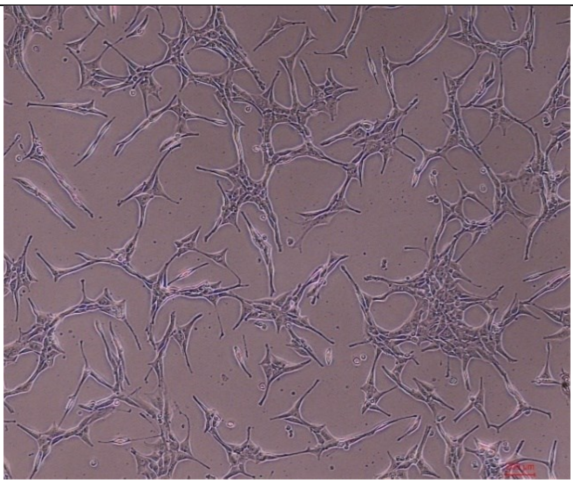<br>E1	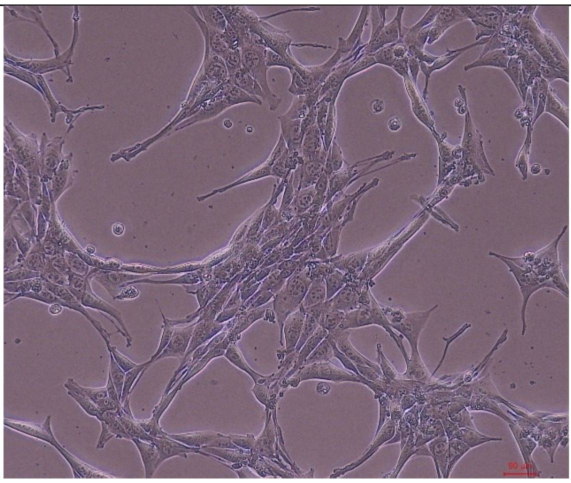<br>E2

**[$MoO_2(HL^2)$],<br>25 µM<br>+ H_2O_2, 500 µM**<br><br>F1,F2. The typical morphological structure of the cells is maintained. Decrease in cell confluence, change in the shape of the cells, among which there are elongated. Only sporadic damaged, round-shaped cells are visible.	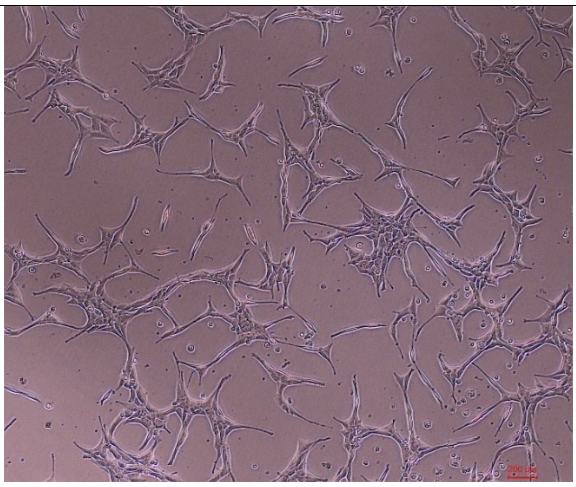<br>F1	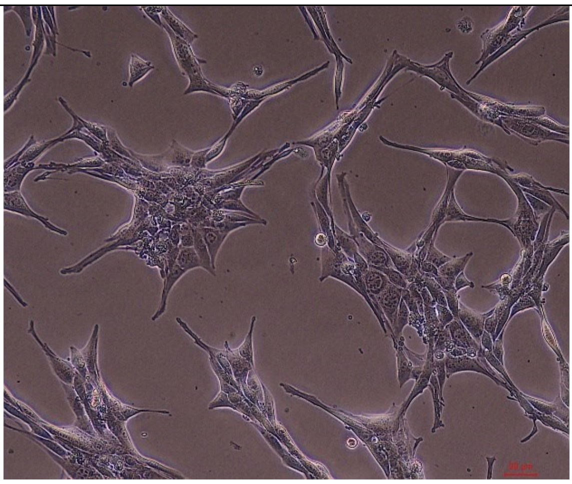<br>F2
**[$MoO_2(HL^2)$],<br>50 µM<br>+ H_2O_2, 500 µM**<br><br>G1,G2. The typical morphological structure of the cells is maintained. Decrease in cell confluence, change in the shape of the cells, among which there are elongated. Only sporadic damaged, round-shaped cells are visible.	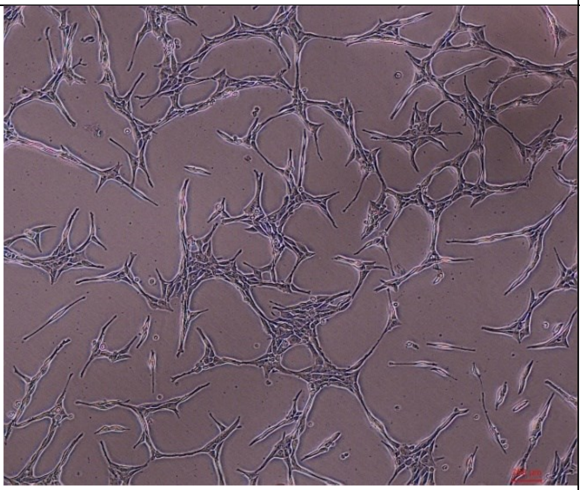<br>G1	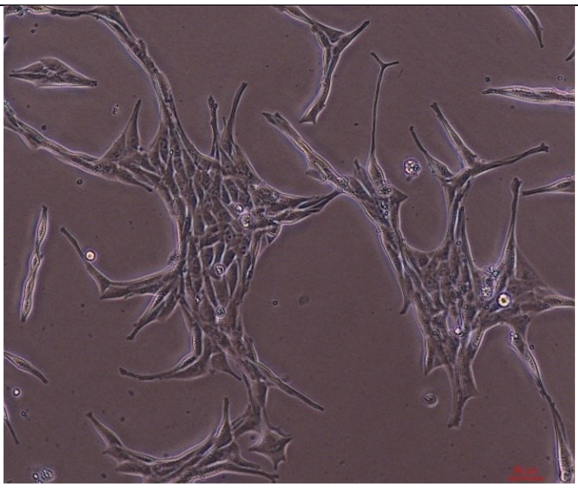<br>G2
**[$MoO_2(HL^2)$],<br>100 µM<br>+ H_2O_2, 500 µM**<br><br>H1,H2. Significant decrease in cell confluence, change in the shape of the cells, among which there are elongated. The damaged, round-shaped cells are present in significant quantities, as well as the fragments of cell debris.	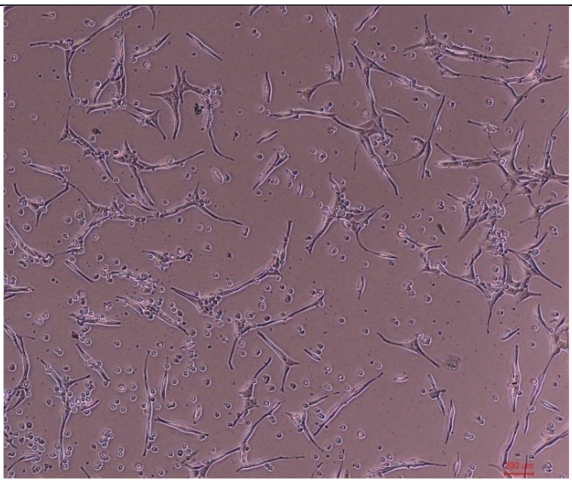<br>H1	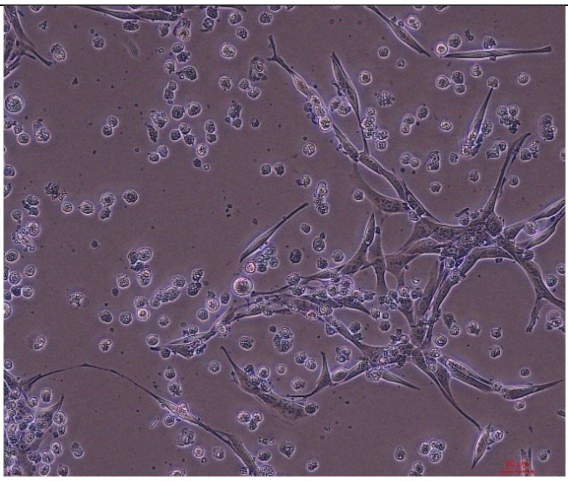<br>H2

3.1. Microphotographs of control HT-22 cells and the cells under the conditions of H_2O_2-induced cytotoxicity incubated with cyanocobalamin (1.0 µM; 5 µM; 10 µM; 25 µM) for 48 hours.

Since not all concentrations were accessed at once, the correspondent representative control photos are given in each dataset.

**Control**<br><br>A1,A2. Typical morphological structure of the control cells, which are attached, the cells shape is unchanged.	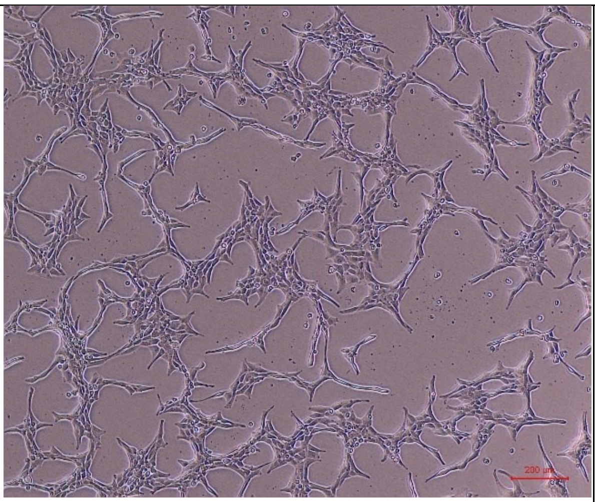<br>A1	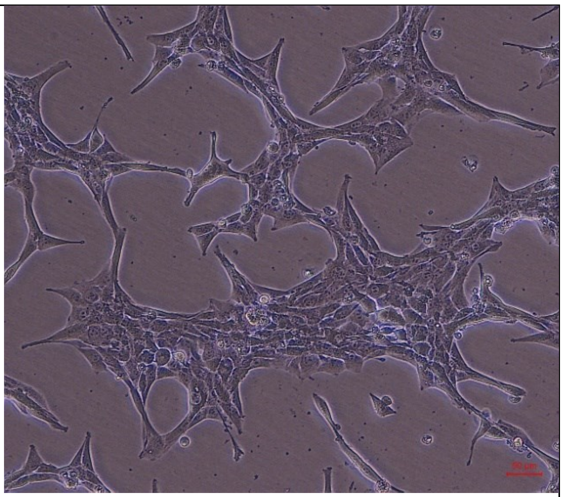<br>A2
**H_2O_2, 500 µM**<br><br>B1,B2. The cell confluence is considerably decreased, change in the shape of the cells, among which there are elongated. The damaged, round-shaped cells are present in significant quantities, as well as sporadic fragments of cell debris.	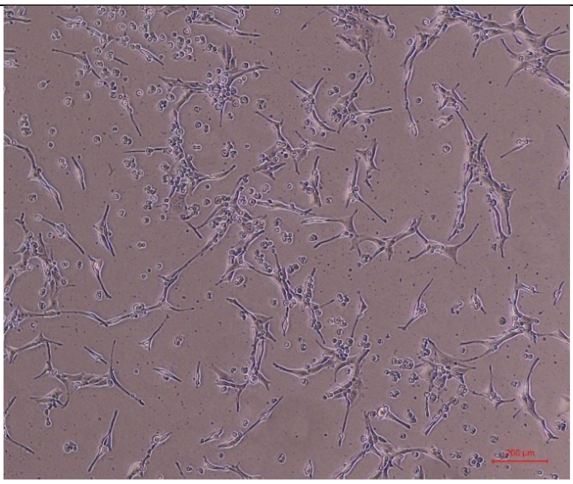<br>B1	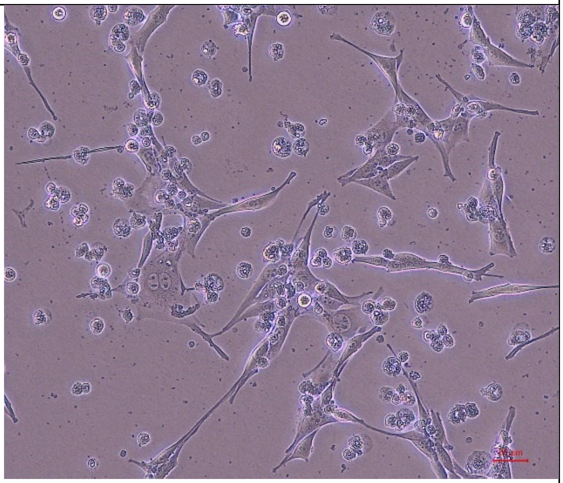<br>B2
**Cyanocobalamin, 1.0 µM + H_2O_2, 500 µM**<br><br>C1,C2. The cell confluence is considerably decreased, change in the shape of the cells, among which there are elongated. The damaged, round-shaped cells are present in significant quantities, as well as sporadic fragments of cell debris.	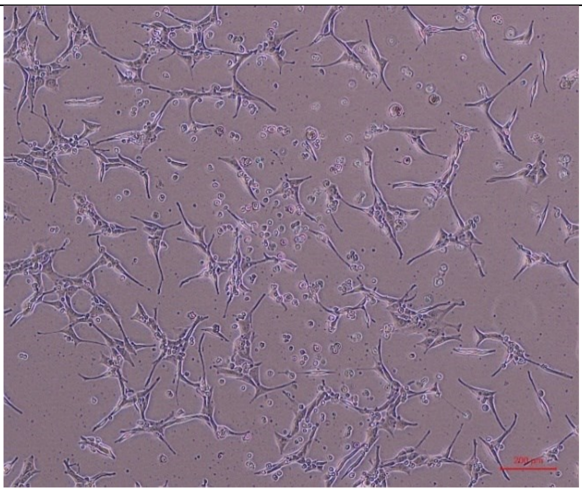<br>C1	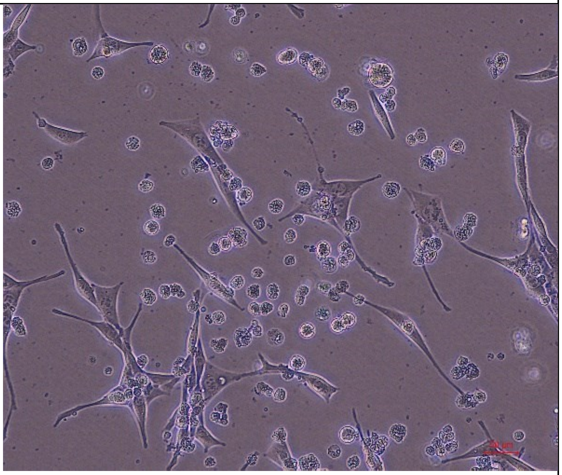<br>C2

**Cyanocobalamin, 5 µM + H_2O_2, 500 µM**<br><br>D1,D2. The cell confluence is considerably decreased, change in the shape of the cells, among which there are elongated. The damaged, round-shaped cells are present in significant quantities, as well as sporadic fragments of cell debris.	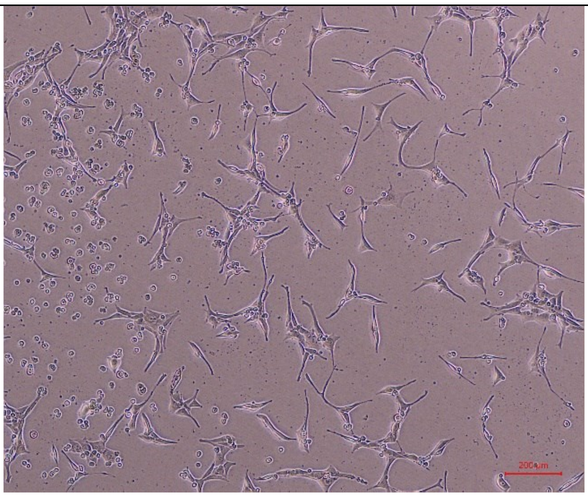<br>D1	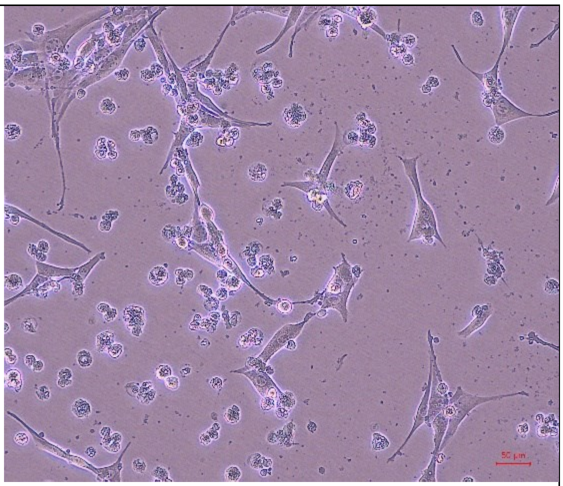<br>D2
**Cyanocobalamin, 10 µM + H_2O_2, 500 µM**<br>E1,E2. The cell confluence is considerably decreased, change in the shape of the cells, among which there are elongated. The damaged, round-shaped cells are present in significant quantities, as well as sporadic fragments of cell debris.	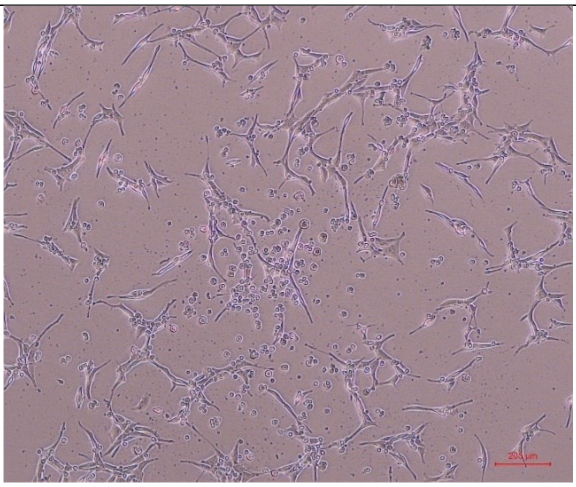<br>E1	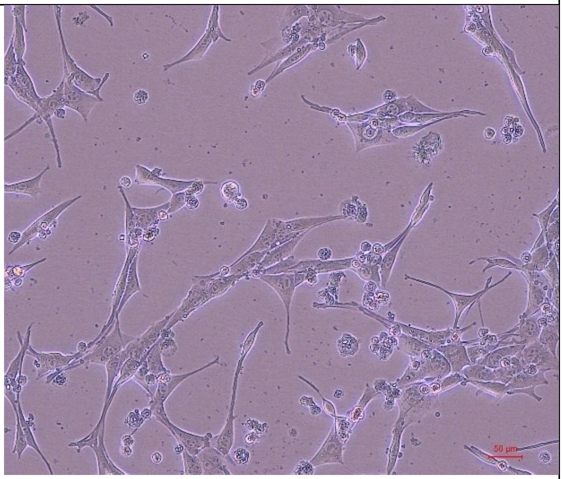<br>E2
**Cyanocobalamin, 25 µM + H_2O_2, 500 µM**<br><br>F1,F2. The cell confluence is considerably decreased, change in the shape of the cells, among which there are elongated. The damaged, round-shaped cells are present in significant quantities, as well as sporadic fragments of cell debris.	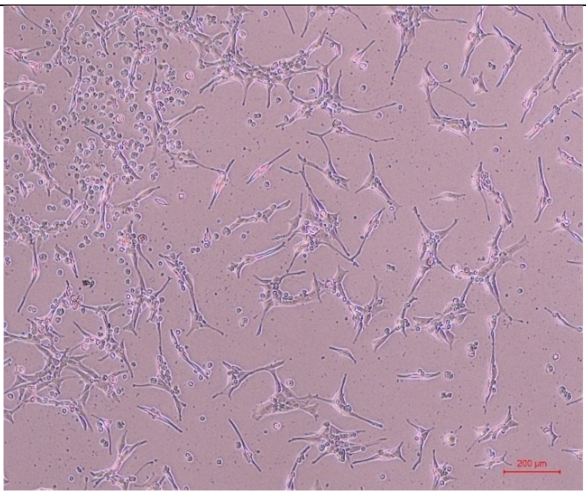<br>F1	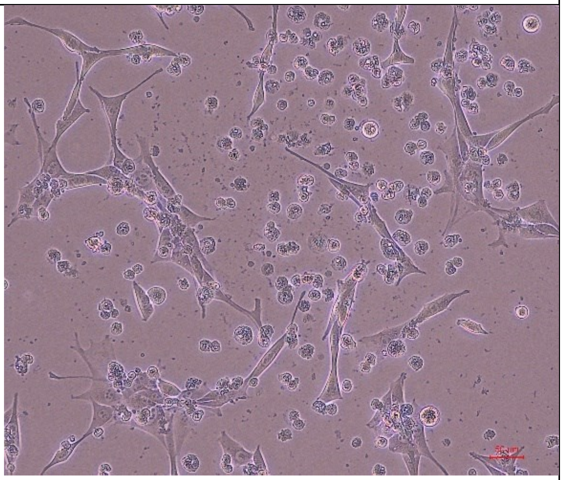<br>F2

3.2. Microphotographs of control HT-22 cells and the cells under the conditions of H_2O_2-induced cytotoxicity incubated with cyanocobalamin (50 µM) for 48 hours.

Since not all concentrations were accessed at once, the correspondent representative control photos are given in each dataset.

**H_2O_2,<br>500 µM**<br><br>B1,B2. The cell confluence is considerably decreased, change in the shape of the cells, among which there are elongated. The damaged, round-shaped cells are present in significant quantities, as well as sporadic fragments of cell debris.	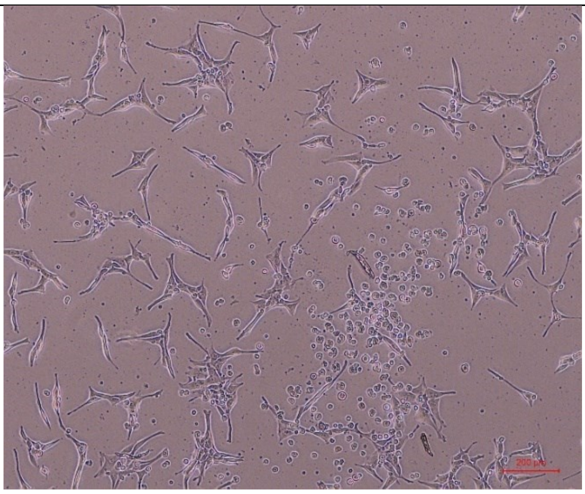<br>A1	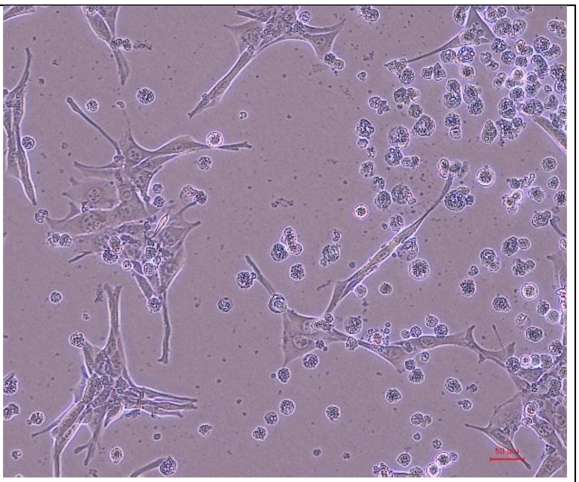<br>A2
**Cyanocobalamin, 50 µM<br>+ H_2O_2, 500 µM**<br><br>C1,C2. The cell confluence is considerably decreased, change in the shape of the cells, among which there are elongated. The damaged, round-shaped cells are present in significant quantities, as well as sporadic fragments of cell debris.	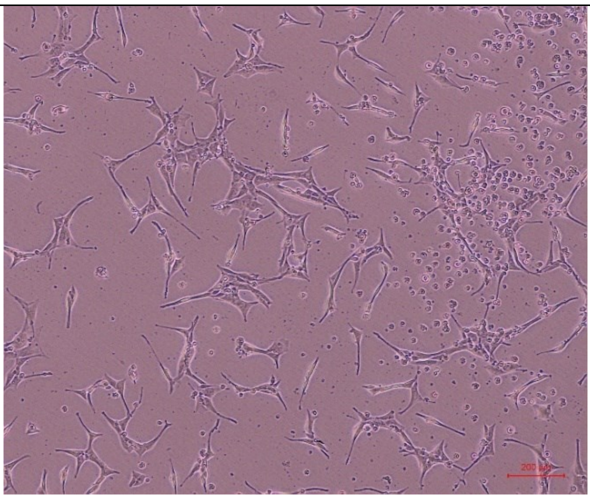<br>B1	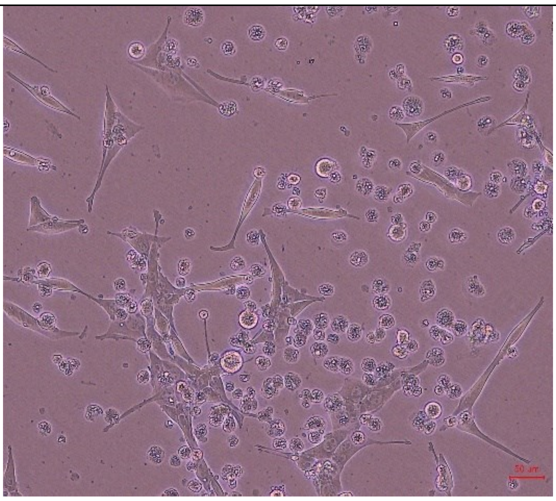<br>B2

3.3. Microphotographs of control HT-22 cells and the cells under the conditions of H_2O_2-induced cytotoxicity incubated with cyanocobalamin (100 µM) for 48 hours.

**H_2O_2, 500 µM**<br><br>A1,A2. The cell confluence is considerably decreased, change in the shape of the cells, among which there are elongated. The damaged, round-shaped cells are present in significant quantities, as well as sporadic fragments of cell debris.	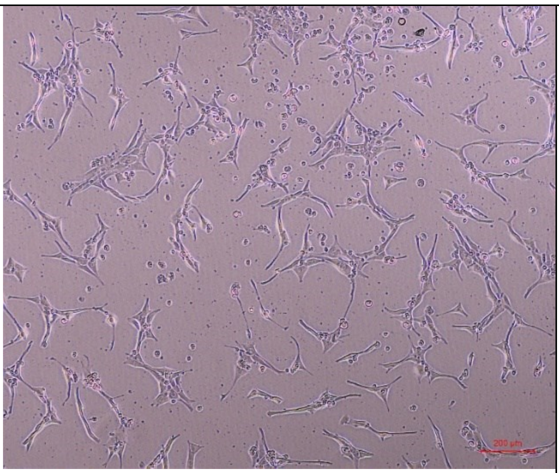<br>A1	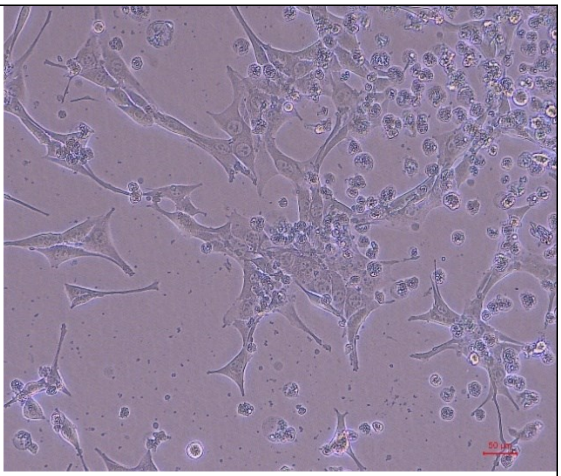<br>A2
**Cyanocobalamin, 100 µM + H_2O_2, 500 µM**<br><br>B1,B2. The cell confluence is considerably decreased, change in the shape of the cells, among which there are elongated. The damaged, round-shaped cells are present in significant quantities, as well as sporadic fragments of cell debris.	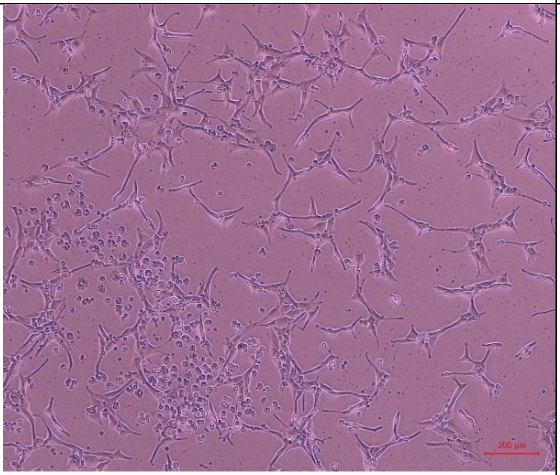<br>B1	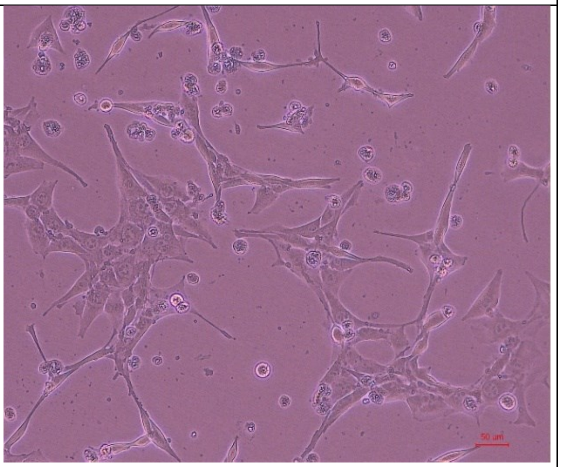<br>B2$_1$

Since not all concentrations were accessed at once, the correspondent representative control photos are given in each dataset.
