# Supplementary material for: Structure-Dependent Cytotoxic and Cytoprotective Effects of Molybdenum(VI) Schiff Base Complexes in the Neuronal Cells In Vitro
Source: Molecules. 2026 Aug 18;31(16):2883. doi: 10.3390/molecules31162883 (PMC13515801; doi:10.3390/molecules31162883)
Supplement: Supplementary file 1 [file molecules-31-02883-s001.zip › SUPPLEMENTARY FILE S3.pdf]

Supplementary file 3.

The effects of [(+)$MoO_2(HL^5)$], [(+)$MoO_2(HL^1)(CH_3OH)$], [$MoO_2(HL^5)$], [$MoO_2(HL^6)$], [$MoO_2(HL^4)$] *per se* on HT-22 cells viability (48 hours)

3.1.1. [$MoO_2(HL^6)$]

Microphotographs of control HT-22 cells and the cells incubated with [$MoO_2(HL^6)$] (25 µM and 50 µM) for 48 hours

**Control**<br><br>A1,A2. Typical morphological structure of the control cells, which are attached, the cells shape is unchanged.	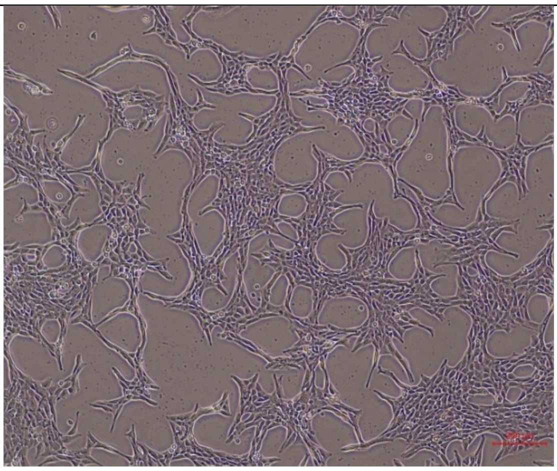<br>A1	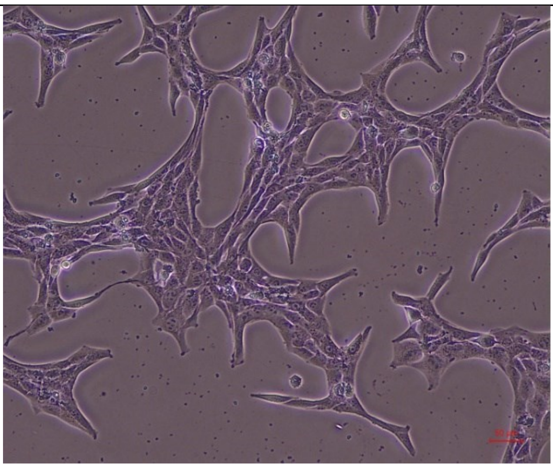<br>A2
**[$MoO_2(HL^6)$], 25 µM**<br><br>B1,B2. The typical morphological structure of the cells is maintained, the cells are attached, the cells shape is unchanged, cell confluence is slightly decreased compared with the microphotographs of the control cells.	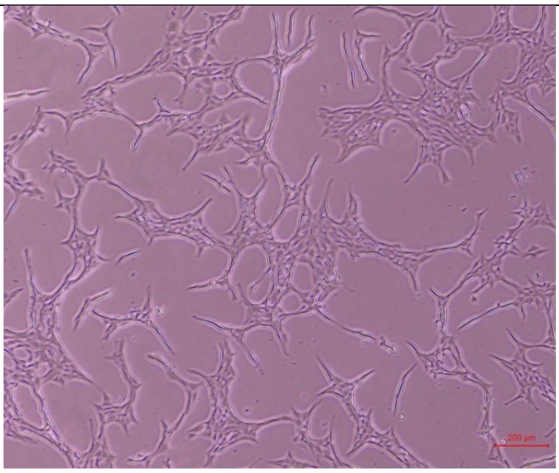<br>B1	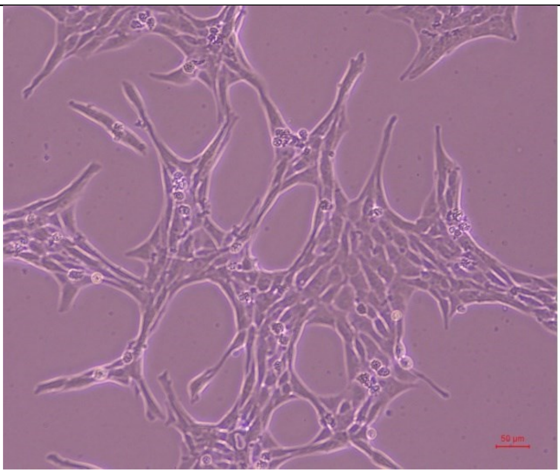<br>B2
**[$MoO_2(HL^6)$], 50 µM**<br><br>C1,C2. The typical morphological structure of the cells is maintained, the cells are attached, the cells shape is unchanged, cell confluence is slightly decreased compared with the microphotographs of the control cells.	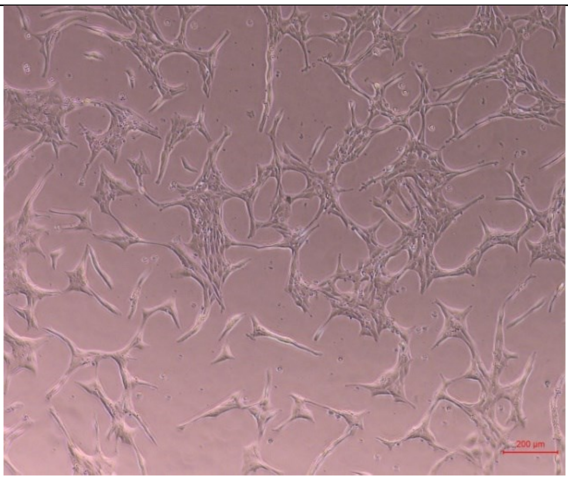<br>C1	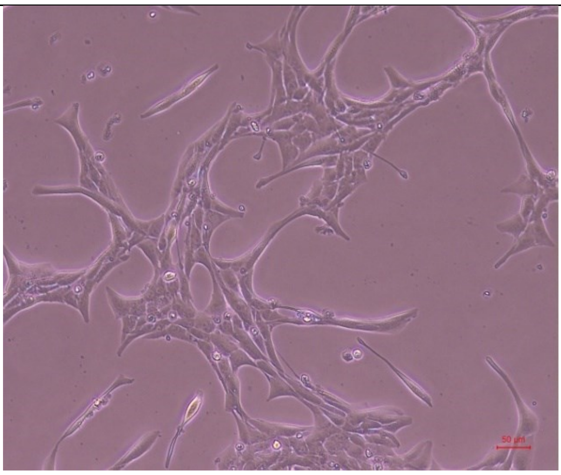<br>C2

3.1.2. $[MoO_2(HL^6)]$

Microphotographs of control HT-22 cells and the cells incubated with $[MoO_2(HL^6)]$ (100 μM) for 48 hours

**Control**<br><br>D1,D2. Typical morphological structure of the control cells, which are attached, the cells shape is unchanged.	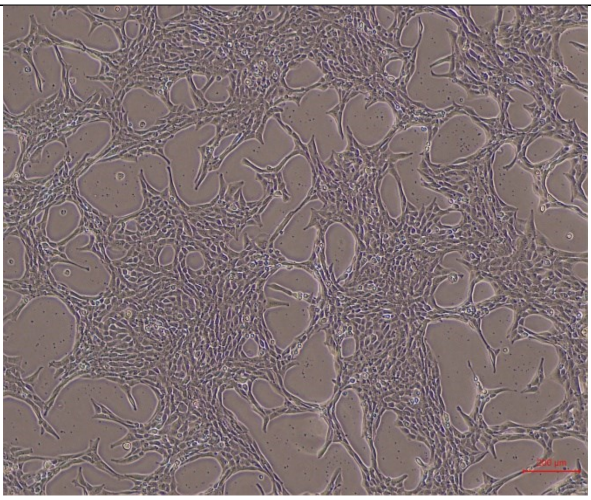<br>D1	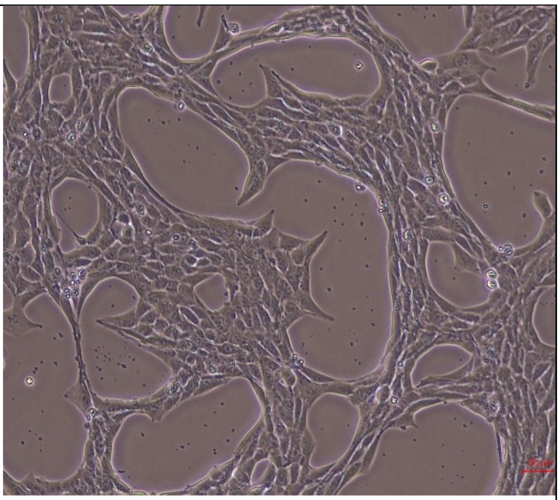<br>D2
**$[MoO_2(HL^6)]$, 100 μM**<br><br>E1,E2. The typical morphological structure of the cells is maintained, the cells are attached, there are no significant visible changes in cell confluence compared with the microphotographs of the control cells (D1,D2). Moderate increase in the quantity of damaged cells which are round-shaped, detached and floating.	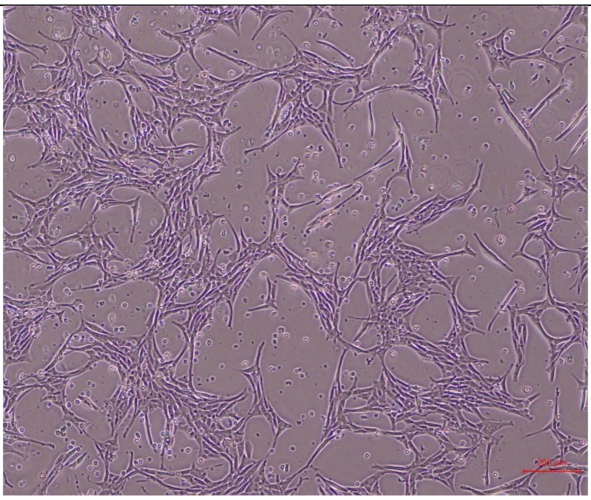<br>E1	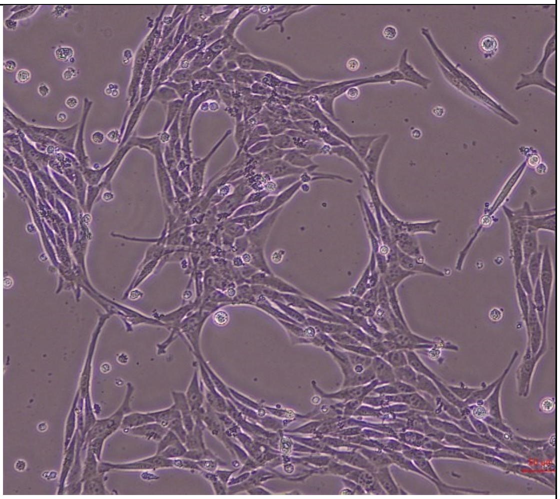<br>E2

3.1.3. [$MoO_2(HL^6)$]

Microphotographs of control HT-22 cells and the cells incubated with [$MoO_2(HL^6)$] (200 µM) for 48 hours

**Control**<br><br>F1,F2. Typical morphological structure of the control cells, which are attached, the cells shape is unchanged.	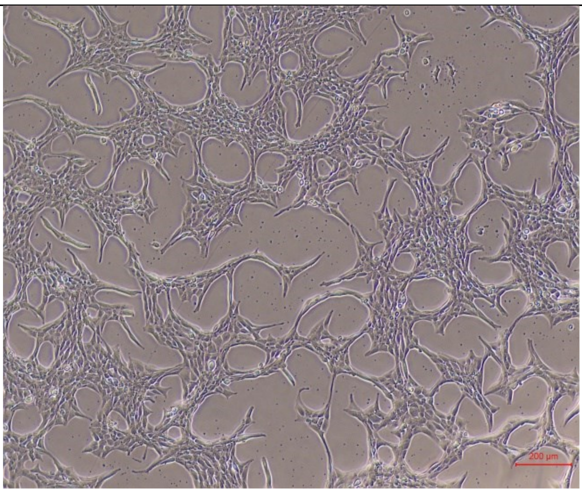 F1	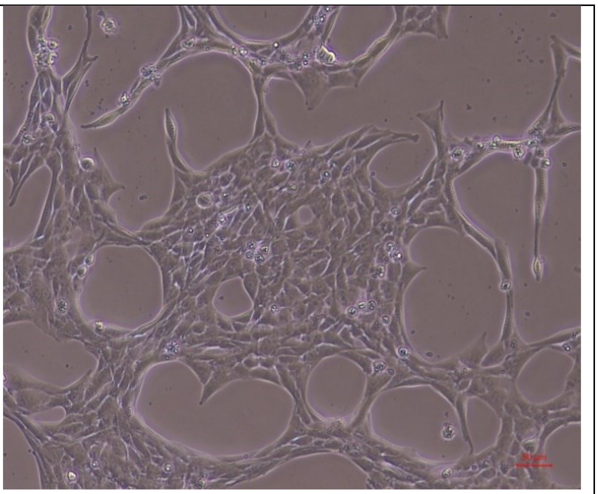 F2
**[$MoO_2(HL^6)$], 200 µM**<br><br>G1,G2. The typical morphological structure of the cells is maintained, the cells are attached, the cells shape is unchanged, cell confluence is slightly decreAsed compared with the microphotographs of the control cells (F1,F2).	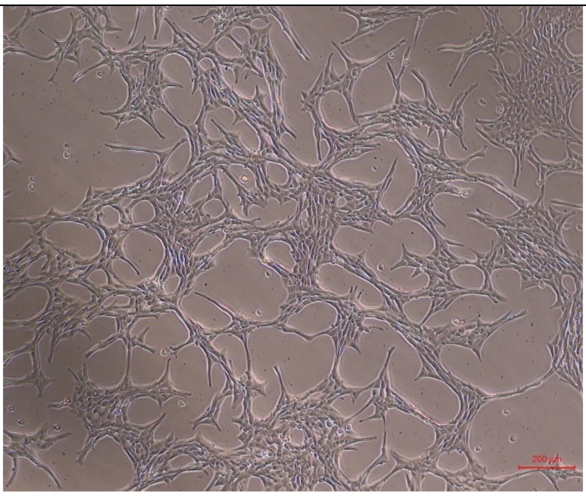 G1	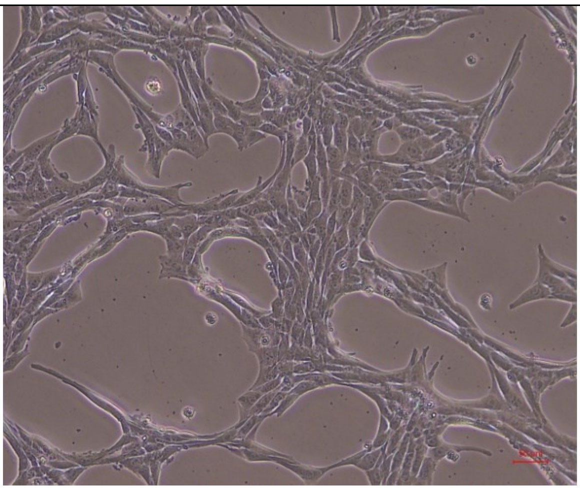 G2

Since not all concentrations were accessed at once, the correspondent representative control photos are given in each dataset.

3.2. $[MoO_2(HL^5)]$
Microphotographs of control HT-22 cells and the cells incubated with $[MoO_2(HL^5)]$ for 48 hours

**Control**<br><br>A1,A2. Typical morphological structure of the control cells, which are attached, the cells shape is unchanged.	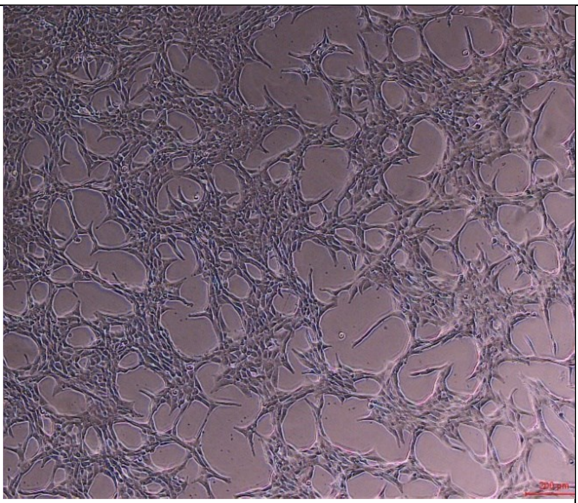<br>A1	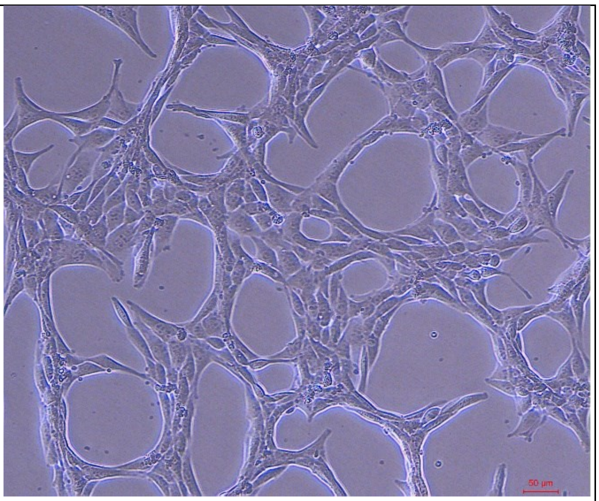<br>A2
**$[MoO_2(HL^5)]$, 10 µM**<br><br>B1,B2. The typical morphological structure of the cells is maintained, the cells are attached, the cells shape is unchanged, there are no visible changes in cell confluence compared with the microphotographs of the control cells (A1,A2).	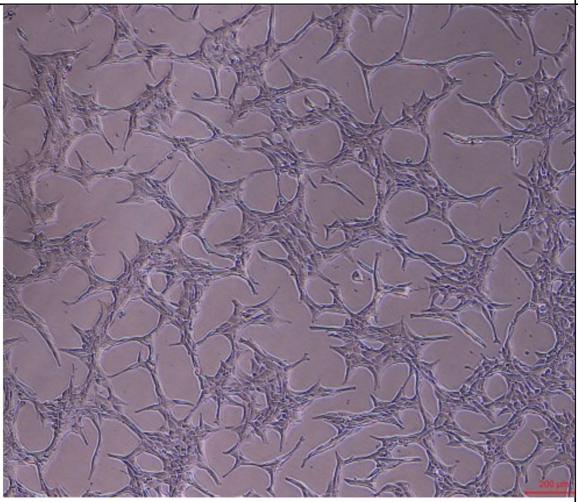<br>B1	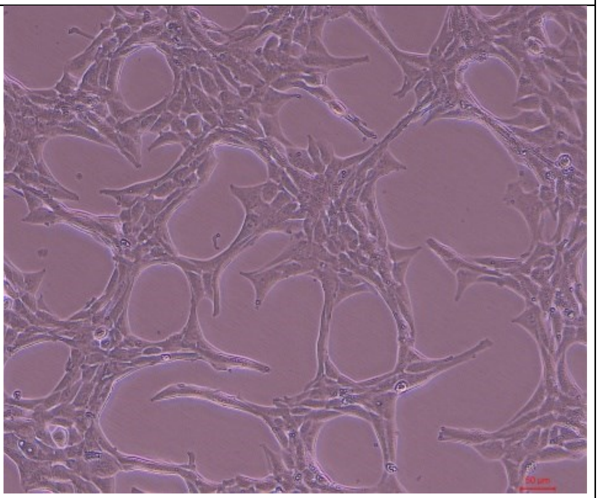<br>B2
**$[MoO_2(HL^5)]$, 25 µM**<br>C1,C2. The typical morphological structure of the cells is maintained, the cells are attached, the cells shape is unchanged, there are no visible changes in cell confluence compared with the microphotographs of the control cells (A1,A2).	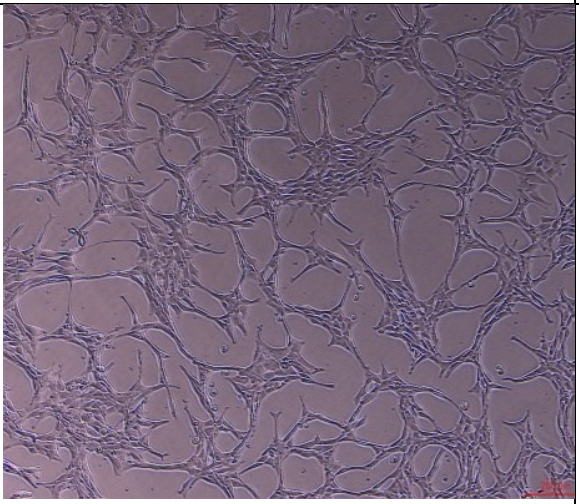<br>C1	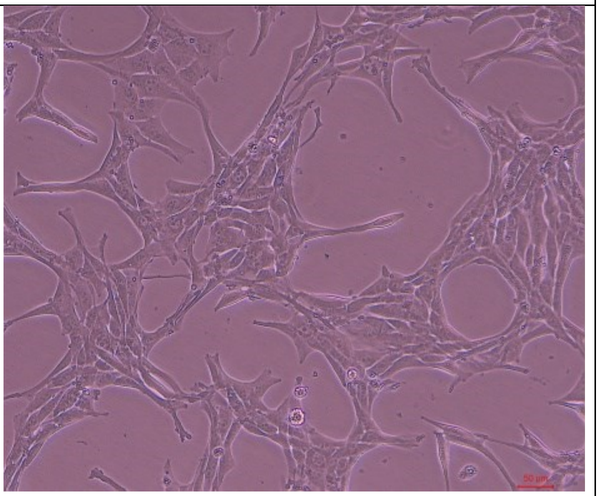<br>C2

**[$MoO_2(HL^5)$], 50 µM**<br><br>D1,D2. Reduction in the quantity of visible attached cells and an increase in the quantity of damaged cells which are round-shaped, detached and floating.	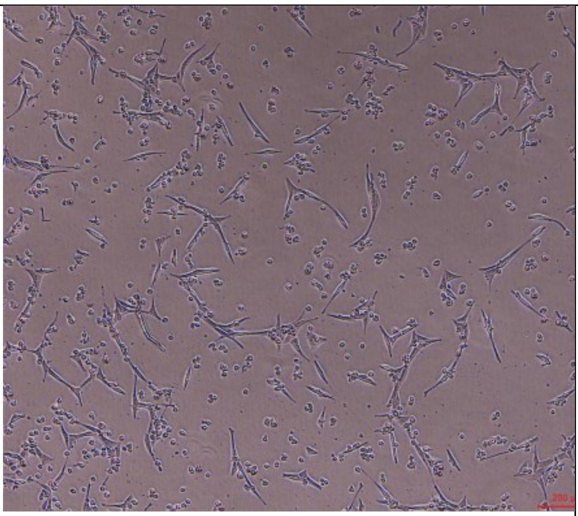<br>D1	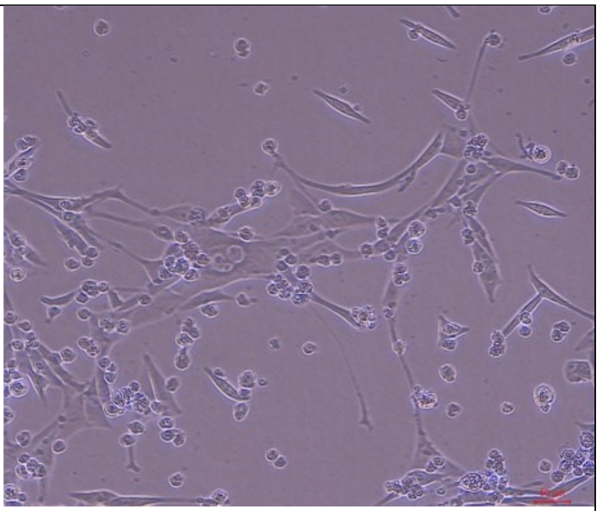<br>D2
**[$MoO_2(HL^5)$], 100 µM**<br><br>E1,E2. Significant reduction in the quantity of visible attached cells (with the normal cells being almost absent) and significant increase in the quantity of the damaged cells which are round-shaped, detached and floating.	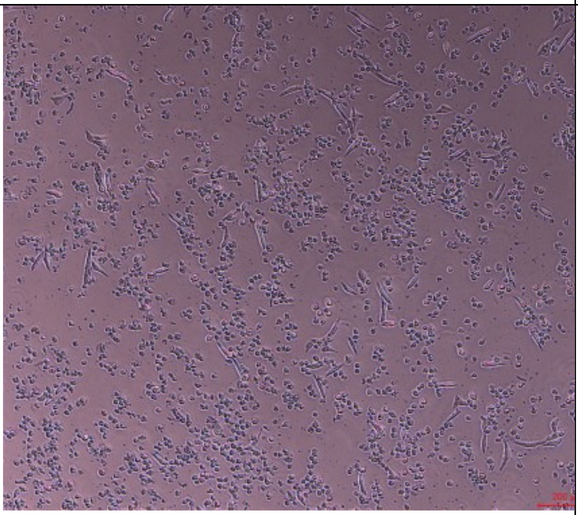<br>E1	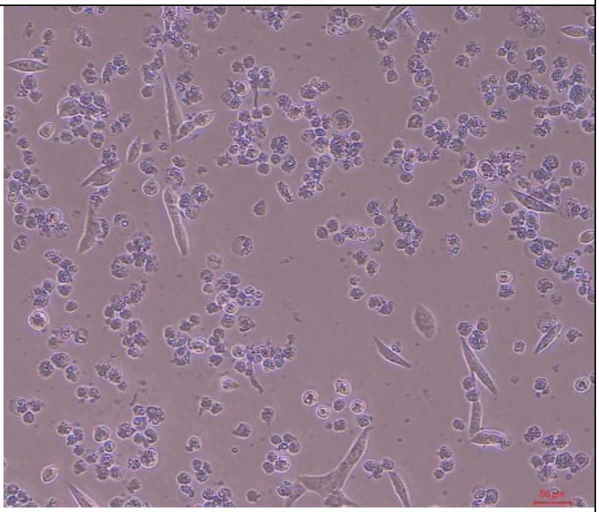<br>E2

3.3.1 [(+)$MoO_2(HL^5)$]

Microphotographs of control HT-22 cells and the cells incubated with [(+)$MoO_2(HL^5)$] (10 µM; 25 µM; 50 µM) for 48 hours

**Control**<br><br>A1,A2. Typical morphological structure of the control cells, which are attached, the cells shape is unchanged.	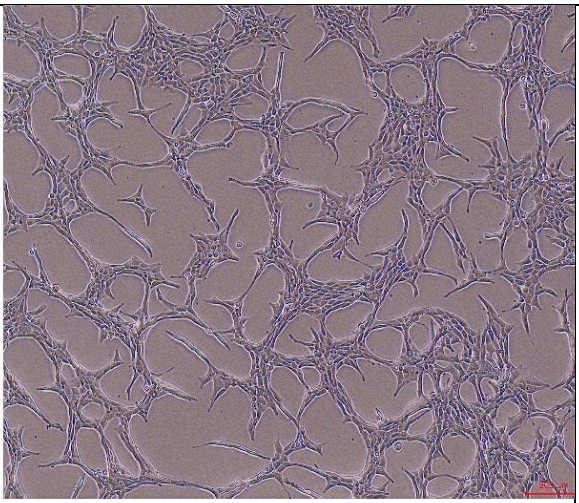<br>A1	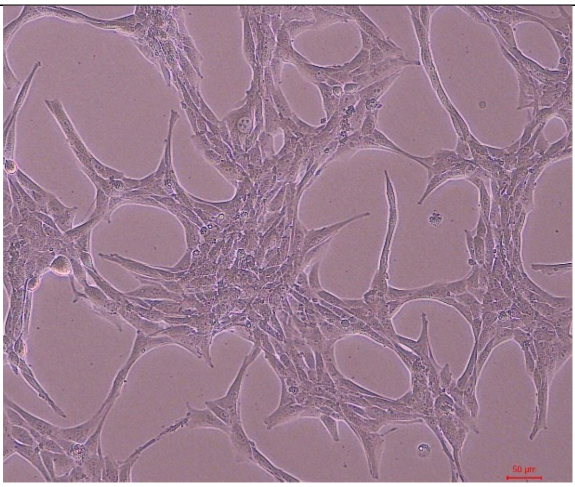<br>A2
**[(+)$MoO_2(HL^5)$], 10 µM**<br><br>B1,B2. The typical morphological structure of the cells is maintained, the cells are attached, the cells shape is unchanged, there are no visible changes in cell confluence compared with the microphotographs of the control cells (A1,A2).	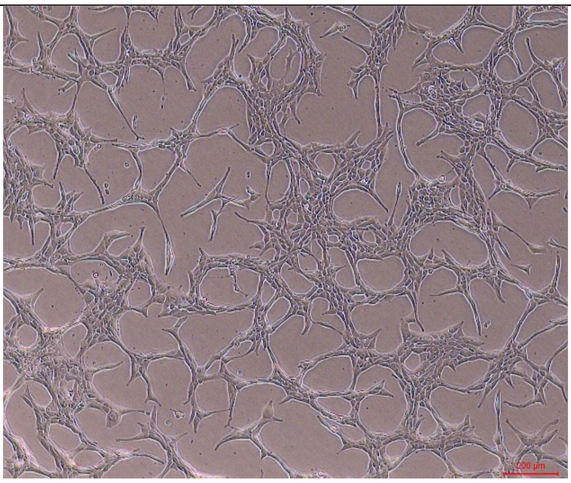<br>B1	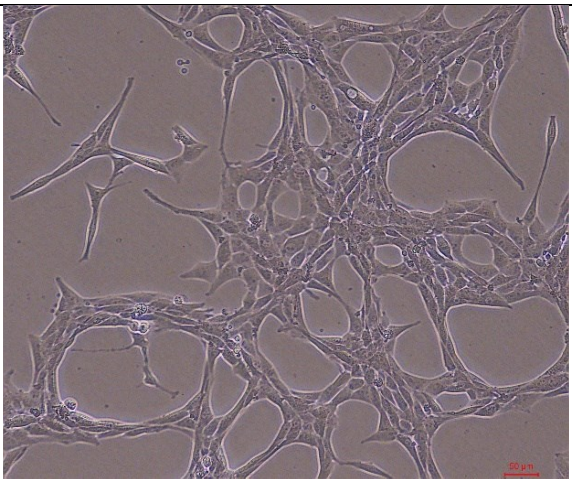<br>B2
**[(+)$MoO_2(HL^5)$], 25 µM**<br>C1,C2. The typical morphological structure of the cells is maintained, the cells are attached, the cells shape is unchanged, there are no visible changes in cell confluence compared with the microphotographs of the control cells (A1,A2).	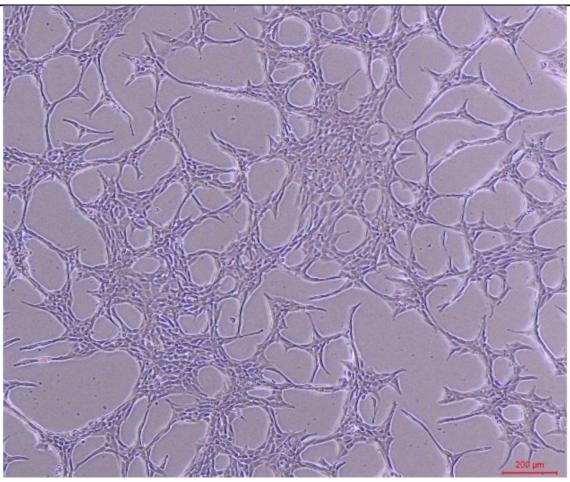<br>C1	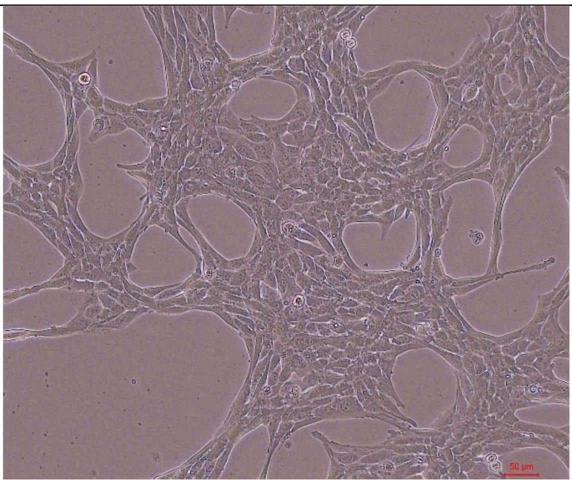<br>C2

**[(+)$MoO_2(HL^5)$],<br>50 µM**<br><br>D1,D2. The typical morphological structure of the cells is maintained, the cells are attached, the cells shape is unchanged, there are no visible changes in cell confluence compared with the microphotographs of the control cells (A1,A2).	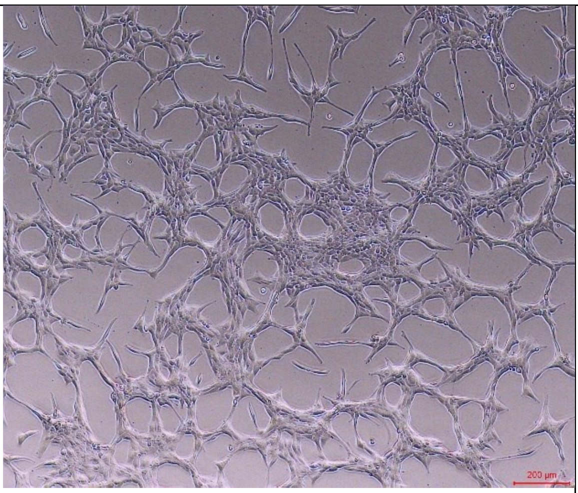<br>D1	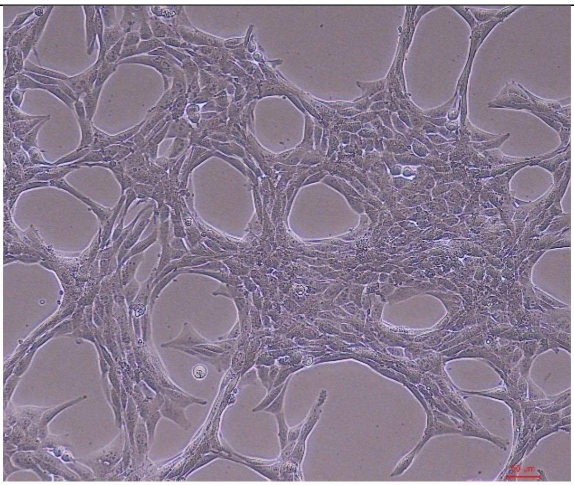<br>D2

3.3.2. [(+)$MoO_2(HL^5)$]

Microphotographs of control HT-22 cells and the cells incubated with [(+)$MoO_2(HL^5)$] (100 μM) for 48 hours

**Control**<br><br>E1,E2. Typical morphological structure of the control cells, which are attached, the cells shape is unchanged.	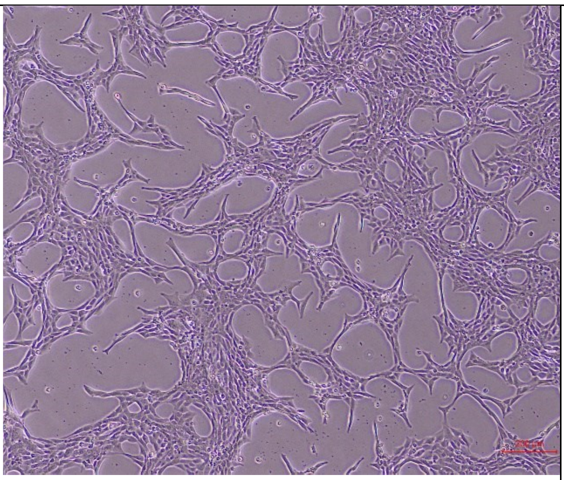<br>E1	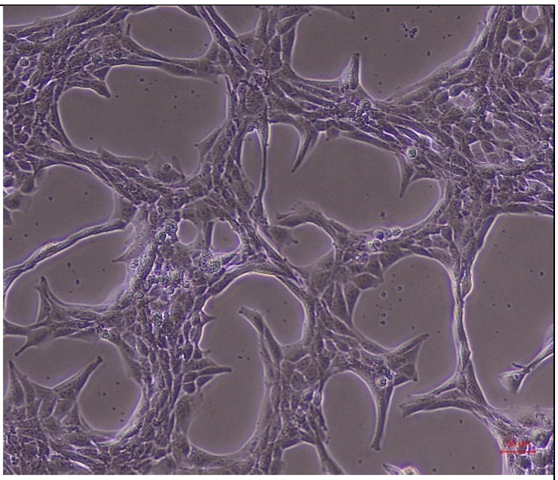<br>E2
**[(+)$MoO_2(HL^5)$], 100 μM**<br><br>F1,F2. The typical morphological structure of the cells is maintained, the cells are attached, the cells shape is unchanged, there are no visible changes in cell confluence compared with the microphotographs of the control cells (E1,E2).	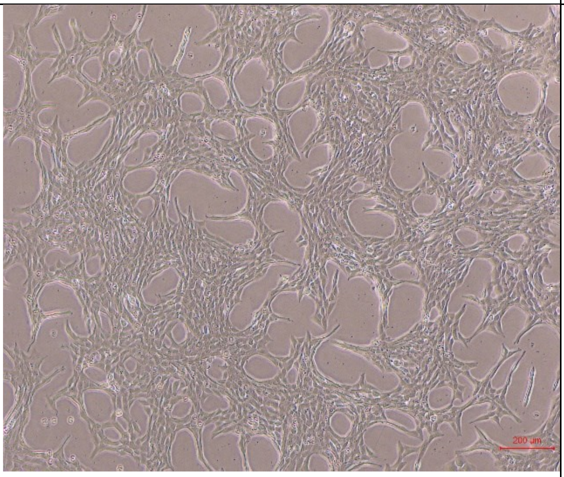<br>F1	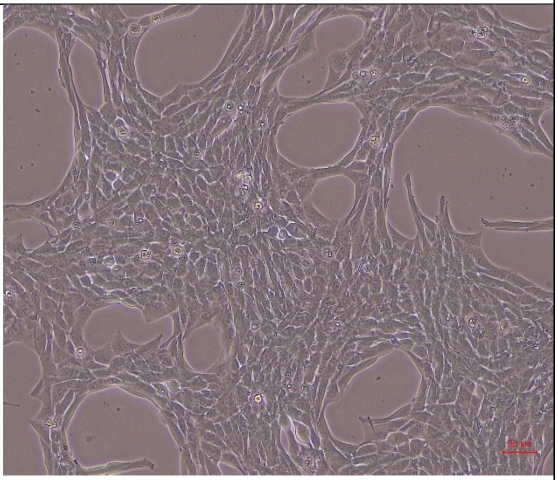<br>F2

Since not all concentrations were accessed at once, the correspondent representative control photos are given in each dataset.

3.4.1. [(+)$MoO_2(HL^1)(CH_3OH)$]

Microphotographs of control HT-22 cells and the cells incubated with [(+)$MoO_2(HL^1)(CH_3OH)$] (10 μM; 25 μM; 50 μM) for 48 hours

**Control**<br><br>A1,A2. Typical morphological structure of the control cells, which are attached, the cells shape is unchanged.	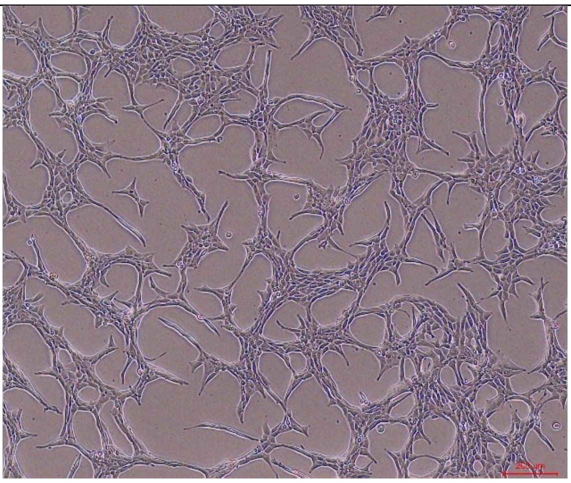<br>A1	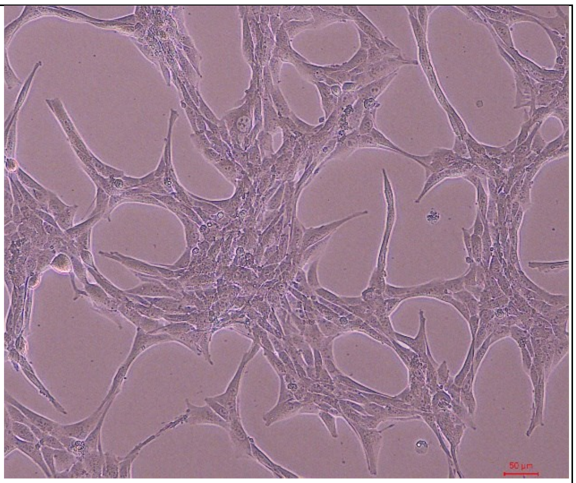<br>A2
**[(+)$MoO_2(HL^1)$ (CH_3OH)], 10 μM**<br><br>B1,B2. The typical morphological structure of the cells is maintained, the cells are attached, the cells shape is unchanged, there are no visible changes in cell confluence compared with the microphotographs of the control cells (A1,A2).	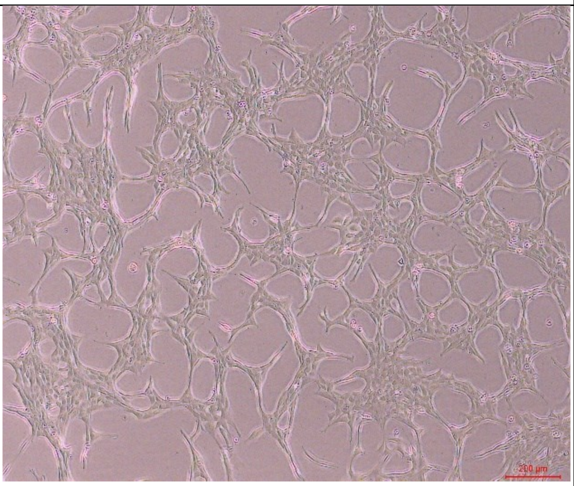<br>B1	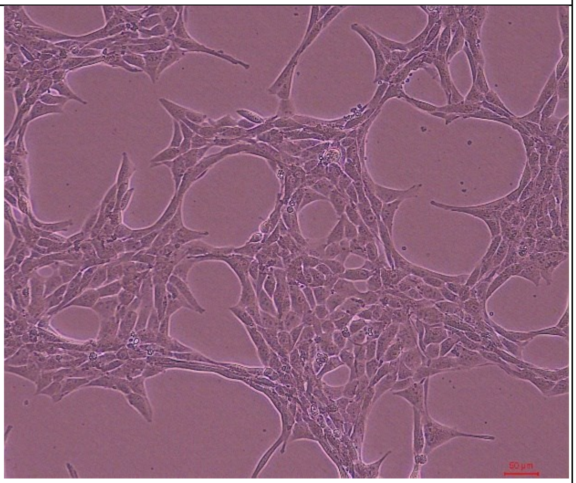<br>B2
**[(+)$MoO_2(HL^1)$ (CH_3OH)], 25 μM**<br><br>C1,C2. The typical morphological structure of the cells is maintained, the cells are attached, the cells shape is unchanged, there are no visible changes in cell confluence	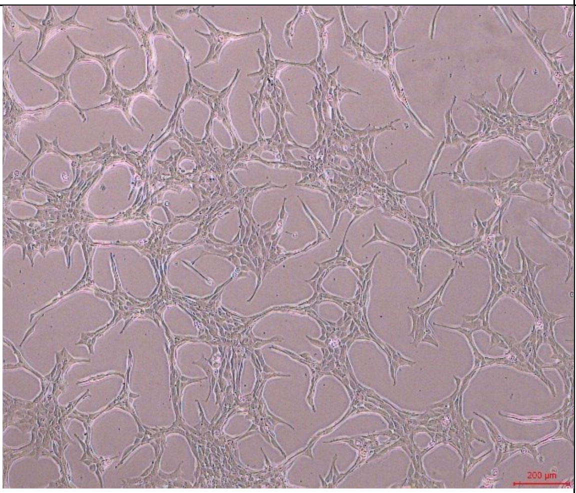<br>C1	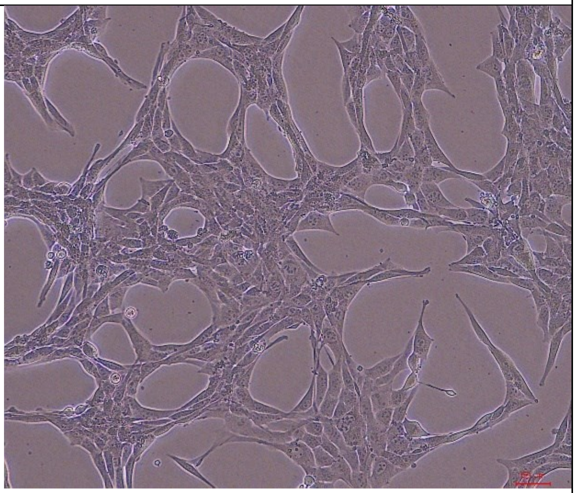<br>C2

compared with the microphotographs of the control cells (A1,A2).		
**[(+)$MoO_2(HL^1)$ (CH_3OH)],**<br>**50 μM**<br><br>D1,D2. The typical morphological structure of the cells is maintained, the cells are attached, the cells shape is unchanged, there are no visible changes in cell confluence compared with the microphotographs of the control cells (A1,A2).	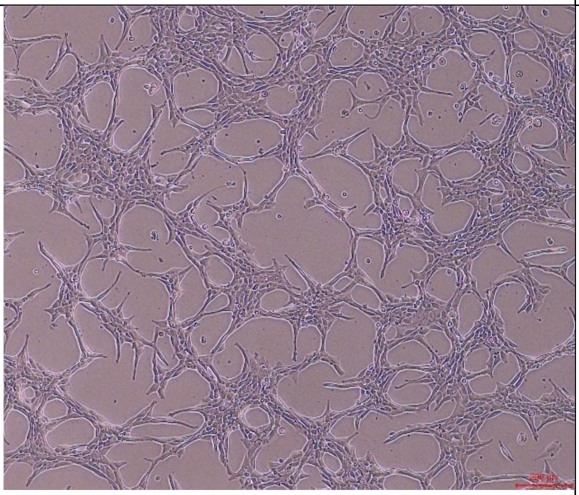<br>D1	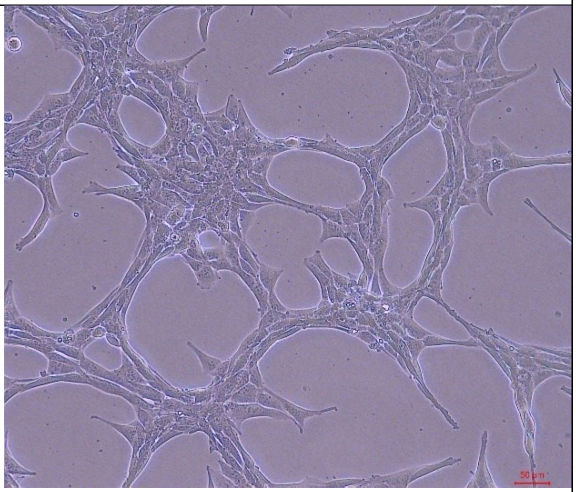<br>D2

3.4.2. [(+)$MoO_2(HL^1)(CH_3OH)$]

Microphotographs of control HT-22 cells and the cells incubated with [(+)$MoO_2(HL^1)$ (CH_3OH)], (100 µM) for 48 hours

**Control.**<br><br>E1,E2. Typical morphological structure of the control cells, which are attached, the cells shape is unchanged.	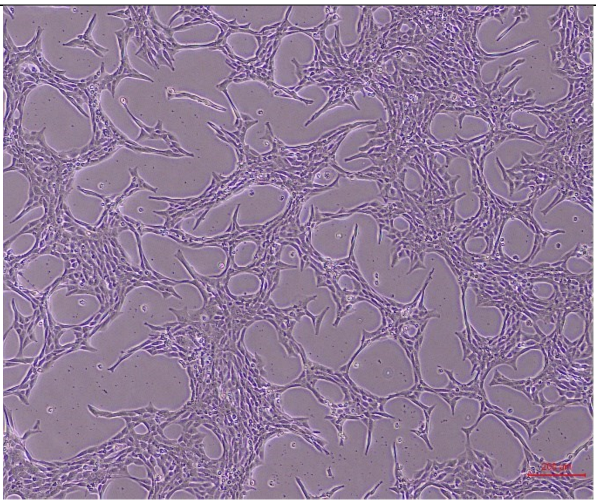<br>E1	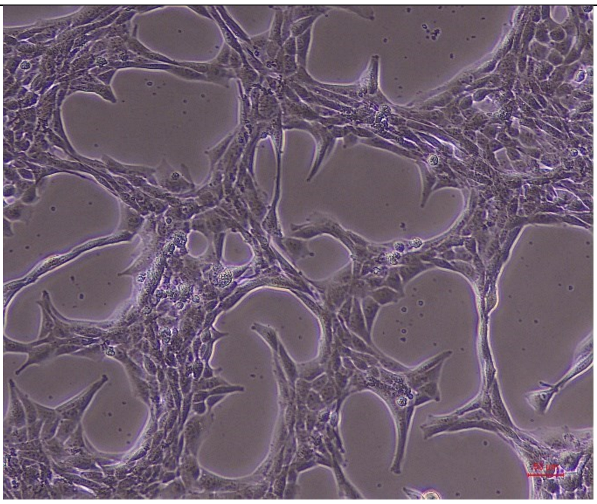<br>E2
**[(+)$MoO_2(HL^1)$ (CH_3OH)], 100 µM**<br><br>F1,F2. The typical morphological structure of the cells is maintained, the cells are attached, the cells shape is unchanged, there are no visible changes in cell confluence compared with the microphotographs of the control cells.	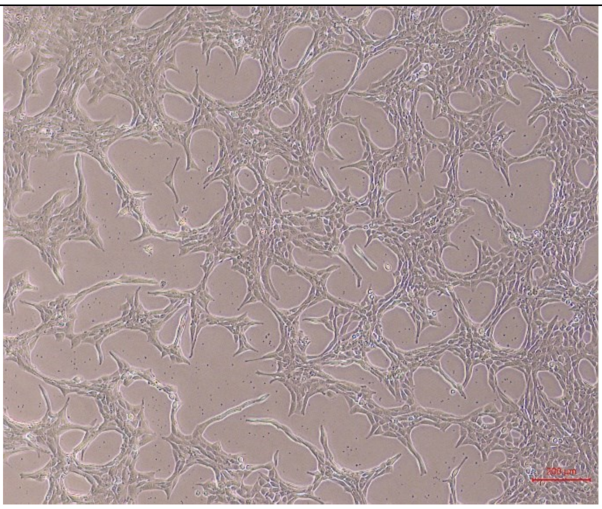<br>F1	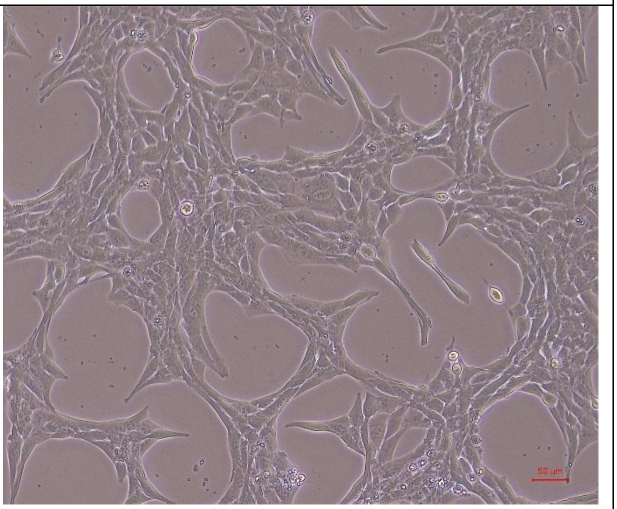<br>F2

Since not all concentrations were accessed at once, the correspondent representative control photos are given in each dataset.

3.5. $[MoO_2(HL^4)]$

Microphotographs of control HT-22 cells and the cells incubated with $[MoO_2(HL^4)]$ (10 µM; 25 µM; 50 µM; 100 µM) for 48 hours

**Control**<br><br>A1,A2. Typical morphological structure of the control cells, which are attached, the cells shape is unchanged.	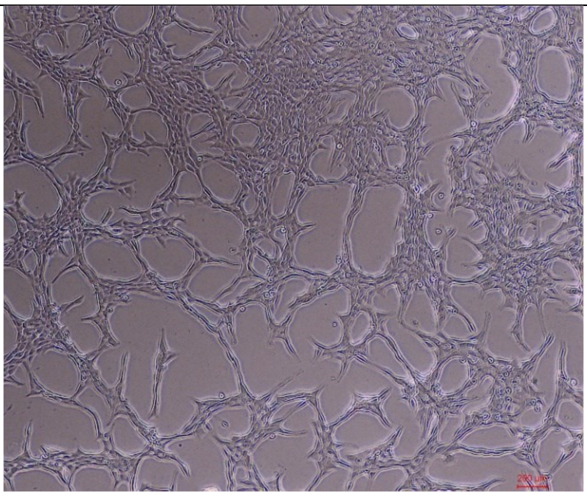<br>A1	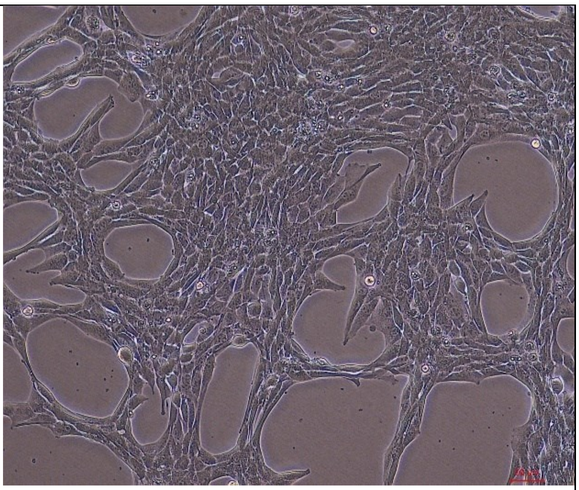<br>A2
**$[MoO_2(HL^4)]$, 10 µM**<br><br>B1,B2. The typical morphological structure of the cells is maintained, the cells are attached, the cells shape is unchanged, there are no visible changes in cell confluence compared with the microphotographs of the control cells.	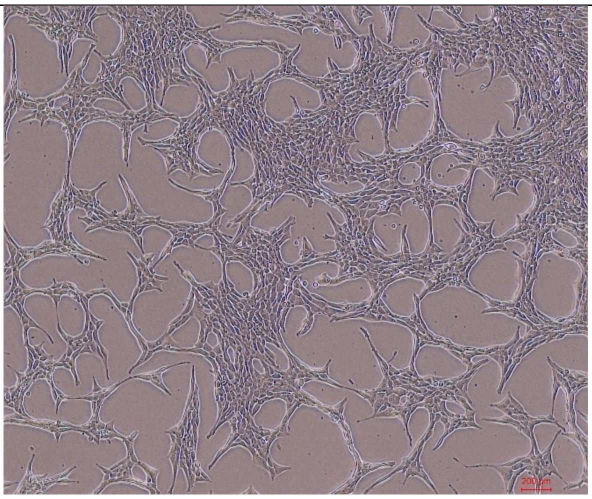<br>B1	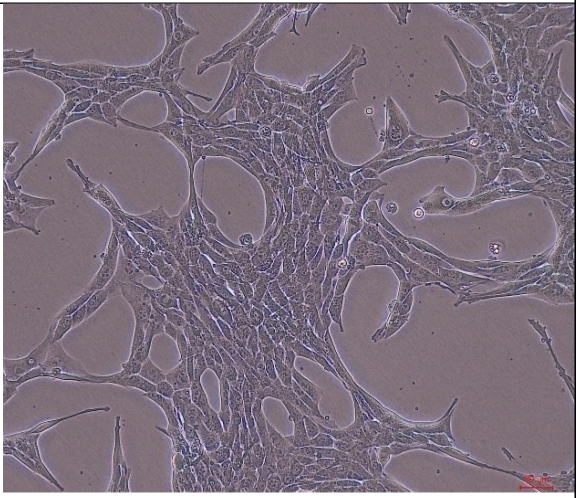<br>B2
**$[MoO_2(HL^4)]$, 25 µM**<br><br>C1,C2. The typical morphological structure of the cells is maintained, the cells are attached, the cells shape is unchanged, there are no visible changes in cell confluence compared with the microphotographs of the control cells.	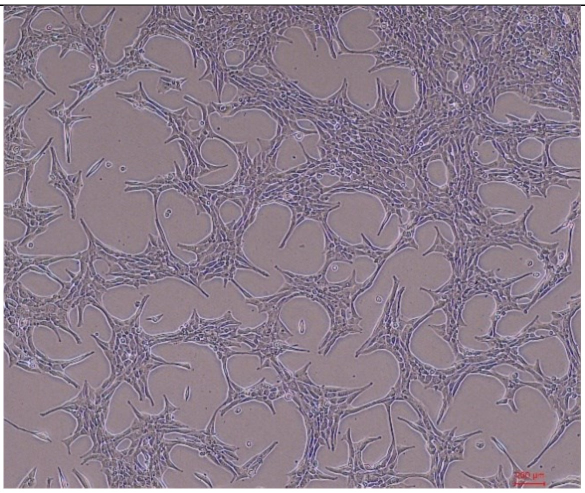<br>C1	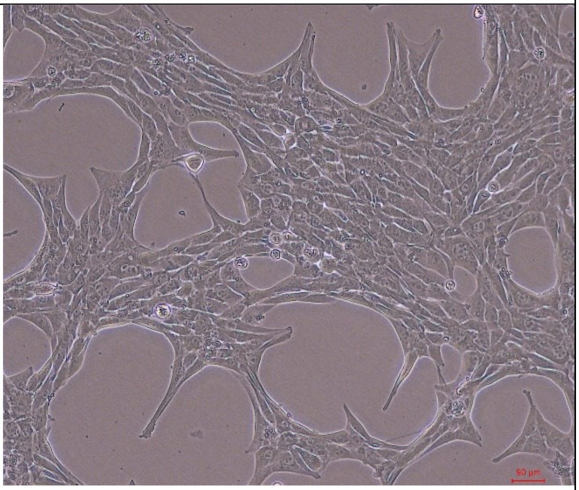<br>C2

<table>
<tr>
<td rowspan="2">[$MoO_2(HL^4)$], 50 µM

D1,D2,D3. Significant reduction in the quantity of visible attached cells and a significant increase in the quantity of highly damaged cells which are round-shaped, detached and floating, presence of cell debris and crystal-like structures (D1,D3, probably formed by the studied compound).</td>
<td>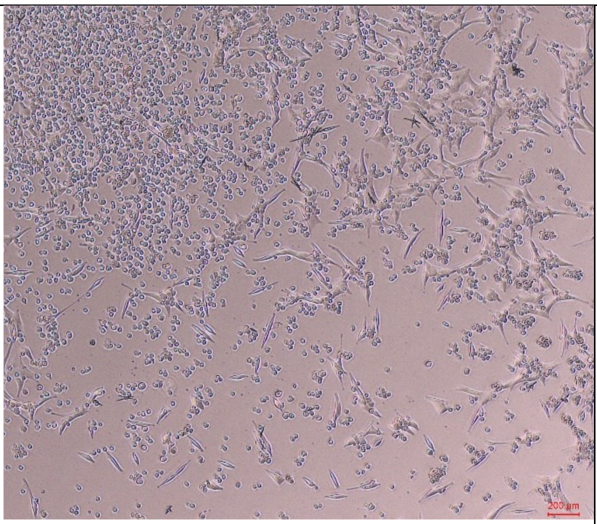
D1</td>
<td>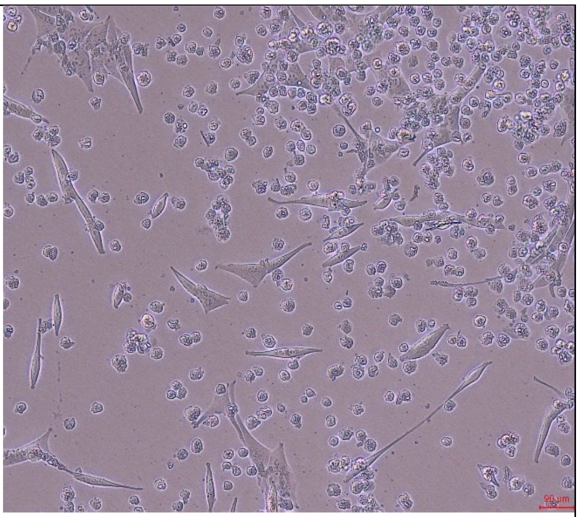
D2</td>
</tr>
<tr>
<td>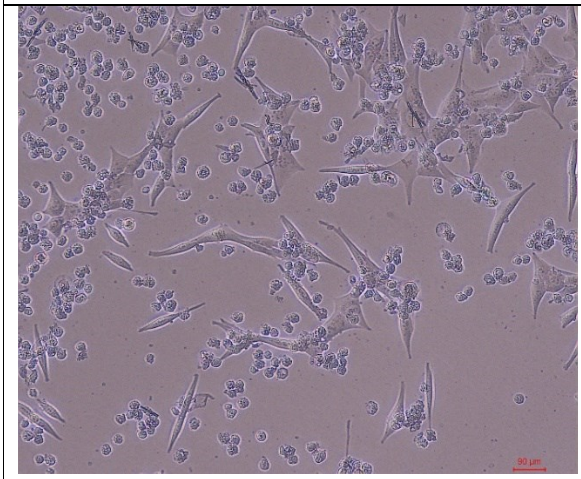
D3</td>
<td></td>
</tr>
<tr>
<td>[$MoO_2(HL^4)$], 100 µM

E1,E2,E3,E4,E5. Abrupt reduction in the quantity of visible attached cells (with the normal cells being almost absent) significant increase in the quantity of highly damaged cells which are round-shaped,</td>
<td>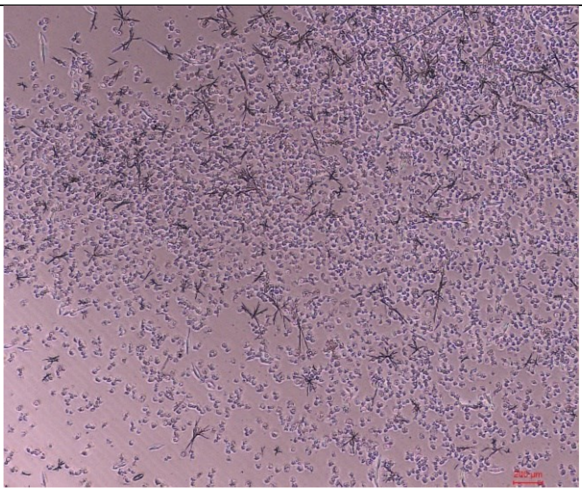
E1</td>
<td>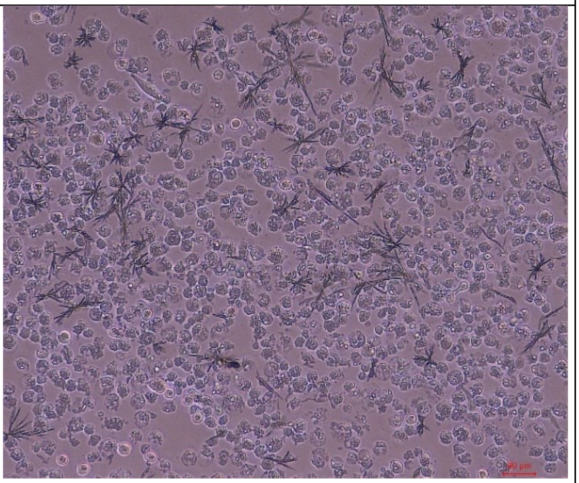
E2</td>
</tr>
</table>

detached and floating, presence of cell debris in considerable quantities and clearly visible crystal-like structures (probably formed by the studied compound).	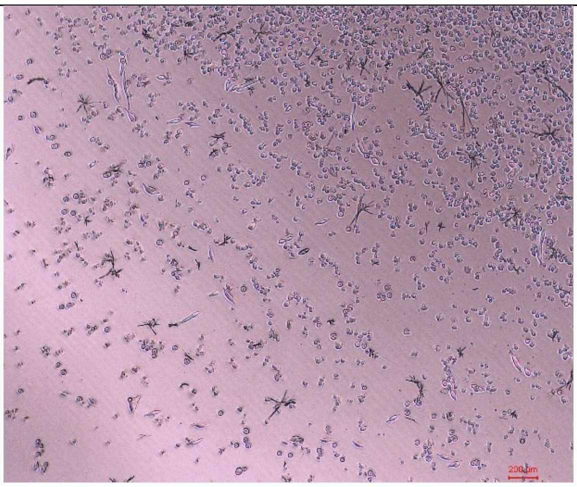 E3	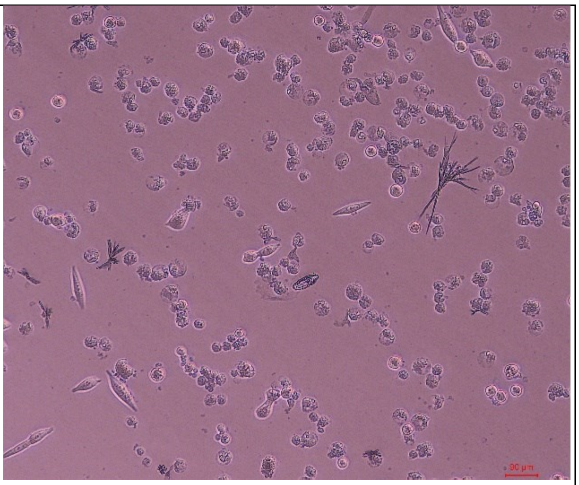 E4
	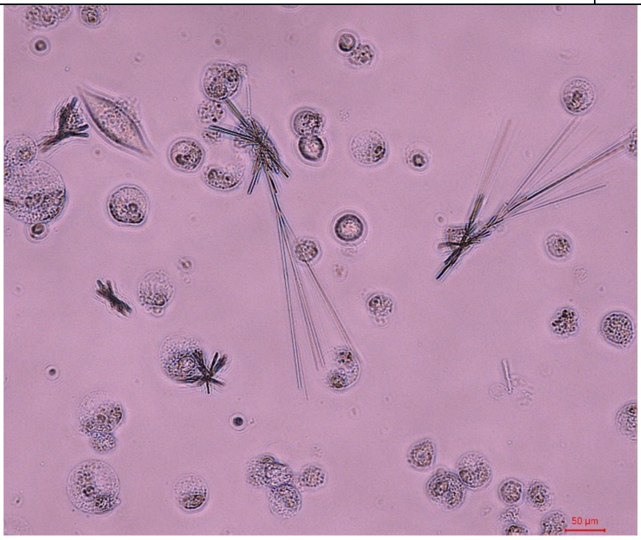 E5	

Figures E6,E7. Crystal-like structures among severely damaged HT-22 cells exposed to **[$MoO_2(HL^4)$]** at a concentration of 100 μM (24 hours)

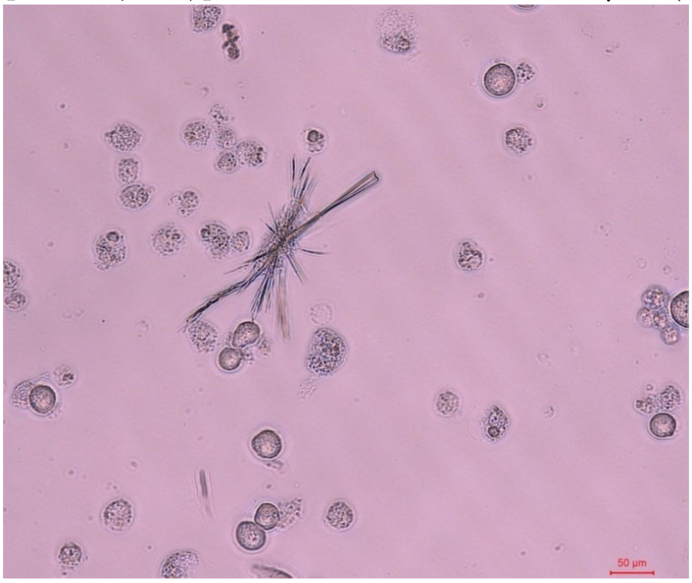

E6

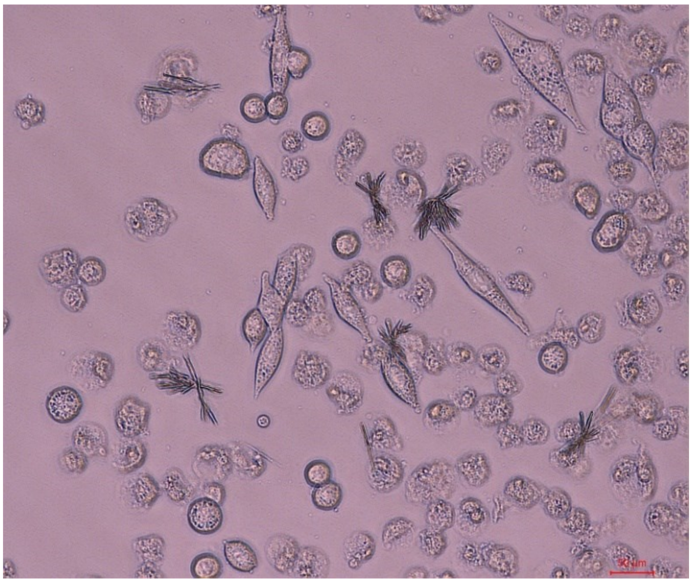

E7

Figure E8. Crystal-like structures visible after **[$MoO_2(HL^4)$]** was dissolved in medium and incubated for 48 hours

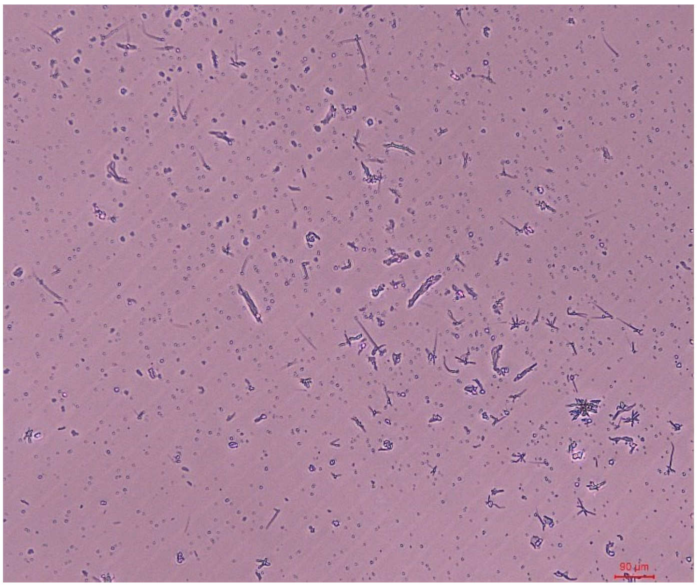

E8

Figure E9,E10. Crystal-like structures visible after **[$MoO_2(HL^4)$]** was dissolved in medium and incubated for 24 hours	Figures E11, E12. Control medium incubated for 24 hours.
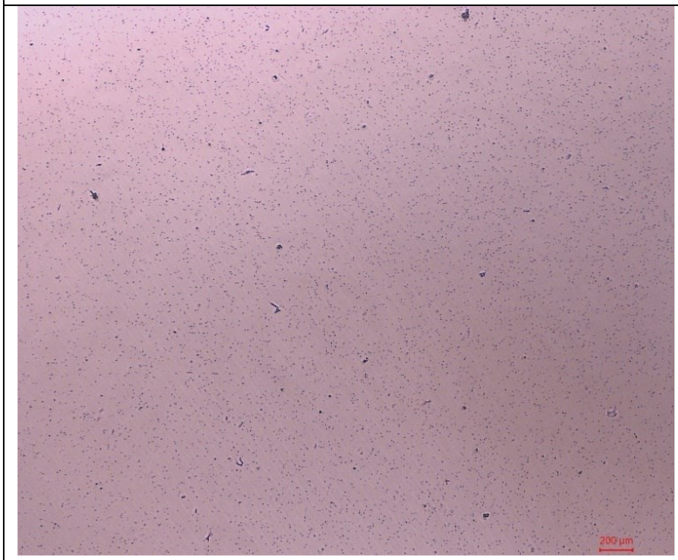 E9. 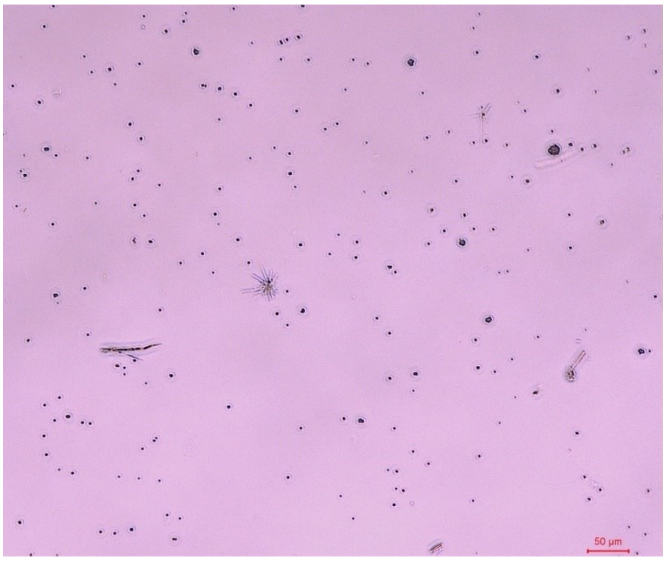 E10	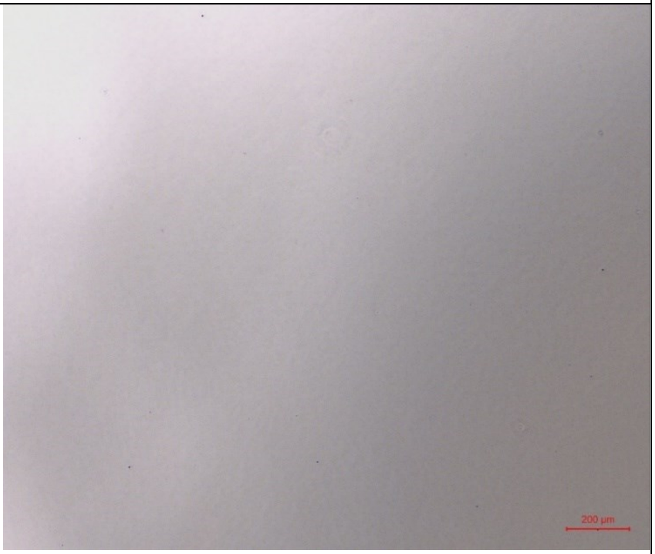 E11. 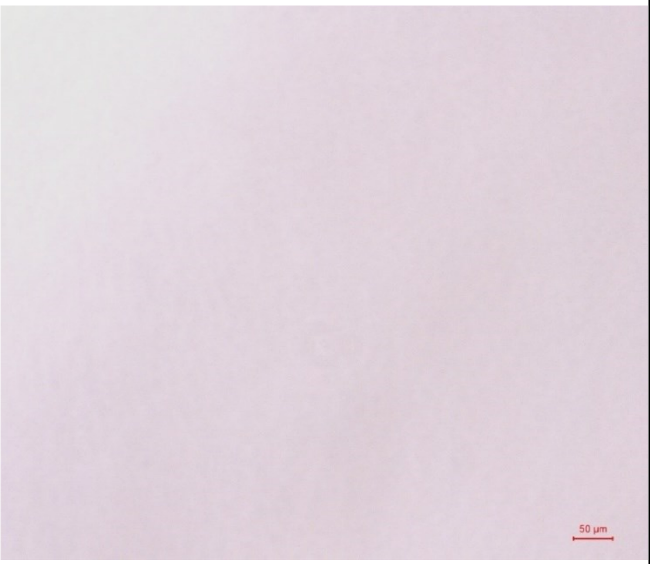 E12
