# Supplementary material for: Structure-Dependent Cytotoxic and Cytoprotective Effects of Molybdenum(VI) Schiff Base Complexes in the Neuronal Cells In Vitro
Source: Molecules. 2026 Aug 18;31(16):2883. doi: 10.3390/molecules31162883 (PMC13515801; doi:10.3390/molecules31162883)
Supplement: Supplementary file 1 [file molecules-31-02883-s001.zip › SUPPLEMENTARY FILE S4.pdf]

Supplementary file 4.

The effects of [$MoO_2(HL^3)$] and [$MoO_2(HL^2)$] *per se* on HT-22 cells viability (48 hours)

4.1.1. $[MoO_2(HL^3)]$

Microphotographs of control HT-22 cells and the cells incubated with $[MoO_2(HL^3)]$ (0.1 µM; 0.5 µM) for 48 hours

**Control**<br><br>A1,A2. Typical morphological structure of the control cells, which are attached, the cells shape is unchanged.	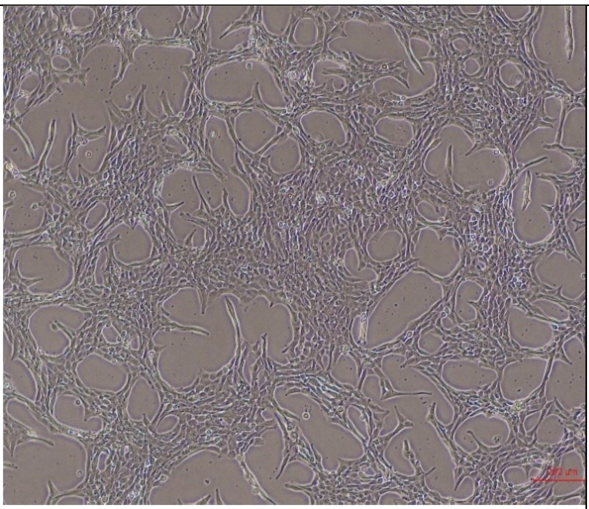<br>A1	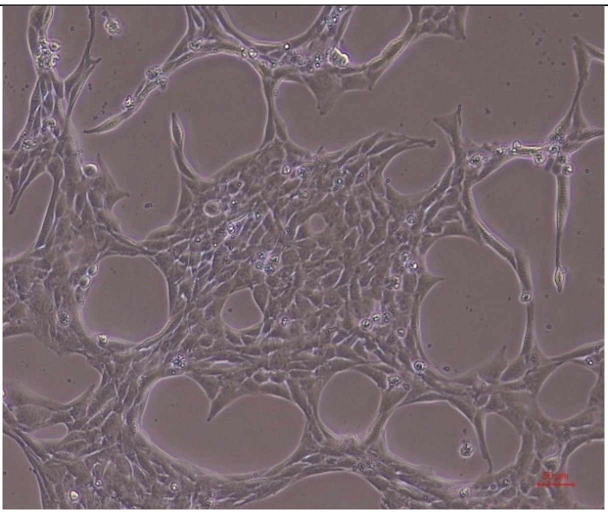<br>A2
**$[MoO_2(HL^3)]$, 0.1 µM**<br><br>B1,B2. The typical morphological structure of the cells is maintained, the cells are attached, there are no significant visible changes in cell confluence compared with the microphotographs of the control cells (A1,A2).	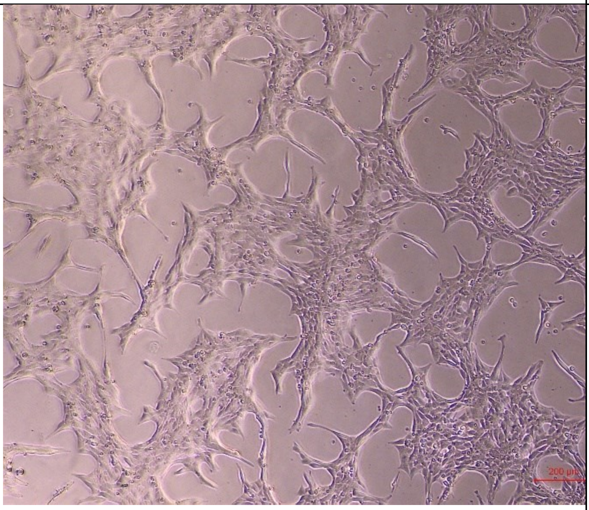<br>B1	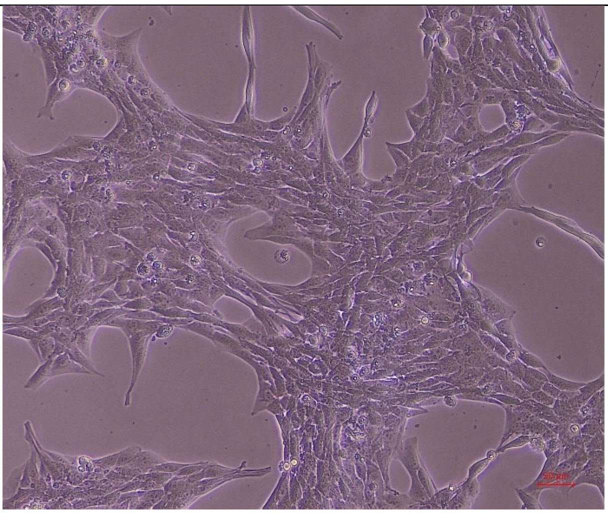<br>B2
**$[MoO_2(HL^3)]$, 0.5 µM**<br><br>C1,C2. The typical morphological structure of the cells is maintained, the cells are attached, there are no significant visible changes in cell confluence compared with the microphotographs of the control cells (A1,A2).	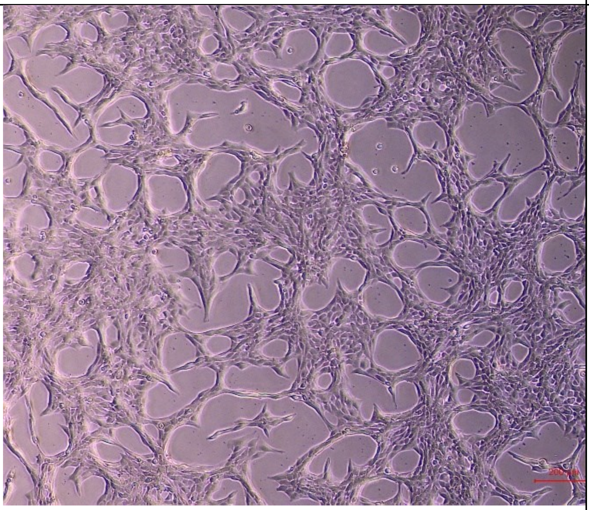<br>C1	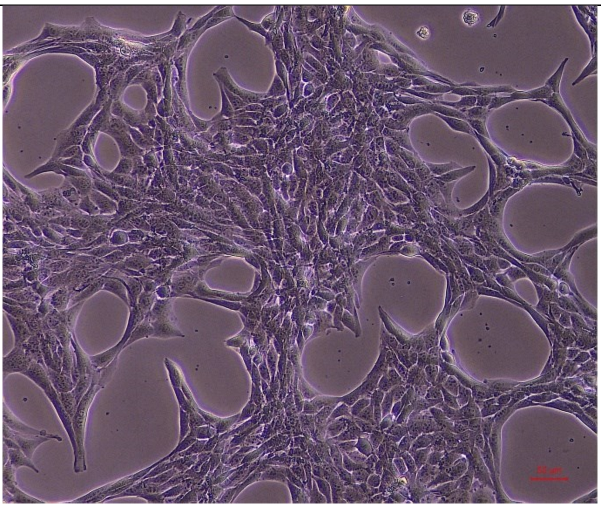<br>C2

4.1.2. [$MoO_2(HL^3)$]

Microphotographs of control HT-22 cells and the cells incubated with [$MoO_2(HL^3)$] (1.0 μM; 2.5 μM; 5 μM) for 48 hours

**Control**<br><br>D1,D2. Typical morphological structure of the control cells, which are attached, the cells shape is unchanged.	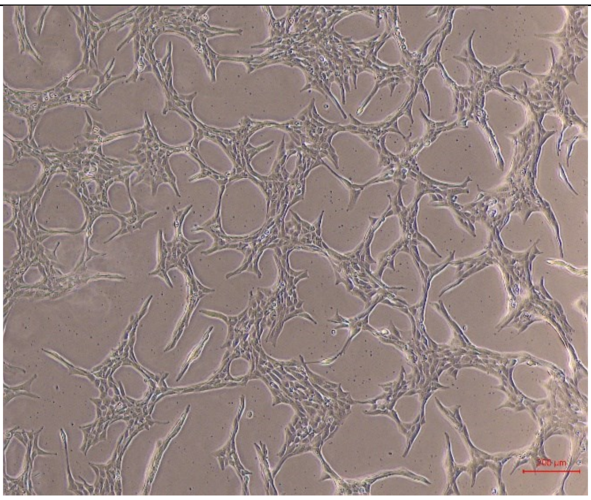<br>D1	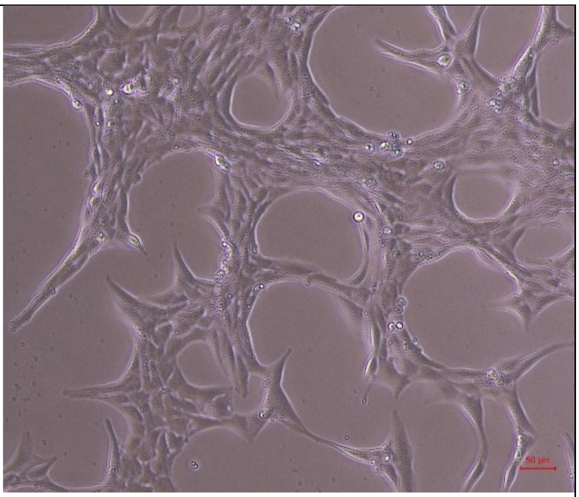<br>D2
**[$MoO_2(HL^3)$], 1.0 μM**<br>E1,E2. The typical morphological structure of the cells is maintained, the cells are attached, there are no significant visible changes in cell confluence compared with the microphotographs of the control cells (D1,D2).	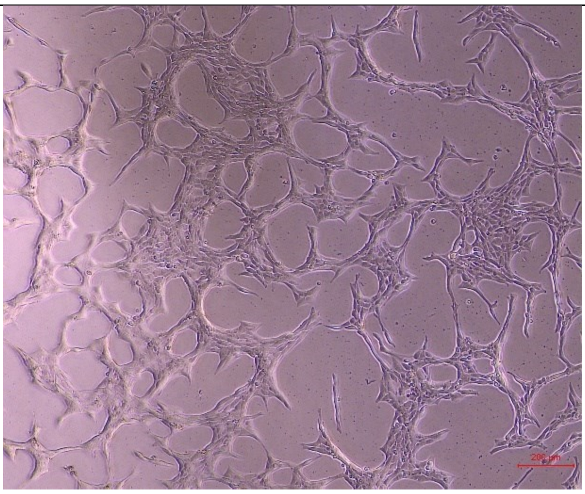<br>E1	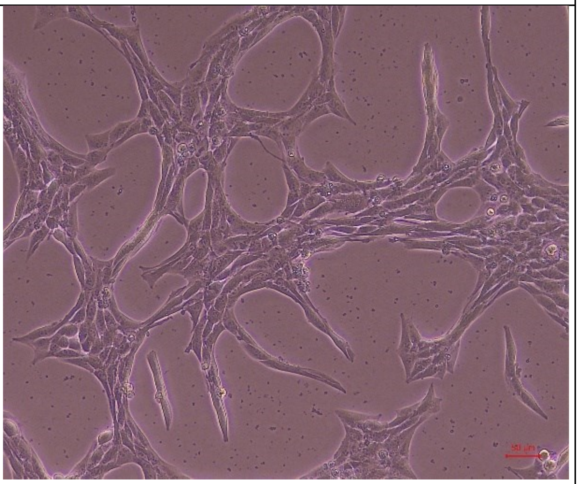<br>E2
**[$MoO_2(HL^3)$], 2.5 μM**<br><br>F1,F2. The typical morphological structure of the cells is maintained, the cells are attached, there are no significant visible changes in cell confluence compared with the microphotographs of the control cells (D1,D2).	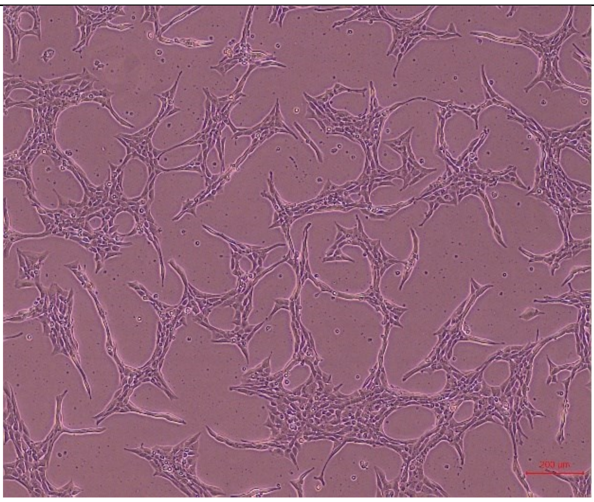<br>F1	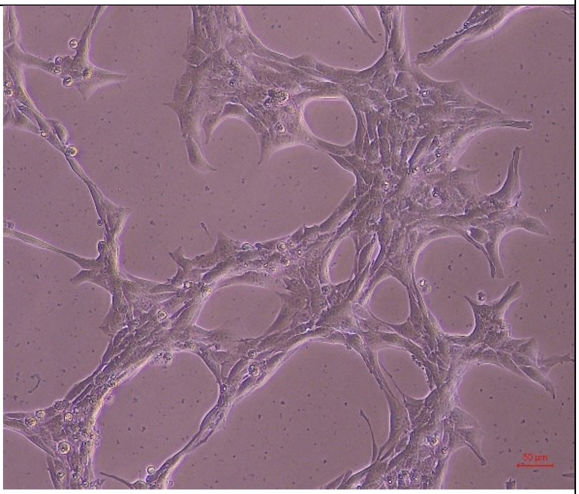<br>F2

**[$MoO_2(HL^3)$],<br>5 μM**<br><br>G1,G2. The typical morphological structure of the cells is maintained, the cells are attached, there are no significant visible changes in cell confluence compared with the microphotographs of the control cells (D1,D2).	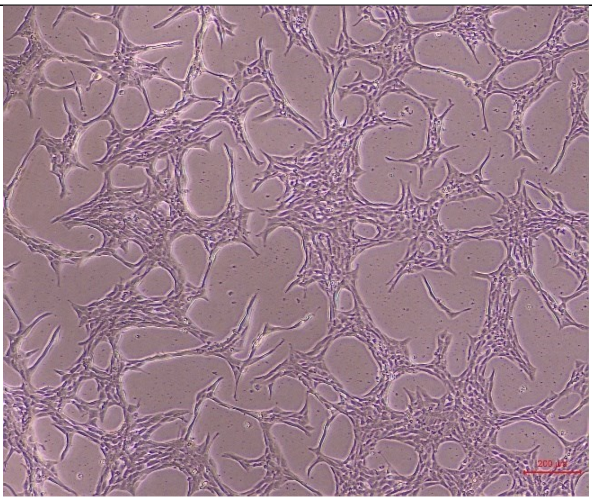<br>G1	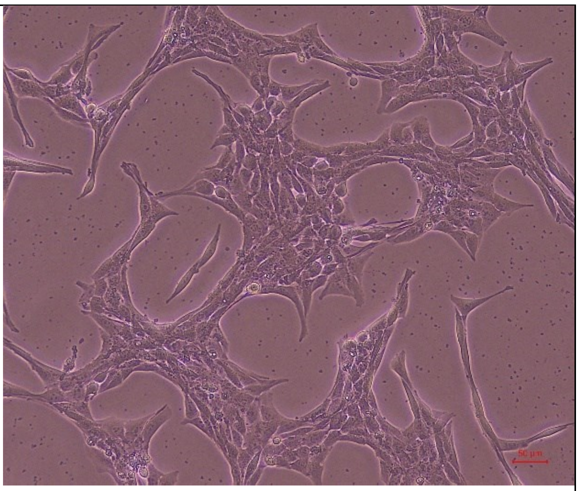<br>G2

4.1.3. [$MoO_2(HL^3)$]

Microphotographs of control HT-22 cells and the cells incubated with [$MoO_2(HL^3)$] (10 µM; 25 µM; 50 µM) for 48 hours

**Control**<br><br>H1,H2. Typical morphological structure of the control cells, which are attached, the cells shape is unchanged.	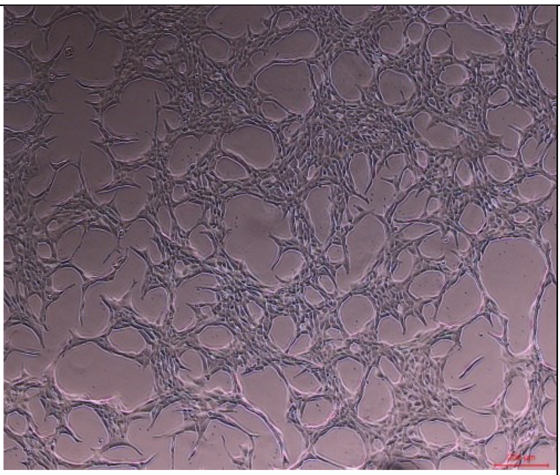<br>H1	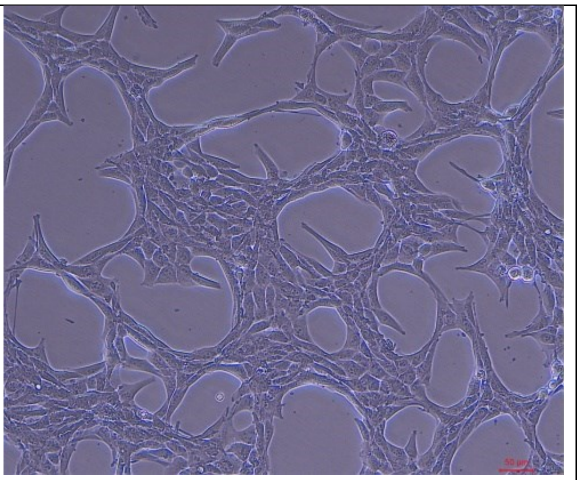<br>H2
**[$MoO_2(HL^3)$], 10 µM**<br><br>I1,I2. The typical morphological structure of the cells is maintained, the cells are attached, there are no significant visible changes in cell confluence compared with the microphotographs of the control cells (H1,H2).	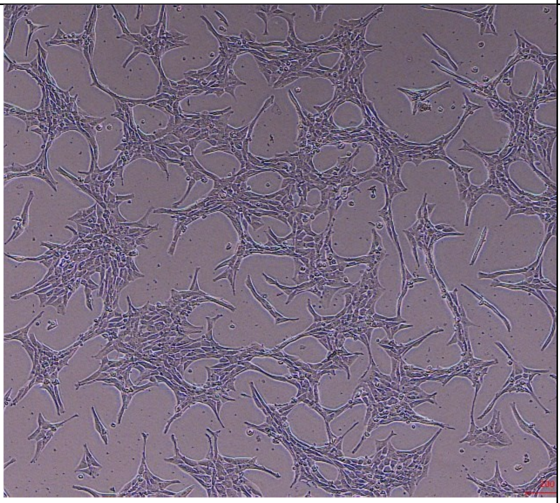<br>I1	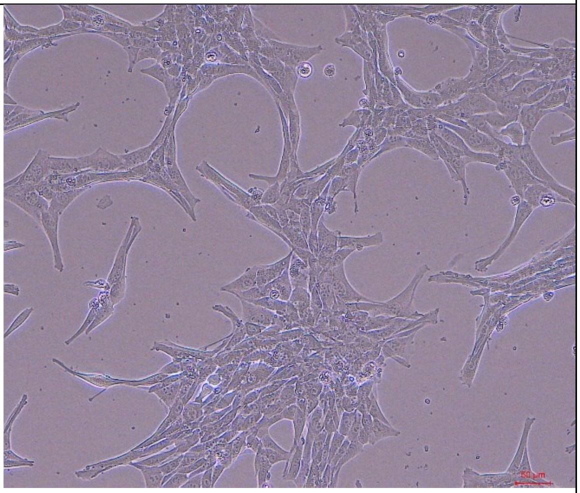<br>I2
**[$MoO_2(HL^3)$], 25 µM**<br><br>J1,J2. The typical morphological structure of the cells is maintained, the cells are attached, there are no significant visible changes in cell confluence compared with the microphotographs of the control cells (H1,H2).	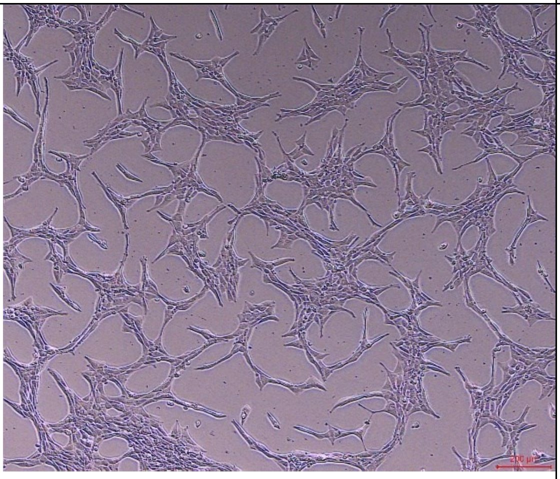<br>J1	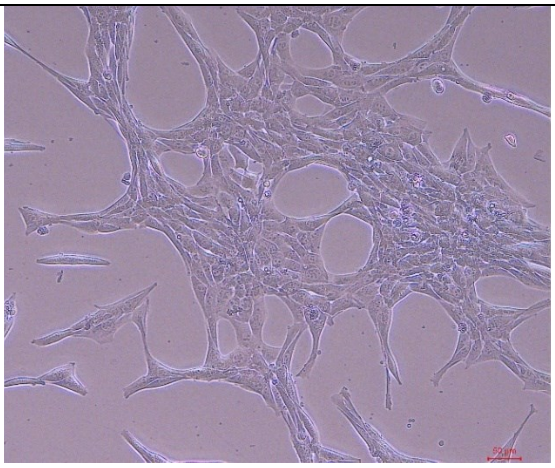<br>J2

**$[MoO_2(HL^3)]$, 50 µM**<br><br>K1,K2. The typical morphological structure of the cells is maintained, the cells are attached, cell confluence is moderately decreased, presence of single damaged cells, which are round-shaped, detached and floating.	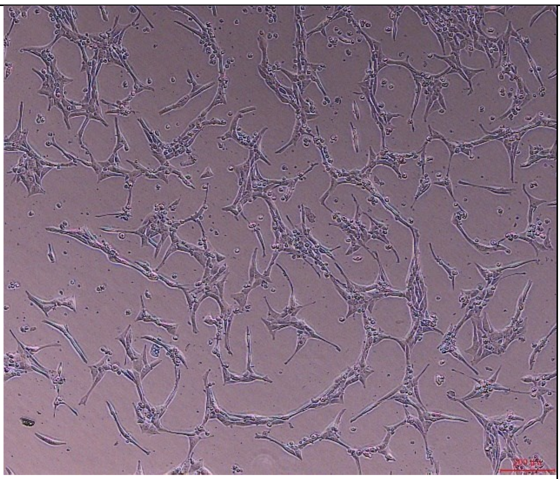<br>K1	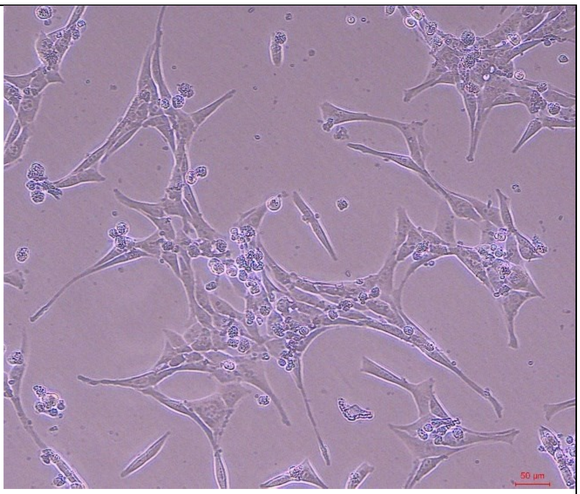<br><br>K2

4.1.4. [$MoO_2(HL^3)$]

Microphotographs of control HT-22 cells and the cells incubated with [$MoO_2(HL^3)$] (100 µM) for 48 hours

**Control.**<br><br>L1,L2. Typical morphological structure of the control cells, which are attached, the cells shape is unchanged.	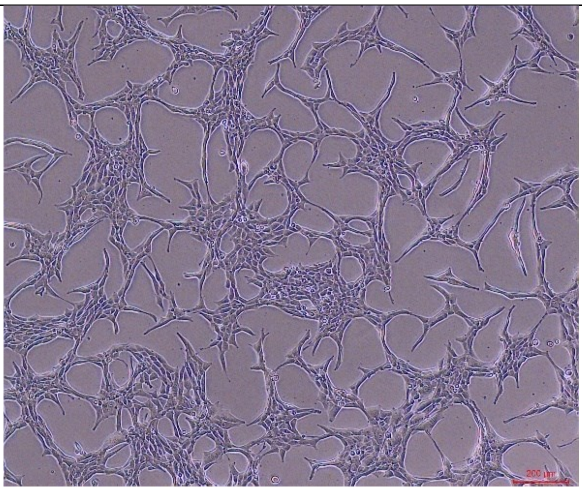<br>L1	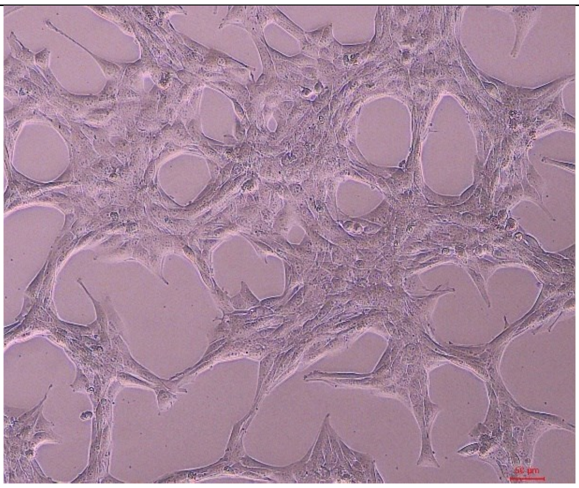<br>L2
**[$MoO_2(HL^3)$], 100 µM**<br><br>K1,K2. Significant decrease in cell confluence, change in the shape of the cells, among which there are elongated. Presence of a significant quantity of severely damaged cells which are round-shaped, detached and floating.	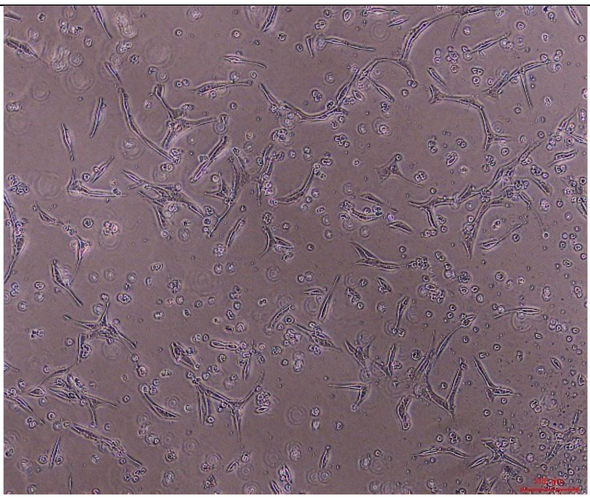<br>M1	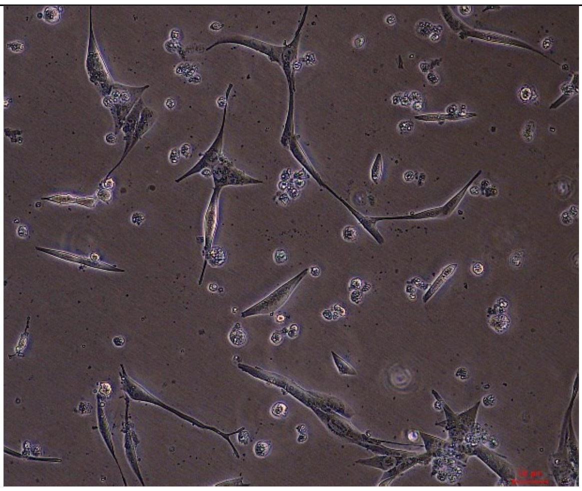<br>M2

Since not all concentrations were accessed at once, several representative control photos are given.

4.2. [$MoO_2(HL^2)$]

Microphotographs of control HT-22 cells and the cells incubated with [$MoO_2(HL^2)$] (1.0 µM; 5 µM; 10 µM; 25 µM; 50 µM; 100 µM) for 48 hours

**Control**<br><br>A1,A2. Typical morphological structure of the control cells, which are attached, the cells shape is unchanged.	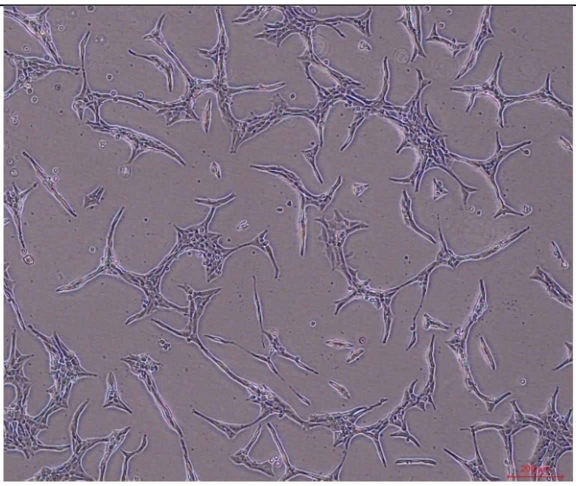<br>A1	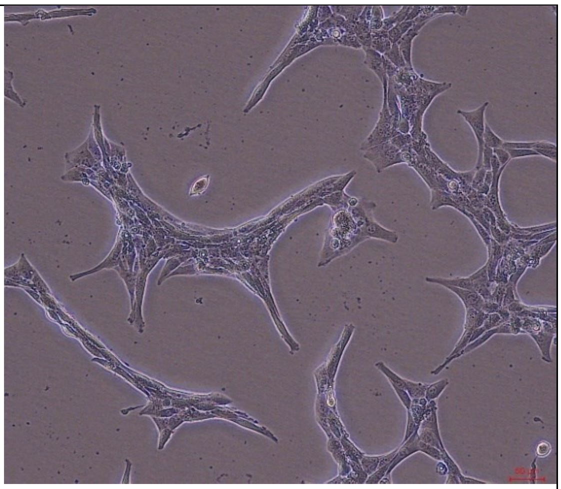<br>A2
**[$MoO_2(HL^2)$], 1.0 µM**<br><br>B1,B2. The typical morphological structure of the cells is maintained, the cells are attached, there are no significant visible changes in cell confluence compared with the microphotographs of the control cells (A1,A2).	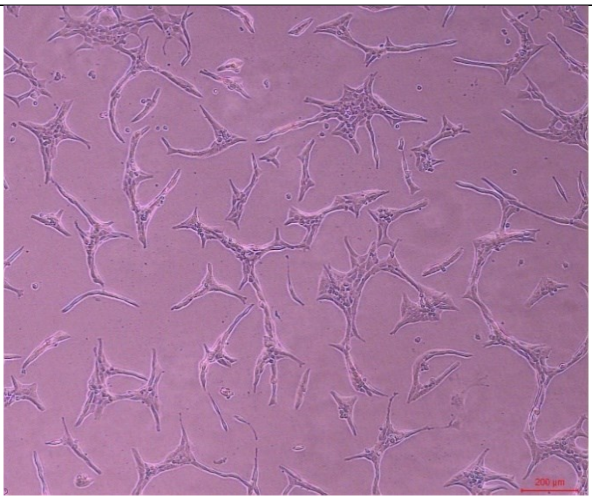<br>B1	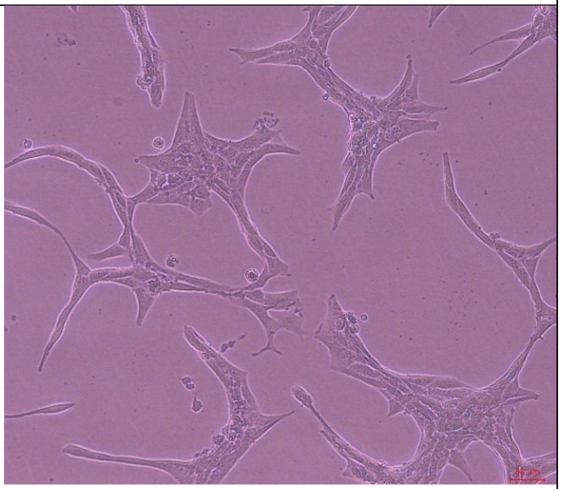<br>B2
**[$MoO_2(HL^2)$], 5 µM**<br><br>C1,C2. The typical morphological structure of the cells is maintained, the cells are attached, there are no significant visible changes in cell confluence compared with the microphotographs of the control cells (A1,A2).	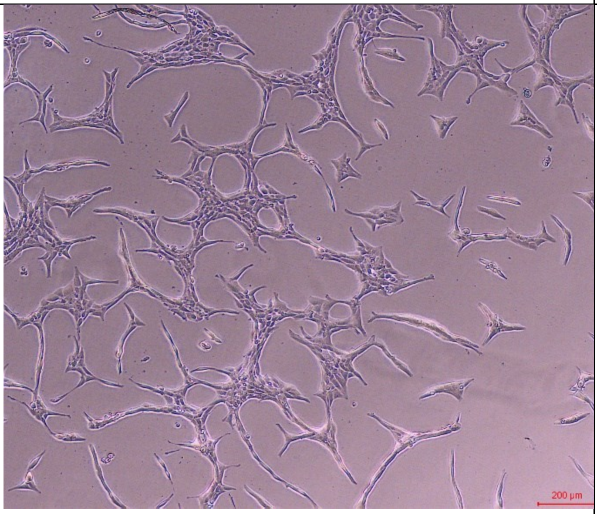<br>C1	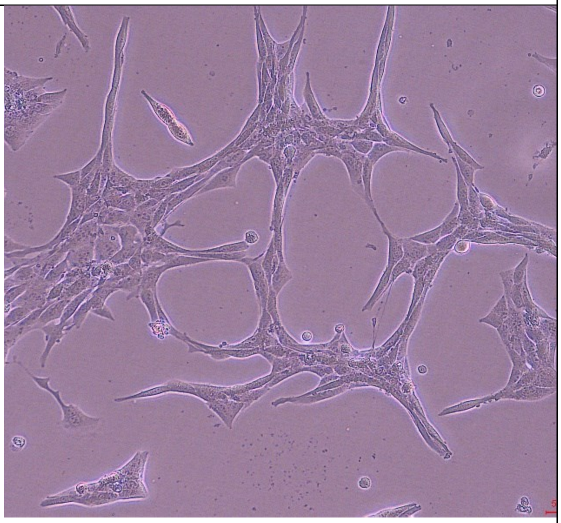<br>C2

**$[MoO_2(HL^2)]$,<br>10 µM**<br><br>D1,D2. The typical morphological structure of the cells is maintained, the cells are attached, there are no significant visible changes in cell confluence compared with the microphotographs of the control cells (A1,A2).	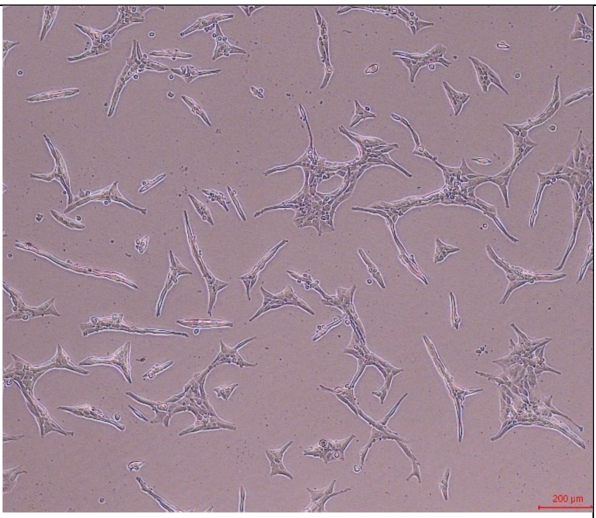<br>D1	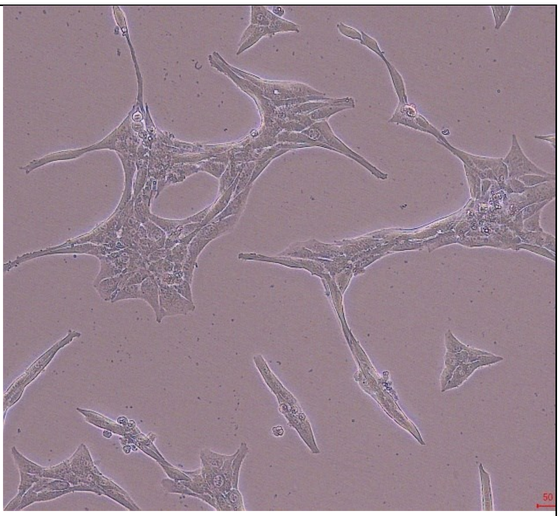<br>D2
**$[MoO_2(HL^2)]$,<br>25 µM**<br><br>E1,E2. The typical morphological structure of the cells is maintained, the cells are attached, there are no significant visible changes in cell confluence compared with the microphotographs of the control cells (A1,A2).	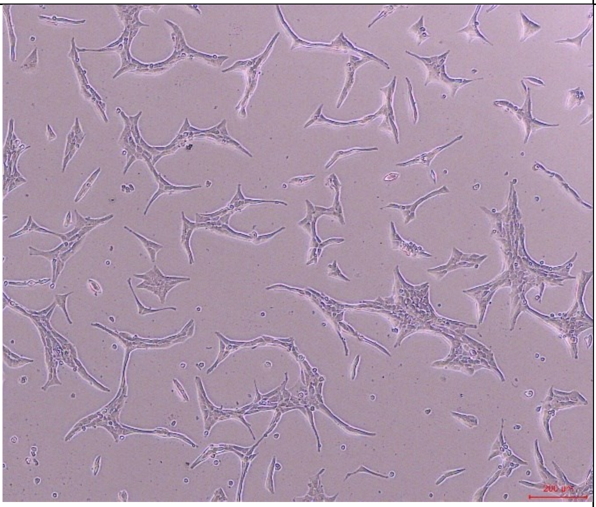<br>E1	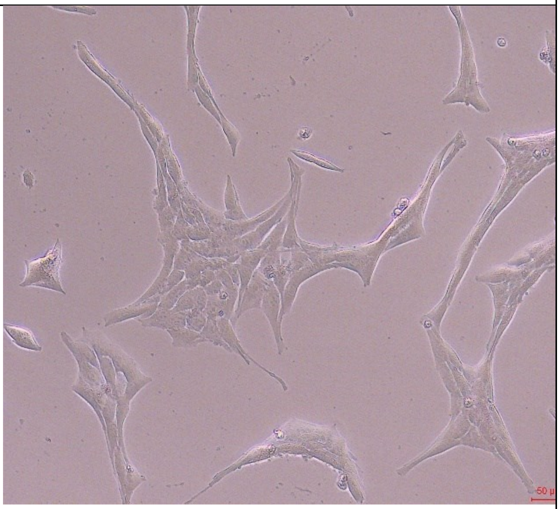<br>E2
**$[MoO_2(HL^2)]$,<br>50 µM**<br><br>F1,F2. The typical morphological structure of the cells is maintained, the cells are attached, cell confluence is moderately decreased, presence of a moderate amount of damaged cells, which are round-shaped, detached and floating.	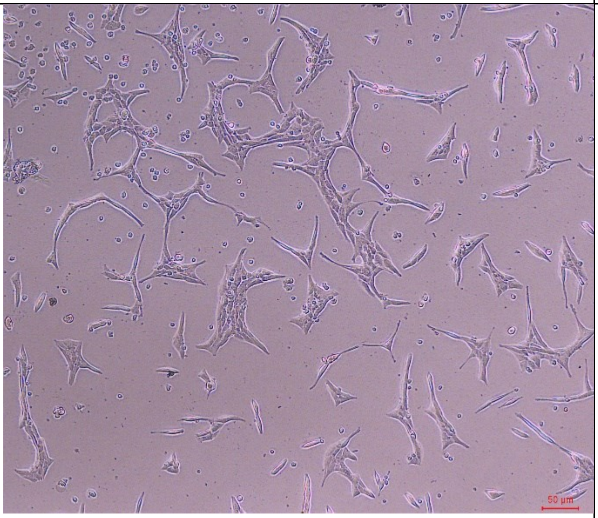<br>F1	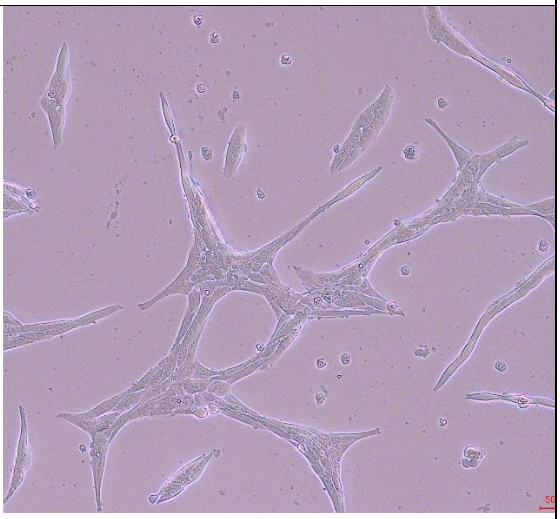<br>F2

**$[MoO_2(HL^2)]$, 100 µM**<br><br>G1,G2. Significant decrease in cell confluence, change in the shape of the cells, among which there are elongated. Presence of a significant quantity of severely damaged cells which are round-shaped, detached and floating.	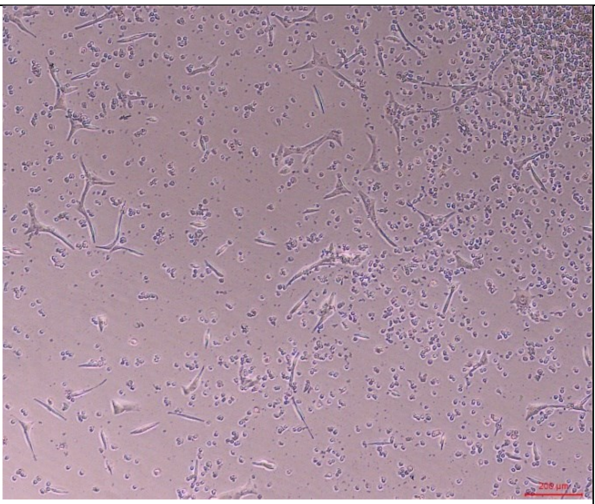<br>G1	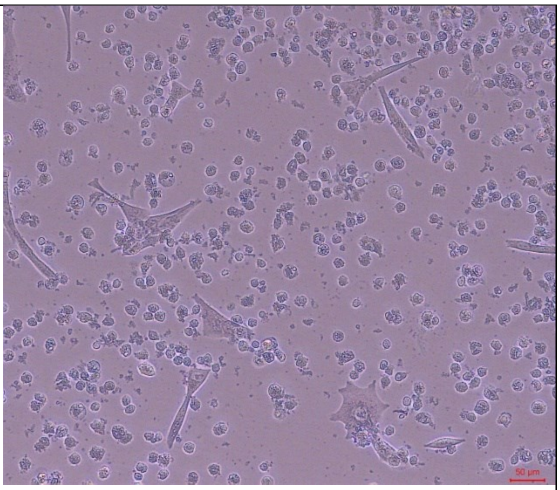<br>G2
