# Supplementary material for: Structure-Dependent Cytotoxic and Cytoprotective Effects of Molybdenum(VI) Schiff Base Complexes in the Neuronal Cells In Vitro
Source: Molecules. 2026 Aug 18;31(16):2883. doi: 10.3390/molecules31162883 (PMC13515801; doi:10.3390/molecules31162883)
Supplement: Supplementary file 1 [file molecules-31-02883-s001.zip › SUPPLEMENTARY FILE S5.pdf]

Supplementary file 5.

The effects of [$MoO_2(HL^3)$] on HT-22 cells viability in the media with the different levels of glucose

5.1. The effects of $[MoO_2(HL^3)]$ *per se* on HT-22 cells viability in the medium with glucose level 25 mM (24 and 48 hours)

<table>
<tr><th>Control cells,
glucose level in medium 25 mM</th><th colspan="2">Cells incubated with $[MoO_2(HL^3)]$,
25 µM, glucose level in medium 25 mM</th></tr>
<tr><td colspan="3">24 hours</td></tr>
<tr><td>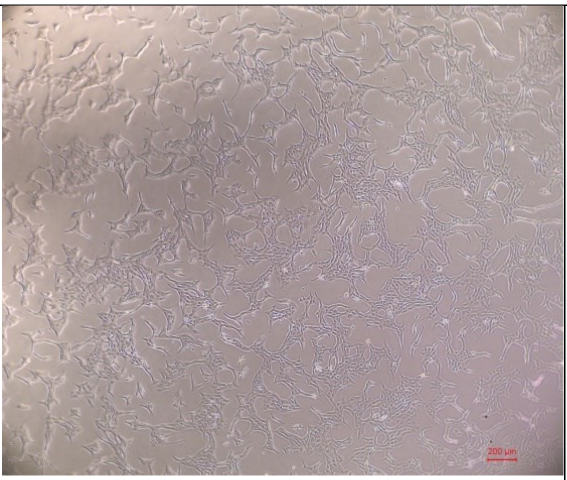
A1
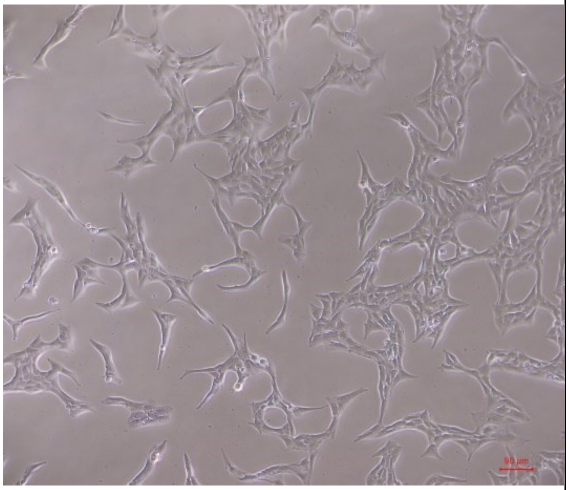
A2</td><td>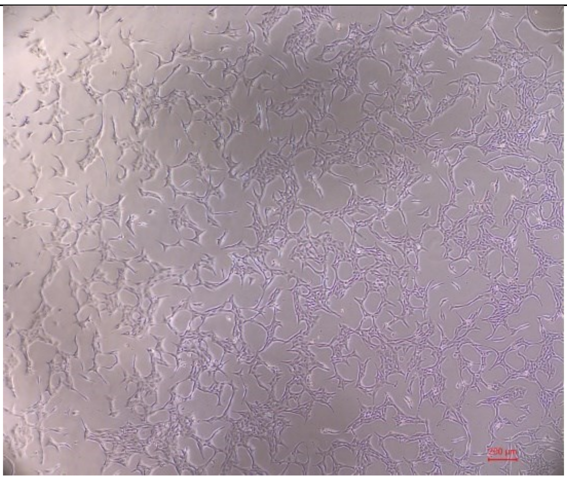
B1
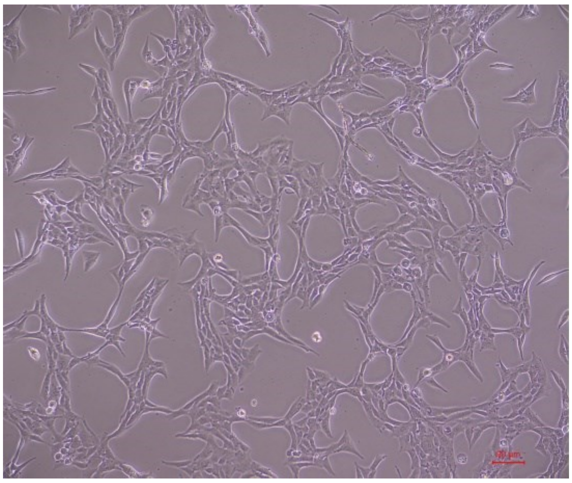
B2</td><td>A1,A2,B1,B2. Typical morphological structure of HT-22 cells, which are attached, the cells shape is unchanged.
Sporadic single round-shaped cells are visible.
There are no significant changes in cell confluence, cell shape and visible structures in cells incubated with the compound 13 compared with the microphotographs of the control cells.</td></tr>
<tr><td colspan="3">48 hours</td></tr>
<tr><td>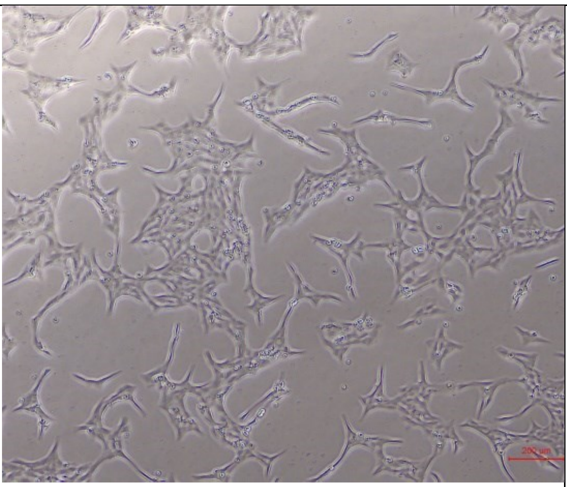
C1</td><td>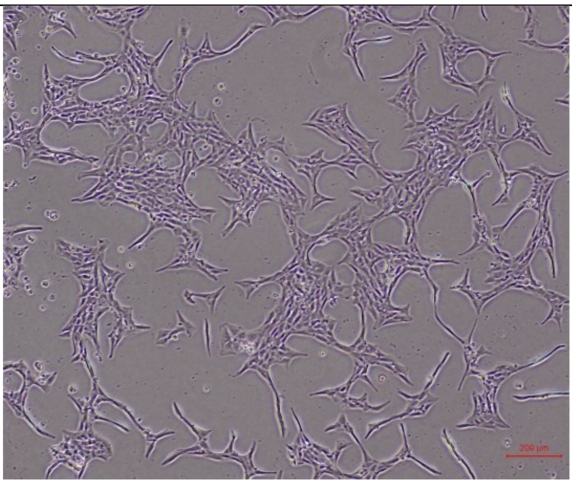
D1</td><td>C1,C2,D1,D2. Typical morphological structure of HT-22 cells, which are attached, the cells shape is unchanged.
Sporadic single round-shaped cells are visible.
There are no significant changes in cell confluence, cell shape and visible structures in cells incubated with the compound</td></tr>
</table>

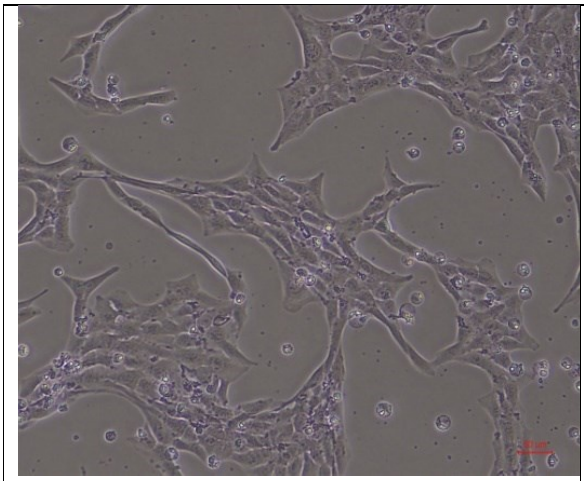 C2	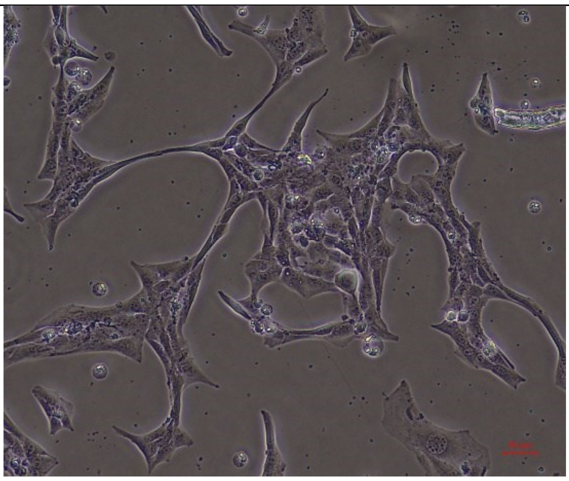 D2	13 compared with the microphotographs of the control cells.

5.2. The effects of $[MoO_2(HL^3)]$on HT-22 cells viability under the conditions of H_2O_2-induced cytotoxicity (500 µM) in the medium with glucose level 25 mM (24 and 48 hours)

**H_2O_2, 500 µM, glucose level in medium 25 mM**	**$[MoO_2(HL^3)]$, 25 µM<br>+ H_2O_2, 500 µM, glucose level in medium 25 mM**	
**24 hours**		
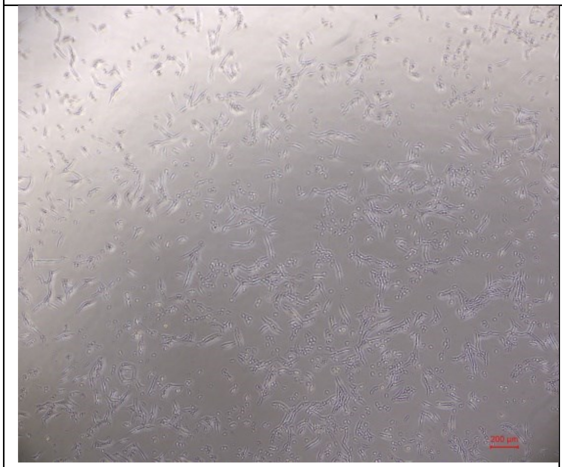<br>A1<br>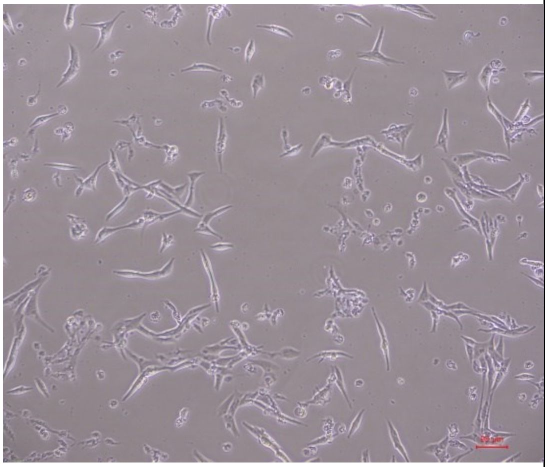<br>A2	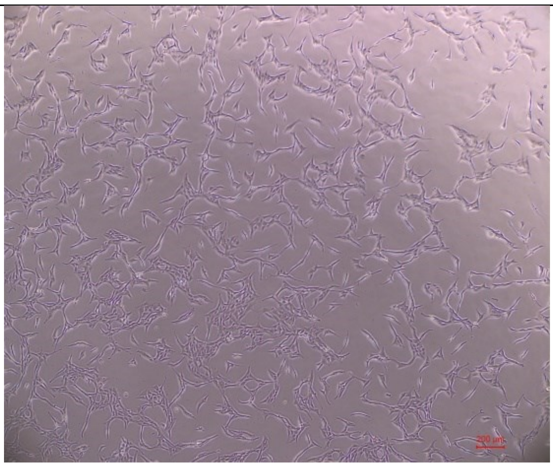<br>B1<br>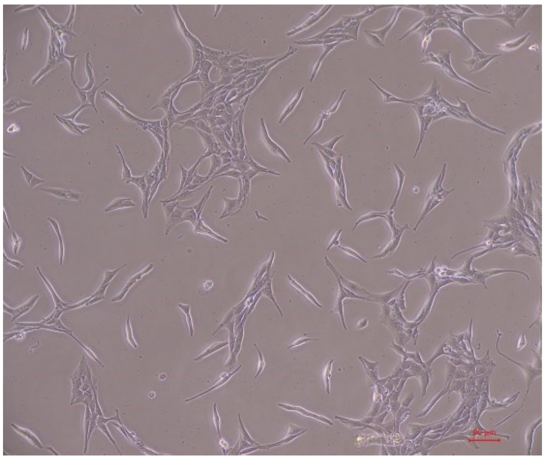<br>B2	A1,A2. Significant reduction in the quantity of visible attached cells and a considerable quantity of damaged cells, which are round-shaped, detached and floating.<br>B1,B2. Moderate reduction in the quantity of visible attached cells. Sporadic damaged cells, which are round-shaped, are visible.<br>Cell confluence and shape are significantly restored compared with the microphotographs A1,A2 (untreated cells injured with hydrogen peroxide).
**48 hours**		
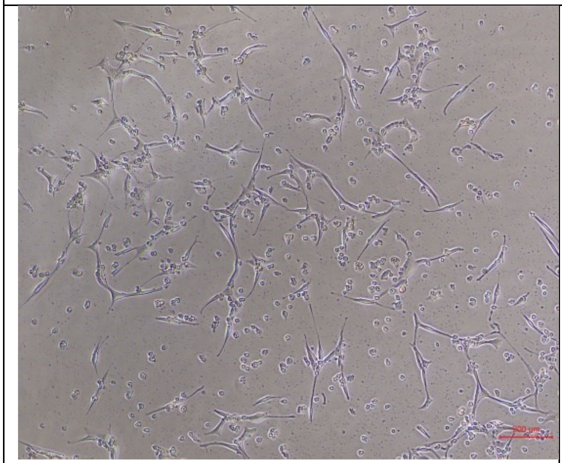<br>C1	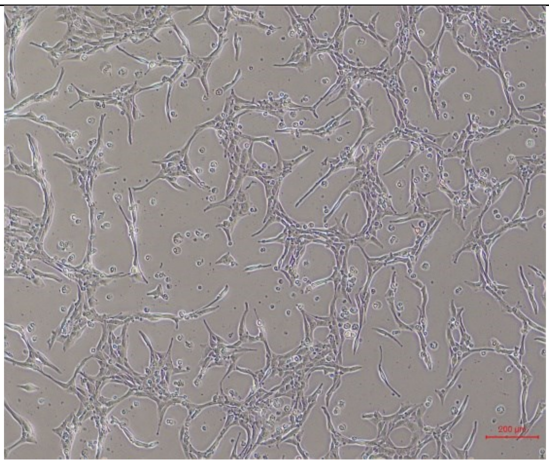<br>D1	C1,C2. Significant reduction in the quantity of visible attached cells and a considerable quantity of damaged cells, which are round-shaped, detached and floating.<br>D1,D2. Moderate reduction in the quantity of visible attached cells. Sporadic damaged cells, which are

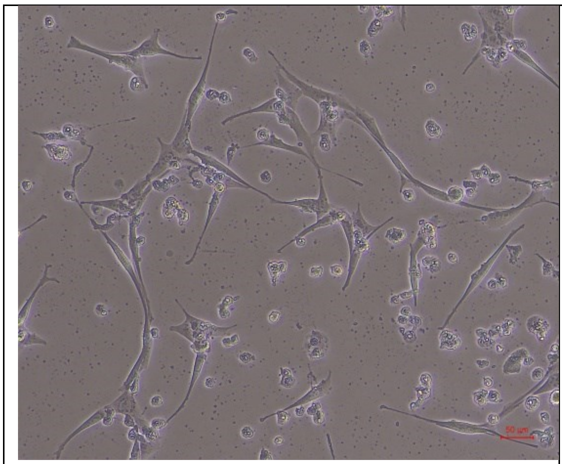 C2	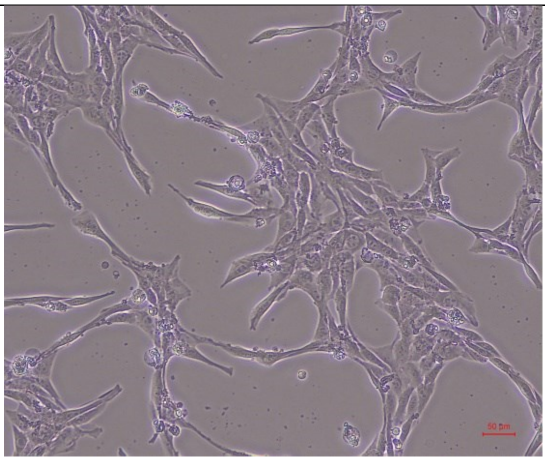 D2	round-shaped, are visible. Cell confluence and shape are significantly restored compared with the microphotographs C1,C2 (untreated cells injured with hydrogen peroxide).

5.3. The effects of $[MoO_2(HL^3)]$ *per se* on HT-22 cells viability in the medium with glucose level 50 mM and the corresponding osmotic control (25 mM glucose + 25 mM mannitol), incubation for 48 hours

<table>
<tr><td>Control cells,
glucose level in the medium
50 mM</td><td>Cells incubated with
$[MoO_2(HL^3)]$, 25 µM, glucose level in the medium 50 mM</td><td rowspan="5">A1,A2,B1,B2,C1,C2,D1,D2. Typical morphological structure of HT-22 cells, which are attached, the cells shape is unchanged.

Cell confluence in the subgroups of extremely high glucose content (50 mM) is moderately decreased (A1,B1) compared with the corresponding group of osmotic control (C1,D1).

There are no significant changes in cell confluence, the cells shape and their visible structures in cells incubated with the compound 13 compared with the microphotographs of the control cells both in the subgroups of extremely high glucose content (50 mM) as well as in the corresponding group of osmotic control.</td></tr>
<tr><td>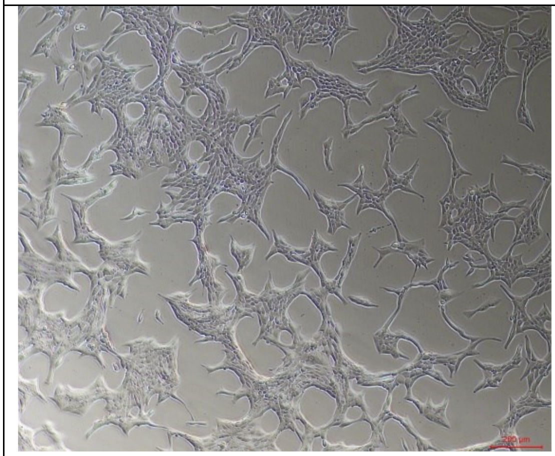
A1</td><td>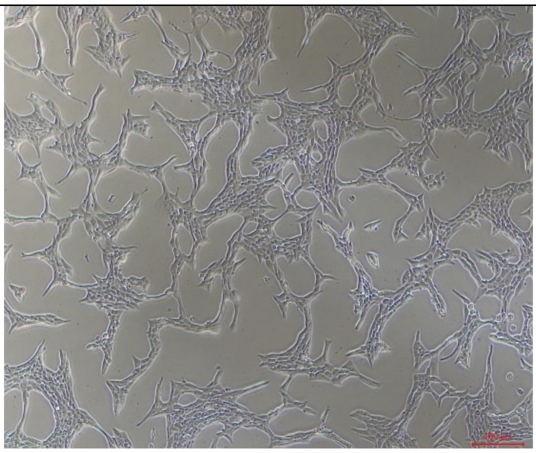
B1</td></tr>
<tr><td>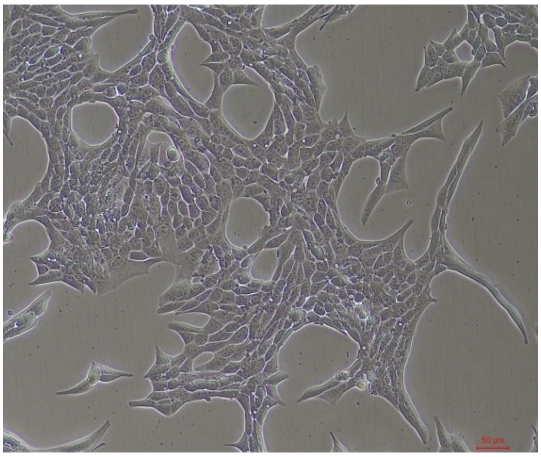
A2</td><td>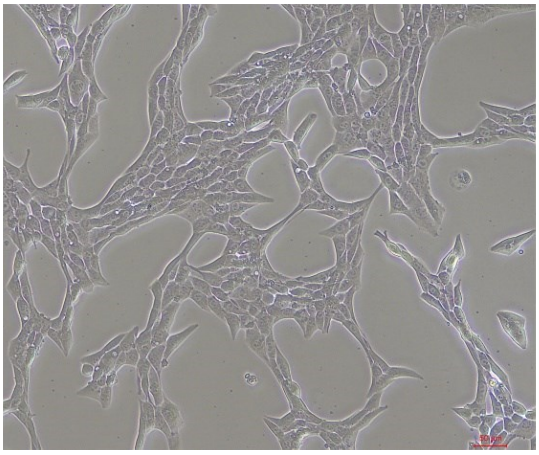
B2</td></tr>
<tr><td>Control cells, “osmotic control,” 25 mM glucose + 25 mM mannitol</td><td>Cells incubated with $[MoO_2(HL^3)]$, 25 µM, 25 mM glucose + 25 mM mannitol (“osmotic control”)</td></tr>
<tr><td>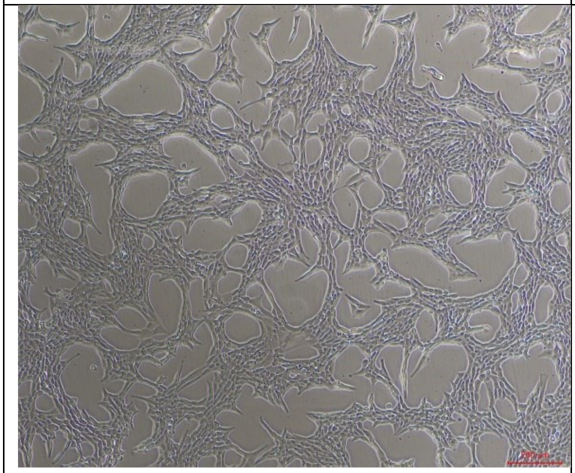
C1</td><td>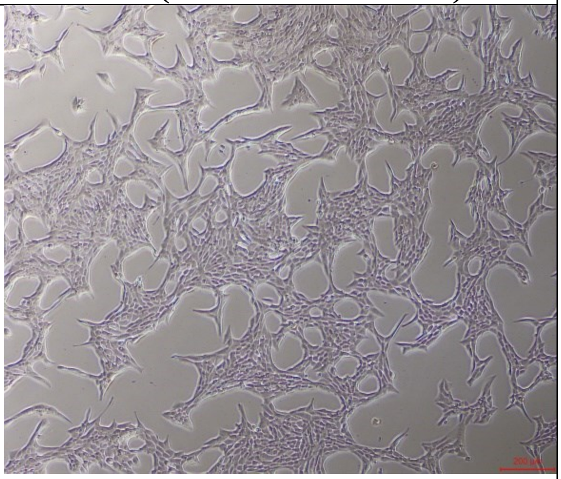
D1</td></tr>
</table>

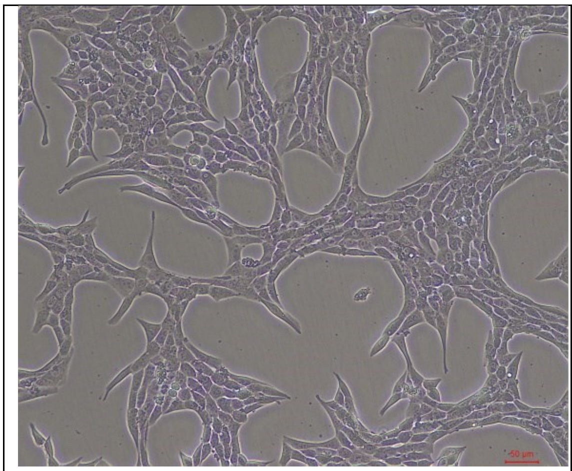 C2	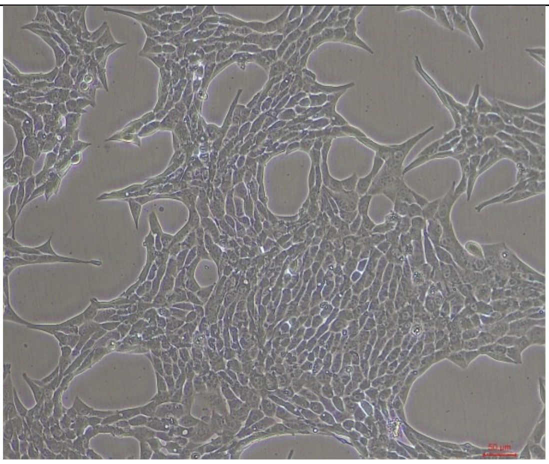 D2	

5.4. The effects of [$MoO_2(HL^3)$]on HT-22 cells viability under the conditions of H_2O_2-induced cytotoxicity (500 µM) in the medium with glucose level 50 mM and the corresponding osmotic control (25 mM glucose + 25 mM mannitol), incubation for 48 hours

**H_2O_2, 500 µM, glucose level in medium 50 mM**	**[$MoO_2(HL^3)$], 25 µM, + H_2O_2, 500 µM, glucose level in medium 50 mM**	
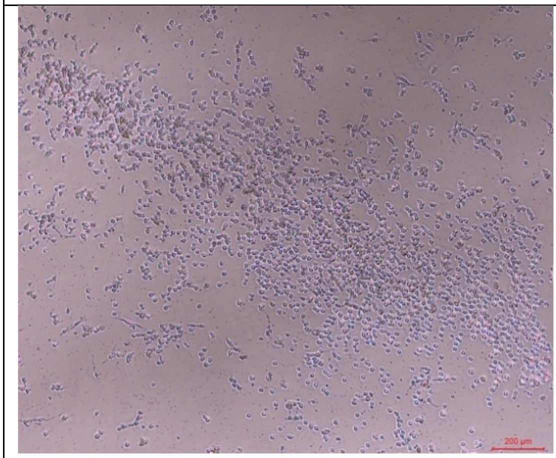<br>A1<br>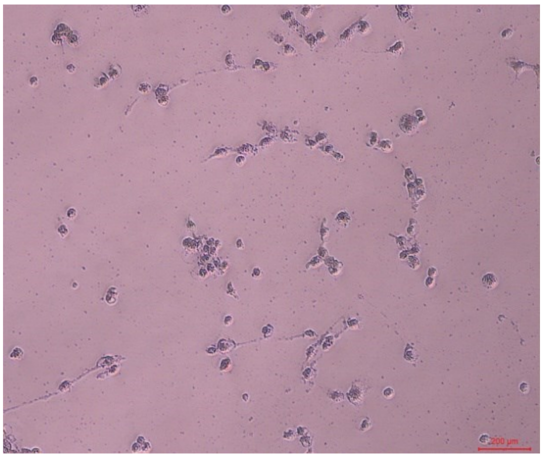<br>A2<br>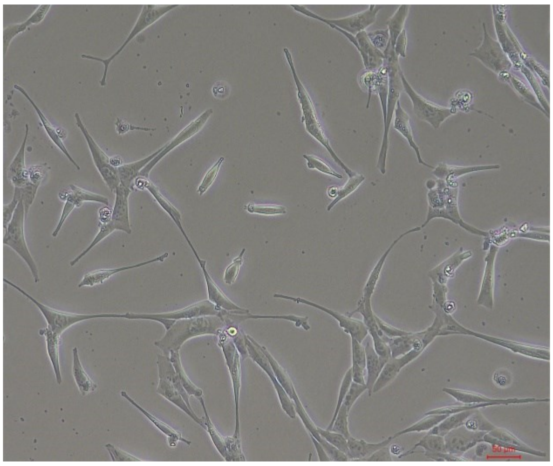<br>A3	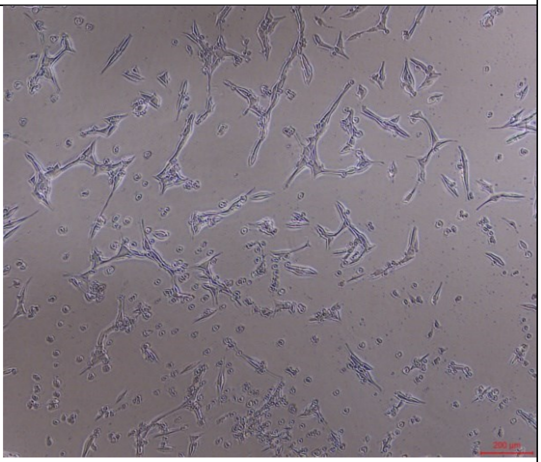<br>B1<br>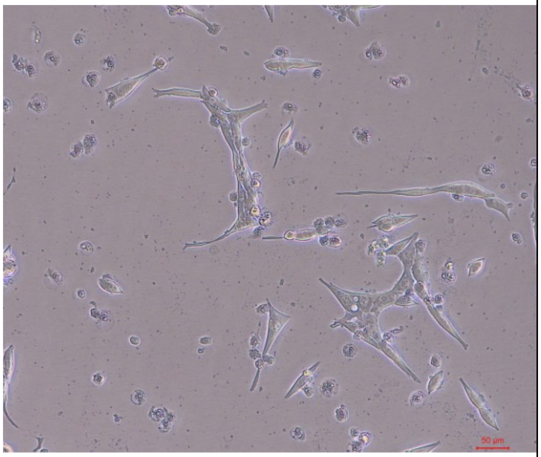<br>B2<br>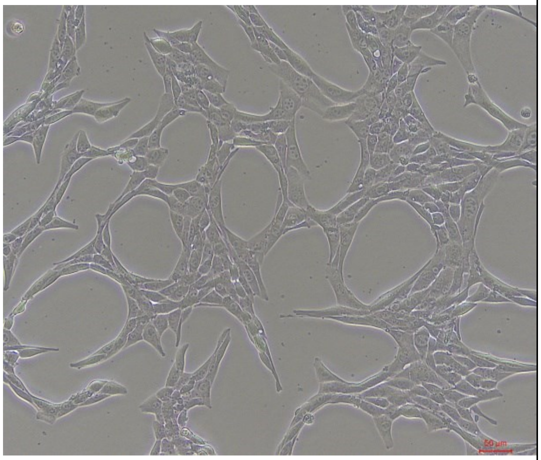<br>B3	A1,A2. Attached undamaged cells are practically absent. Plethora of the severely damaged cells and cell debris.<br><br>B1,B2. Significant reduction in the quantity of visible attached cells, change in the shape of the cells, among which there are elongated, small oval-shaped or small rhomboid. Presence of severely damaged cells which are round-shaped, detached and floating.<br><br>Note. Microphotographs A3,B3,C3,D3 represent the selected representative samples with better maintenance of cellular integrity.<br><br>A3. Significant decrease of cell confluence, change in the shape of the cells, among which there are elongated, small oval-shaped or small rhomboid.<br><br>B3. Moderate decrease of cell confluence.

**H_2O_2, 500 µM, “osmotic control,” 25 mM glucose + 25 mM mannitol**	**[$MoO_2(HL^3)$], 25 µM, + H_2O_2, 500 µM, “osmotic control,” 25 mM glucose + 25 mM mannitol**	
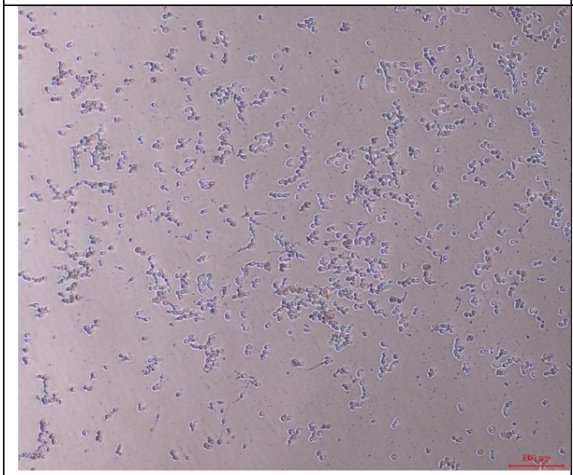<br>C1<br>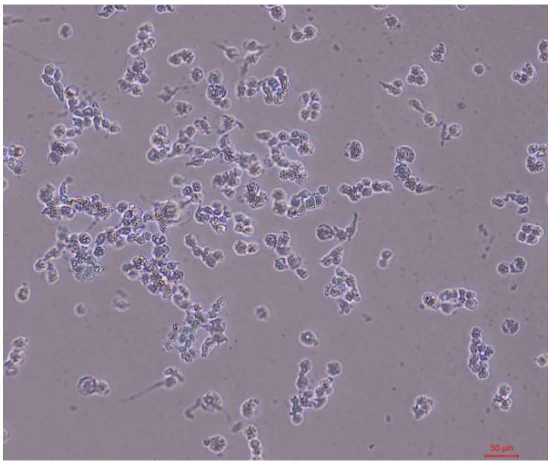<br>C2<br>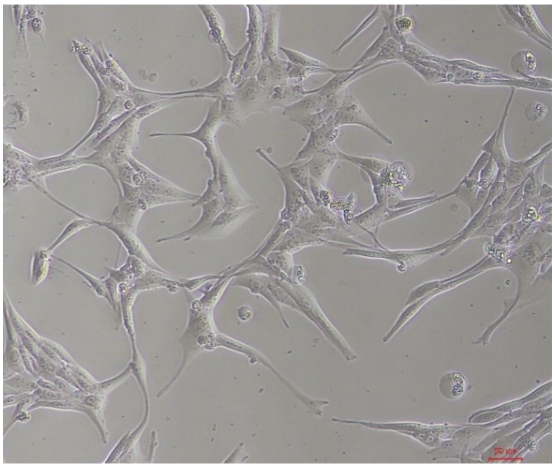<br>C3	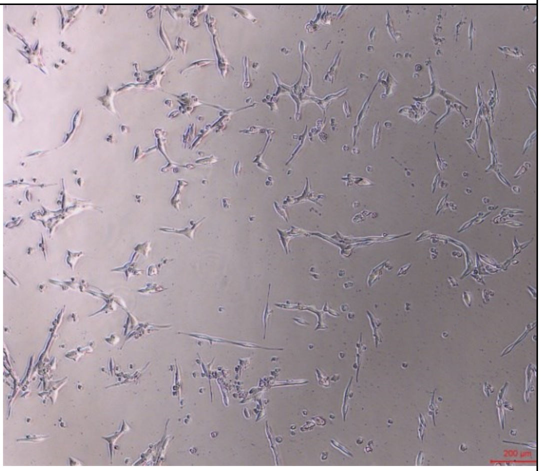<br>D1<br>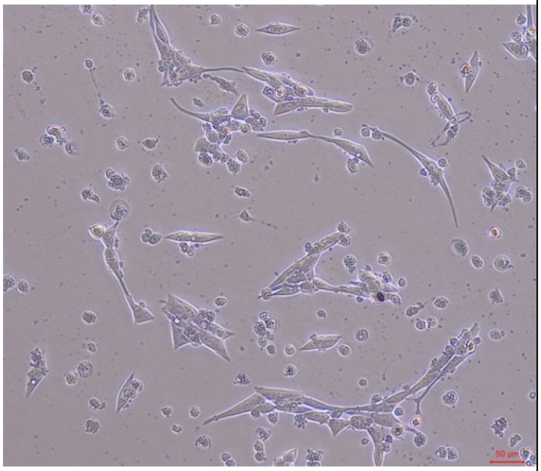<br>D2<br>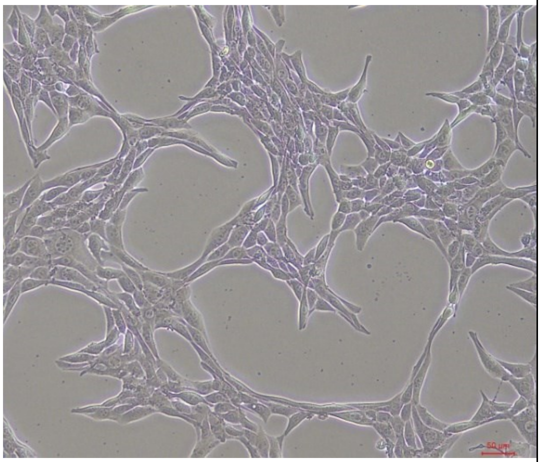<br>D3	C1,C2. Only sporadic attached cells are present. Plethora of the severely damaged cells and cell debris.<br>D1,D2. Significant reduction in the quantity of visible attached cells, change in the shape of the cells, among which there are elongated, small oval-shaped or small rhomboid. Presence of severely damaged cells which are round-shaped, detached and floating.<br><br>Note. Microphotographs A3,B3,C3,D3 represent the selected representative samples with better maintenance of cellular integrity.<br><br>C3. Significant decrease of cell confluence, change in the shape of the cells, among which there are elongated or round-shaped.<br>D3. Moderate decrease of cell confluence.<br><br>Generally, in the microphotographs of the cells incubated with the compound 13, less significant changes in cell confluence and shape are visible compared with the microphotographs of the control cells both in the subgroups of extremely high glucose content (50 mM) as well as in the corresponding group of osmotic control.
