# Supplementary material for: Structure-Dependent Cytotoxic and Cytoprotective Effects of Molybdenum(VI) Schiff Base Complexes in the Neuronal Cells In Vitro
Source: Molecules. 2026 Aug 18;31(16):2883. doi: 10.3390/molecules31162883 (PMC13515801; doi:10.3390/molecules31162883)
Supplement: Supplementary file 1 [file molecules-31-02883-s001.zip › SUPPLEMENTARY FILE S6.pdf]

Supplementary file 6.

The effects of [$MoO_2(HL^3)$] *per se* on the viability of SH-SY5Y cells

6.1. Representative microphotographs of control SH-SY5Y cells and the cells incubated with [$MoO_2(HL^3)$] (10 µM) for 24 hours

**Control**<br><br>A1,A2. Typical morphological structure of the control cells, the cells shape is unchanged. Sporadic single round-shaped cells are visible.	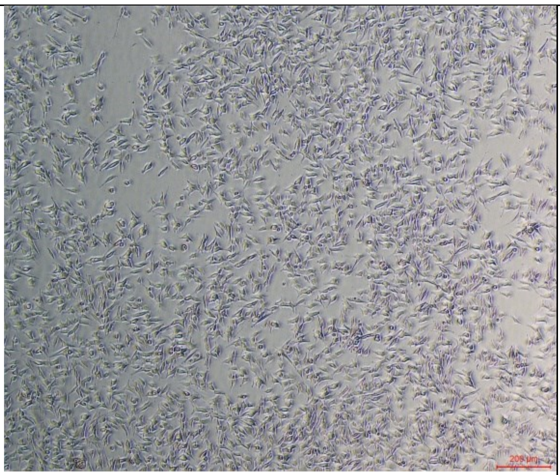<br>A1	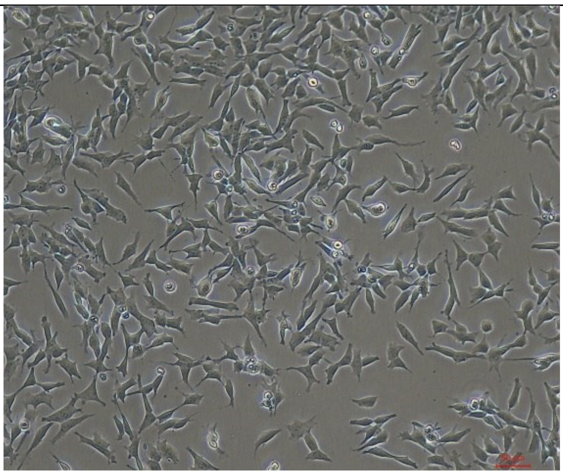<br>A2
**[$MoO_2(HL^3)$], 10 µM**<br><br>B1,B2. The typical morphological structure of the cells is maintained, the cells shape is unchanged, there are no visible changes in cell confluence compared with the microphotographs of the control cells.	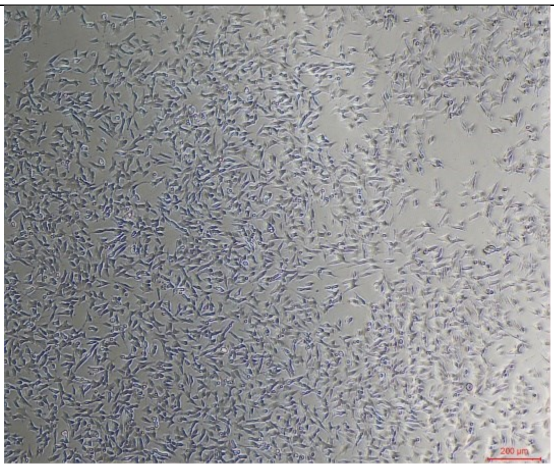<br>B1	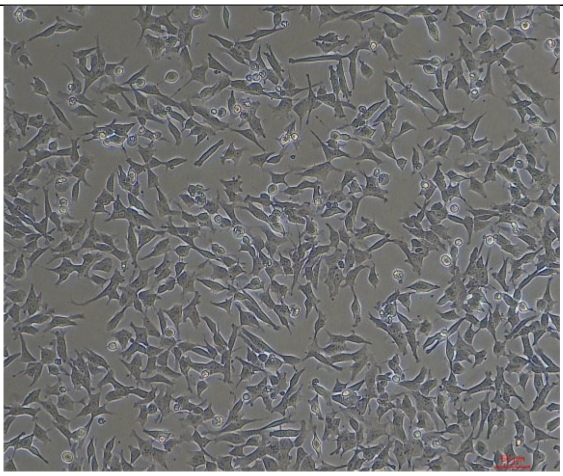<br>B2

6.2. Representative microphotographs of control SH-SY5Y cells and the cells incubated with $[MoO_2(HL^3)]$ (25 µM; 50 µM; 100 µM) for 24 hours

**Control**<br><br>C1,C2. Typical morphological structure of the control cells, the cells shape is unchanged. Sporadic single round-shaped cells are visible.	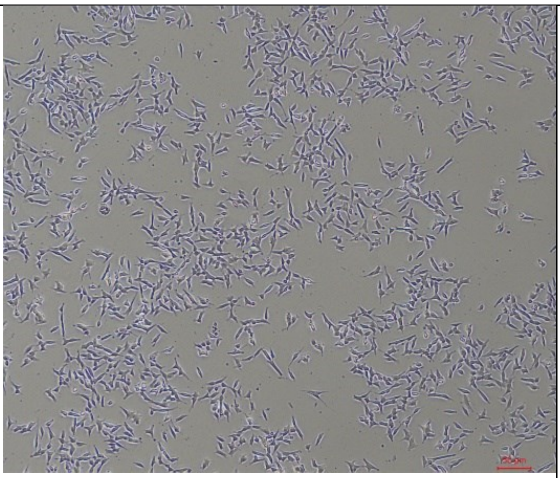<br>C1	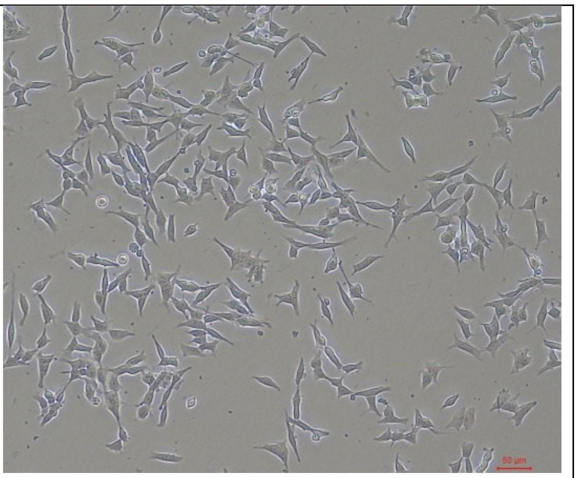<br>C2
**$[MoO_2(HL^3)]$, 25 µM**<br>D1,D2. The typical morphological structure of the cells is maintained, the cells are attached, there are no visible changes in cell confluence compared with the microphotographs of the control cells. Slightly increased quantity of the single round-shaped cells visible at high magnification (D2 compared with C2).	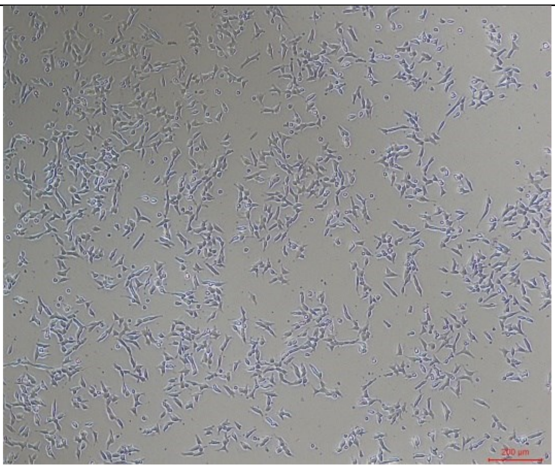<br>D1	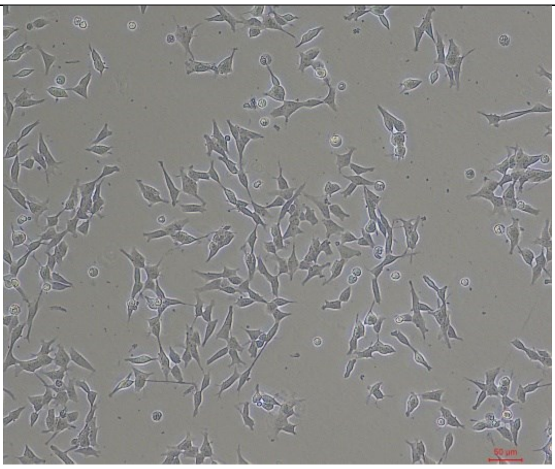<br>D2

**$[MoO_2(HL^3)]$, 50 µM**<br><br>E1,E2. The typical morphological structure of the cells is maintained, increased quantity of round-shaped, detached cells.	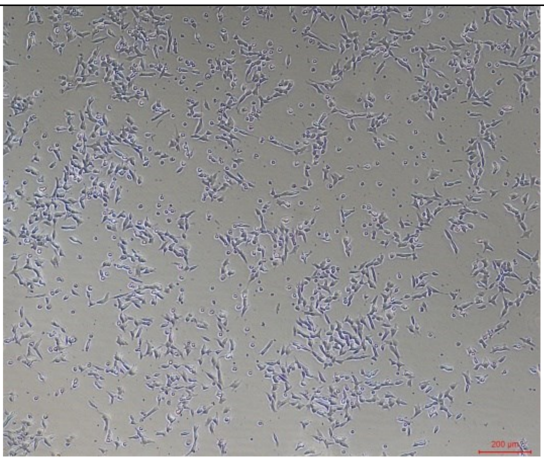<br>E1	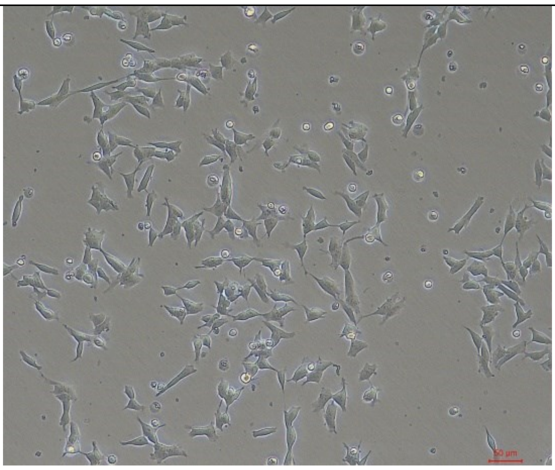<br>E2
**$[MoO_2(HL^3)]$, 100 µM**<br><br>F1,F2. The typical morphological structure of the cells is maintained, increased quantity of round-shaped, detached cells, this increase is more significant compared with other microphotographs (F2 vs. E2).	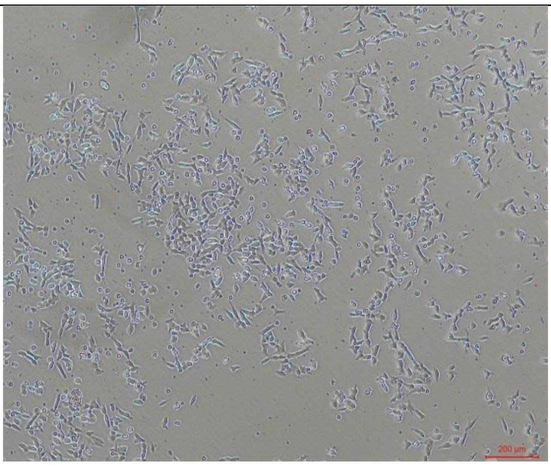<br>F1	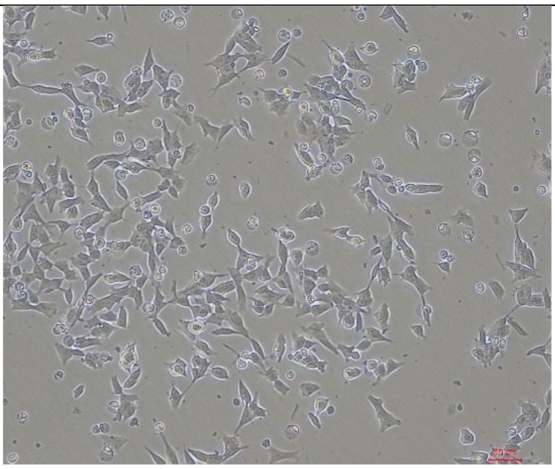<br>F2
