# Supplementary material for: Structure-Dependent Cytotoxic and Cytoprotective Effects of Molybdenum(VI) Schiff Base Complexes in the Neuronal Cells In Vitro
Source: Molecules. 2026 Aug 18;31(16):2883. doi: 10.3390/molecules31162883 (PMC13515801; doi:10.3390/molecules31162883)
Supplement: Supplementary file 1 [file molecules-31-02883-s001.zip › SUPPLEMENTARY FILE S7.pdf]

Supplementary file 7.

The effects of [$MoO_2(HL^3)$] on the viability of SH-SY5Y cells under the conditions of H_2O_2-induced cytotoxicity

7.1. Representative microphotographs of control SH-SY5Y cells and the cells under the conditions of H_2O_2-induced cytotoxicity incubated with $[MoO_2(HL^3)]$ (10 μM; 25 μM) for 24 hours

**Control**<br><br>A1,A2. Typical morphological structure of the control cells, the cells shape is unchanged. Sporadic single round-shaped cells are visible.	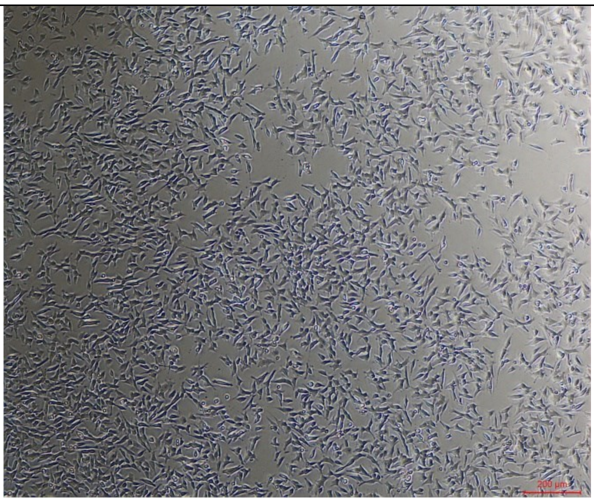<br>A1	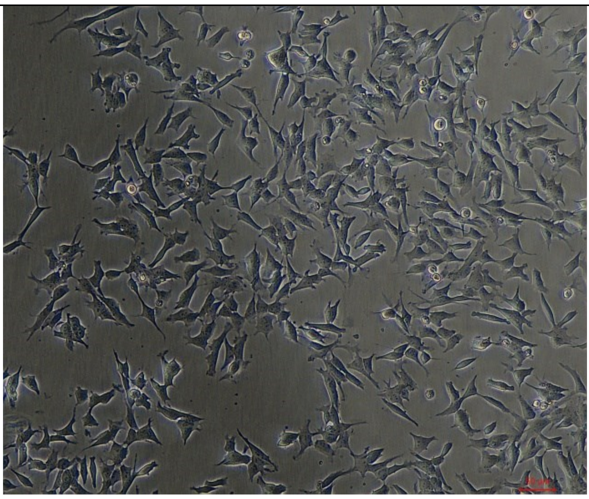<br>A2<br>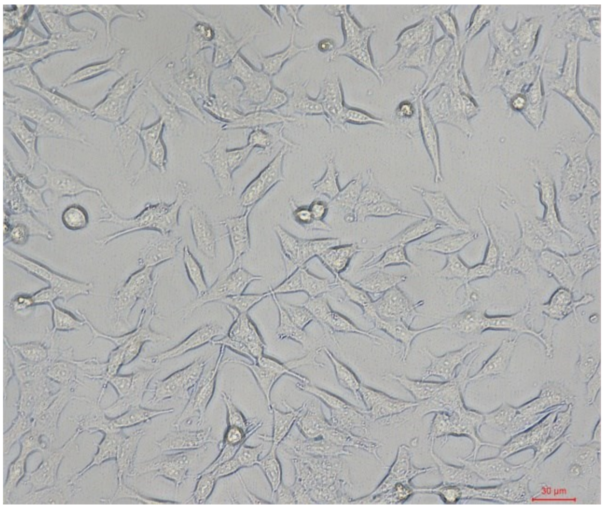<br>A3
**H_2O_2, 175 μM**<br><br>B1,B2,B3. Significant reduction in the quantity of visible attached cells and an increase in the quantity of detached round-shaped cells (B1). Significant quantity of the cells with the different degrees of injury up to	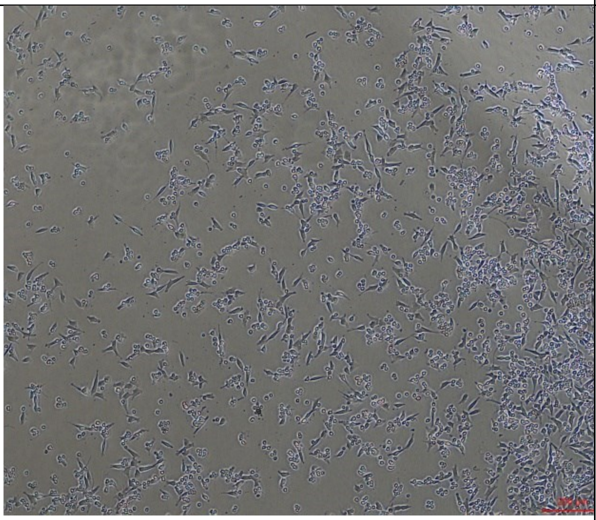<br>B1	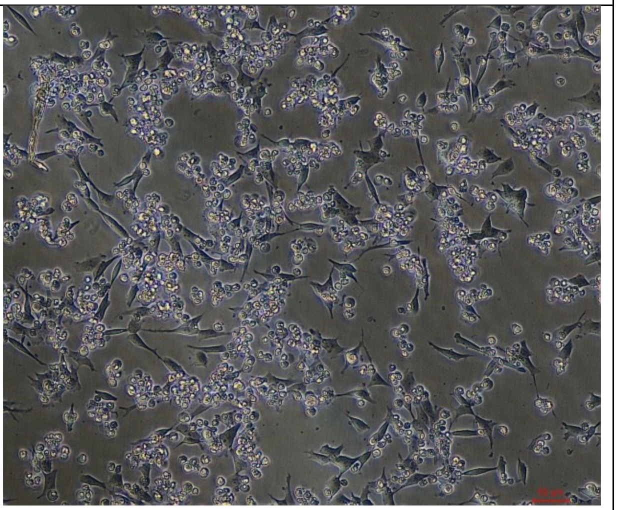<br>B2

cell debris (B2,B3).		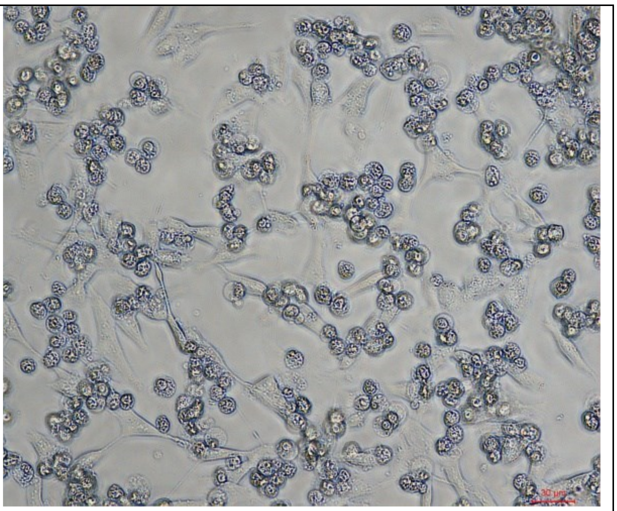<br>B3
**$[MoO_2(HL^3)]$, 10 µM + H_2O_2, 175 µM**<br><br>C1,C2,C3. Significant reduction in the quantity of visible attached cells and presence of detached round-shaped cells (C1). Significant quantity of the cells with the different degrees of injury up to cell debris (C2,C3), still the quantity of these cells is decreased compared with the samples B2,B3.	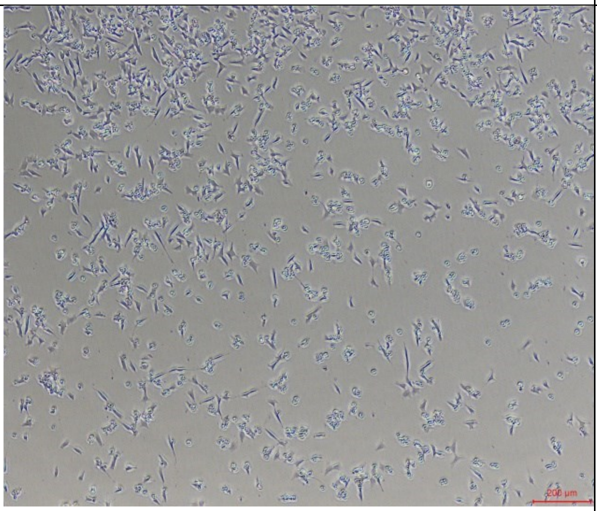<br><br>C1	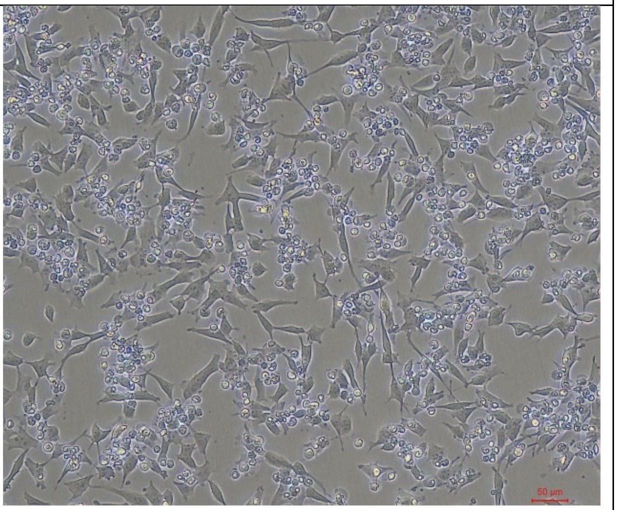<br><br>C2<br>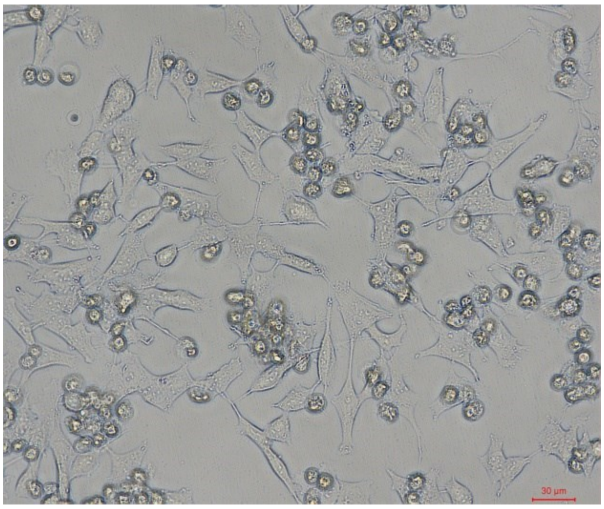<br><br>C3

**[$MoO_2(HL^3)$], 25 µM + H_2O_2, 175 µM**<br><br>D1,D2,D3. The typical morphological structure of the cells and cell confluence are maintained, presence of single round-shaped cells in moderate quantity and sporadic fragments of cell debris.	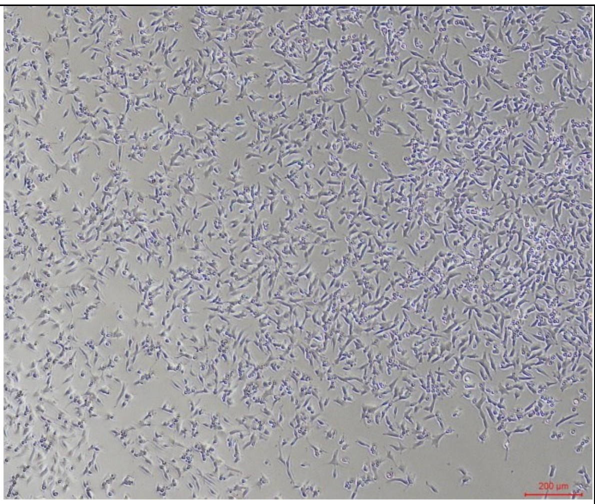<br>D1	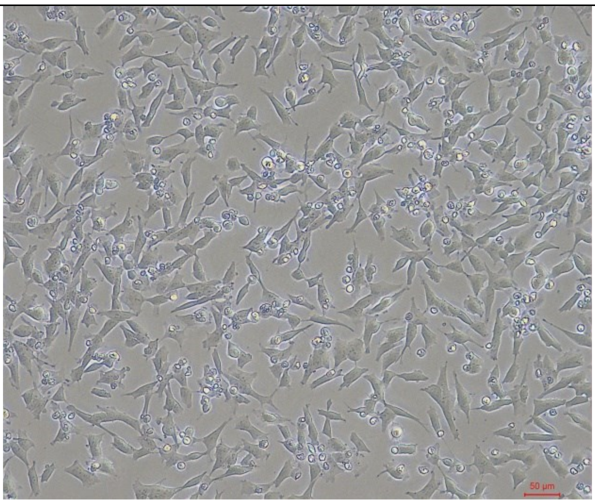<br>D2<br>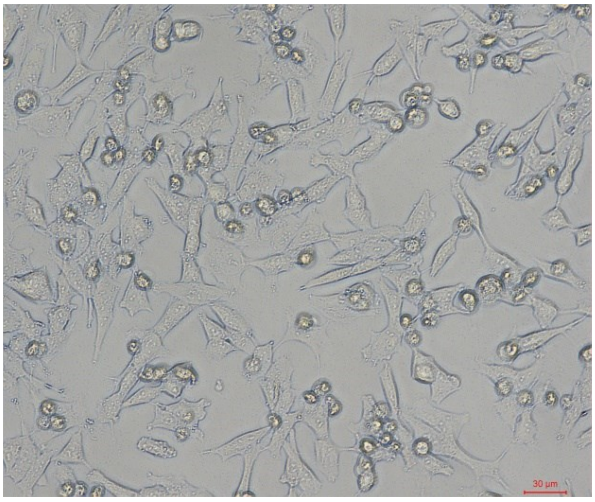<br>D3

7.2. Representative microphotographs of control SH-SY5Y cells and the cells under the conditions of H_2O_2-induced cytotoxicity incubated with $[MoO_2(HL^3)]$ (50 µM) for 24 hours

**Control**<br><br>D1,D2. Typical morphological structure of the control cells, the cells shape is unchanged. Sporadic single round-shaped cells are visible.	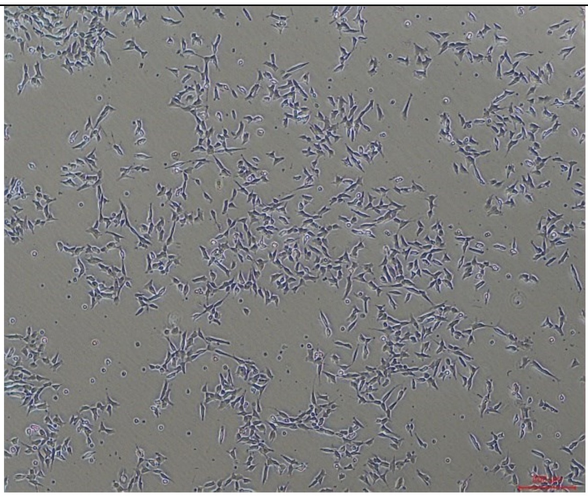<br>D1	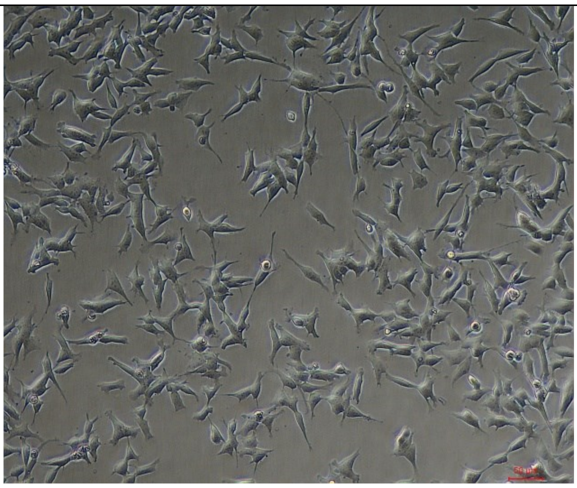<br>D2
**H_2O_2, 175 µM**<br><br>E1,E2. Significant reduction in the quantity of visible attached cells, change in the shape of the cells, which are elongated, presence of round-shaped, severely damaged cells as well as cell debris.	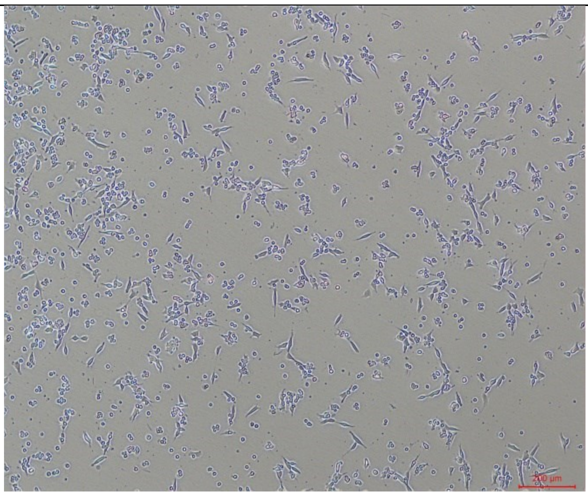<br>E1	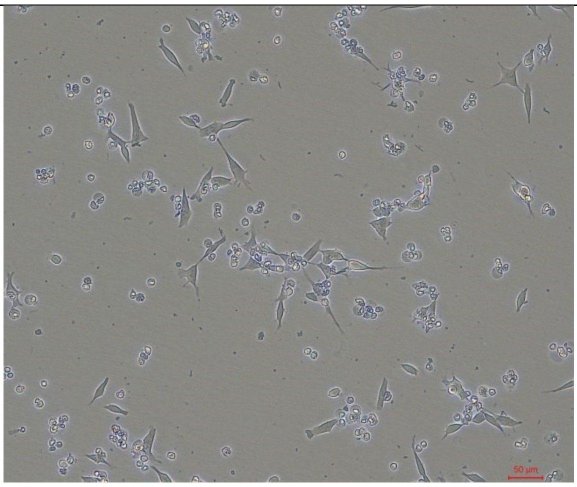<br>E2
**$[MoO_2(HL^3)]$, 50 µM + H_2O_2, 175 µM**<br><br>F1,F2. Significant reduction in the quantity of visible attached cells, presence of round-shaped, severely damaged cells as well as cell debris. Cell confluence is	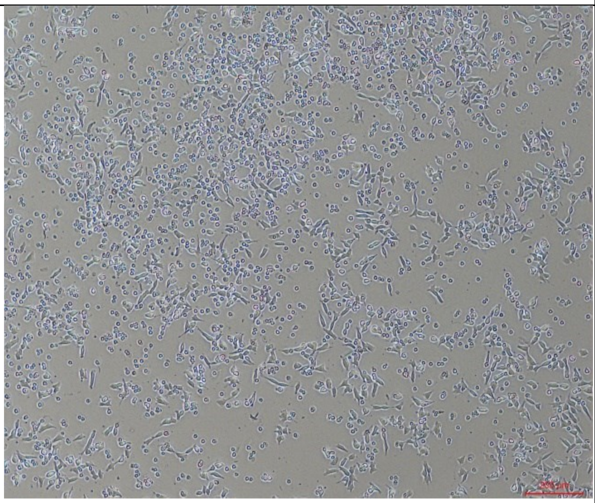<br>F1	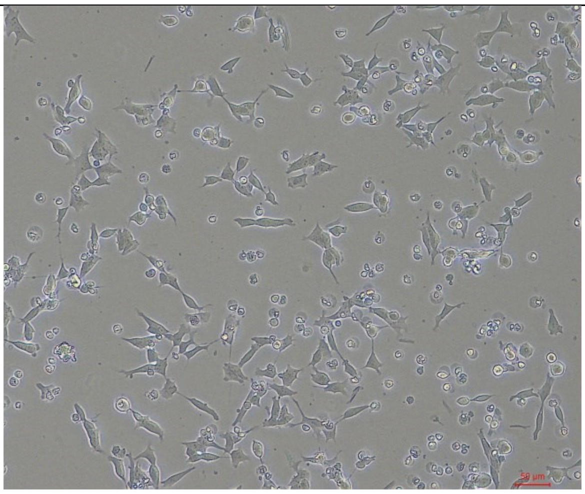<br>F2

higher compared with the microphotographs E1,E2, still the quantity of round-shaped, severely damaged cells is not reduced and even slightly increased.		
